# Employing machine learning for reliable miRNA target identification in plants

**DOI:** 10.1186/1471-2164-12-636

**Published:** 2011-12-29

**Authors:** Ashwani Jha, Ravi Shankar

**Affiliations:** 1 Studio of Computational Biology & Bioinformatics, Biotechnology Division, Institute of Himalayan Bioresource Technology, Council of Scientific & Industrial Research (CSIR), Palampur 176061 (HP), India

## Abstract

**Background:**

miRNAs are ~21 nucleotide long small noncoding RNA molecules, formed endogenously in most of the eukaryotes, which mainly control their target genes post transcriptionally by interacting and silencing them. While a lot of tools has been developed for animal miRNA target system, plant miRNA target identification system has witnessed limited development. Most of them have been centered around exact complementarity match. Very few of them considered other factors like multiple target sites and role of flanking regions.

**Result:**

In the present work, a Support Vector Regression (SVR) approach has been implemented for plant miRNA target identification, utilizing position specific dinucleotide density variation information around the target sites, to yield highly reliable result. It has been named as p-TAREF (plant-Target Refiner). Performance comparison for p-TAREF was done with other prediction tools for plants with utmost rigor and where p-TAREF was found better performing in several aspects. Further, p-TAREF was run over the experimentally validated miRNA targets from species like *Arabidopsis*, *Medicago*, Rice and Tomato, and detected them accurately, suggesting gross usability of p-TAREF for plant species. Using p-TAREF, target identification was done for the complete Rice transcriptome, supported by expression and degradome based data. miR156 was found as an important component of the Rice regulatory system, where control of genes associated with growth and transcription looked predominant. The entire methodology has been implemented in a multi-threaded parallel architecture in Java, to enable fast processing for web-server version as well as standalone version. This also makes it to run even on a simple desktop computer in concurrent mode. It also provides a facility to gather experimental support for predictions made, through on the spot expression data analysis, in its web-server version.

**Conclusion:**

A machine learning multivariate feature tool has been implemented in parallel and locally installable form, for plant miRNA target identification. The performance was assessed and compared through comprehensive testing and benchmarking, suggesting a reliable performance and gross usability for transcriptome wide plant miRNA target identification.

## Background

miRNAs have emerged as a major regulatory components of cell system, which are active in almost all of the multicellular organisms. These noncoding RNA elements are around 21 bp long and bind the target mRNA sequences which share complementarity with the targeting miRNA sequences. However, for a long time it has been believed that miRNA targeting in plants requires almost complete complementarity while in animal it is incomplete complementarity where seed regions play the critical role in binding and subsequent targeting [1,2]. Some recent studies have emerged out where translational repression and some inexact complementarity have been suggested to be existent in plant miRNA targeting too [3-5]. Some groups, encouraged with these findings, have started looking into such aspects in more detail, studying interactions which may not display exact complementarity as well as instances which are left undetected by existing plant miRNA target prediction tools [5,6]. Li *et al *conducted an experiment, where they suggested that complementarity and homology based target identification tools, which compose the major approach of target identification in plants, may miss out several valid targets in plants. Such targets actually may not obey conservation, homology or exact complementarity [7]. The major drawbacks of most of the existing plant miRNA target prediction tools have been that they follow the exact complementarity, most of them do not consider any flanking region sequence contribution to better the target prediction, they hardly leverage from machine learning like powerful approaches to handle multiple features for target prediction more accurately. Most of them lack the realistic time approach to handle the genome or transcriptome wide data to facilitate faster target predictions as most of them are serially coded and web-server based. A major reason could be a predominant belief that unlike animal system, targeting in plants has been not much complex. Pertaining to this, exact complementarity search centered tools were used for plant target predictions while animal target identification witnessed large number of innovations [8]. Few of the most frequently used plant miRNA target prediction tools relied strongly upon exact pattern search and local alignments. PatScan [9] was a tool developed to look for exact similar matching patterns for target, where users could modify the match and mismatch values as well as select for wobble. However this tool did not consider bulge or seed specific scoring and its use has been nonspecific as it is used for other pattern match based purposes too, besides target finding. Another tool, miRNAassist, used BLAST search for complementary regions of miRNAs [10]. Using BLAST, already known miRNAs from other species were used as a database to search against *Brassica *EST sequences. Following almost similar approach, Carrington group proposed another protocol where BLAST was replaced by FASTA34 [11]. They also introduced some scoring rules of alignment to separate the seed region from rest of the regions as well as relaxed values for mismatches and wobbles. However BLAST based approaches are good for instances where the query length is longer as for smaller sequences, hits come up with very low significance making a random hit case. Considering this Zhang [12] developed a new tool, miRU, which replaced BLAST with Smith-Waterman local alignment, weighting more for seed regions and allowed bulges. These all tools were centered around complementarity search. Acknowledgement for limitations of exact complementarity and alignment based methods was conspicuous with release of new generation tools like TAPIR [13]. TAPIR worked with two different options: 1) Scan for targets using FASTA program based alignment or 2) By applying more sensitive approach of running RNAhybrid [14] and considered thermodynamic and mismatch factors together. Use of RNAhybrid in the back-end also ensured that unlike previously employed tools, TAPIR was able to detect multiple target sites in a given mRNA sequence. Contemporary to this, Xie and Zhang developed a novel tool Target-align [15]. Target-align was implemented by considering some rules while performing alignments. These rules were about the number of allowed mismatches, consecutive mismatches, number of allowed gaps and strict mismatch conditions in the seed region. However unlike TAPIR, focus of Target-align was on Smith-Waterman based alignment for complementarity search with several conditions. An advantage with Target-align has been its availability as local standalone version, unlike majority of plant miRNA target identification tools. Very recently, Dai *et al *acknowledged about the various lacunae in existing plant miRNA target identification tools, including centrality of alignment based approach, no proper consideration for imperfect complementarity, no consideration for role of flanking regions, inability to detect multiple sites as well as unavailability of locally downloadable standalone version to perform large and genomic scale studies [16]. Considering the various existing demerits, this group implemented the role of target site accessibility and flanking regions by using RNAup [17]. RNAup is a tool to predict RNA-RNA interaction, considering single strandedness of a given RNA sequence while deriving partition function for various nucleotides in secondary structures. RNAup and similar approaches have been used frequently in animals for miRNA target identification with likes of Sfold [18], PITA [19] and MicroTAR [20]. However, applications of such tools have some limitations, as they are based on single sequence secondary structure and energy based features, whose accuracy and reliability drop drastically with increase in the length of sequences [21,22]. Considering this, Heikham and Shankar [23] had proposed a novel approach to consider the flanking region sequence information, bypassing the chances of getting trapped into the issues arising from limitations of thermodynamics and structure based modeling. It successfully applied varying dinucleotide density profile with respect to putative target positions to decipher the role of flanking region in miRNA targeting in animal system. In case of plants, considering such approach becomes more relevant as unlike animals, where targeting is preferred in the 3' UTR regions, in plants miRNA targeting can occur to any region of the full length mRNA.

In the present work, these findings have been extended with flanking regions sequence information role in determining miRNA targets [23], by applying and assessing the theory on plant system too. Here a machine learning based reliable approach with multiple features oriented statistical learning has been applied, having a clear edge over rule based approaches. *Arabidopsis thaliana *has been used as the source to derive plant specific features which were modeled using Support Vector Regression to classify as well as to implement an effective scoring scheme through regression score. Besides this, a concurrent architecture with multi-threads has been implemented, making the tool application easily deployable even on simple desktop machine in concurrent mode, enabling it to scan plant mRNA sequences for targets in transcriptome wide manner.

## Implementation

### Basic working approach

**Figure 1:**
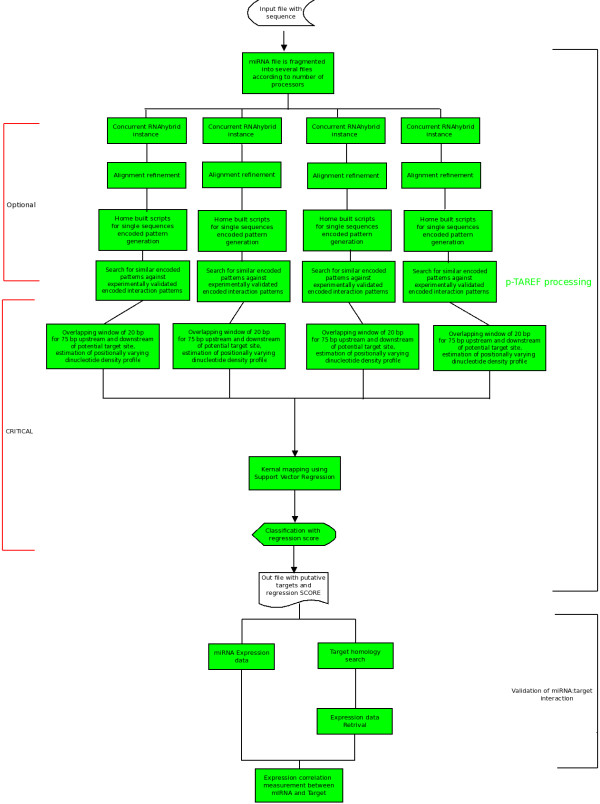
**p-TAREF workflow**. The figure illustrates the various working stages involved in p-TAREF along with concurrency.

p-TAREF has been designed specifically to detect plant miRNA targets, applying the following basic steps: A) Conversion of target:miRNA pairing into single dimension encoded pattern for interactions, which retains the various interaction features combinations found in plant system. This is done for experimentally validated as well as predicted RNA:miRNA interactions. An uniform alignment step precedes it to maintain a common alignment approach B) Using target:miRNA binding thermodynamic, implemented through RNAhybrid, initial set of targets are generated. This is followed by an optional filtering step. The library of experimentally known encoded patterns is scanned against the predicted interaction patterns as combinations of match, mismatch, wobble and bulge may hold interaction state information for target:miRNA C) Evaluation of putative miRNA target site as a potential target site based on plant specific flanking region dinucleotide density profile variation in position specific manner with respect to the possible target site. It uses multivariate classifier with capability to transform between non-linear and linear spaces. When applied with Support Vector Regression, the position specific dinucleotide density profile variation patterns were found to possess strong discriminative power to precisely classify targets and non-targets. Dinucleotide density variation pattern also retains nearest neighbor information for nucleotides, a property useful in determining the accessibility and structural conditions of RNAs. The entire process has been implemented in a parallel mode. Figure 1 shows the working implementation of p-TAREF along-with concurrency. The following sections give more details about the implementation of the entire approach.

### Sequence data

The present work has used several sequence resources. miRNA sequences for plants were downloaded from Mirbase version 16 [24]. 243 mature miRNA sequences were retrieved for *Arabidopsis*, 414 for *Oryza*, 234 for *Populus*, 51 for *Medicago *and 37 for Tomato. All these miRNAs have been integrated in the presented tool. Experimentally validated *Arabidopsis thaliana *miRNA targets and their corresponding targeting miRNAs were retrieved from ASRP database [25] as well as from the list of miRNA:target pairs validated through RACE PCR as reported in the supplementary material provided by Beauclair *et al *[5]. *Arabidopsis *sequences were downloaded from TAIR, version 10. Experimentally validated targets for *Medicago *and Rice were retrieved from various literatures [7,26]. Negative instances of false targets were built from the dataset used previously as well as random sequences [13,23].

### Plant specific encoded interaction pattern generation

Instances were extracted, using the list of RACE PCR validated miR:target interactions for *Arabidopsis*, submitted by Beauclair *et al *[5] in their supplementary material. Experimentally validated miRNA and target interactions for other plants species like Rice, *Medicago*, Tomato, *Populus*, were also derived from various literatures [7,26,27]. All miRNAs and target partners were retrieved for a separate run of RNAhybrid. RNAhybrid predicts miRNA:Target interaction by considering thermodynamic parameters for interactions and multiple-sites while applying information from statistical distribution in its backdrop. Also RNAhybrid run is a common step between encoded interaction pattern generation for experimentally validated instances as well as during the prediction run over any unknown query sequences. This way, it maintains a common approach. Output of RNAhybrid over experimental datasets provided exact binding pictures of interactions, which was further refined by applying Needleman-Wunch global alignment algorithm based local alignment tool, Stretcher, from Emboss-package. In order to consider the G:U wobble, the scoring matrix was adjusted accordingly with +1 advantage for G:U wobble, gap opening penalty of -15 and extension penalty of -5. Through this, sequence similarity as well as thermodynamic considerations was implemented to derive the interaction patterns. Using local scripts, all such interactions were converted into single encoded patterns, where information was reduced to single dimension alone, with match states of nucleotides i.e. bulge on miRNA strand, bulge on target strand, mismatch, match and wobble. All experimentally validated interactions were finally represented into only this form. Same protocol was used by the tool to generate interaction patterns for the predicted targets automatically. For every predicted target, the entire library of experimentally validated encoded patterns is scanned for similarity with scope to look for inexactness. This step defines the primary filtering step based on similarity of interaction patterns with experimentally known interactions. At present, total 268 different interaction patterns have been included considering miRNA:target interaction cases from *Arabidopsis *(157), *Medicago *(7), *Populus *(42), Tomato (11) and Rice (51).

### Support Vector Regression (SVR) model building for plants

Unlike rule based approaches of identification and classification, machine learning approaches have emerged much superior for the process of classification. Among them, Support Vector Machine (SVM) has appeared as highly reliable one as it can handle large number of features together to derive a suitable classifier using multivariate statistical learning, which is comparatively tough to achieve by rule based approach of classification. Another advantage of SVM has been that unlike other machine learning approaches it concentrates upon evolving a classifier boundary with maximum margins, lowering the chance of misclassification and error drastically. This property is also controlled by the type of kernel selected for training and classification purpose, as linear kernel applies linear boundary, Gaussian kernel applies normal distribution boundary while polynomial kernel has capability to evolve convolute boundary to handle the cases where instances from different classes are very mixed up for the given set of features. The final classification by SVM assigns the classified instances their respective class as either 1, 0 or -1. However, this does not come with any clear confident value for the classification. This degree of confidence could be derived through some scoring scheme, which is provided by the Support Vector Regression (SVR). In the current study, a more evolved Support Vector approach, the SVR, has been used to implement training and classification along with a scoring scheme, regressions score. For training purpose, a sequence dataset comprising 104 experimentally validated *Arabidopsis *sequence instances reported by Beauclair *et al *[5] (Supplementary Material, 2010) as well as negative target instances used by Heikham and Shankar [23] was formed. The negative target sequences has randomly generated sequences as well as some experimentally validated negative targets which were predicted as targets but experimentally validated as false positives. 75 bases flanking regions around the target sites in negative as well as positive instances are considered through 20 bases long sliding windows, estimating the dinucleotide density and its variations with respect to the target-site. Discrimination through dinucleotide density variation with respect to position was found to be the best for window size of 20. Mean distribution based feature selection procedure was applied to learn about the most discriminating features in plants. The Support Vector Regression Machine was applied through SVMTorch [28], where every learning instance was converted into position specific dinucleotide density variation profile with respect to the (possible) target sites. Training and model generation were performed separately for three different Kernel classes: Linear, Gaussian and Polynomial. The best emerging models for plant systems for each Kernel class were saved and integrated into the plant target identification tool developed. This way the user gets three choices of plant models to select from.

### Expression data support integration and visualization

Various array expression experiments and data (Affymetrix Rice Genome Array, Affymatrix Arabidopsis Tilling Array 1.0 R and AT-TAX) were used in the present study. Data normalization was done using gcRMA method implemented in "R" Statistical Package. The expression data ('.CEL' format) was downloaded from GEO for *Oryza sativa*. Expression studies and data for 17 *Oryza *miRNA families (156, 159, 160, 166, 168, 172, 396, 444, 528, 806, 810, 820, 1318, 1875, 2055, 2906, 395) and 57,359 RNA sequences (excluding miRNAs) were used. For *Arabidopsis *miRNAs, the available expression related studies and data for 31 miRNA families (156, 157, 159, 163, 164, 165, 166, 167, 169, 171, 172, 319, 390, 391, 393, 394, 396, 398, 399, 401, 403, 404, 405, 406, 407, 413, 414, 417, 447, 824, 834) and 30,166 mRNA transcripts were considered. For several of these array based experimental data, RT-PCR based validations for sets of associated representative genes were reported by the submitting authors. The RNA sequences for *Arabidopsis *were downloaded from TAIR and *Oryza *RNA sequences from RiceGE.

To calculate correlation coefficient, the submitted target(s) is first searched in the locally installed database of *Oryza *or *Arabidopsis *(to be opted by the user) using BLASTn. The top most hit amongst all the hits, found by BLASTn, is extracted. The identifier of best hit is scanned across the inbuilt library of expression data files to finally calculate the Pearson Correlation Coefficient for co-expression. Modules for scanning and data parsings for expression correlation analysis part were implemented through codes developed in PERL, PHP and Java. miRNA:target association graph was generated using graphviz and Java libraries, JgraphT and JGraph.

### Introduction of Concurrency

Concurrency enables the system to perform the same task with higher speed by harnessing the available logical processors on a given machine. Currently, even a simple desktop or laptop comes with multicore CPUs, having two or more processors/cores, which can go upto more than 50 in current generation servers. Implementation of concurrency was done using Java Concurrent Library (JCL) while applying multi-threaded processing of tasks. The developed tool provides the user an option to select the total number of processors to be used for target scanning. Accordingly, multithreads are created to process the query sequences. A single query sequence is chopped into several small subsequences with minimum 50 bp length (considering that usually a miRNA:target interaction stays below 50 bp), in overlapping manner and distributed across the number of processors selected, to run the following steps of target identification. For every such processor and batch of allocated sequences, RNAhybrid is run separately; output is manipulated and parsed for coordinates, separately and concurrently. Similarly, the alignment step is run concurrently. Only the Support Vector Regression step is not concurrent as it is quite faster. The RNAhybrid, alignment, parsing and union steps are quite time consuming and application of concurrency saves the time by providing manifolds acceleration while performing analysis on large amount of data.

### Standalone and Server Implementation

The entire tool has been developed as a web-server as well as Linux based standalone GUI version. The web-server version has been developed using Linux-Apache-PHP, along with concurrency. The standalone version has core programs and scripts written in Python, PERL, Java and C, while its GUI wrapper has been developed using QT C++ GUI library. The standalone version, too, supports concurrency.

### Performance measurement

Six major different tests were done to assess the performance of the developed tool, p-TAREF, for miRNA target-identification: 1) Testing for performance on dataset containing training set (total 104 positive and 119 negative instances) 2) Dataset containing 287 *Arabidopsis *positive instances from ASRP database. 3) Dataset containing experimentally validated targets from Rice, *Medicago*, *Populus *and Tomato 4) Comparison of p-TAREF with TAPIR and Target-align, for performance over TAPIR/Target-align reference dataset 5) Performance comparison between p-TAREF, Target-align and psRNATarget [29]. 6) Comparison between Target-align and p-TAREF for time performance on a given set of sequences. The performance measure terms, Sensitivity (Sn), Specificity (Sp), Accuracy (Ac) and Mathew Correlation Coefficient (MCC) were calculated using the following equations:

$$\text{Sn }=\text{ TP}∕\left(\text{TP }+\text{ FN}\right)$$

$$\text{Sp }=\text{ TN}∕\left(\text{TN }+\text{ FP}\right)$$

$$\text{Ac }=\text{ TP }+\text{ TN }∕\left(\text{TP}+\text{TN}+\text{FP}+\text{FN}\right)$$

$$\text{MCC}=\;\left\{\left(\text{TP}*\text{TN}\right)\;-\left(\text{FP}*\text{FN }\right)\right\}∕\text{ SQRT}\left\{\left(\text{TN}+\text{FN}\right)\left(\text{ TN}+\text{FP}\right)\left(\text{ TP}+\text{FN}\right)\;\left(\text{TP}+\text{FP}\right)\right\}$$

ROC curve based on 10 fold cross validation was done to estimate the performance and robustness of the classifier models and associated tests.

### Gene Ontology and enrichment studies

Gene Ontology information for Rice transcriptome was derived from Ensemble Plants. Enrichment analysis for gene categories predominant in miRNA target system was conducted through two different ways: A) Using multiple Binomial tests. B) Using Hyper-geometric exact tests. The null hypothesis was derived using the distribution of various GO categories and their terms in whole transcriptome of rice. For multiple Binomial tests, we developed in-house script in "R", while hyper-geometric tests were conducted using Cytoscape module of Bingo [30].

## Result and Discussion

### Web interface of p-TAREF server and GUI Standalone

**Figure 2:**
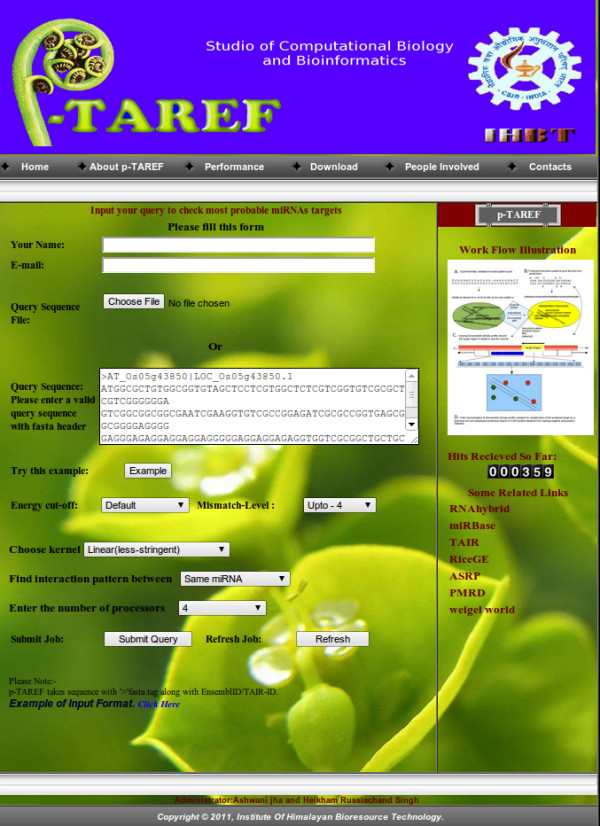
**The p-TAREF webserver**. The web-server provides a friendly interface to load query sequences, with various parameter settings which include selection of energy cut-off, mismatch level allowed, SVR Kernel to be used, number of processors to be used, etc. Its performance tab contains detailing about all performance measures done for p-TAREF performance benchmarking and comparison with other tools.

p-TAREF comes as a server as well as standalone version. The web-server takes single as well as batch mode submission of the query sequences. However, considering the connectivity dependence upon network, it is quite advisable to use the web-server version for single sequence or small number of sequences. The input of sequence requires FASTA manner entry where the first line starts with ">" followed by "AT" and accession ID or numeric digits to identify the sequence, without any gap, followed by next line having the sequence. Query could be either pasted directly or uploaded through some text file. The users are given with three choices 1) Type-I: Just submit the query sequence and run the tool from beginning, starting from RNAhybrid step. 2) Type-II: Submit the target mRNA sequence along-with predicted target sequence. 3) Type-III: Choose some miRNA from a drop down menu to identify targets on the submitted query sequences. Type-I facilitates the user to perform all tasks on the given query sequence, while Type-II is more for confirmation and validation of already predicted target by some other method, applying support vector regression module directly. Unlike Type-II, Type-I is more computationally intensive as it involves time consuming step of RNAhybrid, dynamic programming based alignment step, pattern encoding and search as well as large amount of parsing. Considering this, the option of concurrency has been given to the user for Type-I, where the user could choose the number of processors to be used to run the server concurrently and get results faster. Type-I also provides the user with options to select the allowed number of mismatches while estimating similarity between the predicted and experimentally validated encoded patterns for interactions between miRNA and targets. The maximum allowed level goes upto four mismatches. Higher the mismatch level cut-off, more number of total targets may emerge out. There is an option to set the threshold energy cut-off parameter for RNAhybrid run, which is -10 kcal/mol by default. A decisive step in parameter selection is the selection of plant model according to the Kernel (Choice of Kernel). Here, p-TAREF provides three options to choose from: Linear Kernel, Radial Basis Function (Gaussian) and Polynomial function. Linear function runs straight with least accommodative power, Gaussian is moderately stringent and Polynomial tries to cover more spread and deviating instances correctly. The Type-II option is more for validation purpose in case if a user wishes to confirm the predicted target by some other tool or method, by applying SVR approach. In this case, the user has to paste the predicted target region sequence as well as the sequence in which the target was predicted. Based upon the dinucleotide density profile variation method for refinement, SVR scores will be generated for the query. Figure 2 provides the look for Type-I form of the server. Besides this the server provides Type-II option to perform SVR validation for already predicted targets. It takes the predicted target sequence as well as the mRNA sequence, to which the target region belongs. Type-III option provides a list of miRNAs to opt from and perform analysis on the user submitted query sequence.

The server version also provides a provision to scan for possible expression data based expression correlation measurement for the given user query and associated miRNA, found targeting it. The user is asked to select the species to which the sequence belonged or is expected to share a homologous sequence. The server has inbuilt, normalized, expression data for plant miRNAs as well as genes, currently for *Arabidopsis *and Rice. Along-with the expression data, the associated mRNA sequences are also formated for similarity search tools like BLAST, which is enabled to run on multiple processors. The user opts for the species to be scanned for the target gene, in turn, the server preforms a BLAST run to consider the longest and most identical hit, most similar to the query sequence. The corresponding expression data for the target and targeting miRNA is retrieved for expression correlation measurement, which is displayed to the user. The publicly available expression data for all known plant miRNAs and genes will be continuously updated with every release and for various species. It needs to be mentioned that array expression data could be not of much use in case of translational repression by miRNA. A possible analogous facility may be provided in future for targeting cases where translational repression could be involved. The final output page displays the target sequence ID, targeting miRNA, the predicted interaction pattern and closest experimentally validated pattern along-with the partner miRNA, SVR score and choice to scan for expression analysis based validation across different species. The SVR score comes positive for potential miRNA targets while it is negative for non-targets. Higher the absolute value of the SVR score better is the confidence of classification.

**Figure 3:**
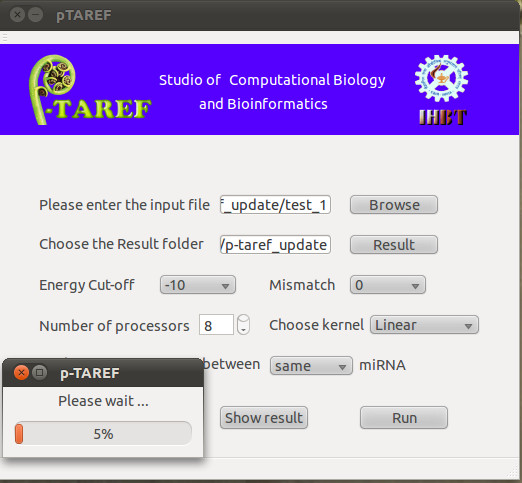
**Snapshot of standalone GUI version of p-TAREF**. Like its web-server counterpart, the standalone GUI version too provides concurrency and most of the features, enabling quick standalone scanning of batch and large amount of sequence data. It also shows a progress bar to intimate about the status of analysis.

The Standalone GUI version of p-TAREF was developed considering the realistic approach to scan large amount of transcriptome data for miRNA targeting. Performing such task on web-server is a time consuming approach which takes lots of time in loading the data itself and fetching it back, while both of the processes are connection and INTERNET availability dependent. In that way, Standalone GUI version could be very helpful in running p-TAREF locally and in user friendly manner. The entire interface has been developed using QT C++ Library which is also available for download from the server page's download section. The Standalone GUI is easily deployable even on a simple laptop or desktop machine as well as on high-end servers. In case, if the required dependencies are not pre-installed on user's machine, p-TAREF installation system verifies this and automatically installs all the per-requisites itself. The standalone GUI version provides an option to load sequence query file in a batch mode, adjust the mismatch level for experimentally validated interactions similarity search, options to adjust the binding free energy cut-off, options to choose for kernel dependent plant models as well as adjust the number of processors to be used to implement concurrency. A progress bar appears to display the running job status and amount of sequences scanned. Figure 3 shows the running state of Standalone GUI version of p-TAREF.

### Performance

**Figure 4:**
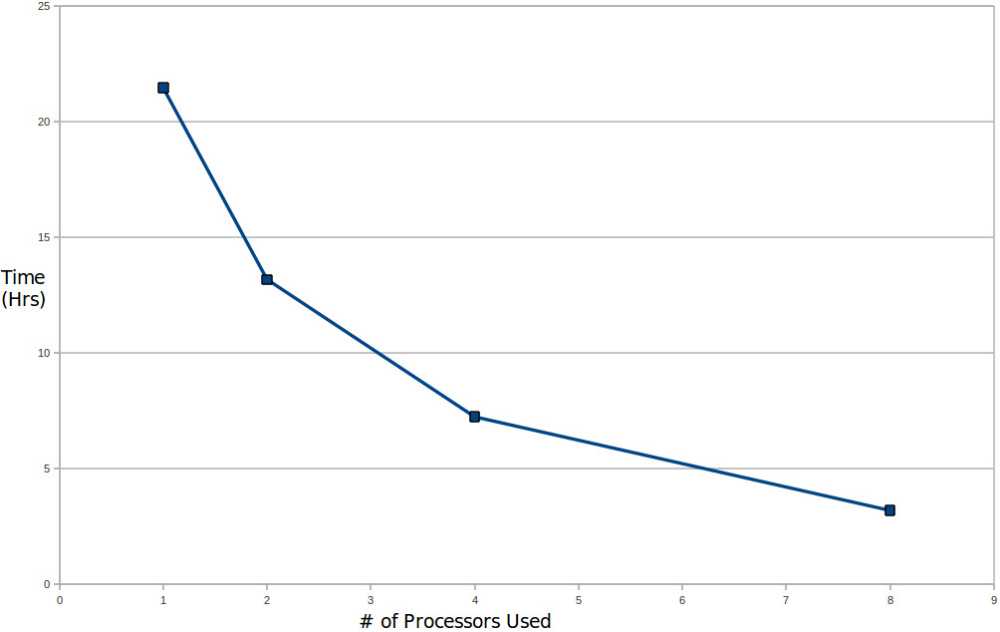
**Impact of concurrency over execution speed**. p-TAREF was run over a set of genes for target identification, with different number of processors added through concurrency. As can be found, concurrency caused drastic reduction in processing time, which is highly beneficial in performing accurate transcriptome wide analysis.

As already discussed in the introduction section, unlike the animal system based miRNA target identification tools, plant miRNA target identification tools have witnessed limited growth till recently. Many of them revolved around complementarity search, using either heuristics like BLAST and FASTA or Smith-Waterman in their core. Most of them are web-server based and barring psRNAtarget, none of them provides the scope of concurrency to enable analysis of large amount of sequence data. Considering the revolutions made by next generation sequencing and systems biology approach, it becomes imperative to analyze transcriptome/genome level data at one go, with high accuracy as well as speed. BLAST and FASTA dependent methods do not require concurrency due to innate advantage of FASTA and BLAST to be much faster, though at the cost of accuracy and reliability. For that, some authors tried Smith-Waterman local alignment to detect complementarity, which becomes sharply slower with increment in the number and length of sequences to be searched and more so if all to all search has to be performed without the prior knowledge of the miRNA. We compared one such tool, Target-align, with p-TAREF, for time performance as among the very few tools available as the standalone version, Target-align is a recently published software with widespread use. We executed Target-align and p-TAREF on 205 plant genes from Arabidopsis and recorded the time taken to finish the job. Though p-TAREF run could be accelerated through concurrency and use of more processors, no such facility was available with Target-align, making us to run it with single processor and compare the performance for time taken. Table 1 summarizes the time performance and impact of introduction of concurrency. Figure 4 displays the plot showing reduction in execution period on introduction of concurrency when run over 790 mRNA sequences associated with plant secondary metabolite pathway. The processing speed of p-TAREF shot up with inclusion of more processors, making it a better choice to look for whole transcriptome wide scanning. Besides p-TAREF, only psRNAtarget provides the advantage of concurrency. However, comparison between them for execution speed was not possible as psRNAtarget is available only as a web-server and its concurrency has been implemented through cluster computers having several processors and large volumes of memory. Unlike psRNAtarget, p-TAREF is easily deployable on any level of machines and can run concurrently even on simple desktop machine.

**Table 1:** Impact of concurrency in p-TAREF.

# of processor/Mismatches	8	4	2	1
4	1 Hour 43 min	3 Hours 21 min	5 Hours 01 min	8 Hours 37 min
3	1 Hours 17 min	3 Hours 00 min	4 Hours 34 min	6 Hours 07 min
2	46 min	2 Hours 21 min	3 Hours 53 min	5 Hours 42 min
1	42 min	1 Hours 52 min	3 Hours 14 min	4 Hours 21 min
0	37 min	1 Hours 14 min	2 Hours 05 min	3 Hours 01 min
Target-Align	NA	NA	NA	92 Hours 26 min

p-TAREF was run over total 205 genes, with different number of processors, having Intel Xeon processors with 2.5 Ghz clock speed. The last row compares it with another tool Target-align, which is available as standalone, serially coded alignment based tool.

Contrary to the strictly similarity search based tools, as described above, p-TAREF, takes advantage from three different approaches: Similarity based, Thermodynamics based (RNAhybrid) and Machine Learning based. Barring psRNATarget, hardly any of the existing plant miRNA target identification tools consider the thermodynamic aspect as well as contribution of flanking region in deciding the target. In animal system it is now well proven that flanking regions reasonably participate in determination of the target site [23,30]. However unlike psRNAtarget, which measures the miRNA-RNA interaction and gross secondary structure of the mRNA using RNAup program [17] at its back-end, p-TAREF prefers to apply dinucleotide density variation profile around the target site as the multivariate feature set for discrimination through statistical machine learning approach, Support Vector Regression. Our previous work with animal system had already shown the effectiveness of detecting miRNA targets in animal system without getting trapped into the limitations of RNA structure prediction [21,23]. In overall, p-TAREF was compared with psRNATarget [29], Target-align [15] as well as TAPIR [13], through several tests. In the first two tests, p-TAREF was compared with Target-align and psRNAtarget, over the dataset created using experimentally validated targets reported in the supplementary material of Beauclair *et al *[5] as well as experimentally validated instances reported in ASRP database [25] along-with the mentioned negative dataset. Performance of p-TAREF was measured for three different kernel based models. It was found that even the lowest performing linear kernel based plant model performed better than psRNAtarget as well as Target-align for the given datasets. Table 2 presents the result of this performance comparison along with Additional File 1 (Table 2). This is to mention that while performing this assessment, the instances taken in training sets were entirely different from the one used in training, keeping it clear to test its performance on a dataset with never seen before instances.

**Table 2:** Performance comparison between psRNA-target, Target-align and p-TAREF.

	psRNA target		Target-align		P-TAREF (polynomial kernel)	
	**Beauclair *et al.***	**ASRP**	**Beauclair *et al.***	**ASRP**	**Beauclair *et al.***	**ASRP**

TP	81	119	64	103	104	262
FN	23	168	40	184	0	25
TN	119	119	119	119	119	119
FP	0	0	0	0	0	0
Sn	77.88	41.16	61.53	35.888	100	91.29
Sp	100	100	100	100	100	100
MCC	0.81	0.4146	0.678	0.4586	1	0.86
ACU%	89.68	58.620	82.06	50.800	100	93.84

*TP = True Positive, FP = False positive, TN = True Negative, FN = False negative, Sn = sensitivity, Sp = Specificity, MCC = Mathew Correlation Coefficient; Ac = Accuracy.

For experimentally validated targets, derived from two different sources, the tools were compared for performance. In the given table, p-TAREF was compared and found performing better than the compared tools for the given datasets. Performance related testing details are given in text, Additional File 1 as well as at server's performance page. The observed MCC value suggests about the robustness of model implemented in p-TAREF.

In the next test, performance of p-TAREF was compared with Target-align and TAPIR, for the reference dataset used by TAPIR [13] and Target-align [15]. Both the tools had used a common dataset for their performance benchmarking. On the same dataset, p-TAREF was run and found performing better than TAPIR and Target-align with sensitivity level of 100%, which is higher than the ones observed for TAPIR (93.14%) and Target-align (97.05%). Though it does not affect much even if same set is used for training and testing, in the present study it has been tried all over to keep the two sets entirely different and unseen. Here also, the number of instances which were common between the sets used for testing by TAPIR and Target-align were searched. Only seven out 105 instances were found common between TAPIR/Target-align reference test set and training set of p-TAREF. For this test, the benchmarking protocol applied by Bonnet *et al *for TAPIR [13] and Target-align [15], was followed exactly, where the predicted targets falling outside the experimentally validated regions were considered as the negative instances to calculate the false positive rates. Compared to these two tools, Target-align and TAPIR, p-TAREF had lower false positive rate. Table 3 presents the result for this benchmarking exercise.

**Table 3:** Performance comparison between TAPIR, Target-align and p-TAREF for Target-align/TAPIR Reference dataset for benchmarking.

	TAPIR		Target-align		p-TAREF
	**Fasta**	**RNAhybrid**	**Less stringent**	**More stringent**	**Polynomial kernel**
TP Rate %	91.83	93.14	97.05	93.14	100
FP Rate %	81.47	88.97	84	57.8	56.2

*TP = True Positive;FP = False Positive

The same benchmarking dataset and procedure was used for p-TAREF as had been used previously by the two tools. P-TAREF was found performing better.

Besides this, p-TAREF was also compared with psRNAtarget for experimentally validated dataset, which was used previously for performance benchmarking of psRNAtarget [31-34]. For all experimentally validated 46 instances of targets, p-TAREF identified 45 of them. Further experimentally validated target instances specific for Tomato, *Populus *and *Medicago *were collected and the performance of p-TAREF was measured on them. For available nine experimentally validated target instances in *Medicago truncatula *specific miRNAs, p-TAREF scored 100%. For all of the available eight experimentally validated targets from tomato, p-TAREF attained 100% accuracy. For *Populus trichocarpa*, 17 out of 21 experimentally validated and submitted instances were available, out of which 16 targets were identified successfully, notching an accuracy of 94.11%. For *Populus euphratica *21 targets out of 24 known instances, were successfully identified (Accuracy% = 87.5%). All the details regarding performance, benchmarking and associated tests are explained elaborately on the performance page of the server as well as in Additional File 1.

**Figure 5:**
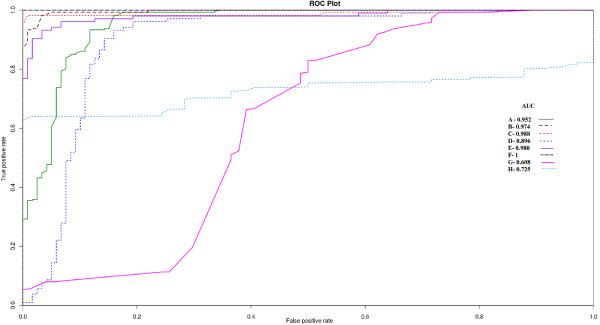
**The ROC plots for classifier models of p-TAREF with 10 fold cross validation**. As the plots show, the classifier was found robust in performance with high AUC values, where the highest one was observed for polynomial kernel model. For cases A-F, two major experimentally validated data sources, Beuclair et al(2010) and ASRP, were used to prepare the datasets. For cases F and H, tests were performed using the reference test set as well as protocol used by TAPIR and Target-align. The curves represent the following tests: A) Linear Kernel/ASRP B) Gaussian Kernel/ASRP C) Polynomial Kernel/ASRP D) Linear/Beuclair E) Gaussian/Beuclair F) Polynomial/Beuclair G)Target-align/(Tapir/Target align dataset) H) p-TAREF(Tapir/Target-Align dataset).

Using 10-fold cross validation, the performance robustness of all the three kernels and different tests was estimated. The Area Under Curve (AUC) values for most of the tests scored above 0.9, suggesting the robustness of the working theory, model built under the three kernels and their reliable performance. Figure 5 shows the Receiver Operating Characteristic curve (ROC) for the three models along-with their respective AUC values. The last two ROC plots are about performance of Target-align and p-TAREF, for the reference dataset used for benchmarking of Target-align by its authors, following the same protocol which they used to judge the false positive rate. The recorded AUC for p-TAREF was reasonably higher than the one observed for Target-align, suggesting more consistent performance by p-TAREF.

### Target identification in Rice transcriptome and emergence of miR156 as a prominent regulator

In the beginning of this part of the study, the validation and performance benchmarking process over the already known and experimentally validated miRNA target instances in rice transcriptome was done. Recently, Sunkar had group performed a degradome sequencing based study to report 153 miRNA targets [7]. For 29 rice specific miRNAs, the authors had reported 56 targets. For validation work the same experiment was used to validate targets identified by p-TAREF in the rice transcriptome. The sequence data was found available for 52 such target genes and p-TAREF identified most of the targets with overall accuracy of 97.33%. Encouraged by this, whole transcriptome analysis for miRNA targets in rice transcriptome sequences was carried out, excluding those sequences on which the above mentioned analysis had been performed already in order to avoid redundancy, looking for new targets and save time.

p-TAREF was run over 57,995 mRNA sequences from rice transcriptome dataset, with upto 4 mismatch level between experimental and predicted interaction patterns and polynomial kernel plant model. Initially, total 36,916 targets were identified for upto four differences from experimentally validated interaction patterns for target:miR interactions. Total 7,996 unique genes were found being targeted. Additional File 2 contains details of all identifications made at different mismatch levels. To validate the predicted targets with support of experimental data, the microarray expression data for all of the predicted target:miRNA pairs was checked. Out of 36,916 predicted miRNA targets, the expression data was available for 33,709 pairs to estimate the expression correlation between the target gene and corresponding miRNAs. After performing the expression correlation analysis, for 27,586 predicted target:miRNA pairs inverse expression correlation was observed, for different experimental conditions and tissue types, suggesting strong concordance with the predicted targets (81.8%). The expression correlation was compared with their respective SVR scoring and a reasonable agreement between the two was found with Pearson correlation coefficient of 0.7. The remaining 18.2% of identified targets had no agreement with expression correlation, which may also include condition like translational repression by miRNAs, which can't be interpreted well through inverse correlation estimation. While discussing this, it needs to be mentioned that expression data has certain limitations for inferences. It could be useful in case of transcript disruption, which is mostly prevalent in the plants. Though unlike animal system where translational repression has been reported more prevalent than transcript decay during miRNA targeting, recent studies have reported existence of translational repression in plants too, as discussed above. In such condition array expression data could not be much of help in inferring the process of targeting by microRNAs.

**Figure 6:**
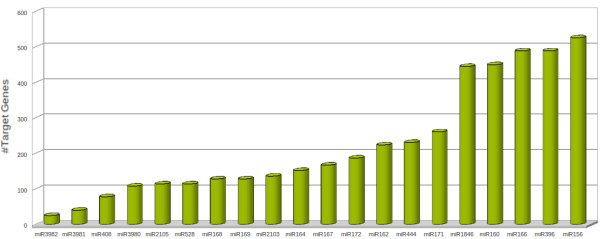
**miRNAs target distribution in Oryza sativa**. The major miRNA families found targeting the various genes in rice transcriptome.

For this run, targets were found mainly for ~20 different miRNA families, with over-representation by certain miRNA families (Figure 6). All such targeting miRNAs and associated target genes, along-with their available expression correlation, mismatch level for nearest interaction patterns and SVR scores are listed in Additional File 3. For the identified miRNA targets, showing strong inverse correlation with the associated miRNAs, the targets were grouped according to the miRNAs targeting them, and separate Gene Ontology analyses were performed over every such group. The related data is given in the Additional File 4.

From this study, miR156 family emerged as an important miRNA in *Oryza *system, with largest number of targets (526 unique genes), many of which also scored high for negative expression correlation with miR156. One of the possible reasoning for observing such high number of targets for miR156 could be attributed to existence of purine richness (GA/AG tract) in miR156 sequence, causing poly-pyrimidine regions to be counted as the targets due to complementarity. Though the algorithm design of p-TAREF has capacity minimize the noise, especially those arising through mere complementarity, yet a couple of analyses were performed to verify the above mentioned possibility. Maintaining the constant dinucleotide composition, a permuted miR156 sequence was generated. If the polypyrimidine tracts could influence the result significantly, one may expect to see the frequency of targets for such permuted miRNA with identical dinculeotide composition as almost of same level. However, when p-TAREF was run with most liberal parameters to find the permuted miR156 targets, only 105 genes were found being targeted and with consideration of only miR156 specific encoded interaction pattern comparisons, absolutely no hit was found for the permuted miR156. The same test was repeated with few more permuted miRNAs and almost similar pattern of lower number of random targets were observed, with absolutely no targets reported when miR156 specific encoded interaction patterns were considered. This suggests high reliability of identifications done by p-TAREF, where the user could also apply the different options parameters to limit the result of interest. Further, a search for polypyrimidine SSR regions in the rice transcriptome reported ~1000 genes with polypyrimidine tracts. When mapped for the target genes for miR156, only 56 genes were found common between these two sets of genes. For several of these 56 genes the target site was found non-overlapping with the polypyrimidine tracts. Therefore, these findings suggest a very limited possible role of repetitiveness/randomness in the observed abundance of miR156 targets. Also, this needs to be mentioned that the mentioned number of target genes for miR156 is the gross number of targets for miR156 obtained with the parameters described in the beginning of this section. Search could be refined further by applying various filters and options provided with p-TAREF, including SVR score cut-off, interaction pattern differences and expression correlation score, etc. Additional Files 2 and 3 hold all such details for rice, which could be used to refine the results further, based upon filters like SVR score/Correlation Score/Differences in encoded pattern/Selection of miRNA specific encoded patterns etc. Applying one of such cut-offs for inverse correlation for expression, we performed an analysis upon the top scoring targets for miR156, as demonstrated below.

**Figure 7:**
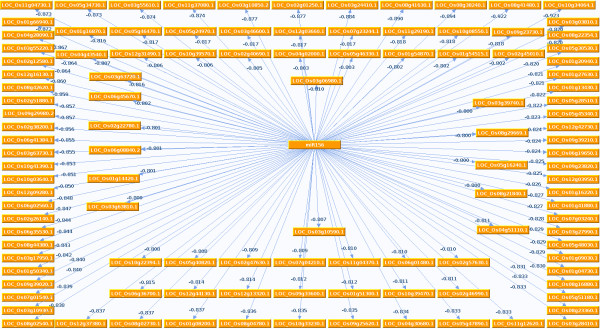
**Graphical representation of targets of miR156 in rice transcriptome**. All the targets shown here scored inverse expression correlation with miR156, having absolute value of 0.8 or higher.

**Figure 8:**
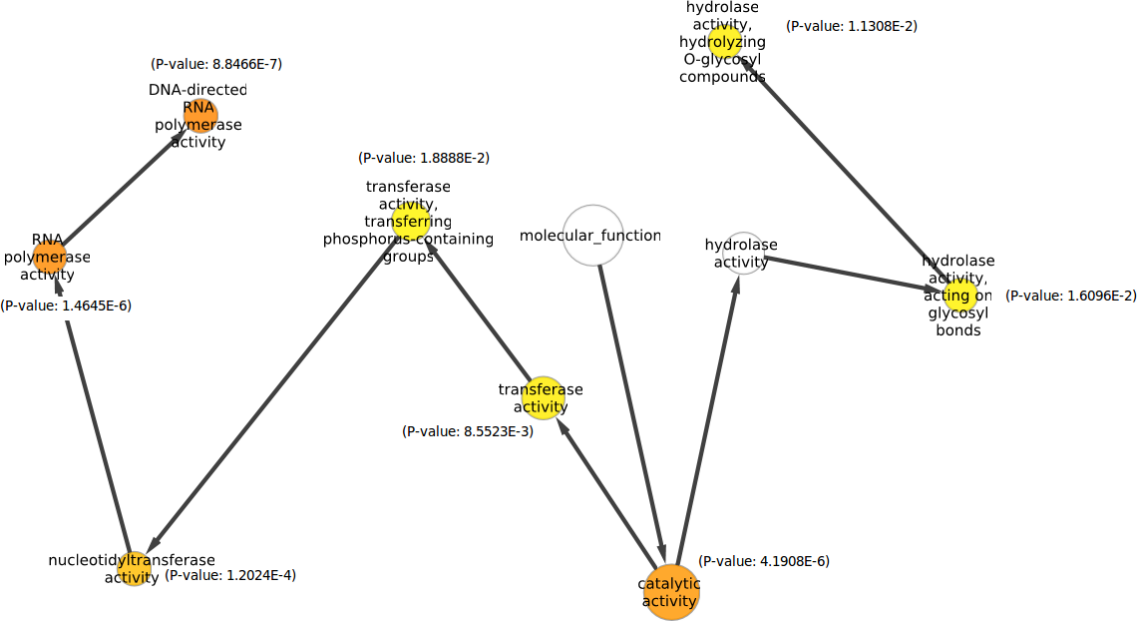
**Hypergeometric tests for enrichment of GO functional categories terms for molecular function**. The observation was made for enrichment of molecular functions found enriched and associated with targets of miR156. The colored nodes are functional categories whose genes were found significantly enriched in the pool of miR156 targets. Darker the color, more significant is the enrichment.

Figure 7 shows a group of identified targets for miR156 and their corresponding inverse correlation scores while Table 4 details about the possible functions and identification of top fifty of these targets along-with their SVR scores. All these targets scored inverse expression correlation values higher than 0.8 (i.e between -0.8 to -1). Gene Ontology studies over the identified miR156 target genes and associated statistical testing for enrichment provided some interesting informations. For miR156 certain biological terms were found enriched. Table 5 shows the top 20 significantly enriched GO terms found associated with miR156 targets in *Oryza sativa*. Figure 8 shows the result of statistical enrichment analysis using hyper-geometric tests, for molecular function categories of genes. For this part of the study, it can be seen that miR156 targeting was found significantly enriched for genes associated with process of transcription, nucleotide transfer process during transcription and catalytic activities. For further analysis, targets were searched for instances where a miR156 target was found being targeted by other miRNAs too. For such pairs some enriched miRNA pair instances were found. miR156-miR160 coexisted in 110 unique genes and transcripts, enriched for molecular functions like RNA polymerase activity (P-value: 1.7E-03). miR156-miR166 coexisted in 25 unique genes and 36 unique transcripts, where genes associated with molecular functions like Beta-Galactosidase activity (P-value: 1.6E-03), were found enriched. miR156-miR396 coexisted in 208 unique genes and 263 unique transcripts, showing enrichment for genes associated with molecular functions like Brassinosteriod-sulfotransferase activities (P-value: 4.9E-04), Fructokinase activities (P-value: 7.3E-04), Glucokinase activities (P-value: 7.3E-04) and UDP-gluco-4-aminobenzoate activities (p-value: 7.3E-04).

**Table 4:** Identified targets of miR156 in the rice transcriptome.

Transcript Id	Transcript annotation	Expression Correlation	SVR Score
LOC_Os08g41480.1	SAM domain containing protein, putative, expressed	-0.92	4.494426
LOC_Os10g34064.1	retrotransposon protein, putative, unclassified	-0.92	3.55511
LOC_Os08g38240.1	transposon protein, putative, CACTA, En/Spm sub-class, expressed	-0.89	0.305948
LOC_Os02g01250.1	LSM domain containing protein, expressed	-0.88	3.846277
LOC_Os03g10850.2	FAD-linked sulfhydryl oxidase ALR, putative, expressed	-0.88	3.9567
LOC_Os03g24410.1	conserved hypothetical protein	-0.88	4.39943
LOC_Os01g66940.1	kinase, pfkB family, putative, expressed	-0.87	3.55511
LOC_Os03g55610.1	dof zinc finger domain containing protein, putative, expressed	-0.87	3.73214
LOC_Os04g28090.1	MYB family transcription factor, putative, expressed	-0.87	3.01288
LOC_Os05g34730.1	ethylene-responsive transcription factor ERF020, putative, expressed	-0.87	3.55511
LOC_Os11g04730.1	DNA-directed RNA polymerases I, II, and III subunit RPABC1, putative, expressed	-0.87	2.02559
LOC_Os11g37080.1	h/ACA ribonucleoprotein complex subunit 1-like protein 1, putative, expressed	-0.87	3.73214
LOC_Os02g12580.1	OsPP2Ac-3 - Phosphatase 2A isoform 3 belonging to family 1, expressed	-0.86	0.591197
LOC_Os02g38200.1	dehydrogenase, putative, expressed	-0.86	3.01288
LOC_Os02g51880.1	amine oxidase, putative, expressed	-0.86	3.08791
LOC_Os03g55220.1	bHelix-loop-helix transcription factor, putative, expressed	-0.86	1.31754
LOC_Os03g63730.1	RNA recognition motif containing protein, putative, expressed	-0.86	0.742023
LOC_Os03g63730.1	RNA recognition motif containing protein, putative, expressed	-0.86	2.63747
LOC_Os06g41384.1	zinc finger C-x8-C-x5-C-x3-H type family protein, expressed	-0.86	3.08791
LOC_Os08g42620.1	zinc finger DHHC domain-containing protein, putative, expressed	-0.86	3.01288
LOC_Os09g29980.2	transposon protein, putative, CACTA, En/Spm sub-class, expressed	-0.86	3.01288
LOC_Os12g16130.1	transposon protein, putative, unclassified, expressed	-0.86	0.656255
LOC_Os02g26140.1	microtubule-binding protein TANGLED1, putative, expressed	-0.85	1.95761
LOC_Os06g02560.1	growth-regulating factor, putative, expressed	-0.85	2.543472
LOC_Os10g03640.1	hypothetical protein	-0.85	2.54657
LOC_Os10g41390.1	protein kinase domain containing protein, expressed	-0.85	0.411063
LOC_Os12g44130.1	expressed protein	-0.85	0.934949
LOC_Os10g41390.1	protein kinase domain containing protein, expressed	-0.85	1.64854
LOC_Os12g09280.1	RNA polymerase subunit, putative, expressed	-0.85	2.54657
LOC_Os01g08200.1	ubiquitin carboxyl-terminal hydrolase 14, putative, expressed	-0.84	2.5038
LOC_Os01g50340.1	transposon protein, putative, unclassified, expressed	-0.84	1.22697
LOC_Os03g10930.1	ribosomal protein L51, putative, expressed	-0.84	0.264129
LOC_Os03g17950.1	expressed protein	-0.84	1.17905
LOC_Os06g35530.1	CGMC_GSK.8 - CGMC includes CDA, MAPK, GSK3, and CLKC kinases, expressed	-0.84	0.996875
LOC_Os07g01540.1	Ser/Thr protein phosphatase family protein, putative, expressed	-0.84	2.517799
LOC_Os08g02540.1	adenylate kinase, putative, expressed	-0.84	2.51778
LOC_Os08g02730.1	plant protein of unknown function domain containing protein, expressed	-0.84	1.78162
LOC_Os08g04780.1	amine oxidase, putative, expressed	-0.84	0.111441
LOC_Os08g44380.1	L1P family of ribosomal proteins domain containing protein, expressed	-0.84	2.54161
LOC_Os09g25620.1	CPuORF8 - conserved peptide uORF-containing transcript, expressed	-0.84	2.08723
LOC_Os09g39020.1	N-rich protein, putative, expressed	-0.84	1.58374
LOC_Os10g33230.1	RNA recognition motif containing protein, putative, expressed	-0.84	1.95032
LOC_Os12g37380.1	RNA pseudouridine synthase, putative, expressed	-0.84	2.08857
LOC_Os01g04730.1	ribosomal protein L24, putative, expressed	-0.83	2.62583
LOC_Os01g09030.1	2-aminoethanethiol dioxygenase, putative, expressed	-0.83	2.63747
LOC_Os01g16220.1	Sad1/UNC-like C-terminal domain containing protein, putative, expressed	-0.83	1.68967
LOC_Os01g41880.1	hyaluronan/mRNA binding family domain containing protein, expressed	-0.83	2.50997
LOC_Os03g27990.1	STRUBBELIG-RECEPTOR FAMILY 7 precursor, putative, expressed	-0.83	2.03837
LOC_Os03g28410.1	ribosomal protein S2, putative	-0.83	1.94684
LOC_Os04g30680.1	conserved hypothetical protein	-0.83	1.94949

The listed targets scored at least 0.8 inverse expression correlation or higher.

**Table 5:** Top 20 most significant GO terms found associated with miR156 targets in the rice transcriptome.

Rank	Cellular component		Molecular Function		Biological function	
	**Go Terms**	**Significance** **(P-value)**	**Go Terms**	**Significance (P-value)**	**Go Terms**	**Significance (P-value)**

1	cell wall	2.20e-16	RNA binding	2.20e-16	cellular protein metabolic process	2.20e-16
2	cytosolic large ribosomal subunit	2.20e-16	copper ion binding	2.20e-16	DNA replication	2.20e-16
3	ribosome	2.20e-16	aspartic-type endopeptidase activity	2.20e-16	response to cadmium ion	2.20e-16
4	ribonucleoprotein complex	2.20e-16	aspartate kinase activity	2.20e-16	DNA integration	2.20e-16
5	mitochondrial inner membrane	2.20e-16	DNA-directed DNA polymerase activity	2.20e-16	translation	2.20e-16
6	Golgi apparatus	2.20e-16	zinc ion binding	2.20e-16	cellular amino acid biosynthetic process	2.20e-16
7	cytosolic small ribosomal subunit	2.20e-16	ubiquitin thiolesterase activity	2.20e-16	microtubule-based movement	2.20e-16
8	nuclear pore	2.20e-16	microtubule motor activity	2.20e-16	cellular amino acid metabolic process	2.20e-16
9	mitochondrion	2.20e-16	triose-phosphate isomerase activity	2.20e-16	intracellular protein transport	2.20e-16
10	cytoplasm	2.20e-16	branched-chain-amino-acid transaminase activity	2.20e-16	protein import into nucleus, docking	2.20e-16
11	cytosol	2.20e-16	structural constituent of ribosome	2.20e-16	shoot development	2.20e-16
12	cytoskeleton	2.20e-16	nucleic acid binding	2.20e-16	proteolysis	2.20e-16
13	cytosolic ribosome	2.20e-16	translation initiation factor activity	2.20e-16	branched chain family amino acid metabolic process	2.20e-16
14	nucleolus	2.20e-16	DNA binding	2.20e-16	ubiquitin-dependent protein catabolic process	2.956e-16
15	plasma membrane	5.21E-015	glyceraldehyde-3-phosphate dehydrogenase activity	2.20e-16	embryo development ending in seed dormancy	1.114e-15
16	proteasome complex	1.09e-14	NAD binding	4.27E-015	vesicle-mediated transport	3.006e-15
17	COPI vesicle coat	1.20e-13	glyceraldehyde-3-phosphate dehydrogenase (NAD+) (phosphorylating) activity	1.649e-14	rRNA processing	1.756e-14
18	outer membrane	1.39e-12	ligase activity	1.96e-14	translational elongation	2.675e-14
19	protein complex	1.43e-15	unfolded protein binding	2.25E-014	protein folding	4.47E-014
20	small ribosomal subunit	3.20e-12	hydrolase activity, acting on acid anhydrides, in phosphorus-containing anhydrides	2.67e-14	response to hormone stimulus	1.870e-13

The top scoring 20 terms associated with three GO categories are given with their associated significance scores (p-value).

Previously done studies have reported critical role of miR156 in plant growth and developmental stage transitions like flowering, fruit ripening and shoot development, controlling some important transcription factors like SPL [35,36]. Some recent studies now suggest that miR156 could be an eternal regulator of vegetative growth in plants and found critical in growth phase transitions [37]. The present study found strong affinity of miR156 towards targeting genes involved in the process of transcription, growth and development which goes in sync with findings made previously with mentioned studies for miR156.

## Conclusion

Like animals, in plant systems too, the role of flanking regions in determining miRNA targets appears as critical one. This was successfully tested for plants and implemented through the developed tool, p-TAREF. It works on statistical machine learning principle, deriving maximum margin classification decision boundary while considering multiple variables, which in the present work has been plant specific dinucleotide density profiles variations with respect to the possible target position. The confidence over that assigned class is derived through the scoring scheme of Support Vector Regression score. Besides this, implementation of concurrency provides p-TAREF an accelerated processing capability to harness multiple processors even on simple desktop machine as well as on big servers. p-TAREF web-server provides scope for expression based evidence for predicted targets, providing confidence on prediction, besides SVR scoring system to gather confidence on identification. The expression data and other associated publicly available information will be updated regularly with release of new data sources. The expression analysis and data in the present work were mainly based upon array experiments, which have some innate limitations. Though such array experiments may not produce the most accurate expression results, they have been used extensively for expression and abundance analysis at genome wide level and may provide a reasonable estimation of expression. For several of these experiments, RT-PCR based validation had been reported for the representative genes. More sensitive expression data from NGS and RT/q-PCR could be added in the upcoming versions of p-TAREF, depending upon the kind of experiments performed on these platforms and their public availability. For performance assessment, one of the most comprehensive performance measurements and comparisons with most recent and contemporary tools for miRNA target identification in plant system has been done, suggesting better performance by p-TAREF. Using p-TAREF, whole transcriptome level targets for rice transcriptome have been identified where miR156 was found as a critical miRNA in rice system. The reported targets were validated in two ways: using support from co-expression data as well as accurate identification of degradome analysis based targets. The identified targets could be an important resource to get clearer picture of regulation in rice. With all this, p-TAREF could be very helpful for the study of gene regulation and becomes more relevant considering the amount of data being produced by next generation sequencing projects, where p-TAREF could be applied over novel plant transcriptomes to discover miRNA targets.

## Availability and requirements

### Project name: p-TAREF

**Project home page: **http://scbb.ihbt.res.in/SCBB_dept/Software.phphttp://sourceforge.net/projects/ptaref/

**Operating system(s): **Platform independent web-server version as well as Linux specific standalone version.

**Programming language: **Python, PERL, Java, R

**Other requirements: **Web-server is recommended for single or small number of sequences. For batch mode analysis, prefer to use the standalone GUI version.

**License:** Free

**Any restrictions to use by non-academics:** None

## List of Abbreviations

ROC: Receiver Operating Characteristic Curve; miRNA: microRNA; Acc: Accuracy; Sp: Specificity; Sn: Sensitivity; MCC: Mathew Correlation Coefficient; AUC: Area Under Curve; NGS: Next Generation Sequencing; GUI: Graphical User Interface; JCL: Java Concurrent Library.

## Authors' contributions

AJ developed the codes, implemented the multi-core parallel versions, developed the web-server and GUI versions of the tool and conducted the entire study and performed the analysis part. RS planned and designed the entire study, developed the computational protocols, algorithms and theory, core basic codes, performed analysis and supervised the entire study. AJ and RS drafted the manuscript. All authors read and approved the final manuscript.

## Supplementary Material

Additional file 1 **Performance tests and benchmarking related details**. This additional file contains the details about the performance benchmarking and tests done for p-TAREF. In overall six different major tests were done for performance benchmarking. Click here for file

Additional file 2 **miRNA target predictions made on rice transcriptome**. This file contains result data on Rice transcriptome specific miRNA targets, with corresponding targeting miRNA, encoded interaction pattern differences and SVR score details. Click here for file

Additional file 3 **Expression correlation between miRNAs and targets**. The file contains details about the miRNA targets found in Rice transcriptome, along with expression correlation values between the target and targeting miRNA. Click here for file

Additional file 4 **miRNA groups and their corresponding functional category enrichments with p-values**. miRNA targets in Rice transcriptome were grouped according to the miRNA targeting them and their associated GO functional categories for Molecular function and Biological processes. Click here for file
