# Supplementary material for: Employing machine learning for reliable miRNA target identification in plants
Source: BMC Genomics. 2011 Dec 29;12:636. doi: 10.1186/1471-2164-12-636 (PMC3293931; doi:10.1186/1471-2164-12-636)
Supplement: Additional file 1 — Performance tests and benchmarking related details. This additional file contains the details about the performance benchmarking and tests done for p-TAREF. In overall six different major tests were done for performance benchmarking. [file 1471-2164-12-636-S1.PDF]

P-TAREF Supplementary file 1

(Related to performance and testing datasets)

p-TAREF is a tool/server to identify miRNA targets in plant transcriptome accurately and precisely with high speed. The training file for model was built by taking 104 experimentally validated sequences (source Beauclair*et al.*. *) as positive dataset and 119 sequences as negative dataset which also contains 32 experimentally validated negative sequences used by Heikham and Shankar (2010), using SVR classifier. We first tested our model on the training file as the test file for classifier. Out of 119 negative instances, our model predicted 100 sequences as negative (TN) and out of 104 positive targets, 98 were identified as positive instance (TP), with sensitivity of94.230 %and specificity of84.033%.The accuracy of p-TAREF for this test was~ 89%.To test our model again, we used experimentallly validated sequences from ASRP** database. We removed redundancy by removing common target sequences shared by Beauclair*et al.*(2010) dataset. After removing redundancy,we had 125 unique sequences on which we built our testing set. These 125 sequences are targets for 287 unique miRNA. Our model predicted 285 targets (TP) out of 287 experimentally validated targets and 100 as non targets (TN) out of 119 negative instances. The sensitivity and specificity for this test have been99.303 %and84.033 %respectively, accuracy in this case is~ 98%. We compared our tool's performance against two recent tools for plant miRNA target identification, and found it performing better than them in many aspects.

Abbreviations:

TP=True Positive

TN= True Negative

FP=False Positive

FN= False Negative

Sn=Sensitivity

Sp=Specificity

MCC=Matthew Correlation Coefficient

	psRNA target		Target-align		p-TAREF	
	Beauclair*et al.**	ASRP**	Beauclair*et al.**	ASRP**	Beauclair*et al.**	ASRP**
TP	245	119	203	103	288	285
FN	73	168	115	184	30	2
TN	119	119	119	119	100	100
FP	0	0	0	0	19	19
Sn	77.044	41.16	63.836	35.888	90.566	99.303
Sp	100	100	100	100	84.033	84.033
MCC	0.6910	0.4146	0.5697	0.4586	0.726	0.874
ACU%	83.895	58.620	73.684	50.800	88.787	97.222

Table 1 shows data for p-TAREF when executed from the beginning. The target sequences reported by Beauclair*et al.*were targeted by 318 miRNAs (104 unique miRNAs). The Kernel used by p-TAREF for this comparison data has been Linear one, which shows that even the less better performing kernel implementation of p-TAREF, achieved higher accuracy than the compared tools.

(Please visit the related table, Table 2, in the main text of the MS, as well as Table 2 of supplementary file 1 as given below, obtained after considering only unique miRNAs).

Impact of kernel selection on accuracy

	Polynomial Kernel		RBF/Gaussian kernel		Linear kernel	
	Beauclair*et al.*	ASRP	Beauclair*et al.*	ASRP	Beauclair*et al.*	ASRP
TP	104	262	98	285	98	285
FN	0	25	6	2	6	2
TN	119	119	113	113	100	100
FP	0	0	6	6	19	19
Sn	100	91.289	94.23	99.303	94.23	99.303
Sp	100	100	94.957	94.95	84.033	84.033
MCC	1	0.8685	0.8918	0.9522	0.781	0.874
ACU%	100	93.842	94.618	98.029	88.787	97.222

Table 2 shows the performance of p-TAREF on diffrent kernel. Use of Polynomial Kernel introduces high stringency, while moderate stringency is achieved by Gaussian Kernel and least stringency is obtained by using linear kernel (As suggested by TP and FP rates at various kernels). As the reported sequences by Beauclair*et al.*were targeted by 318 miRNAs (104 unique miRNAs), in order to make our training set non-redundant, we removed those target sites which are targeted by miRNAs of same family. The micro RNA mir-156 family target the same sequence at same position (as mir-156a,b,c .. have same mature miRNA sequences). Therefore, we removed such sequences from our training data while the experimentally validated sequences from ASRP database, were targeted by 287 unique miRNAs (after removing redundancy).

Area Under the Curve for p-TAREF is 0.9817

p-TAREF performance on Target-align/TAPIR Reference set

We downloaded the reference dataset, contianing 102 (3 targets were targeted by 2 diffrent miRNAs, mir165/mir166 that makes total 105 targets) experimentally validated plant miRNA targets, previously used by TAPIR and Target-align for their performance measurement. The target sequences were downloaded from TAIR and the sequences and their target site was subjected to p-TAREF. The found performance of p-TAREF on the given reference dataset for benchmarking was found to better than TAPIR (91.83%) and Target-align (93.14%), as can be found below:

	Linear kernel	RBF/Gaussian kernel	Polynomial kernel
TP	102	102	105
FN	3	3	0
TP Rate	97.142 %	97.142 %	100 %

The TP Rate and FP Rate comparision between Tapir, Target-align and p-TAREF.

	Tapir		Target-align		p-TAREF
	Fasta	RNAhybrid	Less stringent	More stringent	Polynomial kernel
TP Rate (%)	91.83	93.14	97.05	93.14	100
FP Rate (%)	81.47	88.97	84.0	57.8	56.2

We used the same dataset as was used by Target-align and Tapir to measure their respective performances. Our tool identifies total 689 targets (both positive and negative). After comparing the result with experimentally validated data, as practiced by Bonnet*et al*in their supplementery table, p-TAREF scored 100 % True positive rate (225 targets were matching with dataset, after removing duplicates, all 102 targets were successfully identified). To calculate the false positive rate we considered the targets predicted in target mRNAs, which fall outside the experimetnally validated target sites(similar protocol as used by TAPIR and Target-aling for their performance mesurement) as the false positives.

FP Rate = (339/604) X 100 = 56.2 %
(as applied by Bonnet*et al).*

References:

1. Bonnet E, He Y, Billiau K, Peer YV: TAPIR, a web server for the prediction of plant microRNA targets, including target mimics. Bioinformatics 2010, 12:1566-1568.

2. Xie F, Zhang B (2010) Target-align: a tool for plant microRNA target identification. Bioinformatics 23:3002-3003

Dataset Reference:

1. Endogenous siRNA and miRNA targets identified by sequencing of the Arabidopsis degradome, Addo-Quaye*et al.*, Curr. Biol., 2008, 18, 758-762.
2. microRNA-directed phasing during trans-acting siRNA biogenesis in plants, Allen*et al.*,Cell, 2005, 121, 207-221.
3. Comprehensive prediction of novel microRNA targets in Arabidopsis thaliana, Alves*et al.*, Nucleic Acids Res , 2009, 37, 4010-4021.
4. Global identification of microRNA-target RNA pairs by parallel analysis of RNA ends, German*et al.*, Nat Biotechnol, 26, 941-946.

Performance of p-TAREF on psRNAtarget dataset

The data set used by Daiat al.is used to measure accuracy of our tool. Out of given 46 validated targets, 45 were identified by p-TAREF. The accession Ids of the targets and the corrosponding miRNA is given below:

Experimentally validated Targets id used by Dai*et al.*	miRNA

AT1G27360	ath-miR156g
AT1G27370	ath-miR156g
AT1G53160	ath-miR156g
AT1G69170	ath-miR156g
AT2G42200	ath-miR156g
AT3G57920	ath-miR156g
AT5G43270	ath-miR156g
AT5G50570	ath-miR156g
AT5G50670	ath-miR156g
AT1G27370	ath-miR156a
AT1G53160	ath-miR156a
AT1G69170	ath-miR156a
AT2G33810	ath-miR156a
AT5G43270	ath-miR156a
AT1G66690	ath-miR163
AT1G66700	ath-miR163
AT1G66720	ath-miR163
AT3G44860	ath-miR163
AT1G56010	ath-miR164a
AT3G15170	ath-miR164a
AT5G07680	ath-miR164a
AT5G53950	ath-miR164a
AT5G61430	ath-miR164a
AT1G30330	ath-miR167c
AT5G37020	ath-miR167c
AT1G48410	ath-miR168a
AT5G12840	ath-miR169b
AT2G28550	ath-miR172a
AT3G54990	ath-miR172a
AT4G36920	ath-miR172a
AT5G60120	ath-miR172a
AT5G67180	ath-miR172a
AT1G50055	ath-miR173
AT2G27400	ath-miR173
AT2G39675	ath-miR173
AT2G39681	ath-miR173
AT1G27360	ath-miR157d
AT1G27370	ath-miR157d
AT1G53160	ath-miR157d
AT1G69170	ath-miR157d
AT2G42200	ath-miR157d

AT3G15270	ath-miR157d
AT3G57920	ath-miR157d
AT5G43270	ath-miR157d
AT5G50570	ath-miR157d
AT5G50670	ath-miR157d

Reference:
1. Dai X and Zhao PX (2011) psRNATarget: a plant small RNA target analysis server.

Nucleic Acids Res 2011, 1-5.

Performance of p-TAREF on Rice miRNA target prediction

We downloaded experimentally validated rice miRNA target sequences reported by Li*et al.*.(2010) from RiceGE: Rice Functional Genomic Express Database, and run p-TAREF on these sequences to find out miRNA targets in rice. Identification accuracy of p-TAREF, noted over Rice sequences was tremendous, as can be found below. The list of rice targets gene ID is given below:

Rice gene Ids	miRNA
Os01g69830	osa-miR156
Os06g45310	osa-miR156
Os03g02970	osa-miR162
Os12g41680	osa-miR164
Os05g39650	osa-miR164
Os12g41860	osa-miR166
Os11g30370	osa-miR156
Os06g47150	osa-miR160
Os06g46270	osa-miR164
Os08g10080.1	osa-miR164
Os03g50040	osa-miR164
Os03g01890	osa-miR166
Os10g33960	osa-miR166
Os12g41950	osa-miR167
Os02g53620	osa-miR169
Os03g29760	osa-miR169
Os03g48970	osa-miR169
Os07g41720	osa-miR169
Os07g33790	osa-miR167
Os02g06910	osa-miR167
Os06g46410	osa-miR167
Os03g07880	osa-miR169
Os03g44540	osa-miR169
Os07g06470	osa-miR169
Os02g44360	osa-miR171
Os01g25484	osa-miR2105

Os04g55560	osa-miR172
Os01g04550	osa-miR172
Os05g05800	osa-miR393
Os02g47280	osa-miR396
Os12g42400	osa-miR169
Os04g46860	osa-miR171
Os05g03040	osa-miR172
Os04g32460	osa-miR393
Os01g69940	osa-miR394
Os03g47140	osa-miR396
Os06g02560	osa-miR396
Os07g46990.1	osa-miR398
Os08g37670	osa-miR408
Os08g07540	osa-miR414
Os01g55880	osa-miR414
Os12g29980	osa-miR396
Os06g29430	osa-miR396
Os06g11490	osa-miR408
Os07g46990.1	osa-miR398
Os08g37670	osa-miR408
Os08g07540	osa-miR414
Os01g55880	osa-miR414
Os06g11310	osa-miR528
Os12g29980	osa-miR396
Os06g29430	osa-miR396
Os06g11490	osa-miR408

Accuracy for p-TAREF for prediction for miRNA targets was 88 % (Gaussian kernel), when only Arabidopsis miRNAs were considered. When we incorporated rice specific miRNAs in our miRNA library along with Arabidopsis miRNAs, accuracy reached 94.66%. When we predicted targets directly through the SVR, without incorporating the RNAhybrid and filtering steps, the accuracy achieved was 97.33%, showcasing extremely good performance of p-TAREF

Reference

1. Li Y, Zheng Y, Addo-Quaye C, Zhang L, Saini A, Jagadeeswaran G, Axtell MJ, Zhang W, Sunkar R: Transcriptome-wide identification of microRNA targets in rice. Plant J 2010, 62:742-759.

2. Rice GE : “http://signal.salk.edu/cgi-bin/RiceGE”.

Performance of p-TAREF on Medicago truncatulatarget prediction

We downloaded experimentally validated*Medicago truncatula* target sequences reported by Jagadeeswaran *et al.*. from Medicago genome project, and run p-TAREF on these sequences to find out miRNA targets. In their paper they found 19 targets for miRNAs. The list of *Medicago*

truncatula targets gene ID is given below:

Transcript Ids	miRNA
ES612384	mtr-mir160
AC150443_32.2	mtr-mir162
AC14478_44.4	mtr-mir167
AW773594	mtr-mir168
A238429	mtr-mir172
AC146721_16.4	mtr-mir395 (removed)
AC135467_30.2	mtr-mir397 (removed)
AC161863_13.2	mtr-mir408 (removed)
BQ148941	mtr-mir160
AC203553_1.1	mtr-mir164
CU32639_14.1	mtr-mir167
AC121238_43.2	mtr-mir170
AC133780_22.2	mtr-mir393
AC203224_21.2	mtr-mir397
BG583436	mtr-mir408
AC144658	mtr-mir399
AC202360_18.1	mtr-mir2118
AC143338_38.2	mtr-mir2118
AC203224_171	mtr-mir2118

Out of above mentioned miRNAs, three miRNAs were removed from miRBase (Release 17). We found sequences of 9 transcripts (out of 16 as 3 microRNA were removed from miRBase) and executed p-TAREF on these sequences the accuracy attained for this data is 100%

Reference

1. Jagadeeswaran G, Zheng Y, Li Y, Shukla LI, Matts J, Hoyt P, Macmil SL, Wiley GB, Roe BA, Zhang W, Sunkar R: Cloning and characterization of small RNAs from Medicago truncatula reveals four novel legume-specific microRNA families. New Phytol 2009, 184:85-98.

2. Medicago Genome Sequence Consortium "http://www.medicago.org/genome/downloads/Mt2/".

Performance of p-TAREF onSolanum lycopersicumtarget prediction

12 experimentally validated*Solanum lycopersicum*miRNA-target sequences was reported by Moxon*et al.*for 11 miRNA but some these of miRNAs are discarded in mirbase (version 17), therefore we were left with 8 miRNA-target complex. The mRNA sequences were given in their supplementry file, these targets were subjected to SVM classification all the three kernels predicts all 8 targets successfully (100 % Accuracy). The list of targets gene ID and corrosponding miRNA is given below:

Target Id	miRNA
SGN-U324312	sly-miR156

SGN-U317177	sly-miR156
SGN-U319736	sly-miR156
SGN-U324618	sly-miR160a
SGN-U321033	sly-miR166
SGN-U327976	sly-miR167
SGN-U314858	sly-miR171
SGN-U333058	sly-miR172

Reference :

1. Moxon S, Jing R, Szittya G, Schwach F, Rusholme Pilcher RL, Moulton V, Dalmay T: Deep sequencing of tomato short RNAs identifies microRNAs targeting genes involved in fruit ripening.Genome Res. 2008, 18:1602-1609.

Performance of p-TAREF on Populus euphratica target prediction

We downloaded populus miRNA target sequences validated by *Li et al.* For *Populus euphratica* and *Populus trichocarpa* from Populus trichocarpa v1.1. These targets were subjected to SVM classification. For *Populus trichocarpa* we found sequences of 17 targets out of 21, and 16 targets were successfully identified by both Gaussian and Polynomial kernel, with accuracy of 94.11 %. For*Populus euphratica* 18 and 21 targets out of 24 were successfully identified by Polynomial (Acu = 75.0 %) and Gaussian (Acu = 87.5 %) kernel respectively.

The list of verified miRNA targets of *Populus trichocarpa* is given below:

Target Id	*Populus trichocarpa*miRNA
jgi|Poptr1_1|733659| estExt_Genewise1_v1.C_LG_XV2187	ptc-miR156k
jgi|Poptr1_1|769914|fgenesh4_pg.C_LG_X001404	ptc-miR156k
jgi|Poptr1_1|733659| estExt_Genewise1_v1.C_LG_XV2187	ptc-miR156a
jgi|Poptr1_1|769914|fgenesh4_pg.C_LG_X001404	ptc-miR156a
jgi|Poptr1_1|178285|gw1.I.6885.1	ptc-miR159f
jgi|Poptr1_1|558202|eugene3.00091462	ptc-miR159f
jgi|Poptr1_1|208135|gw1.V.3536.1	ptc-miR164f
jgi|Poptr1_1|218417|gw1.VII.2722.1	ptc-miR164f
jgi|Poptr1_1|570963|eugene3.00130327	ptc-miR475a
jgi|Poptr1_1|570289|eugene3.00120942	ptc-miR156k
jgi|Poptr1_1|570289|eugene3.00120942	ptc-miR156a
jgi|Poptr1_1|208135|gw1.V.3536.1	ptc-miR164d
jgi|Poptr1_1|218417|gw1.VII.2722.1	ptc-miR164d
jgi|Poptr1_1|588910|eugene3.02710006	ptc-miR482-1
jgi|Poptr1_1|675520|grail3.0250000201	ptc-miR482-1
jgi|Poptr1_1|276236|gw1.182.27.1	ptc-miR1444a
jgi|Poptr1_1|769914|fgenesh4_pg.C_LG_X001404	ptc-miR156i
jgi|Poptr1_1|292259|gw1.6326.1.1	ptc-miR166b

jgi|Poptr1_1|778882| fgenesh4_pg.C_LG_XVIII000250	ptc-miR166b
jgi|Poptr1_1|832118| estExt_fgenesh4_pm.C_LG_VI0713	ptc-miR166b
jgi|Poptr1_1|797557|fgenesh4_pm.C_LG_I000560	ptc-miR166n

The list of newly discovered miRNA of*Populus euphratica*and their targets is given below:

Target Id	*Populus euphratica*miRNA
jgi|Poptr1_1|548199|eugene3.00010640	peu-miR30a
jgi|Poptr1_1|579296|eugene3.105640001	peu-miR30a
jgi|Poptr1_1|788190|fgen esh4_pg.C_scaffold_263000013	peu-miR30a
jgi|Poptr1_1|548199|eugene3.00010640	peu-miR30b
jgi|Poptr1_1|430410|gw1.VIII.1137.1	peu-miR67x
jgi|Poptr1_1|554868|eugene3.00031501	peu-miR67*
jgi|Poptr1_1|548199|eugene3.00010640	peu-miR71*
jgi|Poptr1_1|579296|eugene3.105640001	peu-miR71*
jgi|Poptr1_1|640215|grail3.0008024501	peu-miR71*
jgi|Poptr1_1|788190| fgenesh4_pg.C_scaffold_263000013	peu-miR71*
jgi|Poptr1_1|55274	peu-miR77
jgi|Poptr1_1|732312| estExt_Genewise1_v1.C_LG_XIV3469	peu-miR77
jgi|Poptr1_1|806761|fgenesh4_pm.C_LG_XIII000061	peu-miR84*
jgi|Poptr1_1|656445|grail3.0010018301	peu-miR93aa
jgi|Poptr1_1|714215| estExt_Genewise1_v1.C_LG_IV3721	peu-miR93aa
jgi|Poptr1_1|656445|grail3.0010018301	peu-miR93b
jgi|Poptr1_1|570289|eugene3.00120942	peu-miR131
jgi|Poptr1_1|733659| estExt_Genewise1_v1.C_LG_XV2187	peu-miR131
jgi|Poptr1_1|755123|fgenesh4_pg.C_LG_II001303	peu-miR131
jgi|Poptr1_1|769914|fgenesh4_pg.C_LG_X001404	peu-miR131
jgi|Poptr1_1|733659| estExt_Genewise1_v1.C_LG_XV2187	peu-miR58
jgi|Poptr1_1|769914|fgenesh4_pg.C_LG_X001404	peu-miR58
jgi|Poptr1_1|793900| fgenesh4_pg.C_scaffold_9189000001	peu-miR131
jgi|Poptr1_1|829056| estExt_fgenesh4_pg.C_170200031_1	peu-miR106*
jgi|Poptr1_1|837031|estExt_fgenesh4_pm.C_1230037	peu-miR106*
jgi|Poptr1_1|434998|gw1.57.264.1jgi	peu-miR115a
jgi|Poptr1_1|817423|	peu-miR123a

estExt_fgenesh4_pg.C_LG_III1182	
jgi|Poptr1_1|180750|gw1.I.9350.1	peu-miR101a

Reference

1. Li B, Qin Y, Duan H, Yin W, Xia X: Genome-wide characterization of new and drought stress responsive microRNAs in Populus euphratica. J Exp Bot 2011, 10.1093/jxb/err051.
2. Populus trichocarpa v1.1 "http://genome.jgi-psf.org/Poptr1_1/Poptr1_1.home.html".

p-TAREF performance measure on introduction of concurrency

Data for p-TAREF execution time when executed on different processors. Configuration of system on which p-TAREF speed performance was measured "2 X Intel Xeon (Quad core)" processors, RAM 24GB, file size = 381.7 kb, Number of genes is equal to 205

# of processor/ (mismatches)	8	4	2	1
p-TAREF (4)	1 Hour 43 min	3 Hours 21 min	5 Hours 01 min	8 Hours 37 min
p-TAREF (3)	1 Hours 17 min	3 Hours 00 min	4 Hours 34 min	6 Hours 07 min
p-TAREF (2)	46 min	2 Hours 21 min	3 Hours 53 min	5 Hours 42 min
p-TAREF (1)	42 min	1 Hours 52 min	3 Hours 14 min	4 Hours 21 min
p-TAREF (0)	37 min	1 Hours 14 min	2 Hours 05 min	3 Hours 01 min
Target-align	N/A	N/A	N/A	92 Hours 26 min

* Beauclair L, Yu A, Bouché N: microRNA-directed cleavage and translational repression of the copper chaperone for superoxide dismutase mRNA in Arabidopsis. Plant J 2010, 62:454-462.

** Tyler W.H. Backman, Christopher M. Sullivan, Jason S. Cumbie, Zachary A. Miller, Elisabeth J. Chapman, Noah Fahlgren, Scott A. Givan, James C. Carrington, and Kristin D. Kasschau: Update of ASRP: the Arabidopsis Small RNA Project database. Nucleic Acids Res 2008, 36:D982–D985.

Experimentally validated positive target gene ID for *Arabidopsis thaliana* collected from TAIR Version 10.

>AT2G33810
>AT1G27370
>AU1G53160
>AT5G43270

>AT3G15270
>AT2G42200
>AT1G69170
>AT5G55930
>AT5G18100
>AT2G34010
>AT2G32460
>AT4G37770
>AT3G60460
>AT1G77850
>AT2G28350
>AT4G30080
>AT1G06580
>AT1G63150
>AT1G63230
>AT4G30440
>AT1G66690
>AT1G66700
>AT1G66720
>AT3G44860
>AT1G56010

>AT3G15170
>AT5G07680
>AT5G53950
>AT5G61430
>AT5G39610
>AT1G30490
>AT1G52150
>AT2G34710
>AT4G32880
>AT5G60690
>AT5G37020
>AT1G48410
>AT3G58030
>AT5G12840
>AT3G60630
>AT4G00150
>AT2G28550
>AT4G36920
>AT5G60120
>AT5G67180
>AT2G39250
>AT3G54990

>AT2G27400
>AT2G39675
>AT2G39681
>AT1G50055
>AT1G30210
>AT1G53230
>AT2G31070
>AT3G15030
>AT4G18390
>AT3G17185
>AT1G12820
>AT3G23690
>AT3G26810
>AT3G62980
>AT4G03190
>AT1G27340
>AT5G10180
>AT5G43780
>AT2G36400
>AT4G24150
>AT5G53660

>AT2G22840
>AT4G37740
>AT2G45480
>AT1G10120
>AT2G38080
>AT5G60020
>AT2G29130
>AT3G60250
>AT3G15640
>AT1G08830
>AT1G12520
>AT2G33770
>AT1G31280
>AT2G30210
>AT5G05390
>AT5G07130
>AT2G02850
>AT1G72230
>AT2G02850
>AT5G60760
>AT1G53290
>AT5G41610

>AT3G57230
>AT3G14560
>AT1G02860
>AT1G66370
>AT5G38550
>AT5G51270
>AT5G49850
>AT5G41610
>AT3G09220
>AT2G47460
>AT5G49330
>AT1G06180
>AT1G66230
>AT3G49510

ASRP Database positive Id

AT1G11810
AT1G12210
AT1G12220

AT1G12280
AT1G12290
AT1G15890
AT1G17590
AT1G22960
AT1G24793
AT1G24880
AT1G25054
AT1G25141
AT1G25210
AT1G27360
AT1G30060
AT1G30330
AT1G32140
AT1G52050
AT1G52060
AT1G52070
AT1G52130
AT1G54160
AT1G57570
AT1G60110
AT1G62590

AT1G62630
AT1G62670
AT1G62720
AT1G62860
AT1G62910
AT1G62930
AT1G63070
AT1G63080
AT1G63130
AT1G63330
AT1G63360
AT1G63400
AT1G64100
AT1G64580
AT1G67450
AT1G72830
AT1G76810
AT2G04920
AT2G18780
AT2G24510
AT2G25980

AT2G26950
AT2G27520
AT2G35160
AT2G39250
AT2G41720
AT2G45160
AT3G03580
AT3G05690
AT3G12977
AT3G13820
AT3G13830
AT3G16710
AT3G16820
AT3G16880
AT3G17265
AT3G17280
AT3G17490
AT3G17540
AT3G17570
AT3G19880
AT3G20710
AT3G20910

AT3G21170
AT3G22350
AT3G22470
AT3G22700
AT3G22710
AT3G22720
AT3G22890
AT3G24580
AT3G44870
AT3G45090
AT3G49520
AT3G52910
AT3G54990
AT3G57920
AT4G10780
AT4G14680
AT4G19440
AT4G26800
AT4G26930
AT4G27190
AT4G33290

AT4G34060	
AT5G05400	
AT5G16640	
AT5G23480	
AT5G28520	
AT5G36200	
AT5G36730	
AT5G36820	
AT5G39710	
AT5G42460	
AT5G43730	
AT5G46680	
AT5G47260	
AT5G49870	
AT5G50570	
AT5G50670	
AT5G53890	
AT5G55020	
AT5G62310	
AT5G63020	
AT5G65560	

Sequences used as negative instances in model creation

>AT1G01040|AT1G01040.1

ATGGTAATGGAGGATGAGCCTAGAGAAGCCACAATAAAGCCTTCTTATTGGCTAGATGCT
TGCGAGGACATCTCTTGTGATCTTATCGATGATCTCGTGTCTGAATTTGATCCTTCCTCTG
TTGCTGTCAATGAATCCACTGATGAAAACGGCGTCATCAATGATTTTTTCGGTGGGATTG
ATCACATTTTAGATAGTATCAAGAACGGTGGAGGCTTACCAAACAATGGCGTTTCTGATA
CCAATTCTCAAATCAACGAGGTTACTGTAACTCCTCAGGTTATTGCTAAGGAGACAGTG
AAGGAGAATGGGTTGCAAAAGAATGGCGGTAAGAGAGACGAATTCTCGAAAGAGGAA
GGAGACAAGGATAGGAAGAGAGCTAGGGTTTGTAGTTATCAGAGTGAAAGGAGTAACC
TTTCAGGTAGAGGGCATGTTAATAATTCTAGGGAGGGAGATAGGTTTATGAATAGGAAAC
GTACTCGTAATTGGGACGAGGCGGGTAACAATAAGAAGAAAAGGGAATGTAACAATTAC
AGAAGAGATGGTAGAGATAGAGAAGTTAGGGGTTATTGGGAGAGGGATAAAGTTGGTTC
CAATGAGTTGGTTTATAGGTCAGGGACTTGGGAAGCTGATCATGAAAGAGATGTTAAGA
AAGTGAGTGGTGGAAACCGCGAATGCGATGTCAAGGCAGAGGAGAACAAGAGTAAGC
CTGAAGAACGTAAAGAGAAGGTTGTGGAAGAGCAAGCAAGGCGATACCAGTTGGATGT
TCTTGAACAAGCTAAAGCGAAAAACACGATTGCTTTCCTTGAGACCGGTGCTGGAAAG
ACACTTATCGCGATTCTTCTTATTAAAAGTGTTCATAAGGATCTGATGAGCCAGAACAGA
AAAATGCTCTCGGTGTTCTTGGTTCCCAAAGTGCCTTTGGTTTATCAGCAAGCAGAAGT
GATCCGTAATCAAACTTGTTTTCAAGTTGGACATTATTGTGGTGAGATGGGACAGGACTT
TTGGGATTCTCGAAGGTGGCAACGAGAGTTTGAGTCTAAGCAGGTTCTAGTTATGACAG
CACAAATTCTGTTGAATATACTGAGACACAGTATCATTAGAATGGAAACAATTGATCTTC
TTATTCTCGACGAGTGTCACCACGCTGTCAAGAAACATCCATACTCTTTAGTGATGTCAG
AGTTTTACCATACAACTCCTAAAGATAAAAGACCTGCCATCTTTGGAATGACTGCTTCGC
CTGTTAATTTAAAGGGTGTTTCAAGCCAAGTAGATTGTGCGATAAAGATACGTAACCTCG
AGACCAAGTTGGATTCTACGGTTTGTACTATAAAAGATCGAAAAGAATTAGAGAAACAT
GTGCCTATGCCTTCAGAGATAGTCGTCGAGTATGACAAAGCTGCTACTATGTGGTCTCTT
CATGAGACAATAAAGCAAATGATTGCAGCTGTTGAAGAAGCGGCACAAGCAAGTTCAA
GGAAAAGCAAGTGGCAATTTATGGGGGCTAGGGATGCTGGAGCAAAGGATGAATTGAG
ACAGGTTTATGGCGTCTCTGAAAGAACGGAGAGCGATGGTGCTGCCAATTTGATTCATA
AACTTAGAGCTATCAATTATACTCTTGCTGAATTGGGTCAATGGTGTGCTTACAAGGTGG
GACAATCATTCTTGTCTGCTTTGCAAAGTGATGAGAGGGTGAATTTCCAAGTCGACGTG
AAGTTTCAAGAATCATACCTCAGTGAGGTGGTGTCACTCTTGCAATGTGAGCTTCTGGA
AGGCGCTGCTGCTGAAAAAGTCGCGGCGGAAGTTGGCAAACCAGAAAATGGTAATGCA
CATGACGAGATGGAGGAGGGAGAGCTCCCTGATGATCCTGTGGTCTCGGGAGGGGAGC
ACGTTGATGAAGTAATAGGCGCCGCAGTGGCTGATGGGAAAGTTACTCCAAAAGTACAA
TCATTGATCAAACTACTCCTCAAATATCAGCACACAGCTGATTTTCGAGCTATTGTTTTCG
TTGAGAGGGTGGTTGCTGCTTTGGTTCTTCCTAAGGTTTTTGCGGAGCTGCCTTCGCTTA
GTTTTATACGGTGTGCCAGCATGATTGGACACAATAACAGCCAGGAGATGAAATCATCTC
AAATGCAGGATACAATTTCCAAATTCCGAGATGGGCATGTGACACTGTTAGTTGCCACA
AGCGTTGCTGAGGAAGGACTTGATATTAGGCAATGTAACGTTGTTATGCGTTTCGACCTT
GCAAAGACGGTGCTGGCATACATTCAGTCTCGTGGCCGGGCAAGAAAGCCTGGATCAG
ACTACATACTCATGGTTGAGAGAGGAAATGTATCTCACGCAGCGTTCCTAAGGAATGCTA
GGAACAGTGAGGAGACACTTCGAAAAGAAGCAATAGAAAGGACTGATCTTAGTCATCT
CAAAGATACATCGAGATTAATCTCAATTGATGCTGTGCCTGGTACAGTTTATAAGGTGGA
GGCAACTGGTGCCATGGTTAGCTTGAATTCCGCGGTTGGTCTTGTACATTTCTACTGCTC
TCAGCTTCCTGGTGACAGGTATGCAATCCTTCGTCCTGAGTTTAGCATGGAGAAGCATGA
AAAGCCTGGGGGCCACACGGAATATTCATGTAGGCTTCAGCTTCCTTGCAATGCACCGT
TTGAAATACTTGAGGGTCCTGTTTGCAGTTCAATGCGTCTTGCACAACAGGCTGTATGTT
TAGCTGCTTGCAAGAAACTGCATGAGATGGGTGCATTTACCGATATGCTATTACCGGACA
AAGGAAGTGGTCAAGACGCTGAGAAGGCTGACCAAGATGATGAAGGTGAGCCTGTTCC
TGGAACTGCTAGACATAGAGAGTTCTATCCTGAAGGTGTGGCGGATGTACTTAAGGGAG

AATGGGTTTCATCTGGAAAGGAAGTTTGTGAGAGCTCAAAGCTATTCCATTTATACATGT
ATAATGTCAGATGTGTAGATTTTGGCTCTTCAAAAGATCCATTCCTAAGCGAAGTTTCAG
AGTTCGCGATTCTTTTTGGCAATGAGCTGGATGCAGAGGTATTATCGATGTCTATGGATCT
TTATGTTGCTCGGGCCATGATCACTAAAGCATCTCTTGCTTTCAAGGGATCACTTGATATT
ACAGAAAACCAGCTATCATCTCTAAAAAAGTTTCATGTGAGGTTAATGAGTATCGTGTTG
GATGTTGATGTTGAACCCTCCACGACACCATGGGATCCTGCAAAGGCCTACCTGTTTGTC
CCTGTTACTGACAATACGTCTATGGAACCCATAAAAGGGATCAACTGGGAATTGGTTGA
AAAGATTACGAAAACCACAGCGTGGGACAACCCTCTTCAGAGAGCTCGTCCCGATGTAT
ATCTCGGGACTAATGAGAGAACTCTTGGTGGGGACAGAAGGGAATATGGGTTTGGTAAA
CTTCGTCACAACATTGTATTTGGGCAGAAATCTCACCCAACTTATGGTATTAGAGGAGCT
GTTGCATCCTTCGATGTTGTGAGAGCTTCTGGATTGTTACCTGTGAGAGATGCTTTTGAG
AAGGAAGTAGAAGAGGATTTATCAAAAGGAAAATTGATGATGGCTGATGGGTGCATGGT
TGCAGAAGATCTTATTGGGAAAATAGTGACAGCCGCACATTCCGGGAAGCGGTTTTACG
TAGATTCAATTTGTTATGACATGAGTGCAGAAACATCTTTCCCTAGGAAAGAGGGATATC
TTGGTCCCCTAGAGTACAACACGTACGCTGACTATTACAAGCAAAAGTATGGAGTTGATT
TGAACTGTAAGCAACAACCTTTGATTAAAGGACGTGGTGTTTCGTATTGCAAGAACCTT
CTTTCTCCTCGGTTTGAACAGTCAGGTGAATCTGAGACAGTCCTTGATAAGACATATTAC
GTGTTTCTTCCACCTGAACTATGCGTTGTGCATCCGCTTTCGGGTTCACTTATCCGAGGT
GCTCAGAGGTTACCCTCTATAATGAGAAGAGTTGAGAGCATGTTACTCGCTGTTCAACTC
AAAAATTTGATTAGTTATCCTATTCCCACATCAAAGATTCTTGAAGCCTTGACTGCCGCCT
CGTGCCAGGAAACGTTCTGCTACGAGAGAGCTGAGCTTTTAGGAGATGCGTATCTAAAA
TGGGTTGTTAGTCGTTTTCTGTTTCTCAAGTATCCTCAAAAGCACGAGGGTCAGCTTACA
AGGATGAGGCAACAAATGGTTAGTAATATGGTTCTTTATCAGTTTGCTCTGGTTAAAGGG
CTTCAGTCATATATCCAGGCGGATCGATTCGCCCCGTCTAGGTGGTCTGCTCCTGGTGTG
CCTCCGGTTTTCGACGAGGACACAAAAGATGGAGGATCTTCGTTTTTCGATGAAGAGCA
AAAACCTGTTTCCGAGGAAAACAGCGATGTGTTTGAAGATGGGGAGATGGAGGATGGT
GAACTAGAGGGTGATTTGAGTTCGTACCGAGTTTTATCTAGCAAAACGTTAGCTGATGTT
GTTGAGGCTTTGATTGGTGTTTATTACGTCGAAGGGGGTAAGATTGCAGCTAATCATTTG
ATGAAATGGATTGGGATTCACGTGGAGGATGATCCTGATGAAGTCGATGGAACATTGAA
AAATGTTAATGTTCCAGAGAGTGTGCTCAAGAGCATCGACTTTGTTGGTCTTGAGAGAG
CTCTTAAATATGAGTTTAAAGAGAAAGGTCTTCTTGTTGAAGCTATAACACATGCTTCAA
GACCATCTTCAGGTGTTTCGTGTTACCAGAGATTGGAATTTGTTGGTGACGCGGTCTTGG
ATCATCTCATCACAAGACATCTATTTTTCACATACACAAGCCTTCCTCCTGGTCGGTTAAC
AGATCTTCGAGCTGCAGCGGTTAACAACGAGAATTTTGCTCGCGTTGCGGTTAAACATA
AACTCCACTTGTACCTTCGTCACGGTTCAAGCGCCCTCGAAAAACAGATTCGGGAATTT
GTGAAGGAGGTTCAAACCGAGTCATCGAAACCGGGGTTTAACTCTTTTGGTTTGGGAGA
CTGCAAAGCACCAAAAGTTCTTGGAGACATTGTTGAATCTATTGCAGGTGCTATTTTTCT
TGATAGTGGAAAAGATACAACTGCTGCTTGGAAGGTTTTTCAACCTTTGCTTCAGCCCAT
GGTGACACCAGAGACACTTCCAATGCATCCGGTGCGAGAGCTACAAGAGCGGTGCCAG
CAACAAGCAGAAGGGTTAGAATACAAAGCGAGTAGGAGTGGTAACACAGCGACTGTGG
AAGTTTTCATCGACGGTGTTCAAGTTGGAGTAGCGCAAAACCCGCAGAAGAAAATGGC
TCAAAAGCTAGCTGCGAGGAACGCACTTGCAGCTTTGAAAGAGAAAGAAATAGCAGAA
TCAAAGGAGAAGCATATCAACAACGGTAATGCGGGAGAGGATCAAGGCGAGAATGAGA
ATGGGAACAAGAAGAATGGGCATCAGCCGTTTACGAGACAAACGTTGAATGATATTTGT
TTGAGGAAGAATTGGCCAATGCCTTCTTACAGATGTGTGAAAGAAGGAGGACCGGCTCA
TGCAAAGAGATTTACGTTTGGGGTAAGAGTTAATACGAGCGATAGAGGATGGACCGATG
AGTGTATTGGCGAGCCAATGCCGAGTGTTAAGAAAGCTAAGGATTCAGCTGCGGTTCTT
CTACTTGAGCTTTTAAATAAAACTTTTTCTTGA

>AT1G02860|AT1G02860.1

ATGAAGTTTTGTAAGAAGTATGAAGAGTACATGCAAGGACAGAAGGAGAAGAAGAATC

TTCCTGGTGTTGGGTTTAAGAAACTCAAGAAGATTCTCAAGAGATGCAGGAGAAATCAT
GTTCCTTCTAGAATTTCTTTTACTGATGCAATCAACCACAATTGTTCTCGTGAATGCCCAG
TTTGTGATGGGACTTTTTTCCCGGAGCTTCTCAAGGAAATGGAAGATGTTGTTGGATGGT
TTAACGAGCATGCTCAGAAGCTTCTTGAGCTTCATTTAGCTTCTGGTTTTACAAAGTGTC
TTACTTGGCTCAGAGGCAACAGTCGAAAAAAGGACCATCATGGTTTGATCCAAGAGGGT
AAAGATTTGGTTAATTACGCTCTCATCAATGCCGTCGCCATTCGAAAAATCCTCAAGAAA
TATGACAAGATTCATGAGTCTAGGCAAGGACAAGCGTTTAAGACTCAGGTCCAGAAAAT
GCGAATAGAAATCCTTCAGTCACCGTGGCTCTGCGAGCTTATGGCGTTTCACATCAATCT
GAAAGAATCTAAGAAGGAATCTGGAGCTACTATAACTTCTCCTCCTCCTCCTGTTCATGC
ATTGTTTGATGGTTGCGCTTTGACTTTCGACGATGGGAAGCCTTTACTTTCCTGCGAGCT
CTCTGATTCCGTCAAAGTTGACATTGACTTGACTTGTTCAATATGCCTGGACACGGTGTT
TGATCCAATATCTCTAACCTGCGGTCACATATATTGCTACATGTGTGCTTGCTCTGCTGCAT
CAGTAAACGTAGTTGATGGCTTGAAAACCGCAGAAGCAACTGAAAAATGCCCGCTTTG
CCGTGAGGATGGGGTTTATAAAGGTGCTGTTCACTTGGATGAGCTCAATATTTTACTTAA
GCGAAGCTGCAGAGACTATTGGGAAGAAAGGCGTAAAACAGAGAGAGCAGAAAGGTT
ACAACAAGCAAAGGAATATTGGGATTACCAATGCCGAAGCTTCACTGGAATATGA

>AT1G27340|AT1G27340.1

ATGGAAGAAGAGCTTGCCATGCTTAGACAGCTCATCGGTCAGCTTCAAGAGCTCTTGCA
CAACGGCTCTCCTCCTCCTCCTTCTTCTTCTTCATCTCTGTCGTCTTCTTCTCCGTCATTTT
TGGTTCTCCACCATCCTCAGTATCAGAACGGATGGTGTTTGCCCTGTATTGAGGATACTT
CTGCTGATGATTGTTGTGATATTGTAATGGCTGGTGGAAAGAGGCCTGGGATCTTCAAGA
TGTTAGAAACTGTCAAGCCTCCCGTCAAACGAACTCGAAAAGAACGGACTCAAGGGAA
ATCATGTACGGAAGTAGATGAGATAAGTGGGAACATGGATCAAGAAATATGGCAGGAAT
TCCCTCAAGATCTCTTTGAAGACGTTGTTTCCAGACTACCAATGGCTACTTTTTTCCAGT
TCCGTGCAGTTTGCCGTAAATGGAATGCTCTTATTGATTCAGATAGCTTCTCCAGATGCTT
CACTGAGCTTCCTCAGACCATCCCATGGTTCTACACCATAACCCACGAGAATGTCAACTC
AGGACAAGTGTACGACCCTTCTTTGAAGAAATGGCACCATCCGATTATCCCAGCACTAC
CCAAGAAGAGTATTGTTTTGCCAATGGCTTCTGCAGGAGGTCTAGTGTGCTTCCTCGACA
TTGGTCATAGGAACTTCTACGTGAGCAACCCGCTTACCAAGTCTTTCAGGGAGTTGCCT
GCTAGGTCGTTCAAGGTCTGGTCTCGTGTTGCGGTAGGAATGACTCTGAATGGAAACTC
CACCAGTCATGGGTATAAGGTCTTGTGGGTTGGATGTGAAGGAGAGTACGAAGTCTATG
ATTCCCTGAGTAATGTGTGGACCAAACGAGGGACCATCCCGTCCAACATAAAGCTCCCG
GTGTTGCTCAACTTTAAGTCACAGCCAGTGGCTATCCACAGCACACTTTACTTCATGTTA
ACAGATCCCGAAGGGATATTGTCCTATGACATGGTCTCTGGGAAATGGAAACAGTTCATT
ATACCTGGCCCACCAGACCTGAGCGATCACACGCTGGCTGCGTGCGGAGAGCGGTTGAT
GCTGGTGGGTCTACTGACTAAAAACGCTGCCACTTGCGTTTGCATATGGGAGCTGCAGA
AGATGACACTGTTGTGGAAGGAGGTTGACAGAATGCCAAACATATGGTGCTTGGAGTTT
TACGGAAAGCACATTAGGATGAATTGTCTGGGCAACAAAGGTTGTCTGATATTATTGTCC
TTGAGGTCCAGACAGATGAACCGTCTGATAACCTACAATGCTGTGACTAGGGAATGGAC
CAAGGTCCCTGGCTGCACCGTTCCTCGTGGGAGGAAAAGACTTTGGATCGCTTGCGGA
ACAGCGTTTCATCCCTCCCCTACAGCTAGGGCTTGA

>AT1G30490|AT1G30490.1

ATGATGGCTCATCACTCCATGGACGATAGAGACTCTCCTGATAAAGGATTTGATTCCGGC
AAGTACGTTAGATACACGCCGGAACAAGTTGAAGCTCTTGAGAGAGTTTATGCTGAGTG
TCCTAAACCTAGCTCTCTGAGAAGACAACAGCTTATTCGTGAATGTCCCATTCTCTGTAA
CATCGAGCCTCGACAGATCAAAGTTTGGTTCCAGAATCGCAGATGTCGAGAGAAGCAG
AGGAAAGAGTCAGCTCGTCTTCAGACAGTGAACAGGAAGCTGAGTGCTATGAACAAGC
TTTTGATGGAAGAGAATGATCGTTTGCAGAAGCAAGTCTCCAACTTGGTTTATGAGAAT

GGATTCATGAAACATCGAATCCACACTGCTTCTGGGACGACCACAGACAACAGCTGTGA
GTCTGTGGTCGTGAGTGGTCAGCAACGTCAGCAGCAAAACCCAACACATCAGCATCCT
CAGCGTGATGTTAACAACCCAGCTAATCTTCTCTCGATTGCGGAGGAGACCTTGGCGGA
GTTCCTTTGCAAGGCTACAGGAACTGCTGTCGACTGGGTCCAGATGATTGGGATGAAGC
CTGGTCCGGATTCTATTGGTATCGTAGCTGTTTCACGCAACTGCAGTGGAATAGCAGCAC
GTGCCTGTGGCCTCGTGAGTTTAGAACCCATGAAGGTCGCTGAAATCCTCAAAGATCGT
CCATCTTGGTTCCGTGACTGTCGATGTGTCGAGACTCTGAATGTTATACCCACTGGAAAT
GGTGGTACTATCGAGCTTGTCAACACTCAGATTTATGCTCCTACAACATTAGCAGCAGCT
CGTGACTTTTGGACGCTGAGATATAGTACAAGTCTAGAAGATGGAAGCTATGTGGTCTGT
GAGAGATCACTCACTTCTGCAACTGGTGGCCCCAATGGTCCACTTTCTTCAAGCTTCGT
GAGAGCCAAAATGCTGTCAAGCGGGTTTCTTATCCGTCCTTGTGATGGTGGTGGTTCCAT
TATTCACATCGTTGATCATGTGGACTTGGATGTCTCAAGTGTTCCTGAAGTCCTCAGGCC
TCTTTATGAGTCTTCCAAAATCCTTGCTCAAAAAATGACTGTCGCTGCTCTGAGACATGT
GCGCCAAATTGCTCAAGAGACTAGTGGAGAAGTCCAGTATAGTGGTGGACGCCAGCCT
GCAGTTTTAAGGACTTTCAGCCAGAGACTCTGCCGGGGTTTCAATGATGCTGTAAATGG
TTTTGTCGATGATGGATGGTCTCCAATGAGTAGTGATGGAGGAGAGGATATTACGATCAT
GATTAACTCTTCCTCTGCTAAATTTGCTGGCTCCCAATACGGTAGCTCATTTCTTCCAAGT
TTTGGAAGTGGTGTCCTCTGTGCCAAAGCTTCTATGCTGTTGCAGAATGTTCCACCCCTT
GTATTGATTCGGTTCCTGAGAGAACACCGAGCTGAATGGGCAGACTATGGTGTCGATGC
CTATTCTGCTGCATCTCTCAGAGCAACTCCATATGCTGTTCCATGCGTCAGAACCGGTGG
GTTCCCGAGTAACCAAGTCATTCTTCCTCTCGCACAGACACTCGAACATGAAGAGTTTC
TCGAAGTGGTTAGACTTGGAGGTCATGCTTACTCACCTGAAGACATGGGCTTATCCCGG
GATATGTATTTACTGCAGCTTTGTAGCGGCGTTGATGAAAATGTGGTTGGAGGTTGTGCT
CAGCTTGTCTTTGCCCCAATCGATGAATCATTTGCTGATGATGCACCTTTGCTTCCTTCTG
GTTTCCGTGTCATACCACTCGACCAAAAAACAAATCCGAATGATCATCAATCTGCAAGTC
GAACACGGGATCTAGCATCGTCCCTAGATGGTTCCACCAAAACCGATTCGGAAACAAAC
TCTAGATTGGTCTTAACAATAGCCTTCCAGTTCACGTTTGATAACCATTCCAGAGACAAT
GTTGCTACAATGGCGAGACAGTATGTGAGGAACGTTGTTGGTTCGATTCAGAGAGTGGC
TCTAGCCATTACGCCTCGTCCTGGCTCAATGCAACTTCCCACTTCCCCTGAAGCTCTCAC
TCTTGTCCGTTGGATCACCCGTAGTTACAGTATTCATACAGGTGCAGATCTGTTTGGAGC
TGATTCTCAGTCCTGTGGAGGAGACACATTGCTTAAGCAACTCTGGGACCATAGTGATG
CCATATTGTGCTGCTCCCTGAAAACTAATGCCTCACCGGTATTCACATTTGCAAACCAAG
CTGGTTTAGACATGCTTGAAACTACACTTGTGGCACTTCAGGATATAATGCTCGACAAAA
CACTTGATGACTCTGGTCGTAGAGCTCTTTGCTCCGAGTTCGCCAAGATCATGCAGCAG
GGATATGCGAATCTTCCGGCAGGAATATGTGTGTCGAGCATGGGCAGACCGGTTTCGTAT
GAGCAAGCGACGGTGTGGAAAGTTGTTGATGACAACGAATCAAACCACTGCTTGGCTT
TTACCCTCGTTAGTTGGTCGTTTGTTTGA

>AT1G31280|AT1G31280.1

ATGGAGAGAGGTGGTTATCGAGGAGGTCGTGGTGATGGCCGTGGTAGAGGTGGCCGTG
GTTATGGCGGAGGCGGAGGAGGAGGAGAACAAGGTCGTGATCGTGGCTACGGCGGCGG
AGAACAAGGTCGTGGTCGTGGCTCAGAGCGAGGCGGCGGAAATCGTGGTCAAGGTCGT
GGTGAACAACAGGATTTTCGAAGCCAGAGTCAGCGGGGACCTCCGCCAGGTCACGGTG
GCCGTGGGACGACGCAGTTCCAACAGCCTCGACCACAGGTGGCTCCGCAGCCGTCGCA
GGCTCCGGCGAGTTATGCCGGTTCAGTAGGAGGAGTCGCTGGTAGAGGCGCGTGGGGT
CGTAAGCCACAGGTTCCGTCTGATTCGGCTTCTCCGTCCACCAGCACCACCGTGGTTTCT
GAACCCGTTCGTGTAGCTGAAGTTATGAATCTGAAGCCATCGGTGCAAGTTGCGACTTC
TGATAGGAAAGAACCAATGAAGCGACCTGATAGAGGCGGAGTTGTGGCTGTGCGGCGT
GTTAATCTATATGTGAATCATTATAAAGTGAATTTCAATCCTGAAAGTGTTATAAGACATTA
TGATGTTGAAATCAAAGGAGAAATTCCTACCAAGAAGGTTTCGAGGTTTGAGCTAGCTA
TGGTTAGGGACAAGGTGTTCACTGACAATCCCGATGAGTTTCCCTTAGCTATGACAGCTT

ATGATGGTCAGAAGAACATTTTCAGTGCGGTTGAGTTACCTACGGGGTCATACAAGGTG
GAGTATCCTAAAACTGAAGAGATGAGAGGTCGAAGCTATACGTTCACTATCAAACAGGT
GAATGTGCTGAAGCTAGGTGACTTGAAAGAGTACATGACAGGGAGATCGTCTTTCAATC
CGCGTGATGTGTTGCAAGGAATGGATGTTGTGATGAAGGAGCATCCTTCCAAGTGTATGA
TCACTGTTGGTAAAAGCTTTTTCACTCGTGAAACTGAGCCAGATGAAGATTTTCGTTTCG
GGGTTATAGCTGCGAAAGGGTATCGCCACACTCTGAAGCCCACAGCACAAGGTTTGTCT
TTGTGTTTGGATTACTCGGTGTTGGCGTTCCGCAAAGCAATGTCGGTCATTGAATACCTG
AAGTTGTACTTTAACTGGTCTGATATGCGTCAGTTTAGGAGGCGTGATGTGGAAGAGGA
ATTGATTGGTTTGAAAGTCACTGTCAATCATCGGAAGAACAAGCAGAAACTCACCATTG
TAGGGCTGAGTATGCAAAACACAAAAGACATCAAATTTGATCTTATTGATCAAGAGGGA
AACGAGCCGCCAAGGAAGACGTCCATTGTTGAGTATTTCAGGATAAAGTATGGAAGACA
CATTGTTCACAAGGATATACCTTGCTTGGATTTGGGAAAAAACGGTAGGCAAAATTTTGT
GCCCATGGAATTCTGTGACTTGGTTGAGGGACAGATATATCCAAAGGATAACTTGGATAA
AGATTCAGCTTTGTGGCTAAAAAAGTTGTCACTGGTCAATCCACAACAAAGGCAGAGG
AATATAGATAAGATGATAAAGGCTCGTAATGGACCGAGCGGTGGTGAAATCATTGGAAA
CTTTGGATTGAAAGTGGATACAAACATGACACCGGTGGAAGGTCGTGTACTCAAGGCTC
CATCATTGAAGTTGGCAGAGAGAGGGAGAGTTGTGCGTGAGGAACCCAACCCGAGACA
GAACAACCAATGGAACCTTATGAAGAAGGGAGTCACAAGGGGATCTATAGTCAAGCATT
GGGCTGTACTTGACTTCACTGCATCCGAGAGATTTAACAAGATGCCTAATGACTTTGTGG
ATAACCTTATTGATCGTTGTTGGAGACTTGGGATGCAGATGGAGGCTCCTATCGTTTACA
AAACATCGAGAATGGAAACACTTTCTAATGGTAATGCTATTGAGGAATTGCTTCGATCCG
TGATAGATGAAGCTTCTCGTAAGCATGGTGGGGCTCGTCCAACTCTTGTTCTGTGTGCTA
TGTCTCGGAAAGACGATGGCTATAAGACTCTGAAATGGATAGCCGAGACCAAACTTGGT
CTGGTGACTCAGTGTTTCTTGACTGGTCCTGCCACTAAAGGAGGTGATCAGTACCGGGC
AAATCTTGCCCTCAAGATGAACGCAAAGGTTGGTGGAAGCAATGTCGAGCTTATGGATA
CTTTCTCTTTCTTCAAAAAAGAGGATGAGGTCATGTTCATTGGTGCTGATGTCAATCATC
CCGCTGCTCGGGACAAGATGAGCCCGTCCATTGTTGCTGTTGTGGGAACTCTTAACTGG
CCTGAAGCAAATCGCTATGCAGCTAGAGTCATTGCCCAGCCTCACCGCAAAGAGGAAAT
ACAAGGATTTGGCGACGCTTGTTTGGAGCTTGTCAAAGCTCATGTTCAGGCCACAGGGA
AACGGCCTAACAAGATTGTGATATTCCGTGATGGTGTCAGCGATGCTCAGTTCGATATGG
TTCTCAATGTGGAGTTGCTTGATGTTAAGCTAACTTTTGAGAAGAATGGTTACAATCCAA
AGATAACGGTAATCGTAGCCCAGAAACGTCATCAAACCCGTTTCTTCCCAGCCACAAAT
AATGATGGAAGTGATAAGGGCAATGTGCCTTCAGGTACGGTTGTTGATACTAAAGTTATT
CACCCGTATGAGTATGATTTCTACCTCTGCAGTCACCACGGAGGGATAGGGACAAGCAA
ACCGACTCATTACTACACTCTTTGGGACGAACTTGGATTCACTTCGGATCAGGTGCAGA
AGCTCATCTTCGAGATGTGCTTCACTTTCACTCGCTGCACCAAACCCGTCTCTCTTGTTC
CGCCGGTGTATTATGCTGACATGGTTGCTTTTAGAGGAAGGATGTACCACGAGGCAAGC
TCTCGTGAGAAGAACTTTAAGCAGCCGCGGGGAGCGTCAACCTCTGCTGCTTCGCTTGC
CTCTTCATTATCTTCTCTTACAATTGAGGACAAAGCGATTTTCAAGCTGCATGCAGAGCT
TGAGAATGTTATGTTCTTCGTCTGA

>AT1G48410|AT1G48410.1

ATGGTGAGAAAGAGAAGAACGGATGCTCCATCTGAAGGAGGTGAAGGCTCTGGGTCTC
GTGAAGCTGGTCCAGTCTCAGGTGGTGGACGTGGTTCACAGCGAGGTGGTTTCCAGCA
GGGAGGAGGACAACACCAAGGTGGAAGGGGTTATACTCCTCAACCTCAACAGGGAGGT
CGTGGTGGTCGTGGATATGGGCAACCACCACAACAGCAACAACAGTATGGAGGACCAC
AAGAGTACCAAGGAAGAGGAAGAGGAGGACCTCCTCATCAAGGAGGTCGAGGAGGGT
ATGGCGGTGGCCGTGGAGGTGGACCTTCTTCTGGACCACCGCAGAGACAATCAGTTCCC
GAGCTGCATCAAGCTACCTCACCTACTTATCAAGCGGTGTCTTCTCAGCCTACACTGTCT
GAGGTGAGTCCTACCCAGGTACCAGAACCTACTGTTCTGGCTCAGCAATTTGAACAACT
CTCTGTTGAACAAGGAGCTCCCAGTCAGGCAATCCAGCCTATACCTTCTTCTAGCAAGG

CTTTCAAGTTTCCAATGAGGCCTGGTAAAGGACAGAGTGGAAAGCGTTGCATTGTGAAG
GCTAACCATTTCTTTGCTGAACTGCCTGATAAGGATTTGCACCATTATGATGTTACCATTA
CTCCGGAAGTTACATCAAGGGGTGTCAATCGTGCTGTGATGAAACAACTTGTTGATAATT
ATCGTGATTCTCACCTTGGAAGTCGTCTTCCAGCGTATGATGGTCGAAAAAGTCTTTACA
CTGCTGGTCCACTTCCCTTTAACTCCAAGGAGTTCAGAATCAATCTTCTTGACGAAGAA
GTAGGGGCTGGAGGTCAAAGACGAGAAAGGGAATTTAAAGTTGTGATCAAGCTAGTTG
CACGTGCTGATCTGCATCACCTAGGAATGTTTTTGGAGGGGAAACAATCAGATGCCCCA
CAGGAAGCTCTGCAGGTTCTTGACATTGTTCTTCGTGAGCTGCCGACCTCTAGGTATATT
CCGGTGGGCCGGTCCTTTTATTCCCCTGATATAGGAAAAAAACAATCATTGGGGGATGGC
TTGGAGAGCTGGCGTGGATTCTACCAAAGCATTCGTCCTACACAGATGGGCTTATCACTC
AATATTGATATGTCATCGACAGCCTTCATAGAGGCAAACCCTGTGATTCAGTTTGTCTGTG
ATTTGCTTAACCGGGATATTTCTTCTCGACCTTTATCTGATGCTGATCGTGTTAAGATAAA
AAAGGCTCTTAGAGGTGTCAAAGTTGAAGTGACTCATCGAGGAAACATGCGCCGGAAG
TACCGCATTTCCGGTTTGACTGCTGTGGCCACTCGGGAATTGACATTCCCAGTAGATGAA
AGAAATACTCAGAAATCTGTTGTAGAATACTTCCACGAAACATATGGTTTTCGCATTCAG
CACACTCAACTACCATGCTTGCAAGTTGGGAATTCTAATAGGCCTAATTACTTACCAATG
GAGGTATGCAAGATTGTTGAAGGCCAGCGGTATTCCAAAAGATTGAATGAGAGACAGAT
CACTGCTTTGCTGAAGGTTACCTGTCAGCGCCCGATAGATCGAGAAAAAGATATCTTACA
GACGGTGCAACTCAATGATTATGCTAAAGATAATTATGCTCAAGAGTTTGGCATCAAAAT
AAGTACTTCTCTGGCTTCTGTTGAGGCTCGTATACTGCCTCCTCCATGGCTTAAGTACCA
CGAGTCTGGAAGGGAAGGGACTTGTCTGCCACAAGTTGGTCAATGGAACATGATGAATA
AGAAAATGATCAATGGTGGAACGGTGAATAATTGGATCTGCATCAACTTTTCTAGGCAA
GTGCAGGACAATCTAGCGCGTACATTTTGTCAGGAACTTGCTCAAATGTGTTACGTATCT
GGCATGGCATTTAATCCGGAACCAGTCCTCCCACCAGTCAGTGCTCGCCCTGAGCAAGT
AGAGAAGGTCTTGAAGACTAGATATCATGATGCCACATCAAAACTCTCCCAAGGAAAAG
AAATTGATCTGCTTATTGTCATTCTGCCCGATAATAATGGATCATTATACGGTGATTTGAAA
CGCATATGTGAGACTGAACTCGGCATAGTCTCTCAATGTTGCCTGACAAAGCATGTCTTT
AAGATGAGCAAACAATACATGGCTAATGTTGCGCTGAAGATTAATGTGAAGGTTGGAGG
AAGAAACACAGTGCTTGTTGATGCTCTATCTAGGCGGATTCCTCTAGTCAGTGATCGACC
CACCATTATATTTGGTGCTGATGTTACCCACCCTCACCCTGGAGAGGATTCAAGCCCATCT
ATTGCTGCTGTTGTGGCATCTCAGGATTGGCCTGAAATCACTAAATATGCTGGATTAGTTT
GCGCTCAAGCGCATAGGCAGGAGCTCATTCAGGATCTGTTCAAAGAGTGGAAGGATCCT
CAGAAAGGTGTGGTGACTGGTGGCATGATAAAGGAGTTGCTCATAGCCTTCCGTAGATC
AACTGGGCATAAACCACTAAGGATCATCTTCTACAGGGATGGAGTCAGTGAGGGACAAT
TTTACCAAGTTTTGCTCTATGAACTTGATGCCATCCGCAAGGCCTGTGCTTCGCTGGAAG
CAGGTTATCAACCACCAGTGACATTTGTGGTGGTGCAGAAGCGTCATCACACGAGGCTG
TTTGCTCAGAACCACAATGATCGCCATTCGGTGGACAGAAGTGGGAATATTTTACCTGGC
ACTGTTGTGGACTCTAAAATCTGCCACCCTACAGAGTTTGACTTTTACCTCTGTAGTCAT
GCTGGTATTCAGGGCACTTCTCGACCTGCTCATTACCACGTTCTTTGGGATGAGAACAAC
TTTACTGCAGATGGACTTCAATCTCTGACCAATAACTTATGTTACACGTATGCAAGATGCA
CACGCTCAGTTTCAATTGTTCCCCCTGCATATTATGCACATCTAGCAGCTTTTAGGGCTCG
ATTCTACATGGAGCCAGAGACATCAGACAGTGGCTCAATGGCTAGTGGGAGCATGGCAC
GTGGAGGTGGAATGGCTGGTAGAAGCACACGCGGGCCTAATGTCAATGCTGCAGTGAG
GCCACTCCCAGCTCTGAAAGAGAATGTGAAGCGTGTCATGTTCTACTGCTGA

>AT1G17590|AT1G17590.3

ATGGATAAGAAAGTTTCATTTACTAGCTCTGTGGCACATTCAACTCCACCATACCTTAGTA
CTTCCATCTCATGGGGACTTCCAACCAAATCCAATGGTGTGACTGAATCACTGAGTTTGA
AGGTGGTAGATGCAAGACCAGAACGTCTTATAAACACAAAGAATATCAGTTTCCAGGAC
CAGGATTCATCTTCAACTCTGTCCTCTGCTCAATCTTCTAACGATGTTACAAGTAGTGGA
GATGATAACCCCTCAAGACAAATCTCATTTTTAGCACATTCAGATGTTTGTAAAGGATTT

GAAGAAACTCAAAGGAAGCGATTTGCAATTAAATCAGGCTCCTCCACGGCAGGAATCG
CTGATATTCACTCTTCTCCTTCCAAGGCTAACTTCTCATTTCACTATGCCGATCCACATTTT
GGTGGTTTAATGCCTGCGGCTTACCTACCACAGGCAACAATATGGAATCCCCAAATGACT
CGAGTTCCGCTACCATTCGATCTCATAGAGAATGAGCCTGTCTTTGTCAATGCAAAGCAA
TTCCATGCAATTATGAGGAGGAGGCAACAGCGTGCTAAGCTAGAGGCGCAAAACAAAC
TAATCAAAGCCCGTAAGCCGTATCTTCATGAATCTCGACATGTTCACGCTCTTAAACGAC
CTAGAGGATCTGGTGGAAGATTCCTAAACACCAAAAAGCTTCAAGAATCTACAGATCCA
AAACAAGACATGCCAATCCAACAGCAACACGCAACGGAAACATGTCAAGATTTGTGC
TTTATCAGTTGCAGAACAGCAATGACTGTGATTGTTCAACCACTTCTCGCTCTGACATCA
CATCTGCTTCTGACAGCGTTAATCTCTTTGGACACTCTGAATTTCTGATATCAGATTGCCC
ATCTCAGACAAACCCAACAATGTATGTTCATGGTCAATCAAATGACATGCATGGAGGTAG
GAACACACACCATTTCTCTGTCCATATCTGA

>AT1G12820|AT1G12820.1

ATGAATTATTTCCCAGACGAGGTTATAGAGCACGTGTTTGACTTCGTAGCTTCTCACAAA
GACAGGAACTCGATATCTCTGGTCTGCAAATCATGGCACAAGATCGAGAGGTTTAGTAG
GAAGGAAGTGTTCATCGGAAACTGCTACGCGATTAACCCGGAGAGGTTGATCAGGAGG
TTTCCATGTCTCAAATCCTTAACTTTAAAAGGGAAGCCTCATTTTGCAGACTTCAACTTG
GTTCCTCATGAATGGGGAGGTTTCGTGCATCCTTGGATTGAAGCTTTGGCTAGAAGCCGT
GTGGGACTTGAGGAGCTGAGGTTGAAGCGGATGGTTGTAACAGATGAAAGCTTGGACC
TTCTTTCACGTTCTTTTGCAAATTTCAAGTCTTTGGTTCTTGTTAGCTGTGAAGGGTTTAC
CACTGATGGCTTAGCTTCCATTGCCGCTAATTGCAGGCATCTTCGTGAGCTGGACTTGCA
AGAGAATGAGATTGATGATCATAGAGGTCAATGGCTGAACTGTTTTCCAGATAGCTGCAC
TACTCTTATGTCGTTGAATTTCGCTTGCCTTAAAGGAGAGACCAATGTTGCTGCTTTAGA
AAGGCTTGTTGCTAGGTCACCAAACCTGAAGAGCTTGAAGTTAAACCGTGCAGTACCGC
TTGACGCACTCGCAAGGTTAATGAGTTGTGCGCCGCAGCTAGTGGACTTAGGAGTAGGG
TCTTATGAGAATGAGCCAGATCCTGAATCTTTTGCAAAACTCATGACTGCCATTAAGAAA
TACACATCGTTAAGGAGCTTGTCTGGCTTTTTAGAGGTTGCTCCACTCTGCCTCCCAGCG
TTCTACCCAATTTGCCAAAACCTTATCTCTTTGAACCTCAGCTATGCAGCTGAAATCCAA
GGCAACCACCTCATTAAGCTTATTCAGCTTTGCAAGAGACTTCAACGATTATGGATATTG
GATAGTATTGGTGACAAAGGACTTGCGGTTGTCGCTGCCACATGTAAAGAGTTACAAGA
GCTTAGAGTTTTTCCCTCTGATGTACATGGTGAAGAAGATAACAACGCATCTGTGACTGA
GGTTGGACTAGTCGCCATTTCCGCAGGTTGCCCTAAACTTCATTCGATTCTGTACTTCTG
CAAACAGATGACAAACGCAGCGCTCATAGCCGTGGCCAAAAACTGTCCAAACTTCATCC
GGTTCAGGCTATGCATTCTCGAGCCACACAAACCTGACCACATTACATTTCAATCACTGG
ACGAGGGCTTTGGTGCAATCGTACAAGCTTGCAAGGGTCTAAGACGGCTCTCTGTCTCC
GGTCTCTTAACCGATCAAGTCTTTCTCTACATCGGTATGTACGCGGAACAGCTCGAGATG
CTTTCGATAGCTTTTGCGGGGGACACTGACAAAGGAATGCTCTATGTGTTGAATGGATGC
AAAAAAATGAGGAAGCTGGAGATAAGGGACAGTCCTTTTGGGAACGCTGCGCTTCTTG
CTGACGTGGGTAGGTACGAAACAATGCGATCCCTTTGGATGTCGTCTTGTGAAGTAACA
CTCGGTGGCTGCAAGAGGCTCGCGCAGAATTCGCCACGGCTTAACGTAGAGATCATCAA
CGAGAATGAGAATAATGGGATGGAACAGAATGAAGAAGATGAAAGAGAGAAGGTTGAT
AAACTTTACCTCTACCGAACAGTGGTTGGGACTAGAAAAGATGCACCACCATATGTTAG
GATTCTTTAG

>AT1G27370|AT1G27370.1

ATGGACTGCAACATGGTATCTTCGTTCCCGTGGGACTGGGAGAATTTGATCATGTCCAAT
CAGTCGAAGACTGAAAATGAAAAAAAAACAGCAATCTACTGAGTGGGAATTTGAAAAAG
GTGAAGGAATTGAATCTATAGTTCCAGATTTCTTAGGCTTTGAGAAAGTCAGTAGTGGCT
CTGCTACTAGTTTCTGGCACACTGCCGTATCAAAAAGCTCGCAGTCGACCTCTATCAACT

CATCATCTCCCGAGGACAAACGATGCAATCTTGCATCACAAAGTTCCCCTGGAGATTCTT
CCAGCAACATAGATTTTCTCCAGGTGAAACCATCCACAGCTCTCGAGGTACCTATTGCCT
CAGCTGAATCAGATCTTTGTTTGAAACTAGGAAAGCGGACATACTCTGAAGAATTTTGG
GGTAGGAACAATAATGACCTTTCAGCGGTTTCTATGAATTTGTTGACTCCATCTGTTGTTG
CTCGGAAGAAAACCAAATCGTGTGGTCAGAGCATGCAAGTTCCGCGTTGCCAAATTGAT
GGCTGTGAGCTGGATCTCTCATCTTCTAAGGATTATCATCGCAAGCATAGAGTCTGCGAA
ACGCATTCAAAGTGCCCAAAAGTTGTTGTGAGTGGCCTGGAACGTCGTTTCTGCCAACA
GTGTAGCAGGTTCCATGCTGTCTCAGAATTTGATGAAAAGAAACGAAGCTGCCGCAAAC
GTCTTTCTCATCATAATGCAAGGCGTCGCAAGCCACAAGGAGTATTTCCACTGAATTCAG
AGAGGGTGTTCGATCGAAGACAGCATACAAGTATGTTGTGGAATGGGTTGTCCCTTAAC
ACGAGATCTGAAGAAAAGTATACATGGGGTACCACTTATGAGACAAAGCCTACACAGAT
GGAAAGCGGCTTTACTCTGAGCTTCCAGAGAGGTAATGGCTCTGAGGACCAACTGTTTA
CTGGTAGCACCCTCTCTTTCTCTGCGTTTCAAACATCTGGTGGGTTCTCAGCAGGGAAAT
CCAACATTCAACTTCCAGACAAAGGTGTGGGAGAATGCTCAGGAGGCCTCCATGAATCT
CATGATTTCTACAGTGCTCTCTCTCTTCTGTCAACTACTTCGGATTCACAAGGGATCAAA
CACACTCCCGTGGCCGAACCACCGCCAATATTTGGCACTTTCCCTAGTCATTTCATCTGA

>AT1G30330|AT1G30330.1

ATGAGATTATCTTCAGCTGGGTTTAATCCTCAACCTCATGAAGGAGAGAAAAGAGTTCTT
AATTCTGAGCTCTGGCATGCTTGTGCTGGTCCTCTTGTCTCACTACCTCCTGTTGGAAGC
AGAGTTGTGTATTTTCCTCAAGGTCACAGTGAACAGGTTGCTGCTTCGACCAACAAAGA
AGTAGATGCTCATATACCAAATTATCCGAGCTTGCATCCGCAGCTTATCTGTCAGCTTCAT
AATGTTACAATGCATGCTGATGTGGAGACTGATGAAGTCTATGCACAGATGACTTTGCAA
CCGTTGAATGCGCAAGAGCAAAAAGATCCTTACCTTCCCGCGGAATTAGGTGTCCCAAG
TAGACAGCCTACAAACTATTTCTGTAAAACTCTGACTGCTAGTGATACAAGCACTCACGG
AGGTTTTTCTGTACCTCGCCGAGCTGCTGAGAAAGTTTTCCCTCCCTTGGATTACTCGCA
GCAGCCACCAGCTCAAGAGTTAATGGCGAGGGATCTGCATGATAATGAATGGAAGTTCA
GGCATATTTTCCGAGGCCAACCAAAGAGACATCTCCTTACCACGGGTTGGAGCGTATTTG
TGAGTGCTAAAAGGCTTGTTGCTGGTGACTCTGTTCTTTTCATCTGGAACGATAAGAATC
AATTACTTCTTGGTATAAGACGAGCCAACCGACCACAAACTGTCATGCCTTCATCTGTTT
TGTCAAGTGACAGTATGCATTTAGGCCTTCTTGCTGCAGCAGCTCATGCTGCCGCTACAA
ACAGCCGATTCACCATCTTCTATAACCCGAGGGCGAGTCCATCAGAGTTTGTTATACCCC
TGGCTAAGTATGTGAAAGCGGTTTATCACACTCGCGTCTCTGTTGGTATGCGGTTTAGGA
TGCTGTTTGAAACTGAAGAATCTAGTGTTCGTCGGTACATGGGTACAATAACTGGCATTT
GTGATCTAGATCCTACTCGTTGGGCTAATTCGCATTGGCGATCTGTCAAGGTTGGGTGGG
ACGAATCTACTGCAGGAGAGAGACAGCCAAGGGTTTCATTGTGGGAGATTGAGCCTTTA
ACAACATTCCCTATGTATCCATCTCCTTTTCCTCTCAGGCTTAAACGGCCGTGGCCTCCTG
GTCTCCCATCTTTCCATGGCCTTAAAGAAGATGATATGGGTATGAGTATGAGTTCACCGCT
TATGTGGGATCGAGGACTCCAATCTCTAAACTTTCAAGGTATGGGAGTAAACCCGTGGAT
GCAGCCGAGACTTGATACGTCGGGCTTGCTTGGTATGCAAAATGATGTGTACCAAGCAA
TGGCTGCAGCTGCCCTTCAAGACATGAGAGGCATTGATCCTGCAAAAGCTGCTGCTTCA
CTTCTTCAGTTCCAAAATTCGCCGGGGTTCTCAATGCAATCTCCGTCCTTAGTGCAGCCG
CAGATGCTGCAGCAGCAACTCTCTCAGCAGCAGCAACAACTCTCTCAGCAGCAGCAGC
AGCAGCAACAGCTCTCCCAACAGCAGCAGCAACAGCTCTCTCAACAGCAGCAGCAGCA
GCTCTCTCAACAGCAGCAGCAACAACTCTCTCAGCAGCAGCAGCAACAGGCGTATCTT
GGCGTTCCTGAAACCCACCAGCCTCAGTCTCAGGCTCAATCACAGTCAAACAATCATCT
TTCTCAGCAGCAGCAGCAAGTAGTGGATAATCATAATCCGTCTGCGTCCAGTGCTGCTGT
TGTTTCCGCTATGTCTCAATTTGGTTCTGCTTCTCAGCCCAACACGTCACCACTCCAGTC
CATGACCTCTCTGTGTCATCAGCAAAGCTTCTCCGATACCAACGGAGGAAACAATCCTAT
TTCTCCACTTCACACTCTTCTCAGTAACTTCTCTCAAGACGAATCTTCTCAACTGCTCCA
CCTCACTAGAACAAACTCTGCAATGACTTCATCCGGTTGGCCATCAAAGCGTCCTGCAG

TTGATTCATCGTTCCAGCACTCTGGAGCCGGTAATAATAACACTCAATCCGTATTGGAGC
AACTGGGACAGTCCCACACAAGCAACGTTCCTCCAAACGCTGTCTCGTTGCCTCCATTT
CCCGGTGGTAGAGAGTGCTCGATCGAGCAAGAAGGAAGCGCCTCAGACCCGCATAGCC
ATCTTCTCTTTGGAGTCAATATAGATTCATCTTCTCTTTTGATGCCGAACGGAATGTCAAA
CCTTAGAAGCATTGGTATTGAAGGTGGTGACTCCACGACTTTACCCTTCACATCATCTAA
TTTCAACAATGATTTCTCGGGTAATCTTGCAATGACAACACCTTCTAGTTGCATAGATGAA
TCGGGTTTTCTACAATCCTCAGAAAACCTCGGTTCCGAGAACCCACAATCTAACACCTTT
GTGAAGGTGTACAAGTCAGGGTCTTTTGGAAGATCGTTAGATATATCAAAGTTTAGCAGC
TACCACGAGCTGCGAAGCGAGCTTGCTCGCATGTTTGGCCTCGAAGGCCAATTAGAAGA
CCCTGTGAGATCAGGCTGGCAGCTTGTATTTGTTGACCGAGAGAACGACGTTCTTCTCC
TCGGCGATGACCCTTGGCCGGAGTTTGTGAGCAGCGTGTGGTGCATTAAGATACTGTCA
CCACAAGAAGTGCAACAAATGGGAAAAAGAGGCCTTGAGCTTCTCAACTCCGCGCCAT
CTTCCAACAATGTCGATAAGCTCCCGAGCAACGGGAACTGTGACGACTTTGGGAACCG
GTCAGACCCGAGGAATCTCGGTAACGGTATCGCATCAGTTGGGGGTTCATTCAACTACTA
G

>AT1G54160|AT1G54160.1

ATGCAAGTGTTTCAAAGGAAAGAAGATTCATCTTGGGGAAACTCAATGCCTACAACAAA
TTCAAATATTCAAGGATCTGAATCTTTCAGCTTGACTAAGGATATGATAATGTCTACAACA
CAATTACCCGCGATGAAACATTCGGGTTTGCAGCTGCAAAATCAAGATTCAACCTCATCA
CAATCTACTGAAGAAGAATCAGGCGGCGGTGAAGTTGCAAGCTTTGGAGAATATAAGCG
TTATGGATGCAGCATTGTTAATAACAATCTCTCAGGTTACATCGAAAACTTGGGAAAGCC
TATTGAAAATTATACTAAGTCAATTACTACCTCGTCGATGGTGTCTCAAGACTCTGTGTTT
CCTGCTCCTACTTCTGGTCAAATATCTTGGTCTCTTCAATGTGCTGAAACGTCACATTTCA
ATGGTTTCTTGGCTCCTGAATATGCATCAACACCAACGGCGCTGCCACATTTAGAGATGA
TGGGTTTGGTTTCTTCAAGAGTGCCATTGCCTCATCACATTCAAGAGAATGAACCAATAT
TTGTCAATGCGAAACAGTATCATGCGATTCTCCGTCGCAGGAAGCACCGTGCTAAACTC
GAAGCTCAGAACAAACTCATCAAATGCCGTAAACCGTACCTTCATGAGTCTCGCCATCT
TCATGCTTTAAAGAGAGCTAGAGGCTCCGGTGGACGTTTCCTCAATACAAAGAAGCTTC
AAGAATCATCAAACTCACTGTGTTCTTCTCAAATGGCAAATGGACAAAATTTCTCTATGA
GCCCTCACGGTGGTGGAAGCGGAATCGGGTCTAGTTCGATCTCACCGAGCTCCAATTCA
AACTGTATCAACATGTTCCAAAACCCGCAGTTCAGATTCTCAGGTTATCCGTCAACACAC
CATGCCTCAGCTCTCATGTCAGGGACTTGA

>AT2G22740|AT2G22740.1

ATGGAAATGGGAGTTATGGAGAATTTGATGGTACATACGGAGATATCAAAGGTTAAGTCT
CAGAGCAATGGTGAGGTGGAGAAACGAGGAGTGTCAGTTTTGGAGAATGGTGGTGTTT
GCAAGCTTGATCGAATGAGTGGGCTTAAGTTTAAGCGTCGGAAGGTTTTTGCTGTGAGG
GACTTTCCACCGGGTTGTGGTTCGAGAGCAATGGAAGTGAAGATTGCTTGTGAGAATGG
GAATGTTGTTGAAGATGTTAAGGTTGTTGAGTCTTTGGTTAAAGAGGAGGAGAGTTTGG
GGCAGAGAGATGCCTCTGAAAACGTGAGTGATATCAGAATGGCTGAGCCGGTGGAGGT
TCAGCCTCTGAGAATTTGTTTGCCAGGAGGTGATGTGGTAAGAGACTTGTCTGTTACCG
CGGGAGACGAGTGTTCAAACAGTGAACAGATTGTGGCGGGTTCTGGTGTTAGCAGCAG
CAGCGGAACTGAAAACATTGTCAGGGATATCGTTGTTTACGCAGATGAGAGCAGTCTAG
GAATGGATAATTTGGATCAAACTCAGCCGCTAGAAATTGAAATGTCAGATGTTGCAGTTG
CAAAACCGAGGCTTGTTGCGGGTAGAAAGAAGGCAAAGAAAGGAATTGCATGTCATTC
ATCCTTGAAGGTTGTTAGCCGAGAGTTTGGTGAAGGATCTAGGAAGAAGAAGAGTAAG
AAGAATTTATATTGGCGTGATAGGGAAAGTCTGGATTCTCCTGAGCAGCTTCGGATACTA
GGTGTTGGGACAAGCAGCGGTTCAAGCAGCGGTGATAGTAGTCGGAACAAAGTAAAGG
AAACTCTACGTCTTTTCCATGGCGTTTGCAGAAAGATTCTGCAGGAGGATGAAGCAAAG

CCTGAGGACCAAAGGAGAAAAGGTAAAGGTCTTAGAATAGATTTTGAGGCTTCGACCAT
TCTCAAAAGGAATGGCAAATTTCTCAACTCTGGTGTGCATATTTTGGGAGAAGTGCCTG
GGGTTGAGGTTGGTGATGAGTTCCAATATAGGATGGAGCTGAACATTCTTGGTATACATA
AACCAAGCCAGGCCGGTATCGATTATATGAAATATGGTAAGGCCAAAGTTGCGACAAGTA
TTGTAGCCTCTGGAGGGTATGACGACCATCTGGATAACTCGGATGTCTTGACCTACACTG
GTCAAGGCGGAAACGTGATGCAAGTAAAGAAGAAGGGAGAGGAACTGAAAGAGCCTG
AAGACCAAAAGCTTATTACCGGAAACCTCGCGTTAGCAACCAGCATAGAAAAACAAAC
CCCTGTGCGTGTCATTAGAGGCAAACATAAGTCAACTCATGATAAATCAAAAGGTGGAA
ATTATGTTTACGACGGATTATATCTTGTGGAAAAGTATTGGCAACAAGTCGGATCTCACG
GTATGAATGTCTTCAAGTTTCAACTTAGGCGCATACCTGGACAACCTGAACTTTCTTGGG
TAGAGGTGAAGAAATCTAAGTCAAAATACCGGGAAGGTCTATGCAAGCTTGACATCTCA
GAAGGGAAAGAGCAGTCACCCATTAGCGCTGTGAACGAAATAGACGACGAGAAACCGC
CTTTGTTCACCTACACTGTTAAACTGATTTACCCAGATTGGTGCAGACCAGTTCCTCCAA
AGTCATGCTGTTGCACCACGCGCTGCACCGAAGCTGAAGCGAGGGTGTGCGCTTGTGT
GGAGAAAAACGGTGGAGAGATACCGTACAACTTCGATGGAGCCATTGTTGGTGCAAAA
CCTACTATCTATGAATGTGGCCCACTCTGCAAATGCCCTTCTAGTTGCTACCTAAGAGTCA
CACAGCATGGGATCAAGTTACCGCTTGAGATCTTCAAAACCAAGTCAAGAGGTTGGGG
AGTGAGATGTCTTAAGTCGATCCCTATTGGTAGCTTCATATGTGAATACGTTGGTGAACTT
TTAGAGGATAGTGAAGCAGAAAGAAGAATCGGGAACGACGAGTATCTCTTTGATATTGG
TAACAGATATGATAACTCTTTAGCTCAAGGGATGTCAGAGCTAATGCTGGGGACACAGG
CAGGCCGATCTATGGCAGAAGGTGATGAGTCGAGTGGATTCACCATTGATGCAGCAAGT
AAGGGTAATGTTGGGAGGTTCATAAACCATAGCTGCTCGCCAAATCTGTACGCCCAAAA
CGTTTTGTATGATCATGAAGACTCTAGAATCCCTCACGTGATGTTCTTCGCACAGGACAA
TATACCTCCGCTTCAAGAACTCTGTTACGACTACAATTACGCGCTCGATCAGGTACGTGA
CTCTAAGGGTAATATCAAGCAGAAACCTTGCTTTTGTGGTGCTGCCGTATGTAGGCGTAG
GCTCTATTGA

>AT2G29130|AT2G29130.1

ATGGTTACGTGGGTTTTGAATTATCTTCTTGTGGCATTTCTATTTGCCATTAGTTACAACAT
CGATGCAGCATCTGCAGGAATCACGCGGCACTACCAATTCGATATTCAACTGAAAAACA
TCACAAGGTTGTGCAAGACAAAAACAATCGTGACGGTCAACGGAAAATTCCCAGGACC
TAGAGTTACCGCAAGAGAAGGAGATAATCTACAAATCAAAGTTGTTAATCATGTATCCAA
TAACATTTCTATACATTGGCATGGTATCCGGCAGCTAAGGAGTGGTTGGGCGGATGGACC
GTCCTATGTGACACAATGTCCAATTCGGATGGGACAAAGTTATGTTTACAACTTCACGGT
GACTGGACAAAGAGGAACCTTGTGGTGGCATGCTCATATTCAGTGGATGAGAGCTACTG
TGTACGGACCATTAATCATTCTCCCTAAGCTTCATCAGCCTTACCCTTTTCCCAAACCCTA
CAAACAAGTCCCTATACTCTTTGGTGAGTGGTTTAATGCTGATCCTCAAGCAGTGGTGCA
ACAAGCTCTTCAGACGGGTGCAGGTCCAAATGCATCTGATGCTCATACTTTTAATGGGCT
CCCAGGTCCATTATACAATTGCTCTACAAAAGACACATACAAACTGATGGTGAAACCTG
GAAAGACGTATCTACTACGATTAATCAATGCTGCACTCAATGACGAACTCTTCTTCACCA
TAGCCAATCATACATTGACGGTCGTAGAAGCGGATGCTTGCTACGTTAAACCATTTCAAA
CCAACATCGTGCTTCTCGGCCCGGGCCAAACCACCAATGTACTACTCAAAACCAAACCT
ATTTATCCAAATGCAACCTTCTACATGTTGGCAAGACCGTACTTCACGGGCCAAGGAAC
AATTGATAATACAACCGTCGCGGGAATTCTCCAATACCAACATCATACCAAGTCTTCTAA
GAATCTTTCTATCATTAAACCATCTCTCCCTCCCATCAATAGCACTAGCTACGCCGCAAAT
TTCACAAAGATGTTTAGAAGTTTGGCAAGTTCTACATTTCCGGCAAACGTGCCAAAAGT
CGTGGACAAACAATATTTCTTTGCCATCGGTTTAGGAACCAACCCTTGTCCTAAAAACCA
AACATGCCAAGGACCAACTAATACAACGAAATTTGCAGCATCTATCAACAACGTGTCTTT
CATCTTACCAAACAAGACTTCTCTATTACAATCATACTTCGTTGGCAAATCCAAGAATGTT
TTCATGACCGATTTCCCAACTGCCCCTATTATTCCATTTAATTACACGGGTACGCCACCAA
ACAACACAATGGTCTCAAGAGGGACCAAAGTTGTGGTCTTGAAGTACAAGACAACTGT

TGAATTGGTGTTACAGGGCACTAGCATCTTGGGTATTGAGGCCCACCCTATACATCTTCAT
GGCTTCAACTTTTATGTGGTGGGTCAAGGCTTTGGCAATTTTAACCCGGCCCGTGACCCA
AAACATTATAACCTAGTCGATCCGGTCGAACGAAACACCATCAATATCCCTTCCGGTGGA
TGGGTCGCCATCCGCTTCCTTGCCGATAATCCCGGTGTGTGGCTGATGCATTGCCACATT
GAGATTCATTTGAGTTGGGGTCTGACCATGGCTTGGGTTGTTTTGGACGGTGACCTTCCA
AATCAGAAGCTCCTTCCTCCACCCTCAGATTTCCCTAAATGTTGA

>AT1G51480|AT1G51480.1

ATGGTGCAGCACTGGGATGGTGACCCAGCTGTGAGAACCAAAAGTCACATAGAGATGTT
GCACCCTGCTTGTGAGATGTCCTGGAACTTGAAGCAACATGACGACACCGCTGCAAGCT
TAATGGTGATCCAAAGTCCAACATCTGATCCAGTTTTCAACCCGGTTCACACCATTAAGC
AATTTGGGGAACGTTACACCCAAGATTTTGTGATACGTGAACGTGATAAGAGTTTTGGTG
TTTTGATTCATTGTTTTTGCAAAATGGCGGATTGGCTTCTACTAATACCCTGGAACAAGAT
CTTCACTGCTGCATGTGGCTGCTTTTTTAGCGATAGAAACTACATTCACAAGATGGAGGC
AAATCTCGATGATCTGCACACAACTATGGAAGAACTTAAGAATGGACGAGATGATTTGTT
AAGAAGAGTTTCCATAGAAGAAGATAAAGGTTTGCAACAGCTTGCTCAAGTCAAAGGA
TGGATTTCAAGGGTAGAAATTGTTGAATCTAGATTCAAGGATCTGCTTGAGGATAAATCA
ACTGAAACTGGAAGATTGTGTTTGTTTGGATTTTGTTCTGAGAATTGCATATCAAGCTAC
AATTATGGTGAAAAGGTAATGAAGAATCTGGAAGAAGTTAAAGAACTTCTATCTAAAAA
ACATTTTGAAGTGGTGGCGCATAAGATTCCTGTACCTAAGGTAGAGGAGAAGAATATCCA
TACCACAGTTGGTTTGTATGCAATGGTTGAAATGGCATGGAAAAGCCTCATGAATGATGA
AATAAGAACCTTGTGTCTTCATGGTATGGGGGGAGTAGGAAAAACCACACTCTTAGCTT
GCATCAACAACAAATTCGTTGAACTGGAGAGTGAATTTGATGTTGTGATATGGGTTGTGG
TATCTAAAGACTTTCAGCTTGAGGGTATTCAAGATCAGATTCTTGGTAGATTACGTCTCG
ACAAGGAATGGGAAAGAGAAACAGAAAATAAGAAAGCCTCACTCATAAACAATAACCT
TAAGAGAAAAAAATTTGTGCTGTTGTTGGATGATCTCTGGAGCGAGGTAGATCTGAACA
AGATAGGAGTTCCACCTCCAACTCGTGAAAATGGAGCGAAGATAGTTTTCACCAAACGT
TCAAAGGAAGTTTCCAAGTACATGAAAGCTGACATGCAAATAAAAGTTTCTTGTTTGTC
ACCAGATGAAGCGTGGGAATTGTTTCGAATTACTGTTGATGATGTCATATTAAGCAGCCA
TGAAGATATTCCTGCACTCGCAAGAATAGTTGCTGCAAAATGTCATGGCTTGCCACTTGC
ACTCATTGTCATTGGGGAAGCCATGGCATGTAAAGAGACTATACAAGAATGGCATCATGC
GATTAATGTTCTGAATTCGCCTGCAGGCCACAAGTTTCCAGGTATGGAAGAAAGAATTCT
TCTCGTTTTGAAGTTCAGTTACGATAGCTTGAAGAATGGTGAAATCAAATTGTGTTTCCT
ATATTGTTCTTTGTTTCCTGAAGATTTTGAAATAGAGAAGGAGAAGTTGATAGAATACTG
GATTTGCGAAGGATATATAAATCCGAACAGATATGAAGATGGAGGTACCAACCAAGGTTA
TGATATAATTGGCTTGTTAGTTCGTGCACATTTGTTGATTGAATGTGAACTCACCACCAAA
GTTAAAATGCATTATGTGATACGTGAGATGGCTCTTTGGATAAATTCTGACTTTGGAAAA
CAGCAAGAAACAATCTGTGTGAAATCTGGTGCTCATGTACGTATGATACCGAATGACATC
AATTGGGAGATTGTAAGACAAGTGTCTTTAATCAGTACTCAGATTGAGAAGATATCTTGT
AGTTCCAAATGCTCCAACCTTTCAACCCTATTACTCCCGTATAACAAGTTGGTGAATATTT
CAGTTGGATTCTTTCTGTTTATGCCAAAACTTGTGGTCTTGGATCTTTCTACAAACATGA
GTCTTATTGAATTACCGGAGGAAATTTCTAACTTATGTTCTCTTCAATATCTTAATTTATCA
AGCACAGGGATAAAATCGCTACCAGGTGGTATGAAGAAGTTGAGGAAACTAATCTACTT
GAATCTGGAGTTCAGTTATAAACTTGAGAGTCTTGTTGGCATATCAGCAACCTTACCAAA
TCTGCAAGTGTTGAAATTATTTTATTCCAATGTTTGTGTTGATGACATATTAATGGAGGAA
CTACAACACATGGATCACTTGAAGATTTTAACGGTAACCATAGATGATGCCATGATTTTG
GAAAGGATACAAGGTATTGACCGATTGGCGAGTAGTATTCGAGGTTTATGTCTAACAAAT
ATGTCAGCACCTCGTGTAGTATTAAGTACGACAGCTCTGGGAGGTCTTCAACAACTTGC
AATTCTGAGCTGCAACATATCTGAGATAAAGATGGATTGGAAAAGCAAAGAAAGAAGG
GAGGTTTCACCAATGGAGATTCATCCATCTACAAGTACAAGTTCTCCAGGCTTCAAGCA
ACTCTCCAGTGTTAATATAATGAAATTGGTAGGGCCAAGGGATTTGTCATGGCTGTTATTT

GCTCAAAATCTAAAGAGTCTACATGTGGGCTTTTCACCAGAAATAGAAGAAATTATAAAT
AAGGAGAAAGGATCGAGTATTACCAAAGAAATTGCCTTTGGAAAATTGGAATCACTTGT
AATATATAAATTGCCTGAACTCAAGGAAATTTGCTGGAATTATCGGACTCTTCCTAACAGT
AGATATTTTGATGTCAAAGATTGTCCAAAGCTACCTGAAGATATTGCTAATTTTCCCATGC
ATGCAGAGGAATAA

>AT1G77850|AT1G77850.1

ATGTCACCGCCGTCGGCAACCGCCGGTGACATCAACCACCGTGAAGTAGACCCGACGAT
CTGGCGCGCTTGTGCTGGAGCCTCCGTCCAGATCCCTGTCCTTCACTCTAGGGTTTACTA
CTTTCCACAAGGTCACGTTGAGCACTGTTGCCCTCTCCTCTCTACTCTTCCTTCCTCCAC
CTCGCCGGTTCCATGTATCATCACTTCAATTCAGTTGCTCGCCGATCCGGTCACCGACGA
GGTCTTTGCTCACCTTATTCTTCAACCGATGACGCAGCAGCAGTTTACTCCGACTAATTAT
TCACGATTCGGCAGATTCGATGGCGATGTTGATGATAACAACAAGGTGACTACCTTCGCC
AAAATTCTCACGCCTTCTGATGCTAACAATGGAGGTGGCTTCTCCGTTCCTCGTTTCTGT
GCTGATTCCGTCTTCCCTCTGCTTAATTTTCAAATCGATCCACCGGTTCAGAAGCTCTAC
GTCACTGATATCCATGGAGCTGTTTGGGATTTCAGGCATATCTATCGCGGTACACCGAGG
CGTCACTTGCTAACAACGGGATGGAGTAAGTTCGTCAATAGCAAGAAGCTCATCGCTGG
AGATTCGGTGGTGTTTATGAGAAAATCTGCAGATGAGATGTTCATCGGCGTTAGGAGAA
CTCCGATCTCAAGCAGCGACGGAGGAAGTAGCTATTACGGAGGAGATGAGTATAACGGT
TACTACAGTCAGAGTAGCGTTGCCAAGGAAGATGATGGGAGTCCGAAGAAGACGTTTA
GGAGATCTGGGAATGGTAAGTTGACTGCTGAGGCTGTGACGGATGCGATCAATAGAGCG
TCTCAGGGATTACCATTTGAGGTGGTGTTTTATCCGGCTGCTGGATGGTCTGAGTTTGTT
GTGAGAGCTGAAGATGTTGAGTCTTCAATGTCTATGTATTGGACTCCTGGGACTCGAGTC
AAGATGGCTATGGAGACTGAAGATTCTTCTCGGATCACATGGTTTCAAGGCATCGTTTCC
TCTACTTATCAGGAGACCGGTCCATGGCGTGGATCTCCATGGAAGCAGCTTCAGATCACA
TGGGATGAACCTGAGATTCTGCAAAACGTGAAGAGGGTGAATCCATGGCAAGTGGAAA
TTGCTGCACATGCAACTCAACTGCATACCCCTTTCCCTCCAGCAAAGAGGTTGAAGTATC
CACAACCCGGAGGAGGGTTCTTGAGTGGAGATGATGGAGAAATCCTTTATCCTCAAAGT
GGACTGTCTAGTGCAGCAGCACCTGATCCAAGTCCTTCTATGTTCTCGTATTCTACATTTC
CTGCTGGCATGCAGGGAGCCAGGCAATATGATTTTGGGTCTTTCAATCCAACCGGATTCA
TTGGAGGAAATCCTCCCCAGCTATTCACCAATAACTTCTTAAGTCCGCTTCCTGATTTGG
GAAAAGTGTCGACTGAGATGATGAACTTTGGCAGTCCGCCATCAGATAACTTATCGCCTA
ATAGCAACACCACTAATCTGTCCTCTGGAAATGACCTGGTTGGAAACCGAGGCCCCCTT
TCAAAGAAAGTTAACTCGATTCAGTTGTTTGGCAAGATCATTACCGTGGAGGAGCATTC
TGAGAGCGGTCCTGCAGAGTCTGGCTTGTGTGAAGAGGATGGCAGCAAAGAGTCCAGC
GACAATGAGACACAGTTGTCCTTATCACATGCTCCTCCAAGCGTGCCTAAACATTCCAAC
AGCAACGCAGGTTCTAGCTCCCAAGGTTAA

>AT2G22840|AT2G22840.1

ATGGATCTTGGAGTTCGTGTTTCTGGTCATGAAACCGTTTCTTCTCCGGGTCAAACTGAA
CTCGGATCTGGTTTCAGTAACAAGCAAGAAAGATCCGGTTTCGATGGTGAAGATTGCTG
GAGAAGTTCAAAGCTCTCACGAACATCAACTGATGGATTCTCTTCTTCCCCTGCCTCTGC
TAAAACGCTGTCGTTTCATCAAGGCATCCCTTTACTGAGATCTACCACTATTAATGATCCT
CGTAAAGGACAAGAACACATGCTTAGCTTCTCTTCTGCTTCAGGCAAATCAGATGTCTC
ACCTTATCTTCAGTACTGTAGAAACTCAGGATATGGTTTAGGAGGAATGATGAACACAAG
CAACATGCATGGAAACTTGTTGACAGGAGTAAAAGGACCTTTTTCATTGACTCAGTGGG
CAGAGCTAGAGCAACAGGCGTTGATCTATAAGTATATCACAGCCAATGTCCCTGTTCCAT
CTAGTTTACTTCTCTCTCTCAAGAAATCTTTTTTCCCTTATGGTTCCTTGCCTCCTAATTCT
TTTGGATGGGGCTCTTTTCATCTGGGCTTTTCCGGTGGTAACATGGATCCCGAGCCAGGG
AGATGTCGCCGGACAGATGGAAAGAAATGGCGGTGCTCGAGGGACGCTGTTCCCGATC

AAAAGTACTGTGAACGACATATTAACAGAGGCCGCCATCGTTCAAGAAAGCCTGTGGAA
GGCCAAAATGGCCACAATACTAATGCTGCCGCCGCTGCTTCTGCTGCTGCCGCTTCTACC
GCTGCTGCTGTGTCCAAAGCGGCAGCGGGGACTTCAGCTGTTGCGATGCGTGGATCAGA
TAATAACAATAGCCTTGCCGCTGCTGTTGGAACACAACATCATACCAATAATCAATCTAC
AGATTCTTTGGCTAACAGAGTTCAAAATTCTCGAGGGGCTTCGGTTTTTCCTGCCACGAT
GAACTTACAGTCGAAGGAAACTCATCCGAAACAAAGCAATAATCCCTTTGAATTCGGAC
TCATCTCTTCTGATTCGTTACTTAATCCGTCGCATAAACAAGCCTCGTATGCAACCTCTTC
CAAAGGCTTTGGATCGTATCTTGACTTCGGCAACCAAGCCAAGCACGCGGGGAATCACA
ACAATGTCGATTCTTGGCCCGAAGAGCTGAAATCGGATTGGACTCAGCTCTCAATGTCA
ATCCCTATGGCTCCATCTTCCCCTGTTCAAGATAAACTTGCACTCTCACCTTTAAGGTTAT
CGCGTGAGTTTGACCCCGCGATCCACATGGGATTAGGCGTCAACACCGAGTTTCTTGAC
CCCGGGAAAAAGACGAATAACTGGATACCAATCTCCTGGGGTAATAACAACTCCATGGG
AGGTCCACTCGGCGAGGTACTAAACAGCACGACCAATAGTCCCAAGTTTGGTTCCTCTC
CAACAGGCGTCTTGCAAAAGTCGACATTTGGTTCTCTTTCTAACAGCAGCTCGGCAAGC
AGCACCATCATTGGCGATAACAACAATAAGAACGGTGATGGAAAAGATCCGCTTGGCCC
GACCACGCTGATGAATACTTCTGCTACTGCTCCTTCTCTGTGA

>AT1G72830|AT1G72830.3

ATGATGCATCAGATGTTGAATAAGAAAGATTCAGCTACTCATTCCACTTTGCCATACCTTA
ATACTAGCATCTCTTGGGGAGTGGTTCCAACTGATTCCGTTGCTAATCGTCGCGGTTCTG
CTGAATCACTAAGCTTGAAGGTTGATTCAAGACCTGGGCATATACAAACTACAAAGCAA
ATCAGTTTTCAGGACCAAGATTCATCTTCAACACAGTCCACTGGTCAATCTTATACTGAA
GTTGCTAGTAGTGGTGATGATAATCCTTCCAGACAAATCTCCTTTTCGGCTAAATCAGGA
TCTGAAATAACTCAACGGAAGGGGTTTGCAAGTAATCCTAAACAAGGCTCGATGACTGG
ATTTCCGAATATTCACTTTGCTCCTGCACAGGCTAATTTCTCATTTCACTATGCTGATCCA
CATTATGGTGGTTTATTAGCTGCAACTTACCTACCACAGGCACCAACATGCAATCCTCAA
ATGCAATACCACGCAATTATGAGGAGGAGACAGCAACGTGCTAAGCTTGAGGCTCAAAA
CAAACTAATCAGAGCCCGTAAGCCCTATCTTCATGAGTCTCGACATGTTCATGCTCTTAA
AAGGCCAAGAGGATCTGGTGGAAGATTCCTAAACACCAAAAAACTTCTTCAAGAATCC
GAACAGGCTGCTGCTAGAGAACAAGAACAGGACAAGTTAGGCCAACAGGTAAACAGA
AAGACCAACATGTCTAGATTCGAAGCTCATATGCTGCAGAACAACAAAGACCGCAGCTC
AACCACTTCTGGCTCAGACATCACCTCTGTTTCCGACGGTGCTGATATCTTTGGACACAC
TGAATTCCAGTTTTCAGGTTTCCCAACTCCGATAAACCGAGCCATGCTTGTTCATGGTCA
GTCTAATGACATGCATGGAGGTGGAGACATGCACCATTTCTCTGTCCATATCTGA

>AT1G53230|AT1G53230.1

ATGGCACCAGATAACGACCATTTCTTAGATTCTCCGTCGCCGCCTCTTCTAGAGATGAGA
CACCACCAATCAGCGACGGAGAACGGTGGTGGTTGCGGCGAGATTGTGGAGGTACAAG
GAGGTCACATTGTTCGGTCAACAGGAAGAAAAGACAGACATAGTAAAGTATGTACAGC
GAAAGGACCACGTGACCGGCGCGTGAGACTCTCAGCTCCGACGGCGATTCAATTCTAC
GATGTTCAAGATAGACTTGGTTTTGATCGACCAAGTAAAGCTGTTGATTGGCTTATTACT
AAAGCTAAATCCGCCATTGATGATCTTGCTCAGCTTCCTCCTTGGAACCCCGCCGATACT
CTTCGTCAACACGCCGCCGCTGCTGCTAACGCTAAACCCAGAAAAACCAAAACTTTAAT
TTCTCCGCCACCGCCACAACCGGAAGAAACAGAGCATCATCGAATCGGAGAAGAAGAA
GATAACGAATCGAGTTTTCTTCCGGCGTCAATGGATTCTGATTCGATAGCTGACACTATA
AAGTCGTTTTTTCCGGTAGCTTCAACGCAACAGAGCTATCATCATCAGCCACCGTCACG
AGGCAATACACAGAACCAAGATCTTCTTCGTCTCTCGCTTCAATCTTTCCAAAATGGTCC
ACCTTTTCCTAATCAAACAGAACCTGCTCTGTTCTCCGGCCAGAGCAATAATCAGTTAGC
GTTTGACTCATCGACGGCAAGCTGGGAACAGAGTCATCAGTCACCGGAATTTGGAAAG
ATACAGAGACTAGTGTCATGGAACAACGTCGGAGCAGCTGAATCCGCCGGAAGTACCG

GAGGATTTGTGTTTGCTTCTCCGTCGTCGTTGCATCCAGTTTATAGCCAAAGTCAGCTTTT
ATCACAGAGGGGTCCCCTTCAGTCCATTAACACACCTATGATTCGTGCTTGGTTTGATCC
TCACCATCATCATCATCATCATCAGCAGTCCATGACCACTGACGATCTCCACCATCATCAT
CCCTACCATATCCCTCCCGGGATTCACCAATCTGCTATTCCAGGCATTGCATTTGCTTCAA
GTGGTGAATTCTCCGGTTTTCGTATACCAGCACGGTTTCAAGGCGAACAAGAGGAGCAC
GGCGGCGACAACAAGCCGTCCTCTGCTTCATCCGATTCTCGCCATTAA

>AT1G66700|AT1G66700.1

ATGACTACTACTCCAGATTGGATCATGATTGGAGGAGACGGTCCTGAGAGTTATAACCAA
CAATCCTCGTATCAGAGAGCATTGTTGGAAGCGACAAAGGACAAGATGACCAAGGCGA
TCTCAGCCAATCTCGACCTAGACTTGATTTCGAATCGCTTCATTGTAGCGGATTTCGGTT
GTGCAAGTGGACCTAACACTTTTGTGGCAGTTCAAAACATAATAGATGCGGTAGAAGAA
AAGTACCGTAGAGAAACCGGACAAAACCCGGCAGATAACATCGAGTTCCAAGTCCTCTT
CAATGATTTCAGCCTCAATGATTTCAACACTCTCTTCCAGACACTTCCACCCGGAAGAA
GATACTTCAGCGCTGGAGTTCCTGGTTCCTTCTTCGAACGTGTTCTTCCTAAGGAGAGTT
TCCACATCGGAGTCATGAGTTACGCGTTCCATTTCACCTCCAAAATCCCCAAAGGGATTA
TGGACCGCGACTCTCCCTTGTGGAACAAAGACATGCAGTGCACGGGGTTCAACCCCGC
TGTCAAGAAAGCGTATCTTGACCAGTACTCTATCGACACCAAAATTCTTTTAGATGCTAG
AGCTGAAGAGCTCGTGCCCGGGGGGTTGATGTTGCTTTTAGGATCGTGTCTAAGAGACG
GAGTTAAGATGTCCGAGACCCCTAAAGGAACTGTAATGGATTTTATTGGAGAATCTCTTA
GCGATCTTGCTAAACAGGGTGTCACCGAGCAAGAGAAGGTGGACACTTTCAGAACCTC
AATTTACTTTGCAGAACAAGGTGAGATAAGGCAAATCATTGAGGAGAATGGGAAGTTCA
CAATCGAGGCTTTTGAAGATATCATTCACGCTAAAAACGAGTTTCCTTTTGACCCCAAGA
CATTGGCCATCTCTTTCAAGGCCTTCTATGGTGCTTTCATTTCCGCACATTTTGGAGTCGA
AGTCATGAGGAAAGCCTTTGAGCTTGTTGAGGTCAAGGCACGCGAACAGATTTCTCGCC
TCCATAACTCCAAACCCGGAATGCAGTACCTCATCGTGCTTCGCAAGAACTAA

>AT2G28350|AT2G28350.1

ATGGAGCAAGAGAAAAGCTTGGATCCACAACTATGGCATGCTTGTGCAGGATCAATGGT
TCAAATCCCTTCACTGAATTCAACGGTTTTTTACTTCGCTCAAGGCCACACAGAGCACG
CTCACGCGCCTCCTGATTTTCACGCGCCGCGCGTTCCACCTCTTATCCTCTGTCGTGTCG
TCTCCGTGAAGTTCCTCGCCGACGCTGAAACAGACGAAGTTTTTGCTAAAATTACGCTT
TTGCCACTTCCGGGAAACGACTTGGATCTAGAAAACGACGCCGTTTTGGGTCTAACTCC
TCCTTCTTCTGACGGTAACGGTAACGGTAAAGAGAAACCGGCGTCTTTCGCTAAAACGT
TAACGCAGTCTGACGCTAATAACGGCGGTGGTTTCTCCGTTCCACGTTATTGCGCCGAGA
CGATTTTCCCGCGGCTTGATTACTCGGCGGAGCCACCGGTTCAAACCGTGATTGCTAAA
GACATCCACGGCGAGACTTGGAAATTCCGGCATATTTACAGAGGAACACCTCGCCGTCA
TCTCCTAACCACCGGTTGGAGCACTTTCGTTAACCAGAAGAAACTAATCGCCGGAGACT
CAATCGTCTTCCTCCGTTCTGAATCCGGTGACCTCTGCGTCGGAATCCGCCGCGCTAAAC
GCGGCGGTCTCGGATCTAACGCAGGATCCGACAATCCTTACCCTGGATTCTCCGGTTTCC
TCCGTGACGACGAGTCAACAACAACAACATCGAAGCTAATGATGATGAAACGCAACGG
AAACAACGACGGAAACGCCGCGGCTACAGGGAGGGTTAGAGTAGAAGCAGTAGCGGA
AGCGGTGGCGCGTGCAGCGTGTGGACAAGCGTTTGAGGTTGTTTATTATCCACGCGCTA
GTACACCGGAGTTTTGCGTAAAAGCAGCTGATGTTAGATCAGCAATGAGGATAAGATGG
TGTAGTGGTATGCGTTTTAAAATGGCGTTTGAAACAGAGGATTCTTCTAGAATCAGTTGG
TTTATGGGTACTGTCTCCGCCGTTCAAGTCGCTGATCCAATTCGTTGGCCTAATTCACCAT
GGCGTCTCCTTCAGGTAGCTTGGGACGAACCGGATTTGTTACAAAACGTTAAGCGGGTT
AGTCCGTGGTTAGTCGAATTGGTATCGAACATGCCTACAATACATTTATCTCCATTCTCTC
CGAGGAAGAAGATTAGGATTCCGCAGCCATTTGAGTTTCCATTCCACGGTACTAAATTCC
CGATTTTCTCCCCGGGATTCGCCAACAATGGCGGTGGCGAGTCCATGTGTTATCTGTCAA

ACGACAACAATAATGCTCCTGCAGGAATACAGGGAGCCAGGCAAGCTCAACAACTCTT
CGGATCACCATCTCCGTCTTTGTTGTCTGATCTCAATCTTAGTAGTTACACCGGTAACAAC
AAGTTACATTCTCCGGCGATGTTTCTATCGAGTTTCAACCCGAGGCATCATCATTATCAGG
CTAGGGATAGTGAGAATAGTAATAACATTTCGTGTTCTTTAACTATGGGGAATCCTGCTAT
GGTTCAGGATAAGAAGAAGTCTGTTGGTTCGGTTAAGACTCATCAGTTCGTGTTGTTCG
GTCAACCGATTTTAACCGAACAGCAAGTTATGAACCGAAAACGGTTTTTGGAAGAAGA
GGCGGAAGCGGAGGAGGAGAAAGGTTTAGTGGCTCGTGGGTTAACATGGAATTATAGTT
TGCAAGGACTTGAGACGGGTCATTGTAAAGTTTTCATGGAATCTGAGGATGTTGGACGC
ACACTCGATCTCTCGGTTATTGGCTCGTACCAAGAATTGTACCGGAAATTGGCTGAGATG
TTTCATATAGAAGAGAGGTCGGATTTGTTGACTCATGTTGTGTACCGGGATGCAAATGGT
GTTATCAAACGTATTGGAGACGAACCTTTCAGTGATTTCATGAAAGCAACTAAACGGCT
AACAATCAAGATGGATATTGGTGGCGACAACGTGAGAAAGACGTGGATAACCGGAATC
AGGACTGGTGAAAATGGTATAGACGCTTCTACGAAGACTGGTCCGCTCAGCATCTTCGC
TTGA

>AT1G66720|AT1G66720.1

ATGTCACCTACTCCAGAATGGGTCATGGTTGGAGGAGAAGGTCCTGAGAGTTACAAGCA
GCATTCTTCGTATCAGAGAGATTTGCTGAAAGCAGCAAAGGATAAAATAAACGCGGTGA
TTTCAACGAACCTCAGCCTCAATTTGATTTCGAATCGGTTCAGTGTTGCGGATTTCGGTT
GTGCAAGTGGACCTAACACTTTTGTCGCAGTCCAAAACATAATAGATGCCGTGGAAGAG
AAGTATCTTAGAGAAACCGGACAAAACCCGGACGATAACATCGAGTTCCAGGTCCTCTT
CAACGACTTAAGCAATAACGATTTCAACACTCTCTTCCAGGGACTTCCTTCTGGCAGGA
GATACTATAGTGCTGCCATTCCTGGTTCCTTCTTTGACCGTGTTCTTCCTAAGCATAGTAT
CCACATAGGAGTCATGAATTATGCTTTTCAATTCACCTCCAAAATCCCCAAAGGGATCTC
AGACCGCAACTCTCCCCTCTGGAACAGAGACATGCATTGCACCGGATTTAACAACAAGG
TCAAGAAAGCGTATCTTGATCAGTTCTCGCTCGACTCCAAGAATATATTGGATGCTCGAG
CTGAAGAGCTTGTGCCCGAGGGATTAATGTTGCTTTTAGGATCGTGTCTAAGAGACGGTA
TCAAGATGTCGGAAACATATAGAGGAATAGTGTTGGACTTAATCGGAGCCTCTTTAAATG
ATCTTGCTCAGCAGGGTGTCATTGAGAAAGACAAGGTGGAGTCTTTCAACATCACACTC
TACATTGCAGAAGAAGGCGAGTTGAGGCAAATCATAGAAGAGAACGGGAAGTTCACAA
TTGAGGCATTCGAGGATATCATTCAGCCAAACGGGGAGTCGCTTGACCCCAAAATCTTG
GCTGTCTCCTTGAAGTCTGCCTTTGGAGGTATCCTCTCCGCACATTTTGGAGCCGAAGCG
ATGATGAAAGCCTTTGAGCTCGTCGAGGCCAAGGCACACCAAGAATTTTCTCGTCTCCA
GAATGCCAAACCCACAATGCAATACCTCATCGTACTTCGCAAGAACTGA

>AT1G56010|AT1G56010.2

ATGGAGACGGAAGAAGAGATGAAGGAAAGTAGTATAAGCATGGTGGAGGCAAAGTTGC
CTCCGGGATTCAGATTTCACCCGAAGGACGATGAGCTTGTCTGCGATTACTTGATGAGAC
GATCGCTTCACAATAATCATCGACCACCTCTTGTCCTGATCCAAGTCGATCTCAACAAGT
GTGAGCCTTGGGACATCCCAAAAATGGCATGCGTGGGAGGGAAGGATTGGTATTTCTAC
AGCCAAAGAGACCGAAAATACGCGACGGGGCTGAGAACTAACCGAGCAACGGCCACC
GGATATTGGAAAGCCACCGGCAAAGACAGAACCATTCTAAGAAAGGGTAAGCTAGTTG
GGATGAGGAAGACATTGGTTTTCTATCAAGGTCGAGCTCCTCGAGGCCGTAAAACCGAT
TGGGTCATGCACGAATTCCGTCTCCAAGGATCTCATCATCCTCCCAATCATTCTCTGAGC
TCTCCAAAGGAAGACTGGGTCTTGTGTAGGGTATTCCATAAGAATACGGAAGGAGTTATA
TGTAGAGACAACATGGGAAGCTGTTTTGATGAGACAGCCTCTGCATCGCTTCCTCCACT
GATGGATCCTTACATCAACTTTGACCAAGAACCCTCTTCTTATCTCAGTGATGATCATCAC
TACATCATCAATGAGCACGTACCCTGCTTCTCCAATTTGTCACAGAACCAAACCTTAAAC
TCGAACCTAACCAACTCAGTCTCTGAACTCAAGATTCCATGCAAGAACCCTAACCCCTT
GTTTACTGGTGGTTCAGCCTCAGCCACGCTCACAGGCCTCGACTCATTCTGTTCTTCAGA

TCAGATGGTTCTCAGAGCTCTACTCAGTCAGCTCACTAAGATTGATGGAAGCCTCGGGC
CTAAAGAATCACAGAGTTATGGAGAAGGTAGCTCGGAGAGCCTCCTGACCGACATCGGT
ATTCCAAGCACTGTTTGGAATTGCTGA

>AT1G53160|AT1G53160.1

ATGGAGGGTAAGAGATCACAAGGACAAGGTTACATGAAAAAGAAGTCTTACCTTGTGG
AAGAAGATATGGAGACTGATACGGATGAAGAAGAGGAAGTAGGTAGGGATAGAGTTAG
AGGGTCTAGAGGTAGCATCAATCGTGGTGGCTCGTTGCGGCTTTGCCAAGTAGATAGAT
GCACAGCTGATATGAAAGAGGCAAAACTGTATCACCGGAGACACAAAGTGTGTGAAGT
TCATGCAAAGGCATCTTCTGTCTTTCTCTCAGGACTTAACCAACGCTTTTGTCAACAATG
CAGTAGGTTTCATGACCTCCAAGAGTTTGATGAAGCTAAGAGAAGTTGCAGGAGGCGCT
TAGCTGGACACAATGAGCGAAGAAGGAAGAGCTCTGGTGAGAGTACTTATGGAGAAGG
ATCAGGTCGGAGAGGAATCAATGGTCAGGTGGTGATGCAGAATCAAGAAAGATCAAGG
GTAGAGATGACACTTCCTATGCCAAACTCATCATTCAAGCGACCACAGATTAGATAG

>AT1G66690|AT1G66690.1

ATGACTACTACTCAAGATTGGATCATGATTGGTGGATATGGCCCTGAAAGTTACAACCAA
CAGTCATCGTATCAGAGAGCTTTGTTGGAAGCGGCAAAGGACAAGATGACCGAGGCGA
TCTCCGCCAACCTCGACCTAGACTTGATTTCGAATCGCTTCATTGTAGCGGATTTCGGTT
GTGCAAGTGGACCTAACACTTTTGTGGCAGTCCAAAACATAATAGATGCCGTGGAAGAG
AAGTATCTTAGAGAAACCGGACAAAACCCGGAGGATAACATCGAGTTCCAAGTCCTCTT
CAACGACTTAAGAATCAATGACTTCAACACTCTCTTCCAGACACTCCCTCCGGGGAGAA
GATACTTTAGCGCCGGGGTTCCTGGTTCCTTCTTCAACCGTGTTCTTCCTAAGCAGAGTT
TCCACATCGCAGTCATGAGTTACGCATTCCTTTTCACCTCCAAAATCCCCAAAGGGATCA
TGGACCGCGACTCTCCGTTGTGGAACAAAGACATGCAGTGCACCGGGTTCAACCCCGC
TGTGAAGAAAGCCTATCTTGAGCAGTACTCTATTGACACCAAAAACCTTTTGGATGCTAG
AGCTGAGGAGCTCATGCCCGGGGGATTGATGTTGCTTTTAGGATCGTGTATGAGAGACG
GAGTTAAGATGTCCGAGACCCTTAAAGGAACAGTAATGGATTTTATTGGAGAATCTCTTA
ATGACCTTGCTCAAAAGGGTGTCACCGAACAGGAAAAGGTGGACACTTTCAAAACCTC
AATCTACTTTGCAGAACAAGGCGAGATAAGGCAAATCATTGAGGAGAATGGGAAGTTCA
CAATCGAGGCTTTCGAGGATATCATTCACTCTAAGAATGAGTTTCCGCTAGACCCCAAGA
CATTGGCCATCTCCTTCAAGGCCTTATATGGTGCTTTTATATCCGCACATTTTGGAATCGA
AGTCATGAGGAAAGCCTTTGAGCTTGTTGAGGTCAAGGCACGCGAACAGATTTCTCGCC
TCCATAAGGTCAAACCCGGGATGCAATACCTCATCGTGCTTCGCAAGAACTGA

>AT1G52150|AT1G52150.2

ATGGCAATGTCTTGCAAGGATGGTAAGTTGGGATGTTTGGATAATGGGAAGTATGTGAGG
TATACACCTGAACAAGTTGAAGCACTTGAGAGGCTTTATCATGACTGTCCTAAACCGAG
TTCTATTCGCCGTCAGCAGTTGATCAGAGAGTGTCCTATTCTCTCTAACATTGAGCCTAA
ACAGATCAAAGTGTGGTTTCAGAACCGAAGATGTAGAGAGAAACAAAGGAAAGAGGC
TTCACGGCTTCAAGCTGTGAATCGGAAGTTGACGGCAATGAACAAGCTCTTGATGGAGG
AGAATGACAGGTTGCAGAAGCAAGTGTCACAGCTGGTCCATGAAAACAGCTACTTCCG
TCAACATACTCCAAATCCTTCACTCCCAGCTAAAGACACAAGCTGTGAATCGGTGGTGA
CGAGTGGTCAGCACCAATTGGCATCTCAAAATCCTCAGAGAGATGCTAGTCCTGCAGGA
CTTTTGTCCATTGCAGAAGAAACTTTAGCAGAGTTTCTTTCAAAGGCAACTGGAACCGC
TGTTGAGTGGGTTCAGATGCCTGGAATGAAGCCTGGTCCGGATTCCATTGGAATCATCGC
TATTTCTCATGGTTGCACTGGTGTGGCAGCACGCGCCTGTGGCCTAGTGGGTCTTGAGCC
TACAAGGGTTGCAGAGATTGTCAAGGATCGTCCTTCGTGGTTCCGCGAATGTCGAGCTG
TTGAAGTTATGAACGTGTTGCCAACTGCCAATGGTGGAACCGTTGAGCTGCTTTATATGC

AGCTCTATGCACCAACTACATTGGCCCCACCACGCGATTTCTGGCTGTTACGTTACACCT
CTGTTTTAGAAGATGGCAGCCTTGTGGTGTGCGAGAGATCTCTTAAGAGCACTCAAAAT
GGTCCTAGTATGCCACTGGTTCAGAATTTTGTGAGAGCAGAGATGCTTTCCAGTGGGTAC
TTGATACGGCCTTGTGATGGTGGTGGCTCAATCATACACATAGTGGATCATATGGATTTGG
AGGCTTGTAGCGTGCCTGAGGTCTTGCGCCCGCTCTATGAGTCACCCAAAGTACTTGCA
CAGAAGACAACAATGGCGGCACTGCGTCAGCTCAAGCAAATAGCTCAGGAGGTTACTC
AGACTAATAGTAGTGTTAATGGGTGGGGACGGCGTCCTGCTGCCTTAAGAGCTCTCAGC
CAGAGGCTAAGCAGAGGCTTCAATGAAGCTGTAAATGGTTTCACTGATGAAGGATGGTC
AGTGATAGGAGATAGCATGGATGATGTCACAATCACTGTAAACTCTTCTCCAGACAAGCT
AATGGGTCTAAATCTTACATTTGCCAATGGCTTTGCTCCTGTAAGCAATGTTGTTTTATGC
GCAAAAGCATCAATGCTTTTACAGAATGTTCCTCCGGCGATCCTGCTTCGGTTTCTGAGG
GAGCATAGGTCAGAATGGGCTGACAACAACATTGATGCGTATCTAGCAGCAGCAGTTAA
AGTAGGGCCTTGTAGTGCCCGAGTTGGAGGATTTGGAGGGCAGGTTATACTTCCACTTG
CTCATACTATTGAGCATGAAGAGTTTATGGAAGTCATCAAATTGGAAGGTCTTGGTCATT
CCCCTGAAGATGCAATCGTTCCAAGAGATATCTTCCTTCTTCAACTTTGTAGCGGAATGG
ATGAAAATGCTGTAGGAACCTGTGCGGAACTTATATTTGCTCCAATCGATGCTTCGTTTG
CGGATGATGCACCTCTGCTTCCTTCTGGTTTTCGTATTATCCCTCTTGATTCCGCAAAGCA
GGAAGTATCTAGCCCAAACCGAACCTTGGATCTTGCTTCGGCACTGGAAATTGGTTCAG
CTGGAACAAAAGCCTCAACTGATCAATCAGGAAACTCCACATGTGCAAGATCTGTGATG
ACAATAGCATTTGAGTTTGGTATCGAGAGCCATATGCAAGAACATGTAGCATCCATGGCT
AGGCAGTATGTTCGAGGTATCATATCATCGGTGCAGAGAGTAGCATTGGCTCTTTCTCCT
TCTCATATCAGCTCACAAGTTGGTCTACGCACTCCTTTGGGTACTCCTGAAGCCCAAACA
CTTGCTCGTTGGATTTGCCAGAGTTACAGGGGCTACATGGGTGTTGAGCTACTTAAATCA
AACAGTGACGGCAATGAATCTATTCTTAAGAATCTTTGGCATCACACTGATGCTATAATCT
GCTGCTCAATGAAGGCCTTGCCCGTCTTCACATTTGCAAACCAGGCGGGACTTGACATG
CTGGAGACTACATTAGTTGCTCTTCAAGACATCTCTTTAGAGAAGATATTTGATGACAAT
GGAAGAAAGACTCTTTGCTCTGAGTTCCCACAGATCATGCAACAGGGCTTCGCGTGCCT
TCAAGGCGGGATATGTCTCTCAAGCATGGGGAGACCAGTTTCGTATGAGAGAGCAGTTG
CTTGGAAAGTACTCAATGAAGAAGAAAATGCTCATTGCATCTGCTTTGTGTTCATCAATT
GGTCCTTTGTGTGA

>AT2G02850|AT2G02850.1

ATGGCCAAGGGAAGAGGCAGTGCATCATGGTCGGCTCGAGCCATAGTGACTCTCATGGC
TGTGTCAGTGTTGTTGCTTCAAGCTGACTACGTTCAAGCTGCAACGTACACGGTCGGTG
ACTCTGGTATCTGGACCTTTAACGCTGTGGGTTGGCCTAAAGGCAAACACTTTAGAGCC
GGCGACGTTCTTGTGTTTAACTATAATCCGAGGATGCACAACGTAGTGAAGGTAGATAGC
GGAAGTTACAACAACTGCAAAACACCGACAGGGGCAAAACCTTACACTTCAGGCAAAG
ATCGTATAACTTTGTCTAAAGGACAAAACTTTTTCATCTGCAATTTTCCAAACCATTGCG
AAAGCGATATGAAAATCGCAGTCACCGCGGTTTGA

>AT1G53290|AT1G53290.1

ATGCATTCTCCTCGTAAGCTATTTCACGCGCGGTCATCACTCGCCACGCGCCGATCAACG
GCTCTTGTCGTATTAACTTCCCTAGCTATTGGAATCGCCGGTTTCACATTTGGACTCGCCG
TAATTCTTATACCGGGTCTCCGATTAACCGGTCGTAATTGCTTGACGAACACTCCTCCGA
AGACGGTGCGAGTCGTTTGGGACGTCGCCGGAAATAGTAATGGCGTTGTTAGTGGCGAA
AAGAAGAGGCATAAGGTTATGGGATTCGTTGGTATTCAAACCGGATTTGGATCTGCTGGC
CGGAGACGATCACTTAGAAAGACATGGATGCCGTCAGATCCAGAAGGTCTTCGACGTTT
GGAAGAATCTACGGGGTTAGCCATCAGGTTTATGATAGGAAAAACGAAAAGTGAGGAG
AAAATGGCTCAGCTCAGAAGAGAAATCGCAGAGTATGATGACTTCGTACTGCTAGATAT
CGAAGAGGAGTACAGTAAGCTTCCTTACAAAACTTTGGCTTTCTTCAAAGCTGCGTACG

CGCTTTATGATTCTGAGTTCTATGTCAAAGCTGATGATGATATATACTTGAGGCCAGATCG
ACTCTCTCTGCTATTGGCGAAAGAGCGGAGTCACTCTCAGACGTACCTAGGATGCTTGA
AAAAGGGTCCGGTTTTCACAGATCCTAAGCTCAAATGGTATGAACCATTGTCTCATCTGC
TGGGAAAAGAGTACTTTCTTCATGCTTATGGCCCGATTTATGCTCTCTCTGCTGATGTGGT
AGCCAGTTTGGTTGCCCTCAAGAATAACAGTTTCAGGATGTTTAACAACGAGGACGTAA
CAATAGGTGCGTGGATGCTGGCAATGAATGTCAACCACGAGAACCATCACATCCTTTGC
GAACCAGAATGCTCACCTTCTTCTGTTGCTGTTTGGGACATCCCTAAATGCTCAGGTCTT
TGTAACCCAGAGAAAAGGATGTTGGAACTTCACAAGCAAGAAAGCTGCTCGAAAAGCC
CAACTTTGCCATCAGATGATGAATGA

>AT2G31070|AT2G31070.1

ATGGGACTTAAAGGATATAGCGTCGGAGAAGGAGGAGGAGAGATAGTTGAGGTTCAAG
GCGGTCACATAATCCGAGCCACTGGACGCAAAGACCGTCATAGCAAAGTGTTCACATCA
AAGGGTCCACGTGACCGGCGCGTGAGACTCTCAGCTCACACGGCGATTCAATTCTACGA
TGTGCAAGACCGGTTAGGTTATGACCGGCCTAGCAAAGCCGTTGATTGGCTCATCAAGA
AAGCCAAAACCGCTATTGATAAGCTTGAACTCGGGGAGACTACTACTACTACTACTCGTC
AAGAACCGGTGAATACTAAACCGGAATCTCCCACTTTGGTTTTCCAAAGAGAGAACAAT
GACCAAACGCAGTTCGTGGCGGCCAATCTTGATCCTGAAGACGCAATGAAAACGTTTTT
CCCGGCGACGACAACAACAAACGGTGGTGGTGGTACAAACATCAATTTCCAAAACTAC
CCTCATCAGGATGACAACAACATGGTCTCAAGAACAACAACACCACCACCAAACCTTA
GCCAAGATCTTGGTCTCTCTCTTCACCCATTTCAAGGGAACAACAACACAGTCGTAGTT
CCCGAGACCAACAATTTCACCACGACTCATTTCGACACGTTCGGGAGAATCTCTGGATG
GAATCATCACGACTTAACTATGACGTCATCATCCTCATCAGAACATCAACAACAAGAACA
AGAAGAAAGGAGTAATGGTGGTTTCATGGTGAATCATCATCCTCACCATCATCATCATCA
ACCGTCGATGATGACATTGCTTAATAGTCAGCAACAACAAGTGTTTCTTGGTGGCCAAC
AACAACAACAACAAAGGGGTACCCTTCAGTCCAGTTTATTCCCTCATTCGTTTCGTTCTT
GGGATCATCATCAAACGACGTCGGATCATCATCATCATCAGAATCAAGCTTCTTCGATGT
TTGCTTCATCATCACAGTATGGTTCTCATGGGATGATGATGATGCAAGGCCTTAGTTTCCC
CAACACTACAAGACTACTCCACGGAGAAGAAGCTACTCAACCAAACTCTTCTTCTTCTC
CTCCAAACTCACACCTCTAA

>AT1G69770|AT1G69770.1

ATGGCGCCGAAGCGAAAGAGACCTGCGACAAAGGATGACACTACCAAATCCATTCCCA
AACCGAAGAAGAGAGCTCCTAAGCGAGCTAAGACGGTGAAAGAAGAGCCGGTGACAG
TGGTCGAGGAAGGGGAAAAGCATGTTGCGAGGTTTCTAGACGAGCCAATTCCAGAATC
TGAAGCGAAGAGTACCTGGCCTGACAGATACAAACCGATTGAGGTACAGCCACCTAAG
GCTTCGTCAAGAAAGAAGACGAAGGATGACGAAAAAGTTGAGATCATTCGTGCTCGAT
GCCATTATAGACGTGCGATTGTTGATGAGCGTCAGATATATGAGCTGAATGATGATGCTTA
TGTACAGTCTGGTGAGGGAAAGGATCCCTTCATTTGTAAAATCATTGAAATGTTTGAAGG
GGCTAATGGGAAACTGTATTTCACGGCTCGGTGGTTTTATAGACCTTCTGATACTGTAATG
AAAGAGTTCGAGATTCTGATCAAGAAAAAGCGTGTGTTTTTCTCTGAGATACAAGATAC
AAATGAATTGGGATTACTTGAAAAGAAGCTGAACATTTTGATGATTCCCTTGAATGAAAA
TACTAAAGAGACTATCCCTGCAACAGAAAACTGTGACTTTTTCTGTGACATGAACTATTT
CTTGCCTTACGATACATTTGAAGCTATACAACAAGAAACCATGATGGCTATAAGTGAAAG
TTCAACAATATCCAGTGATACTGATATAAGAGAAGGAGCTGCTGCCATATCAGAGATTGG
AGAATGTTCTCAAGAAACAGAAGGTCACAAAAAGGCAACTTTGCTTGACCTTTACTCCG
GCTGTGGAGCTATGTCGACAGGGTTGTGCATGGGTGCACAACTGTCTGGTTTGAACCTC
GTCACTAAATGGGCTGTTGACATGAATGCACATGCATGTAAAAGCTTGCAGCATAACCAC
CCAGAGACAAACGTGAGAAACATGACCGCAGAAGATTTCTTGTTTCTGCTTAAGGAGTG
GGAGAAGCTATGCATTCATTTCTCTTTGAGAAATAGTCCAAATTCAGAAGAATATGCCAA

CCTTCACGGTTTGAATAATGTTGAGGACAATGAAGATGTCAGCGAGGAGAGTGAAAATG
AAGATGATGGAGAAGTTTTTACTGTTGACAAGATTGTTGGTATTTCCTTCGGAGTCCCTA
AAAAGTTATTGAAACGTGGACTTTATTTGAAGGTAAGGTGGCTGAATTATGATGATTCTC
ATGATACATGGGAGCCTATTGAAGGACTCAGTAATTGCCGGGGTAAAATTGAAGAGTTC
GTTAAACTTGGATATAAATCTGGCATCCTTCCGTTACCAGGAGGTGTTGATGTTGTCTGC
GGTGGGCCACCATGCCAAGGAATCAGTGGTCACAACCGCTTCAGGAACTTATTGGACCC
TCTAGAAGATCAGAAAAACAAGCAGCTTTTGGTGTATATGAACATTGTAGAATATTTGAA
GCCTAAGTTCGTTTTGATGGAAAACGTCGTTGACATGCTGAAGATGGCTAAGGGCTATCT
TGCACGGTTTGCTGTTGGACGCCTTCTACAGATGAATTACCAAGTGAGGAATGGAATGA
TGGCAGCTGGAGCTTATGGGCTTGCTCAGTTTCGTTTGAGGTTCTTTCTATGGGGTGCAC
TCCCTAGTGAGATAATTCCGCAGTTCCCACTTCCAACACATGATCTAGTTCATAGAGGAA
ATATTGTCAAGGAGTTTCAGGGAAACATAGTAGCCTATGATGAAGGACATACTGTGAAGT
TAGCAGACAAGCTTTTGTTGAAGGATGTGATTTCTGATCTTCCTGCAGTTGCCAACAGTG
AAAAAAGAGACGAGATTACATATGACAAAGATCCCACAACGCCATTTCAAAAGTTCATC
AGATTGAGAAAGGATGAAGCGTCAGGTTCACAATCAAAGTCCAAGTCCAAAAAGCATG
TCTTATATGATCATCACCCTCTTAATCTTAATATAAATGACTATGAACGGGTTTGTCAGGTC
CCCAAGAGAAAGGGAGCGAATTTTAGGGACTTTCCTGGTGTTATTGTTGGACCTGGTAA
TGTAGTCAAGTTGGAAGAGGGAAAGGAAAGGGTCAAACTTGAATCTGGAAAAACATTG
GTTCCCGATTATGCCTTAACATATGTCGATGGGAAATCATGCAAACCTTTTGGTCGTCTTT
GGTGGGACGAAATTGTCCCCACTGTTGTCACACGGGCAGAACCCCACAACCAGGTGAT
CATTCATCCAGAGCAAAATCGGGTTTTATCCATTCGAGAAAATGCGAGACTCCAAGGCTT
TCCTGATGACTACAAACTCTTTGGCCCACCCAAACAGAAGTACATTCAAGTAGGTAACG
CTGTAGCTGTGCCAGTAGCGAAGGCCCTTGGATATGCTTTGGGAACAGCTTTCCAGGGA
CTCGCAGTTGGGAAAGATCCACTTCTTACTCTGCCTGAAGGTTTTGCATTCATGAAGCCA
ACTCTTCCTTCCGAGCTTGCATGA

>AT2G28550|AT2G28550.2

ATGTTGGATCTTAACCTCAACGCTGATTCTCCCGAGTCGACTCAGTACGGTGGTGACTCA
TACTTAGATCGGCAGACATCAGACAACTCCGCCGGGAATCGAGTGGAAGAGTCCGGTAC
ATCGACGTCGTCAGTTATCAATGCCGATGGAGACGAAGACTCTTGCTCTACTCGAGCTTT
CACTCTCAGTTTCGATATTTTAAAAGTCGGAAGTAGTAGCGGCGGAGACGAAAGCCCCG
CCGCTTCAGCTTCCGTTACTAAAGAGTTTTTTCCGGTGAGTGGAGACTGTGGACATCTAC
GAGATGTTGAAGGATCATCAAGCTCTAGAAACTGGATAGATCTTTCTTTTGACCGTATTG
GTGACGGAGAAACGAAATTGGTAACTCCGGTTCCGACTCCGGCTCCGGTTCCGGCTCAG
GTTAAAAAGAGTCGGAGAGGACCAAGGTCTAGAAGTTCACAGTATAGAGGAGTTACTTT
TTATAGAAGAACTGGTCGATGGGAGTCACATATTTGGGATTGTGGAAACAAGTTTATTT
AGGTGGTTTCGACACTGCTCATGCTGCAGCTAGAGCTTATGATCGAGCTGCTATTAAATT
TAGAGGTGTTGATGCTGATATCAACTTTACTCTTGGTGATTATGAGGAAGATATGAAACA
GGTACAAAACTTGAGTAAGGAAGAGTTTGTGCATATACTGCGTAGACAGAGCACGGGGT
TTTCGCGGGGGAGTTCGAAGTATCGAGGGGTTACGTTACACAAATGTGGTAGATGGGAA
GCTAGGATGGGGCAGTTTCTTGGTAAAAAGGCTTATGACAAGGCTGCAATCAACACTAA
TGGTAGAGAAGCAGTCACGAACTTCGAGATGAGTTCATACCAAAATGAGATTAACTCTG
AGAGCAATAACTCTGAGATTGACCTCAACTTGGGAATCTCTTTATCGACCGGTAATGCGC
CAAAGCAAAATGGGAGGCTCTTTCACTTCCCTTCTAATACTTATGAAACTCAGCGTGGA
GTTAGCTTGAGGATAGATAACGAATACATGGGAAAGCCGGTGAATACACCTCTTCCTTAT
GGATCCTCGGATCATCGCCTTTACTGGAACGGAGCATGCCCGAGTTATAATAATCCCGCC
GAGGTAAAAACATAG

>AT3G15640|AT3G15640.2

ATGTGGAGAAGAATCGTCTCCTCTCAGCTCAAAACCCTAGCCGCCGATGTCGTCGCTGC

TTCTCCTCGTCGATCGATAGCCGCCACCACCAGACCTGTCGGTTTCTATCTCGCCGCCAA
TCGATCAGCCATTTCCGCATCTTCTTTCGTTATCCCTCGTCGTTTCAGCTCCGATTCAGAG
ACTCCTGCGACGAAGAAGGTGGAGGATGTAATGCCCATTGCGACTGGACACGAGAAGG
AAGAGCTTGAAGCTGAACTTGAGGGAAGGAGGCTGGATGATATTGACTTCCCTGAAGG
TCCTTTTGGAACTAAGGAAGCTCCTGCTATTGTGAAGTCCTACTATGACAAGCGAATTGT
GGGCTGCCCTGGTGGTGAAGGCGAGGACGAGCACGATGTTGTCTGGTTCTGGCTGGAG
AAAGGAAAGTCCTTTGAATGCCCGGTTTGCACTCAGTACTTTGAGCTGGAAGTGGTTGG
TCCTGGTGGTCCTCCGGATGGTCACGGTGACGAAGACGATGAGCACCACCACTGA

>AT3G49510|AT3G49510.1

ATGACGACAATTTCCGATCTATCAGATGATTTGGTAGGTGATATTCTTTCTAGGGTTCCAT
TTACATCTCTAATCTCAGTGCGATCTACTTGTAAAAAGTGGAATGCTTTATCGAAAAATCA
GATCTTTGGTAGAAAAACAGCAGCAAGGAATCAATTTCTAGAGTTCATGATTCTAGATTC
TAGAGTTTGTTCTTTGAGATTAGATCTTCAAGGAATCCGTAACGAAGACAAAGAAGACT
TCGTTGATCCATCTATGAAGCTAATAAGTATACCTAGTAATGATGATCAAGTCGAGATATC
TCAAGTCTATCATTGCGATGGCTTATTGTTATGCATCGCCAAAGAGAACTCGAATGTCTTT
GTGTGGAATCCGTATTTGGGGCAAACGAAATGGATTCGACCAAGAAACACATTCCACAG
ATATGACAGGTTTGCTCTTGGATACGACAACAACCGTAACCACAAAATCTTGAGGTTTCT
CTATGATGAAGAGAGCAACGAAAGCAGTCGAAGAACTCACATTGATGTATACGATTTTA
GCTCTGATTCATGGAGGGTTCTTGATGTCAATCCTGACTGTGATATACCGTTTTATCAGAC
CGGAGTGTCTTTGAAGGGAAATACTTACTTCTTCGGTCAAGAAGTGACACAAGCGACG
AAAGTAACAGAGATTGAAACTTGTTTACTCTGTTTTGATTTTACAACAGAGAGATTTGGA
CCGTGTTTGCCTCTGCTGTTTTACCCTCCCTGTCCTAGTTTTGAAACTGTGACTCTATCTT
GGGTTAGAGATGAGAAGCTCGCTGTGTTATACAACCACTACGTCACAGCTGAGATTATAG
AGATTAGGATTTCGACTAAGATTGAACCCAATGCGGTATCCTGGAGCTCTTTTTTGACTG
TGGATATGTCACTAGTCAATGGTTTACCGGATCATTTTTCGATGTACTTTGAAGCTAAGAG
CTTCTTCATTGACGAGGAGAAGAAAGTTGTTGTGCTTTTTGATAGTAAAGAGATCAAGA
CCTGTCGCTACCAAATGGCTTACATCGTTGGAGATGATGGATACTTCAAATCTGTCAACA
TTGGAGAAAAAGTAGGAGATAGAGAAAGAAACGGTTCTCAAATGAAAACCTCTCTTTTA
TGCATCGCCGAAGACAACTCGGGACTTGTGGTATGGAATCCGTATTTGTGTCAAACCAA
ATGGATTCGACTTAGAAACACATTCCACAGATATGACGTGTTTGCTCTTGGACACGACAA
CAACCGTAACCACAAAATCTTGAGGTTTCTTTATGATGCCAAGAAGTACAGAACCGGTC
GCGGACGCGACAATGATGTCTACGATTTTAGCTCTGATTCATGGAGGAGGGTTTTTGATG
TTAATCCCCACAGGGGTGAACTGTTTTATTACAGTGGCGTGTCCTTGAAGGGAAATTCAT
ACTTTTTCGGTCAAGAGGTAACAATAGAGGAAGAAGAAGAAGAAGTGATATCACAACC
GAGAGATTATTTACTATGTTTTGATTTTACAACCGAGAGATTTGGACCGCGTTTGTCTCTG
CCGTTTAACCCTCCCTCTCCTAATTTTGACTATTTGACTCTATCATGGGCTAGAGATGAGA
AGCTGGCTGTGTTATATAACCACTACAACACACGTGAGATTATAGAGATTTGGATTTCGA
CTAAGATTGAACCCAACACGGTATCGTGGAGCACTTTTTTGACAGTGGATACGTCACTAA
TCAATGGTTTACCGGATCGTTTTTCAACTTTCTATGGACCTAGGAGCTTCTTCATTGATGA
GGAGAAGAAAGTCGCTGTGTTTTTTGATAATAAAGGGACCGAGACCGGTTGTTACCAAG
TGGCTTACATCATTGGAGATAATGGATACTTCAAATCTGTCAAAATTGGAGTAATCTCGA
ACTCCCAGTGGCAACAAGGCATACTTGTGTGCTCTTCTTATGTTCCAAGTTTAGTGCAAC
TACAAGATTAG

>AT4G37770|AT4G37770.1

ATGGGTCTCTTGTCAAAGAAAGCTAGTTGCAACACGCACGGCCAAGATTCTTCGTATTTT
TGGGGTTGGGAAGAGTATGAAAAAAATCCTTACGACGAGATCAAGAACCCAGACGGCA
TTATCCAAATGGGTCTAGCAGAAAATCAGTTGTCTTTCGATCTCATTGAGTCATGGCTTG
CTAAGAACCCCGACGCAGCCAATTTCCAAAGAGAAGGCCAATCCATATTTCGGGAATTA

GCTCTCTTTCAAGATTATCATGGCCTTCCTTCCTTCAAGAATGCTATGGCGGATTTCATGT
CGGAAAATAGAGGAAATCGAGTTTCTTTCAATCCAAACAAGCTTGTCCTCACCGCTGGT
GCTACTCCGGCTAACGAGACTCTCATGTTTTGTCTCGCTGATCCTGGAGATGCTTTCTTG
CTCCCTACGCCGTATTATCCAGGATTTGATAGGGATTTGAAATGGAGAACCGGAGCTGAG
ATTGTACCGATCCAGTGTAAGAGTGCAAACGGTTTCCGCATCACAAAAGTAGCACTTGA
AGAAGCCTACGAGCAAGCTCAAAAGCTTAACCTAAAAGTTAAAGGAGTCCTTATAACCA
ACCCATCTAACCCGTTGGGCACTACAACGACACGAACCGAACTAAACCATCTCTTGGAC
TTCATCTCACGTAAGAAGATACATTTGATAAGCGACGAGATCTATTCGGGTACCGTTTTC
ACCAATCCCGGATTCATTAGCGTAATGGAAGTCCTCAAAGACAGAAAGCTCGAAAACAC
CGATGTTTTCGACCGTGTCCACATTGTTTACAGTTTGTCTAAAGATCTAGGCCTACCTGG
TTTTCGCGTTGGGGTGATTTACTCCAACGATGATTTTGTTGTCTCCGCAGCGACAAAAAT
GTCCAGTTTCGGTCTAATCTCTTCTCAAACACAATACCTCTTGTCCGCATTGTTATCAGAC
AAGACCTTCACCAAAAACTACCTCGAAGAAAACCAAATCCGGCTCAAGAACAGACACA
AGAAGCTCGTCTCGGGTCTAGAGGCTGCAGGCATCGAGTGTCTCAAGAGCAACGCCGG
ACTCTTCTGTTGGGTTGACATGAGACACCTATTAAAATCAAACACGTTCGAAGCCGAGA
TTGAGCTATGGAAAAAGATCGTTTACGAGGTTAAGCTCAATATCTCTCCCGGTTCTTCGT
GCCATTGCAACGAACCGGGTTGGTTTAGGGTTTGTTTTGCGAATTTGAGCGAAGAGACA
TTAAAGGTAGCGTTGGATAGATTGAAGAGGTTCGTTGATGGACCGTCGCCTACTAGAAG
AAGTCAAAGTGAACATCAAAGACTAAAGAATCTAAGGAAGATGAAAGTCTCTAATTGG
GTTTTCCGGCTATCGTTTCACGACCGTGAACCCGAGGAACGATAG

>AT5G53950|AT5G53950.1

ATGGACATTCCGTATTACCACTACGACCATGGCGGAGACAGCCAATATCTTCCACCGGGT
TTCAGGTTTCATCCCACGGACGAAGAGCTCATCACTCATTACCTTCTCCGCAAAGTCCTC
GACGGTTGCTTCTCAAGCCGTGCCATCGCAGAAGTTGATCTCAACAAGTGTGAGCCTTG
GCAACTTCCCGGGAGAGCTAAGATGGGAGAGAAAGAATGGTACTTCTTTAGCCTCCGTG
ACCGGAAGTATCCGACGGGACTGAGAACTAACAGAGCAACTGAGGCTGGTTACTGGAA
AGCTACCGGAAAAGACAGAGAGATCTTTAGTTCAAAGACTTGTGCACTTGTTGGGATGA
AGAAGACTCTTGTCTTTTACAAAGGAAGAGCTCCGAAAGGAGAGAAGAGTAATTGGGT
TATGCATGAATATCGTCTTGAAGGCAAATTCTCTTACCATTTCATCTCAAGAAGCTCCAAG
GATGAATGGGTGATCTCTAGGGTTTTCCAGAAAACCACTTTAGCTAGCACCGGAGCCGT
CTCCGAAGGAGGAGGAGGAGGAGGAGCAACTGTGAGCGTAAGCAGCGGTACTGGTCC
ATCTAAAAAGACGAAAGTACCCTCAACAATCTCAAGAAACTATCAAGAACAACCAAGC
TCTCCTTCCTCCGTCTCACTCCCACCTCTCCTGGATCCGACCACTACCCTCGGCTACACC
GACAGCAGTTGCTCCTACGACAGCCGTAGCACCAACACAACCGTCACAGCCAGCGCAA
TAACCGAGCACGTGTCCTGTTTCTCCACTGTCCCTACTACTACTACGGCCTTGGGCTTAG
ACGTTAACTCATTCAGCCGTCTTCCACCGCCGCTAGGGTTTGACTTTGACCCTTTTCCTC
GTTTCGTTTCTAGAAACGTCTCGACTCAATCTAACTTCAGATCGTTCCAAGAAAACTTCA
ATCAATTTCCTTACTTTGGATCGTCTTCTGCATCGACTATGACCTCCGCCGTTAATCTGCC
TTCTTTCCAAGGCGGCGGAGGCGTCTCCGGGATGAATTACTGGCTACCGGCGACTGCCG
AAGAGAATGAGTCAAAGGTCGGTGTGCTTCATGCTGGACTTGACTGTATTTGGAACTAC
TGA

>AT2G33810|AT2G33810.1

ATGAGTATGAGAAGAAGCAAAGCGGAAGGGAAGAGGAGCTTACGAGAACTGAGTGAG
GAAGAGGAAGAAGAAGAAGAAACTGAAGATGAAGATACTTTTGAAGAAGAAGAGGCT
TTGGAGAAGAAGCAGAAAGGTAAAGCTACAAGTAGTAGTGGAGTTTGTCAGGTCGAGA
GTTGTACCGCGGATATGAGCAAAGCCAAACAGTACCACAAACGACACAAAGTCTGCCA
GTTTCATGCCAAAGCTCCTCATGTTCGGATCTCTGGTCTTCACCAACGTTTCTGCCAACA
ATGCAGCAGGTTTCACGCGCTCAGTGAGTTTGATGAAGCCAAGCGGAGTTGCAGGAGA

CGCTTAGCTGGACACAACGAGAGAAGGCGGAAAAGCACAACTGACTAA

>AT3G08500|AT3G08500.1

ATGATGATGAGGAAACCGGACATTACTACGATCAGAGACAAAGGCAAGCCAAATCATGC
ATGTGGTGGTAATAACAACAAACCGAAGCTAAGAAAAGGACTTTGGTCGCCTGATGAA
GATGAGAAGCTGATAAGATACATGTTGACTAATGGACAAGGATGTTGGAGTGACATCGC
TAGAAATGCTGGTCTTTTACGTTGTGGTAAAAGTTGTCGCCTTCGCTGGATCAATTACTT
GAGGCCTGATCTTAAACGTGGATCCTTCTCTCCTCAGGAGGAGGATCTCATCTTCCATTT
GCATTCCATTCTTGGTAACAGGTGGTCTCAAATAGCTACTCGGCTTCCAGGTAGAACAGA
CAACGAGATCAAAAACTTTTGGAACTCGACATTGAAGAAGCGGCTTAAGAACAACAGC
AACAACAATACTTCATCAGGATCATCACCTAACAATAGTAATAGTAATTCCTTGGACCCA
AGAGATCAACATGTGGATATGGGAGGCAACTCAACTTCATTGATGGATGACTATCATCAT
GATGAAAACATGATGACAGTGGGGAACACCATGCGCATGGACTCTTCCTCCCCATTCAA
TGTTGGACCAATGGTTAATAGTGTGGGCTTAAACCAACTTTATGATCCCTTGATGATATCA
GTGCCGGATAACGGATATCACCAAATGGGAAACACAGTGAATGTGTTCAGCGTTAATGG
TTTAGGAGATTATGGAAACACAATTCTTGATCCAATTAGCAAGAGAGTATCAGTAGAAGG
TGATGATTGGTTCATTCCCCCCTCGGAGAATACCAACGTCATTGCTTGTAGTACAAGCAA
CAACCTAAACTTACAGGCCCTTGATCCTTGCTTCAATAGCAAAAATCTTTGTCATTCAGA
AAGCTTCAAGGTAGGGAATGTGTTGGGGATAGAGAATGGTTCTTGGGAAATAGAAAACC
CTAAAATCGGAGATTGGGATTTGGATGGTCTCATCGATAACAACTCTTCTTTTCCCTTCCT
TGATTTCCAAGTCGATTGA

>AT3G15030|AT3G15030.1

ATGTCTGACGACCAATTCCATCACCCGCCGCCTCCTTCTTCAATGAGGCACCGTTCTACG
TCGGATGCGGCGGACGGCGGCTGCGGCGAGATTGTTGAGGTGCAAGGTGGTCACATTG
TTCGGTCTACCGGAAGAAAAGACCGCCACAGCAAAGTCTGCACGGCTAAAGGGCCACG
TGACCGGCGCGTGAGACTCTCTGCTCACACGGCGATTCAGTTTTACGATGTTCAAGACA
GGCTTGGTTTCGACCGACCTAGCAAAGCCGTTGATTGGCTTATCAAAAAGGCTAAGACT
TCCATTGACGAGCTCGCTGAGCTTCCTCCCTGGAATCCCGCCGATGCAATTCGCCTAGCC
GCTGCTAACGCTAAACCCAGAAGAACCACCGCCAAAACCCAAATCTCTCCGTCTCCGCC
ACCGCCGCAACAGCAACAACAACAACAACAGCTTCAGTTCGGTGTTGGCTTCAACGGA
GGAGGAGCAGAGCATCCGAGTAACAACGAGTCGAGTTTTCTCCCGCCGTCAATGGATTC
AGATTCGATAGCTGACACTATAAAGTCGTTTTTTCCGGTGATTGGCTCTTCAACGGAGGC
TCCTTCGAATCATAACCTTATGCACAACTATCATCATCAGCATCCGCCGGATTTGCTTTCT
CGAACTAATAGCCAAAACCAAGATCTCCGTCTCTCGCTGCAATCGTTCCCGGATGGTCC
ACCGTCGCTTCTGCACCACCAACATCACCACCACACCTCTGCTTCCGCCTCCGAGCCTA
CTCTGTTCTACGGACAGAGCAATCCGTTAGGGTTTGACACATCGAGTTGGGAGCAGCAG
TCGTCGGAATTCGGAAGGATTCAGAGACTAGTGGCTTGGAACAGCGGCGGTGGCGGCG
GAGCAACCGATACAGGAAACGGAGGAGGGTTTCTGTTCGCTCCTCCTACTCCTTCAACG
ACGTCGTTTCAGCCAGTTCTTGGCCAAAGCCAACAGCTTTATTCTCAGAGGGGTCCCCT
TCAGTCCAGTTACAGTCCCATGATCCGTGCTTGGTTTGATCCTCACCATCATCACCAATCC
ATCTCCACCGACGATCTCAACCACCACCATCACCTTCCTCCACCGGTTCACCAATCAGCA
ATCCCCGGAATCGGATTCGCCTCAGGTGAATTCTCTTCGGGTTTTCGCATACCAGCACGG
TTTCAGGGCCAAGAAGAGGAGCAGCACGACGGTCTCACTCACAAGCCGTCCTCTGCTT
CCTCTATTTCTCGCCATTGA

>AT4G00150|AT4G00150.1

ATGCCCTTACCCTTTGAAGAGTTTCAAGGGAAGGGGATTTCTTGTTTCTCTTCTTTCTCG
TCTTCCTTCCCCCAACCACCGTCGTCTCCGCTTTTGAGCCACCGCAAAGCAAGAGGCGG

CGAAGAAGAAGAGGAAGAAGTTCCCGCGGCGGAGCCTACCTCTGTTCTGGACTCCCTC
ATAAGCCCAACCTCTTCCTCCACGGTGTCTTCCTCTCACGGCGGAAACAGCGCCGTCGG
AGGCGGCGGCGACGCCACCACCGATGAGCAATGCGGAGCCATTGGGTTGGGTGATTGG
GAGGAGCAAGTTCCTCATGACCACGAACAGAGCATTCTCGGACTCATCATGGGAGATTC
CACAGATCCCTCTCTTGAACTCAACAGCATTCTCCAAACATCTCCCACCTTCCACGACTC
TGACTACTCATCACCCGGTTTCGGAGTCGTCGACACCGGCTTCGGTTTAGACCACCACT
CTGTTCCGCCGTCACATGTTTCCGGTCTTCTGATCAACCAAAGTCAAACCCACTACACAC
AGAATCCTGCGGCTATCTTCTACGGCCACCACCACCATACACCTCCGCCGGCAAAGCGG
CTCAACCCTGGTCCCGTGGGGATAACAGAGCAGCTGGTTAAGGCAGCAGAGGTCATAG
AGAGCGACACGTGTCTAGCTCAGGGGATATTGGCGCGGCTCAATCAACAGCTCTCTTCT
CCCGTCGGGAAGCCATTAGAAAGAGCAGCTTTTTACTTCAAAGAAGCTCTCAATAATCT
CCTTCACAACGTCTCCCAAACCCTAAACCCTTATTCCCTCATCTTCAAGATCGCTGCTTA
CAAATCCTTCTCAGAGATCTCTCCCGTTCTTCAGTTCGCCAACTTTACCTCCAACCAAGC
CCTCTTAGAGTCCTTCCATGGCTTCCACCGTCTCCACATCATCGACTTCGATATCGGCTAC
GGTGGCCAATGGGCTTCCCTCATGCAAGAGCTTGTTCTCCGCGACAACGCCGCTCCTCT
CTCCCTCAAGATCACCGTTTTCGCTTCTCCGGCGAACCACGACCAGCTCGAACTTGGCT
TCACTCAAGACAACCTCAAGCACTTCGCCTCTGAGATCAACATCTCCCTTGACATCCAA
GTTTTGAGCTTAGACCTCCTCGGCTCCATCTCGTGGCCTAACTCGTCGGAGAAAGAAGC
TGTCGCCGTTAACATCTCCGCCGCGTCCTTCTCGCACCTCCCTTTGGTCCTCCGTTTCGT
GAAGCATCTATCTCCGACGATCATCGTCTGCTCCGACAGAGGATGCGAGAGGACGGATC
TGCCCTTCTCTCAACAGCTCGCCCACTCGCTGCACTCACACACCGCTCTCTTCGAATCCC
TCGACGCCGTCAACGCCAACCTCGACGCAATGCAGAAGATCGAGAGGTTTCTTATACAG
CCGGAGATAGAGAAGCTGGTGTTGGATCGTAGCCGTCCGATAGAAAGGCCGATGATGAC
GTGGCAAGCGATGTTTCTACAGATGGGTTTCTCACCGGTGACGCACAGTAACTTCACGG
AGTCTCAAGCCGAGTGTTTAGTCCAACGGACGCCAGTGAGAGGCTTTCACGTCGAGAA
GAAACATAACTCACTTCTCCTATGTTGGCAAAGGACAGAACTCGTCGGAGTTTCAGCAT
GGAGATGTCGCTCCTCCTGA

>AT5G60760|AT5G60760.1

ATGACGGAGACGACGAAGGTGATGTACATAGTGGTGGTCGATGGAACGGATACGGCGG
AGACGGTGGAAGAAGACGGGACCGGGACTTGGAAAGATTCGTTTAGGTATACACGTCC
TGTTTTGCAGAGTACTCTTCAGCTTATGGGATGCAAAGCCCGTCACGCCTTCAAGATTAG
CCGCAGGGTGTTTGAGTTAATAAGAAGTGAGGGATCTTTAATCCTTTCTCCCTCGCTTTC
GCCCTCGCATAGTAAAGAATCTGAATTTCAGAAGACAGGTGATGCTTCAGCTTGTGCTAA
TGTAGAAAAGGCTAACAAAGTTAACAGTTTGGCTACTGATGATGTTGATAAGAGTAAAA
GTAAGCCGTTTGAGGTGTACAAAAGGCGAACAACCGTTGTTGTTTCTCGTGAAATATTT
GTAAACGTTGTCTGTGATGCCCTGGCTGAGTACAAGTATGTGGGTCATGACCAAAGGGC
AGACTTGATTCTGTCGTGCAGAATCCGAGAAAGGAAAGAATCTGTGACTGTTCTACTGT
GCGGTACTAGTGGCTGTGGCAAATCTACACTCTCCGCACTTCTGGGAAGTAGGCTTGGG
ATTACAACGGTGGTCTCAACCGACTCAATCCGTCACATGATGAGGAGTTTCGTTGATGA
AAAGCAGAATCCTTTGCTATGGGCTTCAACATACCATGCTGGAGAGTACCTTGACCCTGT
GGCAGTTGCTGAGTCAAAAGCCAGAAGAAGAAGAGCCAAAAAAATGGATAGTATTGAG
GACGAAAAAGCGAAAGCCTCTGAAGGTGGGAAGGCTAAGAATACTCAACAGACTGATG
TTGGATCAACAAAAAACACCCCTGTATTGTTAAGTCCCAAGCAAATGGCTGTAGAAGGA
TTTAAGGCTCAAAGTGAAATGGTTATTGACAATCTCGATAGGCTCATTACCGCATGGGAA
GAAAGGAAAGAGTCTGTTATTGTTGAAGGTGTCCATTTAAGCCTCAACTTTGTGATGGG
ACTGATGAAAAAGCATCCTTCTATAGTTCCTTTCATGGTATACATCGCAAACGAGGAGAA
ACATTTGGAACGATTTGCAGTTAGAGCTAAGTACATGACCCTGGACCCAGAGAAAAACA
AGTATGTAAAATATATACGTAACATCAGAACAATACAGGATTACTTGTGTAAACGAGCTG
ACAAACATCTCGTCCCCAAGATAAACAATACCAATGTCGACAAGAGTGTGGCTGCGATC
CACGCTACAGTCTTCAGTTGCCTGCGTAGGCGAGAAGCAGGAGAGCATCTCTATGATGC

TACTACAAATACTGTCTCAGTGATTGACGATGAGTATAGAAACCAGTGTACAGCTAATTC
ATTGAGCTCCAAAGGAATGTTTCAGCTGATCCAAAGAAAAGGTTCTTCTAGGCATCTTAT
GGCTCTTCTCAATACTGATGGCACTTTCGCAAGAACTTGGCCCGTTACTGGTAAGGTCGA
TGAGAGTGGAAAGCCAGTCTTTTGTAATGAGATGATTGAAGAAAATGGAATGGAGCACC
CGGTCTATGGATACCTACAGAAAGCTGAACCGGTGAATCTTCAGTTTGGTCTATTTGGGA
TCAGTGCTTGGCCTAGTGATGGAGCCACTAGCCGCGCTGGGAGTGTAGATGACTGTAAA
GCTGACATGGCTGAAACCAGCAGTCGGTATTACTCTTCTTGCTGCAGCTCGCCTAGGATG
TCTGAAGGAACTTCTAAAGAGCTTAAGGAGGATCAGTCAGTCCATGGGAGCGATGAAG
AAGTCGAGGATGATCCTCCCGAGCCAGATACAGATTTTAGCGATGACGATAACAAGCGA
GACCATGACGAGGTGGGTTCGGTTGATGAGCAATCAACAAAGTCAGATGAGGAGTACG
ATGATCTTGCTATGGAAGACAAAAGCTACTGGACAGATGAAGAGGAAGAGTCACGAGAT
ACGATTGCAATAATGTCTGAAAAAAACAACAAGCAAGCAACAAAAGAAGACAAGTACA
TACAGAACTTAGACCTTTTTCTACGGACCGCAAACCAGCAGCTTGTCGAGCCACTTCAG
CTATGTGCATCACTGCTCACATGCGAGAATGGGAACACAAGACTCTGGTTGGGTAAAGA
AAAGATGAGGAAACGTTCTTTAAGCATCTCGGCCATTGGCAAACATGGGTCGGGTTTGG
GTGATGCGATTCTCTTGGGTGCCCCATGA

>AT4G30080|AT4G30080.1

ATGATAAATGTGATGAATCCAATGAAAGGTGGAACAGAGAAAGGTTTAGATCCTCAGCT
ATGGCATGCATGTGCTGGTGGTATGGTTCGTATGCCTCCTATGAACTCTAAAGTCTTTTAC
TTTCCTCAAGGTCACGCCGAAAACGCTTACGATTGTGTCGATTTCGGTAATCTCCCTATT
CCTCCCATGGTTTTGTGTCGTGTTTTAGCCATTAAGTATATGGCTGATGCTGAATCTGACG
AGGTTTTCGCTAAACTGAGATTGATTCCTTTGAAAGATGATGAGTATGTTGATCACGAGT
ATGGTGATGGTGAAGATAGTAACGGTTTCGAGAGTAATAGTGAGAAAACGCCTTCGTTT
GCTAAGACTTTGACTCAGTCTGATGCTAATAACGGTGGGGGTTTCTCTGTTCCTCGTTAT
TGCGCTGAGACGATTTTCCCGAGGTTGGATTATAACGCCGAGCCGCCGGTTCAGACCAT
TCTTGCTAAGGATGTTCATGGTGATGTTTGGAAGTTCAGACATATTTATAGAGGGACGCC
TCGGCGTCACCTTCTTACAACCGGATGGAGTAATTTTGTAAACCAGAAGAAGCTTGTGG
CGGGAGATTCGATTGTCTTCATGAGAGCGGAGAATGGAGATCTTTGTGTAGGTATTAGGA
GGGCTAAGAGAGGAGGGATAGGTAATGGACCCGAATATTCAGCGGGTTGGAATCCGATC
GGTGGAAGTTGCGGCTACTCTTCTCTGTTAAGGGAAGATGAAAGCAATAGTTTGAGGAG
AAGTAATTGTTCCCTTGCGGATAGGAAGGGGAAAGTGACGGCTGAATCTGTTATAGAAG
CAGCCACTCTTGCTATTAGCGGAAGACCGTTTGAGGTTGTGTACTATCCGAGAGCTAGCA
CTTCAGAGTTTTGTGTCAAGGCATTAGATGCTCGAGCTGCCATGCGGATTCCGTGGTGCT
CAGGTATGAGGTTTAAGATGGCTTTTGAGACAGAGGATTCGTCTCGGATAAGTTGGTTTA
TGGGGACTGTTTCAGCTGTTAATGTCTCTGATCCTATCCGTTGGCCTAACTCTCCTTGGCG
GCTTCTACAGGTGGCGTGGGATGAGCCAGATTTACTCCAAAACGTGAAGCGAGTTAACC
CGTGGTTGGTGGAATTGGTATCAAACGTACATCCGATCCCGCTTACTTCGTTTTCGCCAC
CGAGGAAAAAGATGCGGCTACCTCAGCATCCAGATTACAACAATCTGATCAATTCGATTC
CAGTACCTTCATTCCCAAGCAATCCCCTTATTAGATCAAGCCCGTTAAGCTCTGTTCTGG
ACAATGTTCCCGTGGGTTTACAGGGAGCCAGGCATAATGCTCATCAGTACTACGGGTTAT
CATCTTCGGATCTTCACCATTACTACTTGAATAGACCACCTCCTCCTCCTCCTCCATCCTC
TCTCCAACTTTCTCCTTCTCTCGGTCTCCGAAACATCGATACCAAAAACGAAAAAGGATT
TTGCTTTTTGACAATGGGAACAACACCATGCAATGATACCAAATCTAAAAAGTCCCATAT
TGTATTGTTCGGCAAGCTTATACTACCCGAGGAACAGCTATCAGAAAAAGGCTCAACGG
ATACCGCAAACATAGAGAAAACGCAGATTTCATCAGGCGGGTCGAACCAAAACGGCGT
TGCGGGAAGGGAGTTTTCTTCGTCAGATGAAGGATCACCTTGCTCTAAGAAAGTTCATG
ATGCATCAGGTTTGGAAACAGGGCATTGTAAAGTGTTTATGGAGTCAGACGATGTAGGT
CGAACCTTAGACCTATCGGTTCTTGGTTCATACGAAGAATTGAGTCGGAAACTCTCTGAC
ATGTTTGGAATCAAAAAGTCTGAGATGTTAAGCTCTGTTCTCTATAGGGATGCATCAGGA
GCCATCAAATACGCAGGAAACGAACCTTTCAGTGAGTTCTTGAAGACAGCTCGAAGATT

GACAATTCTGACGGAACAAGGAAGTGAGAGCGTTGTAGTATAA

>AT2G34010|AT2G34010.1

ATGTGTAGTAACAACAACACAAGTAGTGGAAGCTATGGAGAGTTACAGTTACATGATGA
GTCCTCTGGTTCTTGTCGGAAGAAACAGAAGAAAGACAAAGTACGACGAAGAGGACCT
GGTGTTGCTGAACTCGAGAAGATCCGTCTTCAAGAAGAGTACAAACCTCCACTTTCTTC
GTCTCCTTCATTACCAAACATTGATCATCATCATCACACTCTCTTTGCTCCGGCTAGTAGC
GTCTATGATTTAGTGATGACTTCTCCAAACTTTTCTTTCCCCGAGAAACTTGCGTCAACG
TTACCGGTTTTCCCGATTTCGTACGGATCTTTGATTCCTCCGGCGCCGATTTTTCAGAGGA
GCCAACATTCTCTAATGATGAATCTACCGAATCCATCTCCAGGAGCAGGAAGATTTTATC
AATTCATAGAGCCCCCTTCAAACCAAAGATCTTGCGTCGATTCTGTGTCTCAGTTTCTTG
AAGAAGAAAATAAAAAGATATTCACTGCTAAGAAGAGGCCATGGCCTTTTCTCACTGAC
ACCACGAAACCTAGTGTTGGACCAACCACAACATCAACAATCATAAGGCCGGATGCGAC
ACAAAACCGGTCAATGGGTATAACTCCGGTTCAAGAGACCGGTACAACAACTAGCAAC
CCCATCGCCATTGATTCACCTACGTCCATTCCACGTCATTACCCGAGGTTTATCCCGCTTG
GTTTACAGTATGAACAACAACAACAAAAACTAAAAGACTTGGATGAGACTATGCAATGG
AGAAGTAAGAAGCCTTTCTACAGCTTTATACCCTCTGGTGATCCGAGCAATGATGACCAA
GAACAACGACCATGTGACCTATTTGGATCAGCTGCTGATCATGGAATTGATCTCAACCTT
AAGTTATAG

>AT5G06510|AT5G06510.2

ATGCAAACTGAGGAGCTTTTGTCGCCACCACAGACTCCTTGGTGGAATGCTTTTGGATC
TCAGCCGTTGACTACAGAGAGCCTTTCCGGCGAAGCTTCTGATTCATTCACCGGAGTTA
AGGCAGTTACTACGGAGGCAGAACAAGGTGTGGTGGATAAACAAACTTCTACAACTCT
CTTCACTTTCTCACCTGGTGGTGAAAAGAGTTCAAGAGATGTGCCAAAGCCTCATGTTG
CTTTCGCGATGCAATCAGCTTGCTTCGAGTTTGGATTTGCTCAGCCAATGATGTACACAA
AGCATCCTCATGTTGAACAATACTATGGAGTTGTTTCAGCATACGGATCTCAGAGGTCTT
CGGGCCGAGTAATGATTCCACTGAAGATGGAGACAGAAGAAGATGGTACCATCTATGTG
AACTCAAAGCAGTACCATGGAATTATCAGGCGACGCCAGTCCCGAGCAAAGGCTGAAA
AACTGAGTAGATGCCGTAAGCCATATATGCATCACTCACGCCATCTCCATGCTATGCGCCG
TCCTAGAGGATCTGGCGGGCGTTTCTTGAACACCAAGACAGCTGATGCGGCTAAGCAGT
CTAAGCCGAGTAATTCTCAGAGTTCTGAAGTCTTTCATCCGGAAAATGAGACCATAAACT
CATCGAGGGAAGCAAATGAGTCAAATCTCTCGGATTCTGCAGTTACAAGTATGGATTACT
TTCTAAGTTCGTCGGCTTATTCTCCTGGTGGCATGGTCATGCCTATCAAGTGGAATGCAG
CAGCAATGGATATTGGCTGCTGCAAACTTAATATATGA

>AT5G43270|AT5G43270.1

ATGGAGTGTAATGCAAAGCCACCGTTTCAATGGGAATTGGAAAACTTGATATCTTTTGGC
ACTTCTACAGCTGAAGTTCCTAGAAAGCTAAAACCAATGGAGTGGGAAATTGATGGATT
TGATTGCACCTCTCTGTATTCTTCAAGCTTTGCCTATGCTGGTAGTTCAGGTTCTGATATA
GCTCATGCTTTCTCTAAAAGCTCAAAGTCAACTTCCATTAGCTCTTCATCAGCTGAAGTA
AGAACACACAATTTTACATCCGAAACTGGTGAAAGTCTTCCTGGAGAATTTGCAAAGGG
GATTGATACTTCTCCAAGTCTTGAACTTTCTTTTGGCTCTGGTGATCCGGTTCTCGGTTTA
AAGCTTGGTAAACGAACGTACTTTGAAGACTTTTGGGAAGTGGAGAATGCTAAAGGTTT
GGGACTTCCAGTGACTCTGGCTTCATCTTCTGTTTCTCCCGTGAAGAAATCGAAATCCAT
TCCTCAGAGGTTACAAACTCCTCACTGTCAAGTTGAAGGCTGTAATCTTGATCTTTCATC
AGCTAAAGACTATCATCGGAAACATAGGATTTGTGAAAATCATTCAAAGTTTCCTAAAGT
CGTTGTGAGTGGCGTAGAGCGTCGGTTCTGCCAACAATGTAGCAGGTTCCACTGTCTCT
CTGAGTTTGATGAGAAGAAACGTAGCTGTCGCCGACGTCTCTCAGATCACAATGCAAGA

CGTCGCAAGCCAAATCCTGGAAGGACATATGATGGGAAACCACAGGTGGATTTTGTATG
GAACAGATTTGCACTTATCCATCCAAGAAGTGAGGAAAAGTTTATATGGCCAAGTTCGA
AGCACGTACCATCAAGAGTGTTAATGCCGCAACCTGCAAAGACTGAGATTTCCGATACC
GAGCACAATAGATTTGGATTGTTGGACCCCAAAACCAAAACCGCAAGAGCCGAGTTATT
CAGTAAAGAAAAGGTCACAATCTCTTCACACATGGGTGCTTCTCAAGATCTTGATGGTG
CTCTCTCTCTTCTGTCAAATTCAACAACATGGGTTTCTTCCTCTGACCAACCAAGGCGTT
TTACCCTTGATCACCATCCCTCAAGCAACCTCCAACCCGTAGCTCACCGGTCTGCGGCTC
AACTCAATTCAGTTTCCGGCTATTGGCAGCCGGACCCACCCGCAGTTGAAGGCCCGACC
GCTCTGCATAGAAATGGGGTAGGCCAGTTTAATGAAAACTACTTCAGCTTGAACCAGTT
TTATAACTGA

>AT2G33770|AT2G33770.1

ATGGAAATGTCCCTTACTGACTCTGATTGGGATAGCTCCAGCGACAGTGGTAGCAGTGA
ACACGAAGAAGTCGAGTTTTCTTATGGTGGACGGGCACAGAACATTTTCTCAAACCTTG
AAGAAACCATTGGCAAAATCGATGAGTTCCTGTCGTTCGAGAGGGGATTTATGTATGGT
GACATTGTGCGGTCCGCCACTGAACCATCAGGACAGAGTGGCAGGGTTATCAACATAGA
CATGTTTGTCAATCTCGAAAGTACTCATGGGAAGATCATGAAGGAAGTTGATACCAAGA
GGCTTCAAAAGTTGCGTTCTATTTCACTCTCTGATTATGTGATTAACGGACCTTGGGTTG
GAAGGGTTGACAAAATAGTTGAGCGTGTCTCTGTCACCCTTGATGATGGGACCAACTAT
GAGGTCCTTGTAGATGGTCAAGATAAACTTGTGGCCATTCCCCCAAATTTACTCGAGGAT
TCTCAATATTCGTATTACCCAGGGCAAAGAGTTCAGGTAAAGCTGGCCCATGCCCCCAGA
TCAACTACATGGTTATGCGGGACCTGGAGAGGAACCCAGGTTATGGGAACTGTTTGCAC
TGTAGAAGCAGGACTTGTCTACGTCGATTGGGTTGCCTCCATCGTAATGGAGGGTGATCG
GAATTTAACTGCACCTCAAGCTTTGCAGAATCCTGAGAGTTTAACTTTGTTACCTTGTGT
TTCTCATGCGAGTTGGCAGCTTGGTGACTGGTGTATACTCCCTGGCTCTTCCCACTGTGA
TATAGCAGAGCGGCAAACTCCAAATGTGGCTGCCTACAATCTCAATGAATGCCATAAGA
CATTCCAAAAAGGGTTTAACAGAAATATGCAGAACTCAGGTTTGGATGAGCTATTTGTTA
TCACAAAGACAAAGATGAAGGTTGCTGTTATGTGGCAAGATGGTAGCTGCAGTCTGGGA
GTTGATTCCCAACAGCTGCTTCCTGTTGGTGCTGTTAATGCTCATGATTTTTGGCCCGAA
CAGTTTGTTGTGGAAAAGGAAACCTGCAACAGCAAAAAATGGGGAGTTGTGAAGGCTG
TCAATGCTAAGGAACAAACTGTGAAGGTACAATGGACAATACAGGTTGAGAAAGAAGC
AACAGGTTGTGTTGATGAGGTGATGGAAGAAATTGTCAGTGCATATGAACTGCTTGAGC
ACCCTGATTTTGGATTCTGTTTCAGCGATGTGGTGGTCAAGTTACTTCCAGAAGGAAAAT
TTGATCCAAATGCAGACACAATCGTCGCTACAGAGGCGAAACACCTACTTACAGAGAGT
GACTACAGTGGCGCATATTTTTTGTCAAGTATTGGTGTTGTTACAGGTTTTAAAAATGGTT
CCGTGAAGGTGAAATGGGCCAATGGTTCTACTAGCAAGGTTGCACCATGTGAAATTTGG
AAAATGGAAAGGTCTGAATATTCCAACTCTAGCACTGTAAGTTCGGAAGGCAGTGTTCA
AGATCTAAGTCAGAAGATTAGTCAATCGGATGAAGCATCTTCAAACCATCAGGAAACGG
GTCTGGTGAAGCTCTACAGTGTTGGTGAAAGTTGCAACGAGAACATTCCGGAATGTAGT
TCATTTTTCCTTCCAAAAGCTGCCATTGGATTTATCACAAACCTTGCATCAAGCCTTTTTG
GTTATCAGGGTTCCACTTCGGTTATAAGTTCACATTCACGTTGCAATGATTCTGAAGATCA
AAGTGACTCTGAGGTCCTTGTTCAAGAAACAGCAGAATCATATGACAACTCTGAAACAA
ATTCAGGTGAAGTGGATATGACCACCACGATGGTCAACATACCTATAGAAGGAAAAGGA
ATTAACAAGACACTGGATTCAACTCTTCTAGAGAACAGCAGGAACCAAGTGAGATTCAG
ACAGTTTGATATGGTTAATGACTGCTCGGACCATCATTTCCTTTCTTCGGATAAAGGGTTG
GCTCAGTCCCAGGTCACAAAGAGTTGGGTGAAGAAAGTCCAGCAAGAATGGAGCAATT
TGGAGGCAAATCTTCCGAACACGATATATGTGCGTGTGTGTGAAGAAAGGATGGACCTT
CTGCGTGCAGCCCTGGTTGGTGCCCCTGGAACGCCATATCACGATGGGCTTTTCTTTTTC
GACATAATGCTTCCACCTCAATATCCTCATGAGCCACCAATGGTACATTATCATTCAGGTG
GGATGCGACTGAATCCGAACCTGTATGAGTCAGGAAGAGTTTGCTTGAGTCTGCTGAAT
ACATGGAGTGGCTCTGGCACTGAAGTATGGAACGCAGGGAGCTCCTCCATCCTTCAACT

TCTTCTTTCGTTTCAGGCTCTGGTTCTGAATGAGAAGCCTTACTTCAATGAAGCTGGCTA
TGATAAGCAGTTGGGCCGAGCCGAGGGAGAGAAAAACTCAGTGAGTTACAATGAGAAT
GCATTCCTCATAACCTGCAAATCCATGATCTCAATGCTCCGTAAGCCTCCAAAGCATTTT
GAGATGCTTGTGAAGGACCATTTTACGCACCGGGCCCAGCATGTTCTGGCTGCGTGCAA
GGCTTATATGGAAGGCGTCCCTGTAGGATCATCAGCTAACCTGCAGGGGAACTCAACCA
CAAATTCCACCGGTTTCAAGATCATGCTCTCCAAACTCTACCCAAAACTTCTTGAAGCAT
TCTCAGAGATTGGAGTTGATTGCGTCCAAGAGATTGGACCAGAATCATAA

>AT5G07680|AT5G07680.2

ATGGATTTGCCTCCTGGTTTTAGGTTTCATCCAACAGACGAAGAACTCATAACCCACTAT
CTCCACAAGAAGGTTCTTGACCTTGGTTTCTCGGCTAAAGCTATTGGTGAGGTTGATTTG
AACAAAGCTGAGCCATGGGAGTTGCCATATAAAGCAAAAATTGGTGAGAAAGAATGGT
ACTTTTTCTGTGTGAGGGATAGAAAGTATCCGACTGGTCTGAGGACTAACCGAGCAACT
CAAGCCGGTTATTGGAAGGCGACAGGGAAGGATAAAGAGATCTTCCGAGGCAAATCGC
TTGTTGGTATGAAGAAAACACTTGTTTTCTACAGAGGAAGAGCTCCTAAAGGCCAGAAA
ACAAATTGGGTGATGCATGAGTATAGGCTAGATGGAAAACTCTCTGCTCACAACTTGCCT
AAAACCGCTAAGAATGAATGGGTGATCTGCAGGGTATTTCATAAAACTGCTGGAGGTAA
GAAGATCCCGATTTCGACGTTAATCCGAATCGGTTCTTACGGAACCGGGTCTAGTTTACC
ACCTTTAACTGATTCTTCACCATACAACGACAAAACCAAGACCGAACCAGTTTACGTGC
CCTGCTTCTCCAACCAAGCTGAAACTAGAGGAACAATACTCAATTGCTTCAGCAACCCT
TCCCTTAGCTCCATCCAACCAGATTTTCTCCAGATGATTCCACTCTACCAACCTCAATCTC
TCAACATTTCCGAGAGTTCCAATCCGGTTCTTACGCAAGAACAATCAGTTTTACAAGCGA
TGATGGAAAACAACAGAAGGCAGAACTTCAAAACATTGAGCATCTCGCAAGAAACCGG
GGTTTCGAACACCGATAACTCATCGGTTTTTGAATTTGGGAGGAAGCGATTTGATCATCA
AGAAGTTCCATCTCCCTCTTCCGGTCCGGTTGATCTTGAACCTTTCTGGAATTACTGA

>AT5G43780|AT5G43780.1

ATGGCTTCTTCAGCAGCAGCCATCGTCTCCGGTTCTCCATTTCGTTCTTCACCATTAATCC
ATAACCATCATGCTTCTCGTTATGCTCCTGGATCCATCTCCGTCGTATCTCTCCCCCGTCA
AGTTTCCCGCCGTGGCCTCTCCGTTAAATCGGGGTTGATCGAGCCAGACGGAGGAAAAC
TCATGAACCTCGTAGTCGAAGAATCACGGCGGCGCGTGATGAAACACGAGGCGGAGAC
GGTTCCGGCAAGGATCAAGCTGAACCGTGTGGATCTAGAGTGGGTACATGTGCTTAGCG
AAGGCTGGGCAAGTCCTCTCAAAGGTTTCATGAGACAGTCAGAGTTCCTCCAAACACTT
CATTTTAACTCGTTCCGGCTCGAAGACGGCTCCGTCGTCAACATGTCGGTTCCGATCGTC
CTCGCTATCGACGACGATCAGAAGTTTCGTATCGGGGATTCCAACCAAGTCACACTCGTT
GACTCGGTTGGTAATCCCATCGCGATTCTCAACGATATTGAGATTTACAAGCATCCCAAA
GAAGAACGAATCGCGAGAACATGGGGAACCACGGCTCGTGGGCTTCCTTATGCGGAAG
AAGCAATCACCAAAGCTGGAAACTGGTTGATTGGAGGTGATTTACAAGTTTTGGAGCCG
ATCAAGTACAATGATGGTTTAGACCGGTTTCGTTTATCTCCGTCGCAGCTGCGGGAGGAG
TTTATAAGGCGCGGTGCGGACGCGGTTTTCGCGTTCCAGCTTAGGAACCCGGTGCATAAT
GGTCATGCGCTTCTTATGACGGATACTCGTAGAAGACTTCTTGAGATGGGTTACAAAAAC
CCTGTCTTGTTGTTGAATCCACTTGGTGGGTTTACTAAGGCTGATGATGTACCTCTCAGC
TGGCGTATGAGACAGCACGAGAAGGTGTTGGAGGATGGTGTACTGGATCCAGAGACCA
CTGTAGTCTCTATCTTCCCATCTCCGATGCTATACGCTGGCCCAACTGAGGTTCAATGGCA
TGCCAAAGCTAGGATCAACGCGGGAGCTAATTTCTACATTGTTGGTCGCGATCCTGCCGG
TATGGGCCATCCGACAGAGAAGAGAGATCTTTATGATGCCGATCACGGGAAGAAAGTGC
TTAGCATGGCACCAGGACTTGAACGCCTCAACATTCTACCCTTCAAGGTCGCTGCATATG
ACAAAACTCAGGGGAAAATGGCCTTCTTTGATCCTTCCAGGTCTCAAGACTTCCTCTTC
ATATCAGGAACCAAGATGCGTGGGTTGGCGAAGAAGAAAGAGAACCCACCAGATGGTT
TCATGTGTCCCTCTGGTTGGAAGGTGTTGGTTGACTACTACGACAGTCTCAGTGCAGAG

ACTGGTAATGGAAGGGTTTCAGAAGCTGTTGCTTCTGCTTGA

>AT3G09220|AT3G09220.1

ATGGAGGGAGTTAGGGTTCCTATTGCTTGCGCTCTAATTCTTCTCGCAATCTCTTCCATAA
CTTCTGCCTCCATTGTGGAACACACCTTCAACGTACAAAACTTAACGGTGTCTAGGCTGT
GCAAACGGCAAGTGATAACGGTCGTTAACGGAAGCCTGCCTGGACCAACAATACGCGT
CAAGGAGGGTGACTCGCTCGTTATTCACGTTCTAAACCATTCCCCTCACAACATCACAAT
TCATTGGCATGGGATCTTCCATAAACTAACGGTTTGGGCAGATGGACCGAGTATGATAAC
GCAGTGCCCAATCCAGCCTGGCCAAAGATACGCTTACAGATTCAACATCACCGGCCAAG
AAGGCACCTTGTGGTGGCATGCACACGCGTCTTTCCTACGCGCCACCGTATACGGTGCT
CTCGTCATCCGCCCTAAATCTGGTCACTCTTACCCGTTTCCCAAACCTCACAAAGAAGTT
CCCATCTTATTTGGTGAATGGTGGAACACCGATGTAGTCGCGTTAGAGGAGGCAGCAAT
CGCCACCGGAGTTCCTCCAAACAATTCCGACGCTTATACGATCAATGGCCGTCCCGGAA
ACCTATATCCATGCTCCAAAGACAGAATGTTTAGCCTAAACGTGGTGAAAGGAAAGAGA
TACTTACTTCGCATCATAAACGCGGCCATGAACATCCAACTGTTTTTCAAGATAGCCAAT
CACAGACTGACAGTTGTAGCCGCCGACGCAGTCTACACCGCACCTTACGTCACCGACGT
CATTGTCATCGCACCGGGCCAAACCATCGACGCCCTTCTCTTCGCCGATCAGTCCGTCGA
TACCTCCTACTACATGGCAGCTCACCCTTACGCCAGCGCTCCCGCCGTCCCTTTCCCAAA
CACCACCACCAGAGGCGTCATTCACTATGGCGGCGCGTCGAAAACAGGGCGATCAAAG
CCCGTGTTGATGCCGAAACTACCTTCCTTCTTCGACACGTTAACGGCTTACAGATTCTAC
TCCAACCTCACTGCCTTAGTCAATGGACCTCACTGGGTTCCAGTTCCACGCTACGTGGA
CGAAGAGATGCTCGTAACCATCGGTCTTGGGCTAGAGGCATGCGCAGATAACACCACGT
GTCCAAAATTCTCAGCTTCTATGAGCAATCACTCTTTCGTGTTACCCAAGAAGCTATCGA
TCTTGGAAGCAGTGTTTCACGACGTTAAAGGGATTTTCACCGCGGATTTTCCAGACCAG
CCTCCGGTTAAGTTTGATTACACGAACCCGAACGTAACCCAAACAAACCCGGGACTTTT
GTTCACTCAAAAATCAACTAGCGCCAAGATTTTGAAATTCAATACGACGGTGGAGGTAG
TGTTACAGAACCATGCGCTTATAGCGGCGGAGAGTCATCCCATGCATCTACATGGATTTA
ATTTCCATGTATTGGCTCAAGGGTTTGGTAATTATGACCCGAGCCGTGACCGGAGCAAGT
TAAATCTCGTTGACCCGCAATCCCGTAACACTTTGGCTGTCCCTGTTGGTGGATGGGCTG
TGATCCGTTTCACTGCTAATAATCCAGGTGCGTGGATTTTTCATTGTCATATCGATGTTCA
CTTACCATTCGGACTAGGGATGATATTTGTGGTTAAGAACGGACCGACCAAGTCGACCA
CCTTGCCACCGCCACCACCAGATCTTCCCAAATGTTAA

>AT3G62980|AT3G62980.1

ATGCAGAAGCGAATAGCCTTGTCGTTTCCAGAAGAGGTACTAGAGCATGTGTTCTCGTTT
ATTCAGCTGGATAAGGATAGGAACTCAGTCTCTCTGGTGTGCAAGTCATGGTACGAGATC
GAGCGGTGGTGCAGGAGGAAAGTCTTCATCGGGAACTGCTACGCCGTGAGTCCAGCGA
CGGTGATTAGGAGGTTCCCGAAAGTGAGATCCGTGGAGCTTAAAGGAAAACCTCACTTT
GCTGACTTTAATTTGGTACCTGACGGATGGGGAGGTTACGTGTATCCATGGATTGAGGCC
ATGTCTTCGTCTTACACGTGGCTTGAAGAGATAAGGCTGAAGAGGATGGTGGTCACCGA
CGATTGCTTGGAGCTCATAGCCAAGTCTTTTAAGAATTTTAAGGTTCTTGTGCTTTCTTCC
TGCGAAGGCTTCTCCACCGATGGTCTCGCTGCTATCGCTGCCACTTGCAGGAATCTGAA
AGAGCTTGACTTACGAGAGAGTGATGTTGACGACGTTAGTGGCCACTGGCTTAGCCATT
TCCCAGATACATACACTTCTTTGGTATCACTCAATATATCTTGCTTAGCATCTGAGGTCAG
TTTCTCTGCTCTGGAAAGGCTGGTGACTAGGTGTCCCAATCTCAAGTCTCTCAAGCTTAA
CCGAGCTGTTCCACTTGAAAAATTGGCTACTTTACTTCAAAGAGCACCTCAATTGGAGG
AATTGGGCACTGGTGGGTACACTGCAGAAGTGCGACCAGATGTTTACTCTGGTTTATCTG
TAGCGCTCTCTGGGTGCAAGGAATTGAGGTGCTTATCTGGATTTTGGGATGCTGTTCCTG
CCTATCTTCCAGCAGTTTATTCGGTTTGCAGTCGGCTTACAACTTTGAATCTGAGTTATGC
AACAGTCCAGAGCTATGATCTTGTCAAGCTTCTTTGTCAATGCCCTAAACTGCAGCGCCT

CTGGGTGCTTGACTACATCGAGGATGCTGGTCTTGAGGTGCTTGCTTCAACCTGCAAGG
ACCTACGCGAGCTGAGAGTGTTTCCGTCCGAGCCTTTTGTCATGGAACCAAATGTGGCA
TTGACGGAACAGGGGCTTGTCTCCGTCTCCATGGGCTGTCCAAAACTCGAGTCGGTTCT
CTACTTCTGCCGTCAAATGACCAATGCTGCATTGATAACCATTGCTAGGAACCGTCCCAA
CATGACTCGCTTCCGTTTGTGCATCATTGAGCCAAAAGCCCCAGACTATCTGACTCTAGA
GCCACTGGATATTGGATTTGGAGCCATAGTAGAGCACTGCAAGGATCTCCGTCGCCTCTC
TCTATCTGGCCTCTTGACCGACAAGGTTTTTGAATACATTGGGACATATGCCAAGAAGAT
GGAAATGCTCTCAGTGGCATTTGCAGGAGACAGTGACTTAGGCATGCATCATGTTTTGTC
CGGGTGCGATAGCTTGAGGAAACTAGAGATAAGGGACTGCCCGTTTGGAGACAAGGCG
CTTTTGGCCAATGCTTCAAAGCTGGAGACAATGCGATCCCTTTGGATGTCTTCTTGTTCC
GTGAGTTTTGGAGCCTGCAAGTTACTAGGACAGAAGATGCCAAAGCTGAATGTGGAAG
TCATCGATGAACGGGGTGCACCGGACTCGAGACCAGAGAGCTGCCCTGTTGAGAGAGT
CTTCATATACCGAACAGTGGCTGGTCCTCGATTTGACATGCCTGGCTTCGTCTGGAACAT
GGACCAAGACTCAACAATGAGGTTTTCCAGGCAAATCATTACTACTAACGGATTATAA

>AT4G30440|AT4G30440.1

ATGCCTTCAATAGAAGATGAGCTGTTTCCGTCAACGCCGGGTAAATTCAAAATTGACCG
GTCAAACCGTCAGCTCCACCGTTGTTTCGCTTCTACGAGCACCATGTTTCTTTGGGCTCT
CTTCCTCATCGCTCTCACCGCTTCTTACTTGAGTTTCCAAAGCTTCGTCGATTCCGGTAG
CCGTTATCTAACCGCTTCTTGGGGCGGCATCCAGTGGGAGAAACAGGTTCGTACCTCCG
CTCAGATCCATCGCTCCGGCGGTATCTCCGTCCTTGTTACCGGCGCTACCGGATTCGTCG
GTAGCCACGTGTCACTTGCCTTGAGGAAACGCGGCGATGGTGTTGTTGGACTTGATAAT
TTCAACAATTACTACGATCCGTCGTTGAAACGTGCTCGTAGATCTCTGTTGTCGTCGAGA
GGGATCTTCGTCGTTGAAGGAGATCTAAACGACGCGAAGCTGTTAGCAAAGCTTTTCGA
TGTGGTTGCTTTCACTCACGTGATGCATCTCGCTGCTCAAGCTGGTGTTAGATACGCTTT
GGAGAATCCTCAATCGTATGTTCATAGCAACATCGCCGGACTTGTGAATCTTCTCGAGAT
TTGCAAAGCGGCGAATCCTCAGCCGGCGATAGTTTGGGCTTCATCGAGCTCCGTTTATGG
ACTCAACGAGAAAGTTCCATTCTCTGAATCTGACCGGACGGATCAACCGGCGAGTCTCT
ACGCCGCAACGAAAAAAGCCGGCGAAGAAATCACCCACACTTATAACCATATTTACGGT
CTTGCCATTACCGGTTTAAGATTCTTCACGGTTTACGGACCATGGGGTAGACCGGATATG
GCTTACTTCTCCTTCACCAGAAACATCCTACAAGGTAAACCGATCACAATCTACCGGGGC
AAAAACCGGGTCGATTTAGCCCGGGATTTCACATACATCGACGATATAGTGAAAGGATGT
TTAGGATCTCTGGATTCATCGGGTAAAAGTACCGGGTCGGGTGGTAAAAAACGTGGAGC
AGCACCGTACCGGATATTTAACCTGGGAAACACATCTCCGGTTACAGTACCGATTCTGGT
GGATATATTGGAGAAGCATTTAAAGGTGAAAGCGAAGAGGAACTTCGTGGAGATGCCAG
GAAACGGCGACGTACCGTTCACACATGCCAATATTAGCTCAGCCCGAAATGAATTCGGG
TATAAACCGACAACCGATTTGGAAACCGGGTTGAAGAAGTTCGTTAGATGGTATCTTTCT
TATTACGGATACAATACTAAAGCCAAGCTTGTACATTAA

>AT3G17185|AT3G17185.1

Sequenceunavailable

>AT5G38550|AT5G38550.1

ATGATCCAAAAGTTGGGAGCGAAAGGAATCAAGTCAGATGAACGTAATCAGCGGGAGT
GGGATGATGGATCTGAACATGATGATGTAACAAAGATATACGTACGAGGTGGTCGTGAA
GGAATAAGATCCATTTATTTCAACTATGTGAAGAATGGAAAACCCAAAGATGGATCAATC
CATGGTTACTTTGACTCTGGTTTCACACAAACGTTTGAGATTAACCATCTACGAGGTGAA
TATCTTGAATCTGTTGACGCTTACTACGACAAAAAGTCCTACGGTATGCAAGCAATTCAA
TTCAAAACTAACTTCAGGACTTCTGAACTGATGGGGTATAGTTATGAGTGTACTATGTTT

ACGCTAGCCGTCCAAGGAAAGAAGATCATTGGATTTCATGGATCTAATTATGTACATATAT
TATCTCTTGGAGCTTACTTCATTTCCATTGCTCCTACGAGATTGGAAGTGAAAGGTAGTA
AGGGAAGCAAGAAGTGGGACGATGGATTTGACCATGAAAATGTATCAAAGATCGAGGT
ACTAGGTGGTTTTGAAGGCATACTGTACATCAAAGTTGACTATATCAAGAATGGAAAACT
AGAAACTGGATTAGTCCATGGTCACTCTGGTGGTGATGGTTTTTACAGAAGATGGAGAT
TAACCAGTCAAAGAACGAATATCTAGTATATGTTGAAGGTTACTACGATGATGCTTCTGA
AACCATTCAAGGGCTTCATTTCCAAACTAACCTCAACAATCCTGTTATGATGGGGTATAA
AAAGGGCAGGAAGTTTTTACTTGCATCCAATGGAAATAAGATCATTGGGTTTCATGGATA
TGCCGACAAAAGCCTAAACTCTCTTGGAGCATATTTCAGTACGACCACTCCTAATAAACT
GGAATGCCAAGGTGATCGTAAAGGACTGCCTTGGGATGATGGTTGTAATTATGACGGCGT
AAAAAAGGTGTATGTCGATAGTATAAGTGATATAGATAGTGTCAGGTTTGAGTACGATAA
TGGCGGAAAAGTGGAAAAGACTCCGTACCGGCGTGATGTTACAAATGAGAAGGAGTTT
GTGCTTGACTATCCAAATGAGTTTATCACGTCTGTGGAGGGGACCTTAGCAACTCCGACT
AATTTTGACATTACGTGGATTCTCTCACTGACATTCAAAACATCAAAAGGGAGAACCTCT
CCAACATTTGGATCATCGTCTCCTGGTAGAAAATTTGTGCTGGAGAAGAATGGTAGCGCT
CTTGTTGGGTTCCATGGATATATTGGTCCTGGTTATAATATTAAAGCTCTTGGAGCATATTA
TCGCCCGATTCCTCCTACTCCTGATGTGAAAAGACTAGAAGCACAAGGTGGTGATGGAG
GAGCTTCTTGGGATGATGGTGGTACTTTCAACAGTGTTAGAAAGATCTACATTGGACTAG
GCAAAAACGTTGTAGGCTTTGTCAAGTTTTTGTACTACAAAAACGCTCGTGTTGTCATCG
GAGATGATCATGGCAACAAGACCCTTTCATCTGATCTCCTAGAGTTCTTGTTGGATCCGT
TTGAACATATCATATCAGTGGAGGGTACTTATGATGATACATCTGGAGGTATAACCATGCT
TAGGTTCGAGACCAACTTACAAAAATCTCCATACTTTGGATTTGGTACAACATCAAACTT
CTTGCTTCACAAGGATAATCACCAGATCGTTGGATTCCATGGAAAATCCAGTAACATGCT
TCATCAACTTGGGGTCCACGTTATACCCAACGGTTTTAAATTTATTTAA

>AT5G10180|AT5G10180.1

ATGAAAGAGAGAGATTCAGAGAGTTTTGAATCTCTCTCACATCAAGTTCTCCCAAACAC
TTCAAATTCAACACACATGATCCAGATGGCCATGGCCAACTCAGGTTCATCTGCAGCCG
CACAAGCCGGTCAAGACCAGCCTGACCGGTCAAAGTGGCTGCTTGACTGTCCTGAACC
ACCTAGCCCGTGGCATGAGCTCAAAAGACAAGTCAAAGGCTCTTTCCTAACCAAAGCC
AAAAAGTTCAAGTCACTTCAAAAACAGCCTTTCCCAAAACAAATCCTCTCTGTCCTCCA
AGCCATTTTCCCAATCTTCGGTTGGTGCAGAAACTATAAACTCACCATGTTCAAGAACGA
TCTCATGGCTGGTTTAACCCTCGCTAGCCTCTGCATTCCGCAGAGCATTGGTTATGCAAC
TCTTGCAAAGCTTGATCCTCAATATGGCCTATATACGAGTGTGGTACCACCATTGATATAT
GCATTGATGGGGACATCAAGAGAGATAGCAATCGGACCGGTGGCTGTAGTATCTCTTCTT
ATATCTTCAATGTTGCAGAAACTCATCGATCCAGAAACAGATCCCTTGGGATACAAGAAA
CTGGTCCTAACCACAACCTTCTTCGCCGGGATCTTCCAAGCTTCTTTCGGTTTATTCAGG
TTAGGGTTTCTGGTGGATTTTCTGTCGCACGCAGCCATAGTTGGGTTCATGGGTGGTGCA
GCCATTGTAATTGGACTCCAACAGCTTAAAGGTTTGCTTGGTATCACTAACTTCACCACC
AACACTGACATTGTCTCTGTTCTTCGAGCTGTCTGGAGATCTTGTCAACAACAATGGAG
CCCTCACACTTTCATCCTCGGATGTTCTTTCCTCAGTTTTATCCTTATTACTCGCTTCATCG
GGAAGAAGTATAAGAAGCTGTTTTGGCTACCGGCAATAGCTCCGTTGATCGCCGTGGTA
GTGTCAACACTAATGGTGTTTCTGACTAAAGCCGACGAGCATGGTGTGAAGACAGTGAG
GCACATCAAAGGAGGTCTTAATCCAATGTCCATTCAGGATCTCGACTTTAATACTCCTCAT
CTCGGACAAATCGCTAAAATCGGATTAATCATTGCCATTGTTGCTCTAACCGAGGCGATT
GCGGTGGGGAGGTCGTTCGCCGGAATAAAAGGGTACAGACTCGATGGAAACAAAGAAA
TGGTGGCCATTGGATTTATGAATGTTCTCGGTTCCTTCACATCTTGTTACGCTGCTACTGG
TTCATTCTCTCGGACGGCCGTGAATTTTGCGGCAGGATGTGAGACAGCAATGTCCAACA
TTGTTATGGCGGTTACGGTGTTTGTAGCACTCGAGTGTCTAACGAGGCTTCTCTACTATA
CTCCAATCGCCATCCTCGCTTCAATAATTCTCTCAGCACTTCCGGGACTAATCAACATTAA
CGAGGCTATTCACATTTGGAAAGTCGATAAATTCGATTTTCTTGCTCTCATTGGAGCTTTC

TTTGGTGTTTTGTTCGCTTCCGTTGAGATCGGACTTCTTGTCGCGGTGGTTATTTCGTTTG
CCAAGATCATACTCATATCAATTCGTCCAGGGATAGAAACGCTTGGAAGAATGCCCGGG
ACCGATACTTTTACAGATACTAATCAATATCCTATGACGGTTAAGACTCCCGGAGTGTTGA
TTTTTCGTGTCAAGTCTGCATTGTTGTGCTTTGCCAATGCCAGTTCAATTGAGGAAAGGA
TTATGGGATGGGTCGATGAGGAAGAAGAAGAAGAAAACACAAAGAGCAATGCCAAGA
GAAAGATCCTCTTTGTAGTCCTTGATATGTCAAGTTTGATCAACGTCGATACATCGGGGA
TTACTGCTTTGCTGGAACTGCATAACAAATTAATCAAAACTGGTGTTGAGCTAGTGATCG
TTAACCCGAAATGGCAAGTAATCCACAAGCTGAATCAAGCAAAGTTCGTCGACAGAATC
GGTGGCAAAGTTTACTTGACGATCGGCGAAGCTCTTGATGCTTGCTTTGGATTAAAAGTT
TAA

>AT5G60020|AT5G60020.1

ATGGCGTTACAGCTACTCCTAGCTGTATTCTCTTGTGTTCTTCTTCTTCCTCAACCTGCAT
TTGGGATTACAAGGCATTATACGCTGGAAATCAAAATGCAGAACGTAACACGTCTTTGCC
ACACAAAGAGCCTTGTTTCTGTAAACGGGCAGTTTCCAGGTCCTAAGCTTATTGCTAGA
GAAGGTGACCAGGTTCTGATCAAAGTCGTTAATCAAGTGCCAAACAACATCTCTCTCCA
CTGGCATGGGATCCGGCAATTACGAAGTGGTTGGGCTGATGGTCCAGCCTATATAACCCA
ATGTCCTATTCAGACAGGACAAAGCTATGTTTACAACTATACCATTGTTGGTCAACGAGG
CACTCTGTGGTACCATGCTCACATTTCATGGCTAAGATCAACAGTCTATGGTCCACTTATC
ATCCTTCCCAAACGCGGAGTTCCTTACCCGTTTGCTAAACCTCACAAAGAAGTTCCCAT
GATCTTTGGGGAGTGGTTCAACGCAGACACTGAGGCAATCATCCGCCAAGCAACCCAA
ACAGGAGGTGGTCCCAATGTCTCTGATGCTTACACGATAAACGGGCTTCCTGGTCCATTA
TACAACTGCTCCGCAAAAGATACATTCAGACTGAGAGTGAAGCCAGGAAAAACATACCT
TCTCAGGCTAATCAATGCTGCACTTAATGACGAGCTCTTTTTCAGCATCGCAAATCACAC
GGTTACGGTTGTTGAAGCTGATGCGATCTATGTTAAGCCATTTGAGACTGAAACCATCTT
AATTGCTCCTGGTCAGACCACAAACGTCCTGCTGAAGACTAAATCTAGTTATCCGAGTG
CCTCCTTCTTCATGACTGCTAGACCATACGTCACAGGTCAAGGAACTTTTGATAACTCTA
CAGTTGCTGGAATCTTAGAATATGAACCACCTAAACAGACCAAAGGTGCTCACTCAAGG
ACCTCTATCAAAAATCTTCAACTCTTCAAACCGATACTCCCTGCTCTAAACGATACAAAT
TTTGCTACCAAGTTCAGTAATAAGCTACGCAGCCTGAACAGCAAAAACTTTCCAGCAAA
CGTGCCTCTGAATGTTGATCGGAAGTTCTTCTTCACAGTAGGACTGGGAACAAACCCGT
GCAATCATAAGAATAACCAGACATGCCAAGGTCCTACTAACACCACAATGTTTGCTGCTT
CAATCAGTAACATTTCATTCACAATGCCAACAAAAGCTCTCCTTCAATCTCACTATTCTG
GGCAATCTCATGGAGTGTATTCCCCAAAATTCCCATGGAGTCCCATTGTCCCTTTTAACTA
CACAGGCACTCCACCTAACAATACTATGGTTAGTAACGGGACAAACTTGATGGTTCTACC
TTATAACACCAGTGTGGAGTTGGTGATGCAAGACACTAGCATTCTTGGCGCAGAAAGCC
ATCCTCTTCATCTTCATGGGTTCAACTTCTTTGTTGTTGGCCAAGGGTTTGGGAATTTCG
ACCCGAACAAGGATCCTAGAAACTTCAACCTTGTTGACCCAATAGAGAGGAACACAGT
CGGTGTGCCATCTGGTGGATGGGCTGCTATTCGATTCCTTGCAGATAACCCAGGAGTGTG
GTTCATGCACTGTCACTTGGAAGTGCATACCAGTTGGGGTCTGAGGATGGCTTGGCTTG
TTCTTGATGGAGATAAACCTGATCAGAAACTTCTTCCTCCTCCTGCAGACTTGCCCAAAT
GCTGA

>AT3G15170|AT3G15170.1

ATGGATGTTGATGTGTTTAACGGTTGGGGGAGGCCAAGATTTGAAGATGAATCCCTTATG
CCACCTGGGTTTAGGTTTCATCCAACTGATGAAGAGCTGATCACTTACTATCTCCTCAAG
AAGGTTCTTGACTCTAATTTCTCTTGTGCCGCCATTTCTCAAGTTGATCTCAACAAGTCT
GAGCCTTGGGAGCTTCCTGAGAAAGCGAAAATGGGGGAGAAGGAGTGGTACTTCTTCA
CACTAAGAGACCGTAAATACCCAACGGGACTGAGAACGAACAGAGCAACAGAAGCTG
GTTACTGGAAAGCCACTGGTAAAGACAGAGAGATCAAAAGCTCAAAGACAAAATCACT

TCTCGGGATGAAGAAAACTCTTGTCTTTTACAAAGGCAGAGCTCCTAAAGGAGAGAAG
AGTTGTTGGGTCATGCATGAGTATCGCCTTGACGGCAAATTCTCTTACCATTACATTTCCT
CCTCCGCTAAGGATGAATGGGTTCTCTGTAAAGTTTGTCTGAAAAGCGGCGTAGTTAGTA
GAGAGACGAACTTGATCTCTTCTTCTTCTTCTTCTGCCGTCACCGGAGAGTTCTCCTCTG
CCGGTTCTGCAATTGCTCCGATCATCAATACCTTTGCGACGGAGCACGTGTCCTGTTTCT
CCAATAACTCTGCTGCTCATACCGATGCGAGCTTTCATACATTCCTTCCCGCTCCACCGCC
GTCACTGCCCCCACGTCAGCCACGTCACGTCGGTGATGGCGTGGCGTTTGGTCAGTTTC
TGGATTTGGGATCATCGGGACAGATTGATTTCGATGCAGCAGCAGCAGCGTTCTTTCCGA
ATCTACCTTCTCTGCCTCCCACGGTTCTTCCTCCTCCTCCGTCATTTGCAATGTACGGTGG
AGGCTCCCCCGCCGTGAGTGTGTGGCCGTTTACTCTCTGA

>AT5G55930|AT5G55930.1

ATGACGAGCGTTTTCGACGAACATAAACCCTCCGATGACTCTCACGAGTCCAAGATCGT
CATCAATGGAGAAGAAGAGGTGTTGGAGGAGGAGAATGATAATCCGATTGAAGAAGTC
CGGTTAACGGTACCGATAACCGACGATCCAACACTACCGGTGCTAACGTTTCGAACATG
GACTCTTGGACTTTTCTCTTGCATATTGCTTGCCTTCGTGAACCAGTTCTTTGGTTTCCGG
TCGAACCAGCTTTGGGTATCTTCAGTTGCGGCACAGATCGTGACCCTTCCGCTAGGGAA
ACTCATGGCAAAGACGCTACCGACAAAGAAATTTGGGTTTCCCGGGACGAATTGGTCGT
GGTCTTTTAACCCTGGTCCGTTCAATATGAAGGAACATGTTCTTATTACAATATTTGCTAA
CACCGGAGCTGGTGGAGTTTACGCCACCAGTATTATTACCATTGTTAAAGCGTTTTATAAT
CGTCAACTTAATGTCGCCGCCGCCATGTTGCTCACTCAAACCACTCAGTTGTTGGGATAT
GGATGGGCTGGAATCTTCAGGAAGTTCCTTGTGGATTCACCGTATATGTGGTGGCCGTCA
AATTTGGTTCAAGTCTCACTCTTTAGAGCTTTACATGAGAAGGAGGATCTACAAAAAGG
GCAACAAACAAGGTTTAGATTCTTCATCATTGTCTTCTGTGTGAGCTTTGCTTACTACATA
ATCCCCGGCTACCTTTTCCCTTCCATTTCCGCAATTTCATTTGTTTGCTGGATTTGGAAAA
GCTCAGTGACCGCTCAGATTGTCGGTTCTGGGCTCAAGGGTTTAGGTATTGGTTCGTTCG
GTCTTGACTGGTCCACAGTTGCTGGTTTCTTAGGCAGTCCGTTGGCTGTACCATTCTTCG
CCATTGCCAATTTCTTTGGAGGATTCTTCATCTTTTTATACATTGTTCTACCTATCTTCTACT
GGACTAATGCTTATGATGCTCAGAAGTTTCCGTTCTATACTTCACACACCTTTGATCAGAC
TGGACACACCTATAACATTACCCGTATCCTCAATGAGAAGAATTTCGATATCAATCTAGAC
GCTTACAACGGTTACAGCAAGCTCTATTTGAGTGTGATGTTCGCGTTGCTCTATGGACTC
AGCTTTGGTTCTCTTTGCGCTACCATCTCTCATGTTGCCCTCTATGATGGAAAGTTTATAT
GGGGAATGTGGAAGAAGGCAAAGACAGCAACAAAGGACAAGTACGGTGACGTGCATT
CGAGATTGATGAAGAAGAATTATCAATCAGTACCTCAATGGTGGTTCATCGCAGTTCTAG
TTATTTCCTTTGCTTTCGCACTCTATGCTTGCGAGGGCTTTGACAAACAGCTTCAGCTCC
CATGGTGGGGACTTATACTCGCTTGCGCCATCGCTTTGTTCTTCACATTACCTATCGGAGT
TATCCAAGCAACTACTAACCAGCAAATGGGTCTTAACGTTATAACAGAACTGATCATCGG
GTACTTATACCCAGGAAAGCCACTAGCCAATGTCGCTTTCAAGACATACGGATACATCAG
TATGTCTCAAGCCTTGTACTTTGTAGGAGACTTCAAGCTTGGTCACTACATGAAGATTCC
TCCAAGATCAATGTTCATCGTCCAGCTTGTTGCAACTGTGGTTGCATCAACTGTCTGCTT
CGGAACAACCTGGTGGCTCATTACATCCGTCGAGAACATATGTAATGTCGATTTGCTCCC
GGTGGGTAGTCCATGGACTTGTCCTGGAGATGAAGTGTTCTACAATGCATCAATCATATG
GGGAGTGATTGGTCCAGGGAGAATGTTTACCAAAGAAGGTATCTATCCCGGGATGAACT
GGTTCTTCCTTATCGGTCTCCTCGCTCCAGTTCCCTTCTGGTACCTATCGAAGAAGTTCCC
AGAGAAGAAATGGCTAAAACAGATCCATGTTCCCTTGATCTTCTCTGCAGTAAGCGCCA
TGCCACAAGCTAAGGCTGTGCATTACTGGTCCTGGGCCATCGTTGGGGTTGTGTTCAACT
ACTACATCTTCAGGAGGTTCAAAACTTGGTGGGCGAGGCACAATTACATCCTCTCTGCG
GCGCTTGATGCAGGTACTGCGATTATGGGAGTGTTGATATTCTTCGCATTCCAGAACAAT
GATATAAGCTTACCTGATTGGTGGGGGCTTGAGAATTCAGACCATTGCCCTCTAGCGCAT
TGCCCTCTAGCCAAAGGTGTTGTTGTTGAAGGTTGTCCCGTGTTTTAA

>AT2G45480|AT2G45480.1

ATGCAGAGCCCTAAAATGGAGCAGGAGGAGGTTGAGGAGGAGAGGATGAGGAATAAGT
GGCCGTGGATGAAGGCGGCGCAGTTAATGGAGTTTCGGATGCAAGCTTTGGTGTATAGA
TACATAGAGGCTGGTCTCCGTGTGCCTCATCATCTCGTGGTGCCTATTTGGAACAGTCTT
GCTCTCTCTTCTTCCTCCAATTACAACTATCACTCTTCTTCTCTGTTGAGTAACAAGGGAG
TAACCCATATCGACACGTTGGAAACTGAACCAACTAGGTGCAGGAGAACAGATGGGAA
GAAATGGCGCTGTAGCAACACGGTCCTTCTATTCGAGAAGTACTGTGAACGGCACATGC
ATAGAGGTCGTAAACGTTCAAGAAAGCTTGTGGAATCTTCTTCTGAGGTTGCTTCATCAT
CAACCAAATACGACAACACTTATGGTTTGGATAGGTATAACGAGAGTCAGAGTCATCTTC
ATGGGACAATCTCGGGTTCTAGTAATGCGCAGGTAGTTACCATTGCTTCACTGCCTAGTG
CCAGATCCTGTGAAAATGTCATTCGTCCGTCTTTAGTGATCTCTGAATTCACAAACAAAA
GTGTGAGTCACGGCAGAAAGAACATGGAGATGAGTTATGATGACTTTATTAATGAAAAA
GAGGCGAGTATGTGTGTTGGAGTTGTTCCTCTTCAAGGTGATGAGAGCAAACCTTCGGT
TCAAAAGTTCTTCCCTGAGGTATCTGATAAATGCTTAGAAGCTGCAAAATTCTCAAGCAA
CAGGAAGAATGATATAATTGCAAGAAGCAGAGAATGGAAGAATATGAATGTTAATGGTG
GTTTGTTTCATGGTATCCACTTTTCTCCAGACACTGTTCTTCAAGAACGTGGTTGTTTTCG
TTTACAAGGAGTTGAAACAGACAATGAACCAGGAAGGTGCCGAAGAACAGATGGGAA
GAAGTGGAGATGCAGCAAAGATGTTTTGTCTGGTCAGAAGTACTGCGATAAGCACATGC
ATAGAGGTATGAAGAAGAAGCATCCAGTTGATACTACTAACTCACATGAGAATGCCGGG
TTTAGCCCGTTAACCGTGGAAACAGCTGTTAGATCGGTTGTGCCTTGCAAAGATGGAGA
TGACCAGAAGCATTCTGTTTCAGTCATGGGAATTACACTGCCCCGAGTTTCTGATGAGA
AGAGCACTAGCAGTTGCAGTACCGACACTACCATTACTGACACAGCTTTAAGGGGTGAA
GACGACGATGAGGAGTACTTGTCTTTGTTTTCACCAGGTGTTTAG

>AT5G41610|AT5G41610.2

ATGCTTGGACCTTCTCTTCTTGGTCGTTCCAAGGCTTTTCTAGACGCTGTGTTCCCGAAG
AAAAGCTTGACTGTTCTTGAAACTCTGGCTAATCTCGGCCTTCTCTTCTTCCTTTTCCTTG
CTGGTCTAGAGATTGATACCAAAGCCCTTCGTCGCACCGGGAAAAAGGCTTTGGGAATT
GCCTTGGCTGGAATAACCCTCCCGTTTGCTCTTGGCATTGGTTCTTCATTCGTTTTAAAA
GCAACAATCTCCAAAGGAGTGAATAGTACCGCGTTTCTCGTCTTCATGGGTGTGGCACT
CTCTATCACTGCATTCCCTGTGTTGGCCCGTATCTTAGCGGAGCTAAAGCTTCTAACTACT
GAGATAGGACGACTTGCTATGTCGGCAGCTGCCGTTAACGATGTGGCAGCTTGGATTCTA
CTTGCTCTAGCCATTGCTCTCTCGGGATCCAATACCTCTCCCCTTGTCTCTCTCTGGGTTT
TCCTTTCCGGGTGTGCTTTTGTTATCGGTGCGTCTTTCATCATTCCACCAATCTTTAGATG
GATTTCCCGGAGGTGCCACGAGGGAGAACCTATCGAGGAAACCTACATCTGTGCCACTT
TGGCTGTAGTTCTTGTTTGTGGATTCATAACTGATGCCATTGGAATCCACTCCATGTTTGG
TGCCTTTGTGGTGGGTGTTTTGATTCCAAAGGAAGGACCTTTTGCTGGTGCACTAGTTG
AGAAGGTGGAAGATCTTGTATCCGGTCTCTTCCTGCCGCTTTACTTTGTGGCAAGTGGTC
TGAAAACTAATGTGGCAACGATCCAAGGAGCTCAGTCTTGGGGGTCTTCTGGTTTTAGTC
ACCGCCACAGCTTGTTTCGGTAAGATATTGGGCACTCTCGGTGTTTCCCTCGCCTTCAAG
ATACCTATGCGAGAAGCAATAACATTAGGATTCCTCATGAACACTAAAGGATTGGTCGAA
CTCATCGTCCTCAACATCGGAAAAGATCGAAAGGTTCTTAATGATCAGACATTTGCCATA
ATGGTTCTAATGGCTCTCTTCACAACCTTCATCACCACACCAGTCGTAATGGCAGTCTAC
AAACCAGCGAGAAGAGCGAAGAAAGAAGGAGAATACAAACACAGGGCAGTAGAACG
AGAGAACACAAACACACAGCTTCGAATCCTCACATGTTTCCACGGAGCAGGAAGCATT
CCTTCAATGATAAACCTCCTAGAAGCATCAAGAGGTATCGAAAAAGGCGAAGGGCTCTG
CGTTTACGCTCTCCACTTACGGGAACTATCAGAGAGATCATCAGCTATTCTAATGGTTCAT
AAAGTTCGGAAAAACGGAATGCCGTTTTGGAACAGAAGAGGAGTAAACGCAGACGCT
GATCAAGTAGTAGTTGCTTTCCAAGCTTTTCAACAGCTAAGCAGAGTCAATGTCCGACC
AATGACAGCAATCTCTTCAATGTCTGATATTCATGAAGATATCTGCACAACAGCTGTAAG

AAAAAAAGCAGCCATTGTGATTCTTCCGTTTCACAAGCATCAACAGCTTGATGGTTCGTT
AGAGACTACACGTGGCGATTACCGTTGGGTTAACCGGAGAGTTTTGCTTCAAGCTCCTT
GTTCGGTTGGTATATTCGTTGACCGTGGACTCGGAGGTTCAAGTCAAGTCTCAGCTCAA
GATGTTTCTTACTCTGTTGTTGTTCTGTTCTTCGGTGGTCCTGATGATCGTGAAGCGTTGG
CTTATGGATTACGTATGGCGGAGCATCCCGGAATTGTTTTGACTGTTTTCCGATTTGTGGT
ATCGCCGGAGAGAGTCGGAGAGATTGTTAATGTTGAAGTGAGTAACAATAACAACGAG
AATCAGAGTGTGAAGAATCTGAAATCGGATGAGGAGATTATGTCTGAGATAAGGAAGAT
ATCTTCGGTGGACGAATCGGTTAAGTTCGTGGAGAAACAGATTGAAAACGCCGCCGTGG
ATGTTAGATCGGCGATTGAGGAAGTGCGGCGGAGCAATTTGTTTTTGGTTGGTAGAATG
CCAGGTGGAGAAATTGCATTGGCGATTAGGGAGAATAGTGAATGTCCGGAGCTTGGACC
TGTAGGAAGCTTGTTGATTTCGCCGGAATCTTCGACAAAAGCATCGGTTTTGGTGATTCA
GCAGTACAACGGCACCGGAATAGCTCCGGATTTGGGTGCGGCTGAGACGGAGGTATTGA
CTTCTACGGATAAAGACTCTGATTGA

>AT4G24150|AT4G24150.1

ATGAGGATGCTTCTTGGGATTCCTTACGTAGACAAGTCGGTTCTTTCCAACTCTGTTCTT
GAGAGAGGCAAGCAGGATAAAAGCAAACTATTGTTAGTCGACAAATGCCATTATGAGCT
TGATGTTGAAGAACGCAAGGAAGATTTTGTTGGTGGGTTTGGATTTGGTGTTGTAGAAA
ATTCGCATAAAGACGTTATGGTGCTACCTCATCATCACTATTATCCATCATATTCATCACCT
TCCTCTTCTTCTTTGTGTTACTGTTCTGCTGGTGTTAGCGATCCCATGTTCTCTGTTTCTAG
CAATCAGGCTTACACTTCTTCTCACAGTGGTATGTTCACACCCGCCGGTTCTGGTTCTGC
TGCTGTGACTGTAGCAGATCCTTTTTTCTCCTTGAGCTCTTCAGGGGAAATGAGAAGAA
GTATGAACGAAGATGCTGGTGCAGCTTTCAGCGAAGCTCAATGGCATGAGCTTGAGAGG
CAGAGGAATATATACAAGTACATGATGGCTTCTGTTCCTGTTCCTCCAGAGCTTCTCACA
CCCTTTCCCAAGAACCACCAATCAAACACTAACCCGGATGTGGATACATATAGGAGTGG
AATGTTTAGTATTTATGCTGATTACAAGAATCTGCCGTTGTCTATGTGGATGACAGTAACT
GTGGCAGTGGCGACAGGAGGCTCATTGCAGCTGGGGATTGCTTCAAGCGCAAGCAATA
ACACGGCTGATCTGGAGCCATGGAGGTGCAAGAGAACAGATGGGAAGAAATGGAGGTG
CTCTAGAAACGTGATTCCTGATCAGAAATACTGTGAGAGACACACACACAAGAGCCGTC
CTCGTTCAAGAAAGCATGTGGAATCATCTCACCAATCATCTCACCACAATGACATTCGTA
CGGCTAAGAATGATACTAGCCAGCTTGTGAGAACTTATCCTCAGTTTTACGGACAACCTA
TAAGCCAGATCCCTGTGCTTTCTACTCTTCCGTCTGCCTCCTCTCCATATGATCACCACAG
AGGACTGAGGTGGTTTACGAAAGAAGATGATGCCATTGGAACCTTAAACCCGGAGACT
CAAGAAGCTGTCCAGCTGAAAGTTGGATCAAGCAGAGAGCTCAAACGGGGATTCGATT
ATGATCTGAATTTCAGGCAGAAAGAGCCAATAGTAGACCAGAGCTTTGGAGCATTGCAG
GGTCTATTAAGTCTAAACCAGACACCACAACATAACCAAGAAACAAGACAGTTTGTTGT
AGAAGGAAAGCAAGATGAAGCGATGGGAAGCTCTCTGACACTCTCAATGGCTGGAGGA
GGCATGGAGGAAACAGAGGGAACAAACCAGCATCAGTGGGTTAGCCATGAAGGTCCAT
CATGGCTCTATTCAACAACACCAGGTGGACCATTGGCTGAAGCACTGTGTCTCGGTGTC
TCCAACAACCCAAGTTCTAGTACTACTACTAGTAGCTGCAGCAGAAGCTCAAGCTAA

>AT4G32880|AT4G32880.1

ATGGGAGGAGGAAGCAATAATAGTCACAATATGGACAACGGGAAGTACGTGAGGTACA
CTCCTGAACAAGTGGAAGCTCTGGAGAGACTCTACAATGACTGTCCTAAACCGAGCTCT
ATGCGCCGCCAACAGCTAATCCGCGAATGTCCTATCCTCTCCAACATCGAGCCTAAACAG
ATAAAAGTCTGGTTCCAGAACCGCAGGTGTAGAGAAAAACAGCGAAAAGAGGCGTCAC
GACTTCAAGCTGTGAATAGGAAGCTAACGGCAATGAACAAGCTTTTGATGGAAGAGAAT
GACCGCTTGCAAAAGCAAGTGTCTCACTTGGTTTATGAGAACAGCTATTTTCGCCAACA
TCCTCAAAACCAGGGGAATTTGGCTACCACAGATACTAGCTGTGAGTCAGTGGTGACGA
GTGGTCAACACCACTTGACCCCTCAACATCAGCCTCGTGATGCTAGTCCTGCTGGATTAT

TGTCCATTGCGGATGAAACTTTAACAGAGTTCATTTCCAAGGCGACTGGAACCGCTGTC
GAGTGGGTCCAAATGCCTGGGATGAAGCCTGGTCCGGATTCCATAGGAATCGTTGCTATT
TCTCATGGATGCACGGGAATCGCTGCTCGTGCTTGCGGCCTTGTGGGTCTTGATCCCACA
AGGGTCGCGGAGATCCTAAAGGATAAGCCTTGTTGGTTGCGTGATTGCAGATCTCTGGAT
ATAGTTAACGTACTATCCACTGCAAATGGTGGAACCCTTGAACTAATCTACATGCAGCTT
TATGCGCCGACAACACTGGCACCAGCTCGTGACTTCTGGATGCTACGTTACACATCTGTA
ATGGAAGACGGGAGCCTTGTGATATGCGAACGATCACTGAACAATACACAAAACGGGC
CAAGTATGCCTCCGTCTCCTCATTTCGTTAGGGCAGAGATTTTACCAAGTGGTTACCTCA
TTAGACCTTGCGAAGGAGGTGGATCCATTCTTCACATTGTCGATCATTTCGATCTTGAGC
CATGGAGTGTGCCAGAAGTTCTTCGTTCTCTCTATGAGTCCTCCACTTTACTCGCCCAAA
GAACTACAATGGCCGCTCTGCGCTATTTGAGGCAAATATCTCAAGAGATTTCACAACCTA
ACGTAACAGGTTGGGGAAGAAGACCAGCGGCTCTTAGAGCACTTAGCCAAAGGCTTAG
CAAAGGATTCAACGAAGCAGTGAATGGGTTCAGCGATGAAGGATGGTCCATCCTAGAG
AGTGATGGTATCGATGATGTCACTCTTCTTGTGAACTCCTCTCCCACAAAGATGATGATG
ACTTCAAGTCTCCCATTTGCCAATGGCTACACTTCTATGCCTAGCGCGGTCTTATGTGCCA
AAGCTTCCATGTTATTACAAAATGTTCCACCTTCGATTCTGCTGCGGTTCTTGAGGGAAC
ATAGACAAGAATGGGCAGACAATAGCATCGATGCATATTCGGCCGCAGCCATCAAAGCA
GGCCCTTGTAGCTTACCAATCCCTCGCCCAGGGAGCTTTGGTGGTCAAGTCATTCTTCCT
CTAGCTCACACTATAGAGAATGAAGAGTTTATGGAAGTCATTAAGCTTGAGAGCTTGGG
GCACTACCAAGAAGACATGATGATGCCTGCTGATATCTTCCTTCTGCAAATGTGCAGTGG
GGTGGATGAGAACGCAGTTGAATCATGCGCAGAGCTTATATTTGCACCAATCGACGCATC
TTTCTCTGATGATGCACCAATCATTCCTTCCGGTTTCCGCATCATTCCTCTAGATTCCAAA
TCAGAGGGGTTGAGTCCTAACCGAACGCTAGACCTAGCGTCGGCTCTAGACGTAGGGA
GCAGAACAGCCGGAGATTCATGTGGAAGCAGAGGAAACTCAAAGTCCGTTATGACTATA
GCGTTTCAGCTAGCTTTTGAGATGCATATGCAAGAGAATGTAGCCTCAATGGCTAGACAG
TATGTAAGAAGTGTGATCGCGTCGGTTCAACGGGTCGCACTTGCTCTCTCTCCTTCTTCT
CATCAGCTAAGTGGCTTGCGTCCTCCACCCGCGTCACCCGAAGCTCACACTCTCGCTCG
CTGGATCTCTCACTCTTATAGATGTTACCTTGGCGTTGATCTTCTCAAACCTCATGGAACT
GATCTTCTCAAGTCTCTTTGGCACCATCCTGACGCTGTCATGTGTTGCTCACTCAAGGCC
TTATCTCCGGTATTCACATTTGCGAACCAAGCTGGTTTAGACATGCTGGAGACGACGTTG
GTGGCACTTCAAGATATCACTCTCGACAAGATCTTCGACAACAACAACGGGAAGAAGA
CTTTATCCTCCGAATTCCCTCAGATCATGCAACAGGGGTTTATGTGTATGGATGGAGGAAT
ATGCATGTCGAGCATGGGAAGAGCAGTAACGTACGAGAAGGCTGTTGGGTGGAAAGTG
TTGAACGACGACGAAGATCCTCATTGTATCTGCTTCATGTTCCTCAACTGGTCTTTTATAT
GA

>AT4G37740|AT4G37740.1

ATGGATATTGGTGTTCATGTTCTTGGGTCGGTTACTAGTAATGAAAATGAGTCACTTGGTC
TAAAAGAGCTTATAGGAACTAAACAAGATAGATCCGGATTCATCGGTGAGGATTGCTTGC
AACGAAGCTTGAAGCTAGCAAGAACGACAACTAGAGCGGAAGAAGAAGAAAACTTGT
CTTCTTCTGTTGCAGCTGCTTATTGCAAAACGATGTCGTTTCACCAAGGCATTCCTCTCAT
GAGATCTGCTTCTCCTCTTTCCTCTGATTCTCGCCGTCAAGAACAAATGCTTAGCTTCTC
AGATAAACCAGACGCTCTTGATTTCAGTAAATATGTCGGTTTGGATAATAGCAGTAATAA
CAAGAACTCTCTCTCGCCGTTTCTTCACCAGATTCCTCCACCTTCTTACTTTAGAAGCTC
AGGAGGATATGGTTCTGGTGGAATGATGATGAACATGAGCATGCAAGGGAACTTCACAG
GTGTTAAAGGACCTTTTACATTGACTCAATGGGCTGAGTTAGAGCAACAGGCGTTGATCT
ATAAGTACATCACAGCCAATGTCCCTGTTCCTTCTAGTTTGCTCATCTCTATCAAGAAGTC
TTTTTATCCTTACGGATCTTTGCCTCCTAGTTCCTTCGGATGGGGAACTTTCCATCTCGGT
TTCGCAGGCGGTAACATGGACCCTGAGCCAGGGAGATGCCGCAGAACAGATGGGAAGA
AATGGCGGTGCTCAAGAGACGCCGTTCCTGATCAGAAATACTGTGAAAGACACATCAAC
AGAGGCCGTCATCGTTCAAGAAAGCCTGTGGAAGTCCAATCTGGCCAAAACCAAACCG

CCGCTGCTGCATCCAAAGCGGTTACTACACCACAACAGCCTGTTGTCGCTGGTAATACTA
ACAGAAGCAATGCCCGTGCATCAAGCAACCGCAGCCTCGCCATTGGAAGTCAATATATC
AATCCTTCTACAGAATCTTTACCTAACAACAGAGGAGTTTCGATATATCCTTCCACCGTCA
ACTTACAACCCAAGGAATCTCCGGTTATTCATCAGAAACACAGAAACAACAACAACCCT
TTTGAGTTTGGACACATATCCTCTGATTCGTTACTCAACCCGAATACCGCAAAGACCTAT
GGATCATCGTTCTTGGATTTCAGCAGCAACCAAGAGAAGCATTCAGGGAATCACAATCA
CAATTCTTGGCCTGAAGAGCTGACATCAGATTGGACACAGCTCTCAATGTCAATTCCAAT
AGCATCATCATCCCCTTCCTCCACACACAACAACAACAATGCTCAAGAAAAAACAACAC
TCTCGCCTCTCAGGCTATCCCGCGAGCTTGACCTATCGATCCAAACCGATGAAACAACAA
TCGAGCCTACTGTGAAAAAGGTGAATACTTGGATACCAATCTCATGGGGAAACTCCTTA
GGAGGTCCTCTAGGTGAAGTACTAAACAGTACAACGAATAGTCCAACATTTGGATCTTCT
CCTACAGGGGTTTTGCAAAAGTCCACATTTTGTTCACTCTCTAACAACAGCTCCGTGAG
CAGCCCCATTGCAGAGAACAACAGACACAATGGCGATTACTTTCATTACACAACCTGA

>AT3G23690|AT3G23690.1

ATGAACATGGACAAGGAAACAGAGCAAACCCTAAATTACTTACCTTTGGGTCAGAGCGA
TCCCTTTGGCAATGGCAATGAAGGAACAATTGGAGATTTCCTTGGAAGATACTGCAACA
ACCCTCAGGAGATTTCACCGTTAACTCTACAATCCTTCTCTCTGAATTCTCAGATCTCCG
AGAATTTCCCAATCTCTGGTGGAATCAGATTCCCTCCATATCCAGGTCAATTTGGATCCGA
TCGTGAATTTGGGTCACAACCAACAACGCAGGAGAGTAACAAGAGCTCTTTGTTGGATC
CAGATTCAGTTTCGGATCGAGTTCACACCACGAAATCTAACTCTAGAAAGAGGAAGTCG
ATTCCTAGTGGTAATGGCAAGGAGTCTCCAGCTTCATCGTCTCTTACAGCTTCCAATTCA
AAGGTTTCAGGAGAGAATGGTGGATCTAAAGGTGGGAAGAGAAGCAAGCAAGATGTAG
CTGGGAGTAGTAAAAACGGAGTAGAGAAGTGCGATAGTAAAGGCGACAATAAGGACGA
TGCTAAGCCTCCTGAGGCGCCTAAAGATTATATTCATGTCAGAGCTAGAAGGGGTCAAG
CAACTGATAGCCATAGTCTTGCTGAAAGAGCAAGAAGAGAAAAGATTAGTGAGAGAAT
GACGTTGCTTCAGGATCTGGTTCCTGGTTGCAACCGGATTACAGGGAAAGCAGTCATGC
TTGATGAAATTATAAATTATGTGCAGTCCTTGCAGAGACAAGTTGAGTTCTTGTCCATGA
AGCTGGCTACCGTAAATCCAAGGATGGAGTTTAATGCTAATGCTTCTTTATCCACAGAGA
TGATTCAACCGGGGGAGTCGTTAACGCAGTCTCTTTACGCAATGGCTTGCTCAGAGCAA
AGACTTCCATCAGCATACTATTCACTTGGCAAGAACATGCCAAGATTCTCAGACACACAA
TTCCCCTCAAACGATGGATTTGTTCACACTGAGACACCAGGATTCTGGGAAAATAATGA
CCTGCAAAGCATTGTTCAGATGGGTTTTGGAGATATCCTCCAGCAACAGAGCAACAACA
ACAACAACAACTGTTCTGAGCCAACGCTTCAGATGAAGCTTGAACCATAG

>AT5G60120|AT5G60120.1

ATGCTGGATCTCAATCTCGACGTCGACTCGACCGAGTCTACTCAGAACGAACGAGATTC
GATTACTGTTAAAGGGGTTTCTTTGAATCAGATGGATGAATCGGTGACGTCGAACTCTTC
TGTTGTTAATGCTGAAGCTTCTAGCTGTATAGATGGTGAAGATGAGTTGTGCTCTACACG
AACGGTCAAGTTTCAATTTGAGATACTGAAAGGAGGAGGAGAAGAAGAAGAAGAAGAT
GATGATGAAAGAAGTGCTGTGATGATGACTAAGGAGTTTTTTCCTGTTGCTAAAGGCAT
GAACTTTATGGATTCGAGCGCTCAAAGTTCAAGGAGCACCGTTGATATTTCCTTCCAGAG
GGGGAAACAAGGTGGAGACTTTATAGGTAGCGGGAGCGGTGGTGGCGATGCTTCACGG
GTGATGCAGCCACCATCACAGCCGGTGAAGAAAAGCAGGAGAGGTCCTAGGTCTAAGA
GTTCTCAGTATAGAGGCGTCACTTTCTATCGGAGGACAGGCAGATGGGAATCGCATATCT
GGGATTGCGGTAAACAAGTTTATTTAGGTGGATTTGATACTGCCCATGCTGCAGCTAGGG
CGTATGATCGAGCTGCTGTCAAATTCCGGGGTCTGGAAGCTGACATCAATTTCGTTATTG
GCGATTATGAAGAGGATCTCAAGCAGATGGCGAATCTTTCCAAAGAGGAAGTTGTGCAA
GTACTTCGGCGACAGAGCTCTGGTTTCTCAAGGAATAATTCTAGATATCAAGGAGTTGCT
TTGCAAAAGATTGGCGGGTGGGGAGCTCAAATGGAGCAGCTTCATGGAAACATGGGTT

GTGACAAGGCAGCCGTACAATGGAAGGGAAGGGAAGCTGCTTCCTTAATTGAGCCTCAT
GCATCCCGAATGATTCCCGAGGCAGCTAATGTTAAGCTCGACCTCAACTTGGGAATCTCT
CTTTCACTAGGAGATGGTCCGAAGCAAAAAGATAGGGCCCTCCGGCTTCACCATGTCCC
TAACAATTCCGTATGTGGAAGGAACACCATGATGGAGAACCACATGGCTGCAGCGGCAT
GTGATACGCCTTTCAATTTCTTGAAGAGGGGTTCAGACCATCTTAATAACCGGCATGCCC
TTCCTTCTGCGTTCTTTTCACCCATGGAAAGAACACCAGAGAAAGGGCTTATGTTACGTT
CTCATCAAAGCTTCCCAGCCAGGACATGGCAAGGGCATGATCAGTCCAGTGGTGGGACC
GCCGTAGCAGCTACAGCACCGCCACTGTTTTCAAATGCAGCATCATCAGGATTCTCACTC
TCAGCTACACGCCCTCCTTCATCCACAGCTATTCATCATCCTTCTCAGCCCTTCGTCAATC
TGAATCAGCCCGGTCTCTATGTTATCCACCCATCTGATTACATATCTCAGCACCAACACAA
TCTCATGAACCGCCCACAACCACCACCATAG

>AT2G38080|AT2G38080.1

ATGGGGTCTCATATGGTTTGGTTTCTATTTCTTGTATCCTTCTTCTCTGTGTTCCCAGCTCC
ATCTGAGAGCATGGTTCGCCACTACAAGTTTAACGTTGTAATGAAGAACGTGACTAGATT
ATGCTCAAGCAAGCCAACCGTGACCGTCAACGGTAGATATCCAGGTCCCACAATCTACG
CACGAGAAGATGACACGTTGCTCATCAAAGTCGTTAATCACGTCAAGTACAACGTCTCC
ATCCACTGGCACGGTGTGAGACAAGTGAGAACGGGATGGGCTGATGGGCCTGCTTACAT
AACTCAGTGCCCGATCCAGCCTGGTCAAGTCTACACATACAACTACACTTTGACCGGCC
AACGCGGAACGCTCTGGTGGCACGCTCATATCCTCTGGCTCCGAGCCACTGTTTACGGT
GCATTGGTCATCCTTCCCAAACGCGGTGTTCCCTATCCTTTCCCCAAACCCGACAATGAG
AAAGTCATCGTTCTAGGTGAATGGTGGAAATCGGATACTGAAAATATTATTAATGAGGCG
CTTAAGTCTGGATTAGCCCCTAATGTCTCTGACTCTCACATGATCAACGGACACCCAGGC
CCAGTTAGAAACTGTCCATCTCAAGGTTACAAACTGTCAGTAGAGAATGGCAAAACCTA
TCTGCTACGACTAGTCAACGCTGCACTTAATGAAGAACTCTTTTTCAAAGTCGCCGGCCA
TATTTTCACGGTGGTAGAAGTAGACGCAGTCTATGTTAAACCGTTCAAGACCGACACCG
TCCTTATAGCCCCCGGTCAAACCACCAACGTCCTCCTAACCGCCTCAAAATCCGCCGGG
AAATACCTTGTAACCGCTTCTCCTTTCATGGACGCCCCAATCGCGGTGGACAACGTAACC
GCCACCGCAACTGTTCATTACTCGGGAACACTCTCCTCCTCCCCAACAATCCTCACCCTT
CCTCCCCCGCAAAACGCTACTTCCATAGCCAACAACTTCACAAACTCTCTTCGTAGTCTC
AACTCCAAGAAGTACCCTGCTCTTGTCCCGACCACCATCGACCACCACCTCTTCTTCAC
CGTCGGCCTTGGGCTAAACGCATGCCCTACTTGCAAGGCCGGAAACGGAAGCCGTGTC
GTGGCTAGCATCAACAATGTAACCTTCATTATGCCTAAAACCGCTTTGCTCCCGGCTCATT
ACTTCAACACAAGTGGAGTTTTCACGACAGACTTTCCCAAGAATCCACCACACGTTTTC
AACTACAGCGGAGGATCAGTCACGAACATGGCCACAGAAACCGGCACAAGGCTCTACA
AGCTACCGTATAACGCCACTGTTCAGCTTGTCCTTCAAGATACCGGCGTCATAGCGCCAG
AGAACCATCCAGTACATCTTCACGGTTTTAACTTTTTTGAAGTCGGTCGTGGATTAGGTA
ACTTCAACTCCACGAAAGACCCAAAAAACTTCAATTTGGTAGATCCGGTTGAGAGGAA
CACAATCGGAGTTCCATCCGGTGGATGGGTCGTCATCAGATTCAGAGCAGATAATCCCG
GGGTTTGGTTCATGCATTGTCACTTGGAGGTACACACGACGTGGGGATTAAAGATGGCT
TTCTTGGTGGAGAACGGCAAAGGACCCAATCAGTCGATTTTGCCGCCGCCTAAGGATCT
TCCCAAGTGCTAA

>AT3G15270|AT3G15270.1

ATGGAGGGTCAGAGAACACAACGCCGGGGTTACTTGAAAGACAAGGCTACAGTCTCCA
ACCTTGTTGAAGAAGAAATGGAGAATGGCATGGATGGAGAAGAGGAGGATGGAGGAG
ACGAAGACAAAAGGAAGAAGGTGATGGAAAGAGTTAGAGGTCCTAGCACTGACCGTG
TTCCATCGCGACTGTGCCAGGTCGATAGGTGCACTGTTAATTTGACTGAGGCCAAGCAG
TATTACCGCAGACACAGAGTATGTGAAGTACATGCAAAGGCATCTGCTGCGACTGTTGC
AGGGGTCAGGCAACGCTTTTGTCAACAATGCAGCAGGTTTCATGAGCTACCAGAGTTTG

ATGAAGCTAAAAGAAGCTGCAGGAGGCGCTTAGCTGGACACAATGAGAGGAGGAGGA
AGATCTCTGGTGACAGTTTTGGAGAAGGGTCAGGCCGGAGAGGGTTTAGCGGTCAACT
GATCCAGACTCAAGAAAGAAACAGGGTAGACAGGAAACTTCCTATGACCAACTCATCAT
TCAAGCGACCACAGATCAGATAA

>AT2G36400|AT2G36400.1

ATGGATTTGCAACTGAAACAATGGAGAAGCCAGCAGCAGCAACAACATCAGACAGAGT
CAGAAGAACAACCTTCTGCAGCTAAGATACCAAAACATGTCTTTGACCAGATTCATTCT
CACACTGCAACTTCTACTGCTCTTCCTCTCTTTACCCCTGAGCCTACTTCTTCTAAACTCT
CCTCTTTGTCTCCTGATTCTTCCTCCAGGTTCCCCAAGATGGGGAGCTTCTTTAGCTGGG
CACAGTGGCAAGAACTTGAACTACAAGCTCTGATCTACAGGTACATGTTGGCTGGTGCT
GCTGTTCCTCAGGAGCTCCTTTTACCAATCAAGAAAAGCCTTCTCCATCTATCTCCTTCCT
ACTTTCTTCACCATCCTCTTCAACACCTACCTCATTACCAACCTGCTTGGTATTTGGGAAG
GGCAGCGATGGATCCTGAGCCAGGCAGATGCAGGAGAACGGATGGTAAGAAGTGGAGA
TGTTCAAGAGACGTCTTCGCTGGCCACAAGTATTGCGAGCGCCACATGCACCGTGGCCG
CAACCGTTCAAGAAAGCCTGTGGAAACTCCAACCACCGTCAATGCAACTGCCACGTCC
ATGGCTTCATCAGTAGCAGCCGCAGCCACCACTACAACAGCAACAACAACATCTACGTT
TGCTTTTGGTGGTGGTGGTGGTAGTGAGGAAGTGGTTGGTCAAGGAGGATCTTTCTTCT
TCTCTGGCTCTTCTAACTCTTCATCTGAACTTCTCCACCTTAGTCAAAGTTGTTCGGAGA
TGAAGCAAGAAAGCAACAACATGAACAACAAGAGGCCATACGAGTCCCACATCGGATT
CAGTAACAACAGATCAGATGGAGGACACATCCTGAGGCCCTTCTTTGACGATTGGCCTC
GTTCTTCGCTCCAAGAAGCTGACAATAGTTCAAGCCCCATGAGCTCAGCCACTTGTCTC
TCCATCTCCATGCCCGGGAACTCTTCCTCAGACGTCTCTCTGAAGCTGTCCACAGGCAA
CGAAGAGGGAGCCCGGAGCAACAACAATGGGAGAGATCAGCAAAACATGAGCTGGTG
GAGCGGTGGAGGTTCCAACCACCATCATCACAACATGGGCGGACCATTGGCCGAAGCC
CTGAGATCTTCTTCCTCATCTTCCCCAACCAGTGTTCTCCATCAGCTTGGTGTCTCGACA
CAAGCCTTTCATTGA

>AT4G36920|AT4G36920.1

ATGTGGGATCTAAACGACGCACCACACCAAACACAAAGAGAAGAAGAATCTGAAGAGT
TTTGTTATTCTTCACCAAGTAAACGGGTTGGATCTTTCTCTAATTCAAGCTCTTCAGCTGT
TGTTATCGAAGATGGATCCGATGACGATGAACTTAACCGGGTCAGACCCAATAACCCACT
TGTCACCCATCAGTTCTTCCCTGAGATGGATTCTAACGGCGGTGGTGTTGCTTCTGGCTT
TCCTCGGGCTCACTGGTTTGGTGTTAAGTTTTGTCAGTCGGATCTAGCCACCGGATCGTC
CGCGGGTAAAGCTACCAACGTTGCCGCTGCCGTAGTGGAGCCGGCACAGCCGTTGAAA
AAGAGTCGGCGTGGACCAAGATCAAGAAGTTCTCAGTATAGAGGTGTTACGTTTTACCG
GCGTACCGGAAGATGGGAATCTCATATTTGGGACTGTGGGAAACAAGTTTACTTAGGTG
GATTTGACACTGCTCATGCAGCAGCTCGAGCATATGATAGAGCTGCTATTAAATTCCGTG
GAGTAGAAGCGGATATCAATTTCAACATCGACGATTATGATGATGACTTGAAACAGATGA
CTAATTTAACCAAGGAAGAGTTCGTACACGTACTTCGCCGACAAAGCACAGGCTTCCCT
CGAGGAAGTTCGAAGTATAGAGGTGTCACTTTGCATAAGTGTGGTCGTTGGGAAGCTCG
AATGGGTCAATTCTTAGGCAAAAAGTATGTTTATTTGGGTTTGTTCGACACCGAGGTCGA
AGCTGCTAGAGCTTACGATAAAGCTGCAATCAAATGTAACGGCAAAGACGCCGTGACCA
ACTTTGATCCGAGTATTTACGATGAGGAACTCAATGCCGAGTCATCAGGGAATCCTACTA
CTCCACAAGATCACAACCTCGATTTGAGCTTGGGAAATTCGGCTAATTCGAAGCATAAA
AGTCAAGATATGCGGCTCAGGATGAACCAACAACAACAAGATTCTCTCCACTCTAATGA
AGTTCTTGGATTAGGTCAAACCGGAATGCTTAACCATACTCCCAATTCAAACCACCAATT
TCCGGGCAGCAGCAACATTGGTAGCGGAGGCGGATTCTCACTGTTTCCGGCGGCTGAGA
ACCACCGGTTTGATGGTCGGGCCTCGACGAACCAAGTGTTGACAAATGCTGCAGCATCA
TCAGGATTCTCTCCTCATCATCACAATCAGATTTTTAATTCTACTTCTACTCCTCATCAAAA

TTGGCTGCAGACAAATGGCTTCCAACCTCCTCTCATGAGACCTTCTTGA

>AT3G22890|AT3G22890.1

ATGGCTTCAATGGCTGCCGTCTTAAGCAAAACTCCATTCCTCTCTCAACCACTAACCAAA
TCATCTCCAAACTCCGATCTCCCCTTCGCCGCGGTTTCCTTCCCTTCCAAATCCCTACGCC
GCCGCGTAGGATCAATCCGAGCCGGATTAATCGCTCCCGACGGTGGTAAGCTTGTAGAG
CTCATCGTGGAAGAGCCAAAGCGGCGAGAGAAGAAACACGAGGCGGCGGATTTGCCA
CGTGTTGAGCTGACGGCGATTGACTTGCAATGGATGCATGTATTAAGCGAAGGCTGGGC
AAGTCCACTCGGAGGTTTCATGAGAGAATCCGAGTTCCTCCAAACTCTTCATTTTAACTC
GCTACGTCTTGACGACGGCTCCGTCGTTAACATGTCCGTGCCTATTGTTCTCGCTATTGA
CGATGAACAAAAAGCACGTATCGGCGAGTCTACACGTGTCGCTCTTTTCAATTCCGATG
GTAACCCCGTCGCTATCCTCAGCGATATTGAGATTTATAAGCATCCAAAGGAAGAAAGGA
TAGCTAGAACATGGGGTACGACGGCTCCAGGTTTGCCTTACGTAGACGAGGCGATAACT
AATGCTGGAAACTGGCTCATTGGGGGTGATCTTGAGGTTCTTGAGCCAGTGAAGTACAA
TGATGGGCTTGATCGTTTCAGGCTTTCGCCTGCTGAGTTACGTAAAGAGTTGGAGAAGC
GTAATGCGGATGCGGTGTTTGCTTTCCAGCTGAGGAATCCTGTTCATAATGGTCATGCTC
TTCTTATGACTGATACTCGTAGGAGACTTCTTGAGATGGGTTACAAAAACCCTATTCTTTT
GCTTCATCCGTTAGGTGGGTTTACAAAGGCTGATGATGTTCCTTTAGATTGGAGGATGAA
GCAACACGAGAAGGTTCTAGAGGATGGTGTTCTCGATCCGGAGACTACAGTGGTTTCGA
TATTCCCGTCACCTATGCATTACGCTGGTCCAACCGAAGTGCAGTGGCACGCAAAGGCT
AGAATCAATGCTGGTGCTAACTTTTACATTGTGGGTCGTGATCCTGCTGGGATGGGTCAT
CCAGTAGAGAAACGTGATCTTTACGATGCTGATCATGGAAAGAAAGTACTAAGCATGGC
ACCAGGACTCGAACGACTCAACATCCTTCCTTTCAGGGTTGCTGCATATGACAAGACGC
AAGGCAAGATGGCTTTCTTCGATCCCTCGAGGCCTCAAGATTTCTTGTTCATCTCCGGCA
CTAAGATGCGCACATTGGCAAAGAACAACGAAAACCCGCCAGACGGTTTTATGTGCCCA
GGTGGATGGAAAGTTCTGGTGGATTACTATGAGAGCTTGACTCCGGCGGGTAATGGTAG
ACTACCAGAAGTGGTTCCGGTGTAA

>AT3G19890|AT3G19890.1

ATGACTATGATCTCTGATCTTTCGAAGGATTTGGTAGAGGAGATACTCTCTAAGGCTCCG
ATAACTTCTCTTGGAGCCGTGAGATCCACTCACAAACAATGGAACGCCTTATCCAAAGG
TCGGCTGTTGTATAAAGCGGAAGCGAAGGATCAGTTTCTAGGGTTCATGGTGATGGATCA
TAGGTTTTTATCAATGATATTCCATCTCAATGGAATTCTAAAAGGAGACGGTGAAGGCTT
TGATCGTCCATCTATCAGGGAGGTAGGTGATATAGTTAATCAAATCGACATCTCTAAAGTG
TTTCAATGCGATGGTTTAGTGTTGTGCGTTCCAAGTGACAACTCTAGTGTTGTGGTTTGG
AATCCGTATTTGGGTCAAACCAAGTGGATCGAAGCAAGAGAACCTCACGACGAATCAG
ACATGTTTGCTCTCGGTTACGACAAGGACAAGAACCACAAGATCTTGAGGTTGTACGAT
GAGTGTTATTACTACTACGAAGTTTACAATTTCAAGACAGAATCATGGGGGGAAGAAGA
TCACCTTCCAGGCTGGGATATAGATTCTTATAACCGCGGCGTATCCTTAAATGGAAATACG
TACTTCTTGACTCAAGAGCAAAGAGCAAAAGATAAGTATCGTGTTTTCTTGCTTTGTTTT
AACTTCACAACGGAGAAGTTTGAAAATTTTATTGCTATGCCGTTTAAGTATCATAGGAAA
TACGTTGGAACACTTTCTTGTGTTGGAAATGAAAAACTAGCGGCTCTATATCAGCGCTGG
GATACAGGCGAGATGGCCATATGGGTAACCACTAAGATTGAGTCCAATGAGGTGTTGTG
GAGCAACTTGTTTAAAGTTGATATGAAACCACTCGTTAGGTTTGGCTTTCAACAATGTAA
AGATGAAGCTGGCAGTTTTTTCATTGACGAAGAGAAGAAACTCGCGGTGGTTTTTAATC
TAGATAAAAAACGTGGTAAAAGGAATAACAAGACACGTTGTTACCACACAGCTTACATT
ATTGGAGAGAAGGGATACTTGAAAAAAGAGGTTCTTGGAGAAGCTGTGGAGGTCCGAA
AAGACGTGTATCGGAGCGCACTTGTGTGTTCTAGCTCTTATGTTCCAAGTTTAGAGAAGA
TCAACCAAATTGAAGAGGAAGAGGAAGATAAGTGCAAATCAATCAAAATGGTGGACAC
AAAAAGGCAACGTAAGAAGAGGAAGAGGAAGAGCAAAAGGTAA

>AT5G37020|AT5G37020.1

ATGAAGCTGTCAACATCTGGATTGGGTCAACAGGGTCATGAAGGAGAGAAGTGTCTGA
ATTCTGAGCTATGGCATGCTTGTGCTGGACCATTAGTCTCTCTTCCATCATCTGGTAGTCG
AGTTGTTTACTTTCCACAGGGTCACAGTGAACAGGTAGCTGCTACAACTAATAAGGAAG
TTGATGGTCACATACCCAATTACCCAAGCCTACCACCACAATTGATATGCCAGCTCCATAA
TGTTACAATGCATGCAGATGTTGAGACGGATGAAGTCTATGCTCAAATGACACTTCAACC
ATTGACACCGGAGGAGCAGAAGGAAACATTTGTACCGATTGAGTTGGGGATACCGAGTA
AGCAACCTAGTAATTATTTTTGTAAGACTCTCACAGCTAGTGATACCAGTACACATGGAG
GGTTTTCTGTTCCTAGACGTGCTGCTGAGAAAGTGTTTCCTCCATTGGATTACACACTGC
AGCCACCAGCTCAAGAACTGATTGCAAGGGATCTCCATGATGTTGAATGGAAGTTTAGG
CATATCTTTCGGGGACAGCCCAAACGGCATCTCCTAACTACTGGATGGAGTGTCTTTGTC
AGTGCCAAGCGACTAGTAGCTGGAGATTCTGTCATTTTCATCAGGAATGAAAAGAATCA
ACTCTTTTTGGGAATTCGTCATGCCACTCGGCCGCAGACTATTGTACCATCATCTGTTTTA
TCTAGTGATAGCATGCATATTGGACTCCTTGCTGCTGCTGCACATGCTTCTGCAACTAATA
GCTGTTTCACTGTTTTCTTTCATCCAAGGGCTAGCCAATCTGAGTTTGTGATACAACTTTC
CAAGTACATTAAAGCCGTTTTTCACACGCGTATTTCAGTTGGGATGCGCTTTCGCATGCT
CTTCGAGACAGAAGAGTCGAGTGTCCGCAGGTACATGGGTACTATAACTGGTATTAGTG
ATCTAGATTCTGTTCGTTGGCCAAACTCTCATTGGCGATCTGTGAAGGTTGGTTGGGATG
AATCGACTGCAGGGGAGAGACAGCCAAGGGTTTCTTTATGGGAGATTGAGCCTCTGACT
ACCTTTCCTATGTATCCATCTCTTTTTCCTCTCAGACTAAAACGTCCATGGCATGCTGGCA
CATCATCTTTGCCTGATGGAAGGGGTGATTTGGGAAGTGGTCTAACATGGCTAAGAGGG
GGAGGTGGAGAGCAGCAAGGTTTGCTTCCTCTAAATTATCCATCTGTTGGTTTGTTTCCA
TGGATGCAACAAAGGCTGGATCTCAGTCAAATGGGGACTGATAATAATCAGCAATACCA
AGCAATGTTAGCTGCTGGGTTGCAGAACATCGGCGGTGGAGATCCTTTAAGACAGCAGT
TTGTACAGCTGCAAGAGCCTCACCACCAATATCTTCAACAATCAGCTTCCCATAATTCTG
ATTTGATGCTTCAGCAGCAACAGCAGCAACAAGCGTCACGCCATCTCATGCATGCTCAA
ACACAGATTATGAGTGAGAATCTTCCGCAGCAGAATATGCGACAAGAAGTTAGTAACCA
ACCAGCTGGACAGCAGCAACAGCTACAGCAACCGGACCAAAATGCATATCTTAATGCTT
TCAAAATGCAAAATGGCCATCTTCAACAGTGGCAGCAGCAATCAGAGATGCCATCTCCC
TCGTTCATGAAGTCAGATTTTACTGACTCAAGCAACAAATTTGCAACAACTGCTAGTCC
GGCTTCTGGAGATGGCAATCTTTTGAATTTTTCTATAACCGGTCAGTCTGTACTCCCTGA
GCAGTTAACAACAGAGGGCTGGTCTCCAAAAGCATCCAACACTTTTTCTGAACCGTTGT
CACTTCCACAAGCCTATCCTGGGAAGAGTCTTGCTCTAGAACCCGGAAATCCGCAGAAT
CCCTCTCTTTTCGGTGTTGATCCCGACTCTGGACTCTTCCTCCCCAGTACGGTTCCCCGC
TTTGCTTCTTCATCAGGAGATGCTGAAGCTTCCCCTATGTCACTAACAGATTCAGGATTT
CAGAATTCCTTATATAGCTGCATGCAAGACACAACTCATGAGTTATTGCATGGAGCTGGA
CAGATTAACTCGTCCAACCAAACCAAGAACTTTGTAAAGGTTTATAAATCTGGTTCGGTT
GGGCGTTCATTAGACATCTCCCGATTCAGCAGCTACCACGAGCTGCGAGAAGAGTTAGG
GAAGATGTTTGCTATCGAAGGGTTGTTGGAAGACCCCCTTAGATCAGGCTGGCAGCTTG
TATTCGTTGACAAGGAAAATGATATTCTTCTCCTTGGTGATGACCCATGGGAGTCATTTGT
GAATAACGTTTGGTACATAAAGATACTATCACCAGAAGATGTGCATCAAATGGGAGATCA
TGGAGAAGGCAGTGGTGGGTTATTCCCGCAAAACCCGACCCATCTCTAG

>AT5G39610|AT5G39610.1

ATGGATTACGAGGCATCAAGAATCGTCGAAATGGTAGAAGATGAAGAACATATAGATCTA
CCACCAGGATTCAGATTTCACCCTACTGATGAAGAACTCATAACTCACTACCTCAAACCA
AAGGTTTTCAACACTTTCTTCTCTGCTACTGCCATTGGTGAAGTTGATCTCAACAAGATT
GAGCCTTGGGACTTACCATGGAAGGCTAAGATGGGAGAAAAAGAATGGTATTTCTTCTG
TGTGAGAGACCGGAAATACCCGACCGGTTTAAGGACAAACCGGGCGACAGAAGCCGGT

TATTGGAAAGCCACAGGAAAAGACAAAGAGATATTCAAGGGAAAATCACTTGTGGGTAT
GAAGAAAACTTTGGTTTTCTATAAAGGAAGAGCTCCTAAAGGAGTTAAAACCAATTGGG
TTATGCATGAATATCGTTTAGAAGGCAAATATTGTATTGAAAATCTTCCCCAAACAGCTAA
GAACGAATGGGTTATATGTCGTGTTTTCCAAAAACGTGCCGATGGTACAAAGGTTCCAAT
GTCAATGCTTGATCCACACATTAACCGAATGGAACCAGCCGGTTTACCTTCGTTAATGGA
TTGTTCTCAACGAGACTCCTTCACCGGTTCGTCGTCTCACGTGACCTGCTTCTCCGACCA
AGAAACCGAAGACAAAAGACTTGTCCACGAGTCCAAAGACGGTTTTGGTTCTCTGTTTT
ACTCGGATCCTCTGTTTTTACAAGACAATTATTCGCTAATGAAGCTGTTGCTTGACGGTC
AAGAAACTCAATTCTCCGGCAAACCTTTCGACGGTCGTGATTCGTCCGGTACAGAAGAA
TTGGATTGCGTTTGGAATTTCTGA

>AT3G60460|AT3G60460.1

ATGGAAGCGAAGAAGGAAGAGATAAAGAAAGGTCCATGGAAAGCCGAAGAAGACGAA
GTACTCATCAACCATGTCAAGAGATACGGTCCTCGTGATTGGAGCTCCATTCGATCCAAA
GGTCTTCTTCAACGCACCGGCAAATCCTGTCGTCTTCGTTGGGTCAATAAACTCCGTCCC
AATCTCAAAAATGGATGCAAGTTCTCGGCTGACGAAGAGAGGACTGTGATTGAGTTACA
ATCTGAGTTTGGTAACAAATGGGCGAGAATCGCTACGTATCTACCGGGAAGAACTGATA
ACGATGTGAAGAATTTCTGGAGTAGCAGACAAAAGAGACTCGCTAGGATTCTTCATAAC
TCCTCTGATGCATCGAGTTCGAGTTTCAATCCCAAATCTTCTTCTTCTCATCGACTCAAG
GGCAAAAACGTCAAACCAATCCGTCAATCCTCTCAGGGTTTTGGTTTGGTTGAGGAAGA
GGTTACAGTTTCTTCTTCATGTTCCCAGATGGTTCCTTATTCATCTGATCAAGTTGGTGAT
GAAGTCTTGAGGTTGCCGGATTTGGGTGTTAAGTTAGAGCATCAGCCTTTCGCTTTTGGC
ACTGATCTTGTCCTAGCAGAGTACTCTGACTCACAGAATGATGCAAATCAGCAAGCAAT
CAGCCCTTTCTCTCCAGAAAGCAGAGAGCTTTTGGCTAGACTTGACGACCCTTTTTACTA
TGATATACTTGGACCAGCTGATTCTTCTGAGCCATTGTTCGCTCTCCCTCAGCCGTTCTTC
GAGCCTTCGCCTGTGCCGAGAAGATGCAGACATGTTTCAAAGGATGAAGAAGCTGATGT
TTTCTTAGACGATTTCCCAGCTGACATGTTTGATCAGGTTGATCCAATCCCAAGTCCTTA
G

>AT3G11440|AT3G11440.1

ATGAGTTACACGACGGCGACTGCTGATAGTGATGATGGTATGCACTCCAGCATCCATAAT
GAATCACCAGCTCCTGATAGTATTAGCAATGGCTGCAGAAGTAGAGGGAAAAGAAGTGT
CCTGAAGAAAGGACCATGGACTTCAACTGAAGACGGGATTTTAATTGATTATGTAAAGA
AGCACGGCGAGGGTAACTGGAATGCTGTGCAGAAACACACTAGCCTGGCCCGTTGTGG
TAAAAGCTGTCGTCTGAGATGGGCTAATCATCTGAGGCCAAACTTGAAGAAAGGAGCAT
TTAGCCAAGAAGAAGAACAGCTCATTGTTGAAATGCACGCCAAGATGGGAAATAAATG
GGCACAGATGGCTGAACATTTACCTGGTCGAACAGATAATGAGATAAAGAATTATTGGA
ACACTCGTATCAAGAGGAGACAACGAGCAGGCTTACCACTTTACCCTCCTGAAATCTAT
GTTGATGACCTTCATTGGAGCGAAGAGTATACAAAGAGTAATATCATAAGAGTAGATAGA
AGAAGAAGACATCAAGATTTCTTGCAGTTGGGGAATTCTAAAGATAATGTCTTATTTGAC
GATCTAAATTTTGCTGCTAGCTTGTTACCTGCCGCTTCTGACCTATCAGATTTGGTTGCAT
GCAACATGCTAGGAACTGGCGCAAGTTCTTCCCGGTATGAGAGCTACATGCCACCAATAT
TGCCTTCCCCAAAGCAAATCTGGGAATCTGGATCTCGGTTTCCCATGTGCAGCAGTAACA
TAAAGCATGAATTCCAATCGCCGGAACACTTTCAGAATACGGCTGTACAGAAGAATCCC
AGATCTTGCAGTATCTCGCCTTGTGATGTTGATCATCATCCTTATGAAAACCAACATTCGT
CTCATATGATGATGGTTCCTGATAGCCATACAGTTACGTATGGCATGCATCCTACTTCTAA
GCCCTTGTTTGGGGCAGTGAAGCTGGAGCTCCCTTCATTCCAATATTCAGAAACTAGTGC
ATTTGATCAGTGGAAGACGACTCCGTCACCTCCACACTCAGATCTCCTTGACTCTGTTGA
TGCCTATATTCAATCTCCACCACCATCGCAGGTAGAGGAGTCAGATTGTTTCTCTTCATGC
GACACCGGCCTACTAGATATGTTACTTCATGAGGCCAAGATCAAAACTAGTGCGAAGCA

CAGTTTGTTGATGTCATCACCCCAGAAGAGTTTCAGTTCAACTACTTGCACGACCAATGT
TACTCAGAATGTACCACGTGGCAGCGAAAACCTGATCAAATCAGGAGAATATGAAGATT
CCCAAAAGTATTTGGGTCGCTCCGAGATTACAAGTCCCTCGCAACTTAGTGCAGGTGGT
TTTTCATCAGCTTTTGCAGGGAATGTTGTAAAGACAGAAGAGTTGGATCAGGTTTGGGA
ACCAAAGAGAGTTGATATAACACGGCCTGATGTTTTACTTGCGTCGAGCTGGCTTGACC
AAGGCTGTTATGGCATTGTTAGTGACACAAGCAGCATGAGTGATGCGCTTGCACTTCTTG
GTGGTGACGACATTGGGAACAGTTACGTGACTGTTGGGTCATCATCAGGTCAAGCACCA
CGAGGCGTCGGGTCTTATGGATGGACCAATATGCCTCCTGTTTGGTCGCTGTAA

>AT4G14140|AT4G14140.1

ATGGAAATGGAGACAAAAGCTGGAAAGCAAAAGAAGAGAAGTGTTGACTCGGATGAT
GATGTCTCTAAGGAAAGGAGACCAAAGCGAGCAGCAGCTTGCACAAACTTCAAGGAGA
AATCTCTCCGTATCTCTGACAAATCTGAAACTGTTGAAGCTAAGAAAGAGCAGATCTTG
GCGGAGGAGATCGTGGCCATACAGTTAACTTCTTCTTTGGAGAGCAATGATGATCCTCGT
CCAAACCGGAGGCTGACTGATTTCGTTTTACATGATTCAGAAGGAGTTCCACAGCCTGT
GGAGATGTTGGAACTTGGTGACATATTTATTGAAGGTGTTGTCTTACCTTTAGGTGATGA
GAAAAAGGAAGAAAAGGGTGTCAGGTTTCAATCTTTTGGTCGAGTAGAGAACTGGAAT
ATATCTGGTTATGAAGATGGTTCCCCGGTGATATGGATTTCAACGGCGTTAGCGGATTACG
ATTGCCGTAAACCTTCTAAGAAATACAAGAAATTATATGATTATTTCTTTGAGAAAGCTTG
TGCTTGTGTGGAGGTGTTTAAGAGTTTGTCCAAGAATCCGGATACAAGTCTTGATGAGC
TTCTTGCGGCGGTTTCTAGGTCGATGAGCGGAAGCAAGATCTTTTCTAGCGGTGGAGCC
ATCCAAGAGTTTGTTATATCCCAAGGAGAATTCATATATAACCAACTCGCTGGATTGGATG
AGACAGCCAAGAATCATGAAACATGCTTTGTTGAGAATCGTGTTCTTGTTTCTCTAAGAG
ACCATGAGAGTAATAAAATTCACAAGGCTTTGTCTAATGTGGCTCTGAGGATTGATGAGA
GCAAGGTCGTGACATCTGATCATCTGGTGGATGGTGCCGAGGACGAGGACGTAAAATAT
GCTAAGCTAATCCAAGAAGAAGAGTATCGGAAATCTATGGAGCGGTCGAGAAATAAGAG
AAGCTCAACAACTTCTGGTGGTTCAAGCAGGTTTTACATTAAGATCAGTGAAGATGAGA
TTGCCGATGATTATCCTCTCCCGTCTTACTATAAGAACACCAAAGAAGAAACAGATGAGC
TTGTTCTCTTTGAAGCTGGCTATGAGGTTGATACAAGAGACCTACCTTGCAGAACACTGC
ATAATTGGACTCTTTACAACTCTGATTCACGGATGATATCTTTAGAGGTTCTTCCCATGAG
GCCGTGTGCTGAGATCGATGTCACCGTCTTTGGGTCAGGTGTTGTTGCTGAAGATGATG
GAAGCGGGTTTTGTCTCGATGATTCGGAGAGCTCTACCTCTACGCAATCAAATGATCATG
ATGGGATGAATATATTCCTTAGTCAGATAAAGGAATGGATGATTGAGTTTGGGGCAGAAA
TGATCTTTGTCACATTACGAACTGACATGGCCTGGTACCGACTTGGGAAACCGTCAAAG
CAATATGCTCCTTGGTTTGGAACTGTTATGAAAACAGTAAGGGTTGGCATAAGCATTTTC
AATATGCTCATGAGGGAAAGTAGGGTTGCTAAGCTTTCATATGCAAATGTCATAAAAAGA
CTTTGTGGGTTAGAGGAGAACGATAAAGCTTACATTTCTTCTAAGCTCTTGGATGTTGAG
AGATATGTTGTCGTCCATGGACAAATTATCTTGCAGCTTTTCGAAGAGTATCCTGACAAG
GATATCAAAAGGTGTCCATTTGTTACTAGTCTTGCAAGTAAAATGCAGGATATACACCAC
ACAAAATGGATCATCAAGAAGAAGAAGAAAATTCTGCAAAAGGGAAAGAATTTGAATC
CGAGGGCGGGGATTGCACCTGTGGTATCCAGAATGAAAGCTATGCAAGCAACAACAAC
TCGCCTCGTCAATAGGATTTGGGGAGAGTTTTACTCCATTTACTCTCCTGAGGTTCCATC
GGAGGCCATTAATGCTGAAAATGTGGAGGAAGAGGAGCTTGAAGAGGTAGAAGAGGA
GGACGAGAATGAGGAAGATGATCCAGAGGAGAATGAACTAGAAGCTGTTGAGATTCAA
AATTCTCCTACTCCTAAGAAAATTAAAGGTATTTCTGAAGATATGGAAATAAAATGGGAT
GGTGAGATTCTTGGCAAAACTTCTGCTGGTGAGCCTCTCTATGGAAGAGCCTTTGTTGG
AGGGGATGTGGTGGTGGTAGGTAGTGCAGTCATCTTGGAAGTTGATGATCAAGATGATA
CTCAATTGATCTGTTTTGTGGAGTTCATGTTCGAGAGTTCAAACCACAGCAAGATGCTAC
ATGGGAAACTCTTACAAAGAGGATCTGAGACTGTTCTAGGAATGGCTGCTAACGAGAGG
GAACTGTTCTTGACTAATGAATGTCTTACTGTCCAGCTTAAGGACATAAAAGGAACAGTT
AGTCTCGAGATTCGATCAAGGCTGTGGGGGCATCAATACAGGAAAGAGAACATCGATGT

GGATAAGCTTGACCGGGCAAGAGCAGAAGAAAGAAAAACTAATGGTTTGCCAACAGAC
TACTACTGCAAAAGTTTGTACTCACCTGAGAGAGGTGGATTCTTTAGTCTTCCAAGAAAT
GATATGGGTCTAGGTTCTGGATTCTGTAGTTCATGTAAGATAAGAGAGAATGAAGAGGAA
AGGTCAAAAACTAAACTCAATGATTCAAAGACAGGATTTCTCTCCAATGGGATAGAGTA
TCATAATGGAGATTTTGTCTATGTACTCCCCAACTACATAACTAAAGATGGATTGAAGAA
GGGTAGTAGAAGAACAACTCTTAAGTGTGGTCGGAACGTTGGGTTAAAAGCTTTTGTTG
TTTGCCAATTGCTGGATGTTATTGTTCTAGAAGAATCTAGAAAAGCTAGTAAGGCTTCAT
TTCAGGTTAAACTGACAAGGTTTTATAGGCCCGAGGACATTTCAGAAGAAAAGGCCTAT
GCTTCAGACATCCAAGAGTTGTATTATAGCCAGGATACATATATTCTCCCTCCGGAAGCTA
TACAGGGAAAATGTGAAGTAAGGAAGAAAAGTGATATGCCCCTATGTCGTGAGTATCCA
ATTTTAGACCATATCTTCTTCTGTGAAGTTTTCTATGATTCCTCTACTGGTTATCTCAAGCA
GTTTCCGGCGAATATGAAGCTGAAGTTCTCTACTATTAAAGATGAAACACTTCTAAGAGA
AAAGAAGGGGAAGGGAGTAGAAACTGGAACTAGTTCTGGAATGCTTATGAAGCCTGAT
GAGGTACCTAAAGAGAAGCCTCTAGCTACACTAGATATTTTTGCTGGATGTGGTGGTCTA
TCTCATGGACTAGAAAATGCTGGTGTATCTACTACAAAGTGGGCGATCGAGTATGAAGA
GCCAGCTGGTCATGCGTTTAAACAAAACCATCCTGAAGCAACGGTTTTTGTTGACAACT
GCAATGTGATCCTTAGGGCAATAATGGAGAAATGTGGAGATGTCGATGATTGTGTCTCTA
CTGTGGAGGCAGCTGAACTTGCAGCTAAACTTGATGAGAACCAAAAGAGTACCCTGCC
ACTTCCTGGTCAAGTAGATTTCATCAACGGAGGGCCTCCATGCCAGGGGTTTTCTGGTAT
GAATAGGTTCAGTCACGGCTCTTGGAGTAAAGTCCAGTGTGAAATGATATTAGCATTCTT
GTCTTTTGCTGATTATTTCCGGCCAAAGTACTTTCTTCTCGAGAACGTGAAGAAATTTGT
GACATACAATAAAGGGAGAACATTTCAACTTACTATGGCTTCTCTTCTTGAGATGGGTTA
CCAGGTAAGATTTGGAATCTTGGAGGCAGGTACATATGGAGTTTCCCAACCTCGTAAAA
GAGTTATAATTTGGGCAGCTTCACCAGAAGAAGTTCTTCCAGAATGGCCTGAGCCGATG
CATGTCTTTGATAATCCCGGTAGTAAAATCTCATTACCTCGAGGTTTACGCTATGATGCTG
GTTGTAATACTAAATTTGGTGCACCTTTTCGTTCAATCACGGTGAGAGACACAATCGGCG
ATCTTCCACCAGTAGAAAACGGAGAATCCAAGATAAACAAAGAGTATGGAACTACTCCA
GCCTCGTGGTTCCAAAAGAAGATAAGAGGAAACATGAGTGTTCTCACTGATCATATCTG
CAAAGGGCTGAACGAACTAAACCTCATTCGATGTAAGAAAATCCCAAAGAGGCCTGGT
GCTGATTGGCGTGACCTGCCGGACGAAAATGTGACATTATCAAATGGACTCGTGGAAAA
ATTGCGTCCTTTAGCTCTATCAAAGACAGCTAAAAACCACAACGAATGGAAGGGACTCT
ATGGTAGATTGGACTGGCAAGGAAACTTACCCATTTCCATCACCGATCCTCAGCCCATGG
GTAAGGTGGGAATGTGCTTCCATCCTGAACAGGACAGAATAATCACTGTCCGTGAATGC
GCCCGATCTCAGGGGTTTCCGGATAGCTACGAGTTTTCAGGGACGACAAAACACAAACA
TAGGCAAATTGGAAATGCAGTCCCTCCACCATTGGCCTTCGCTCTCGGTCGGAAGCTCA
AAGAAGCCCTATATCTCAAGAGTTCTCTTCAACACCAATCATAA

>AT2G32460|AT2G32460.2

ATGGTGGGGAGAGGGTTGAAGAAAGGGCCGTGGACAACGACGGAGGATGCGATCTTG
ACGGAGTACGTGAGAAAACACGGTGAAGGTAATTGGAACGCCGTGCAAAAGAACTCA
GGTTTGCTCCGGTGTGGCAAAAGTTGCCGTCTACGGTGGGCGAATCATCTCCGGCCAAA
TCTAAAGAAAGGATCTTTTACTCCTGATGAAGAAAAGATCATCATCGACCTTCACGCTAA
GCTTGGAAACAAATGGGCTCGTATGGCTTCTCAGTTACCTGGAAGAACAGACAATGAGA
TCAAGAACTATTGGAACACGAGGATGAAGAGAAGACAAAGAGCTGGTTTGCCTTTATAC
CCTCATGAGATTCAACATCAAGGGATTGATATTGATGATGAGTTTGAGTTTGATTTAACTT
CCTTTCAGTTCCAAAACCAAGATCTTGATCATAACCACCAAAATATGATTCAGTACACTA
ATTCTTCTAATACTTCATCATCCTCGTCTTCATTCTCTTCTTCATCTTCTCAACCATCAAAA
AGGCTGCGTCCTGATCCTTTAGTCTCTACTAATCCCGGCCTAAACCCGATCCCCGATTCTT
CGATGGATTTTCAAATGTTCTCTCTTTACAACAATAGCCTTGAGAATGACAATAACCAGT
TTGGTTTCTCTGTTCCTTTGTCCTCATCATCCTCGTCTAACGAGGTGTGTAATCCCAACCA
CATCCTTGAGTACATCTCCGAGAATTCGGACACAAGAAATACCAATAAGAAAGACATTG

ATGCTATGAGTTATAGTTCATTGCTTATGGGAGATCTTGAGATAAGATCGAGTTCTTTCCC
TTTAGGACTAGACAATAGCGTCCTAGAGCTTCCTTCAAACCAAAGACCGACCCATTCGT
TCAGTTCTAGTCCTATTATTGACAATGGTGTCCATCTTGAGCCACCTTCTGGCAATAGTGG
ACTACTTGATGCCCTCTTGGAGGAGTCTCAAGCCTTGTCTCGAGGCGGACTCTTCAAGG
ACGTTAGGGTTTCCTCCAGTGACCTATGTGAGGTTCAAGATAAAAGGGTGAAGATGGAC
TTTGAGAATCTTTTAATAGATCATCTAAACTCTTCTAATCATTCATCATTGGGAGCAAACC
CTAATATTCACAACAAGTACAATGAGCCAACAATGGTAAAAGTAACGGTGGATGATGAT
GATGAATTATTGACGAGCCTTCTCAACAACTTCCCTTCAACCACAACACCTTTGCCTGAT
TGGTATCGGGTGACAGAAATGCAAAACGAGGCCTCATATCTTGCCCCACCAAGTGGAAT
TCTTATGGGAAACCATCAAGGTAACGGCAGGGTGGAACCACCCACGGTGCCGCCTTCGT
CCAGTGTAGATCCTATGGCCTCGTTGGGGTCATGCTATTGGAGCAACATGCCTAGCATCT
GTTAG

>AT5G43740|AT5G43740.1

ATGCTGGGCTGGCTTGTAATACCCTGGAATCAAATCTTCACTGCTGCATGTGGCTGCTTT
CTAAGCGATAGAAACTACATTCACATGATGGAGTCAAATCTCGATGCTCTGCAGAAAACT
ATGGAAGAACTTAAGAATGGACGAGATGACCTGTTAGGAAGAGTTTCCATAGAAGAAGA
TAAAGGTTTGCAACGACTTGCTCAAGTCAATGGATGGCTTTCCAGGGTACAAATTGTTG
AATCCGAATTCAAGGATCTGCTTGAGGCTATGTCAATCGAAACCGGAAGATTGTGTTTGT
TGGGATATTGCTCTGAGGATTGCATATCAAGCTACAATTATGGTGAAAAGGTATCGAAGA
TGTTGGAAGAAGTTAAAGAACTTCTATCTAAAAAAGATTTTAGAATGGTCGCGCAGGAA
ATTATACATAAGGTGGAGAAGAAGCTTATCCAAACCACAGTTGGTTTGGATAAACTGGTG
GAAATGGCGTGGAGCAGCCTGATGAACGATGAAATAGGAACCTTGGGACTTTATGGTAT
GGGGGGAGTAGGAAAAACCACCCTCTTAGAAAGTCTCAACAACAAATTTGTTGAACTG
GAGAGTGAATTTGATGTAGTGATATGGGTTGTGGTATCTAAAGACTTTCAGTTCGAGGGC
ATTCAAGATCAGATTCTGGGAAGATTACGTTCTGACAAGGAATGGGAAAGAGAAACAG
AAAGTAAGAAAGCCTCTCTCATATACAATAACCTTGAGAGAAAGAAATTTGTGTTGTTGT
TGGATGATCTCTGGAGTGAAGTAGATATGACTAAGATAGGAGTTCCACCTCCAACTCGA
GAAAATGGATCGAAGATTGTTTTCACCACTCGTTCAACGGAAGTTTGCAAGCACATGAA
AGCTGACAAGCAGATAAAAGTTGCTTGTTTGTCCCCAGATGAAGCGTGGGAATTGTTTC
GACTTACTGTTGGAGATATCATATTAAGGAGCCATCAAGATATTCCCGCACTTGCAAGAAT
AGTTGCTGCAAAATGCCATGGCTTGCCACTTGCACTCAATGTCATTGGCAAAGCCATGTC
ATGTAAAGAAACTATACAAGAATGGTCTCATGCGATTAATGTTCTGAATTCGGCTGGCCA
CGAGTTTCCAGGTATGGAAGAAAGAATTCTTCCCATTTTGAAGTTCAGTTATGATAGCTT
GAAGAATGGTGAAATCAAATTGTGCTTCCTATATTGTTCTCTGTTTCCTGAAGATTCTGA
AATACCGAAGGAGAAGTGGATAGAATACTGGATATGCGAAGGATTTATAAATCCAAACA
GATATGAAGATGGAGGTACCAACCATGGTTATGATATAATTGGCTTGTTAGTTCGTGCACA
TTTGTTGATTGAATGTGAACTCACAGACAACGTTAAAATGCATGATGTGATACGTGAGAT
GGCTCTTTGGATAAATTCTGACTTCGGAAAACAACAAGAAACAATCTGTGTGAAATCTG
GTGCTCATGTACGTATGATACCGAATGACATCAATTGGGAGATTGTGAGAACGATGTCTT
TTACTTGTACTCAGATTAAGAAGATATCTTGTCGTTCCAAATGCCCCAACCTTTCAACCCT
TTTAATCCTAGATAACAGGTTGTTGGTGAAAATTTCAAATAGATTCTTTCGGTTTATGCCA
AAACTTGTGGTCTTGGATCTTTCTGCAAACCTCGATCTTATTAAATTGCCGGAAGAAATT
TCTAACCTAGGTTCCCTGCAATATCTCAATATATCACTCACAGGGATAAAATCGCTACCAG
TTGGTCTGAAGAAGTTGAGGAAACTAATCTACTTGAATCTGGAGTTCACTGGTGTACAT
GGGAGTCTTGTTGGGATAGCAGCAACCTTACCAAATCTGCAAGTGTTGAAATTCTTTTAT
TCCTGTGTTTATGTTGATGACATATTAATGAAGGAATTACAAGACTTGGAGCACTTGAAG
ATTTTAACAGCAAACGTAAAGGATGTCACGATTTTGGAAAGGATACAAGGAGATGACAG
ATTGGCGAGTAGTATTCGAAGTTTATGTCTAGAAGATATGTCAACACCTCGTGTAATATTA
AGCACGATAGCTCTGGGAGGTCTTCAACAACTTGCAATTCTGATGTGCAACATATCCGA
GATAAGGATAGATTGGGAAAGCAAAGAAAGAAGGGAGCTTTCACCAACGGAGATTCTT

CCATCTACAGGTTCTCCAGGCTTCAAGCAACTCTCCACTGTTTATATAAACCAGTTGGAA
GGGCAAAGGGATTTGTCATGGCTGTTATATGCTCAAAATCTAAAGAAACTAGAAGTGTG
CTGGTCACCACAAATAGAAGAAATAATAAATAAGGAGAAAGGAATGAATATTACCAAAC
TGCATCGAGATATTGTTGTTCCATTTGGGAACCTGGAAGACCTTGCGCTACGTCAAATGG
CGGACCTCACGGAAATTTGCTGGAATTATCGGACTCTCCCTAACCTTAGAAAATCATATAT
CAACGATTGTCCAAAGCTACCTGAAGATATTTTCGTGCCTCTGCTTCCTGAAAAGAGTCC
TTCACGTTTTTTTTTCTTTTAA

>AT5G60690|AT5G60690.1

ATGGAGATGGCGGTGGCTAACCACCGTGAGAGAAGCAGTGACAGTATGAATAGACATTT
AGATAGTAGCGGTAAGTACGTTAGGTACACAGCTGAGCAAGTCGAGGCTCTTGAGCGTG
TCTACGCTGAGTGTCCTAAGCCTAGCTCTCTCCGTCGACAACAATTGATCCGTGAATGTT
CCATTTTGGCCAATATTGAGCCTAAGCAGATCAAAGTCTGGTTTCAGAACCGCAGGTGT
CGAGATAAGCAGAGGAAAGAGGCGTCGAGGCTCCAGAGCGTAAACCGGAAGCTCTCTG
CGATGAATAAACTGTTGATGGAGGAGAATGATAGGTTGCAGAAGCAGGTTTCTCAGCTT
GTCTGCGAAAATGGATATATGAAACAGCAGCTAACTACTGTTGTTAACGATCCAAGCTGT
GAATCTGTGGTCACAACTCCTCAGCATTCGCTTAGAGATGCGAATAGTCCTGCTGGATTG
CTCTCAATCGCAGAGGAGACTTTGGCAGAGTTCCTATCCAAGGCTACAGGAACTGCTGT
TGATTGGGTTCAGATGCCTGGGATGAAGCCTGGTCCGGATTCGGTTGGCATCTTTGCCAT
TTCGCAAAGATGCAATGGAGTGGCAGCTCGAGCCTGTGGTCTTGTTAGCTTAGAACCTA
TGAAGATTGCAGAGATCCTCAAAGATCGGCCATCTTGGTTCCGTGACTGTAGGAGCCTT
GAAGTTTTCACTATGTTCCCGGCTGGTAATGGTGGCACAATCGAGCTTGTTTATATGCAG
ACGTATGCACCAACGACTCTGGCTCCTGCCCGCGATTTCTGGACCCTGAGATACACAAC
GAGCCTCGACAATGGGAGTTTTGTGGTTTGTGAGAGGTCGCTATCTGGCTCTGGAGCTG
GGCCTAATGCTGCTTCAGCTTCTCAGTTTGTGAGAGCAGAAATGCTTTCTAGTGGGTATT
TAATAAGGCCTTGTGATGGTGGTGGTTCTATTATTCACATTGTCGATCACCTTAATCTTGA
GGCTTGGAGTGTTCCGGATGTGCTTCGACCCCTTTATGAGTCATCCAAAGTCGTTGCACA
AAAAATGACCATTTCCGCGTTGCGGTATATCAGGCAATTAGCCCAAGAGTCTAATGGTGA
AGTAGTGTATGGATTAGGAAGGCAGCCTGCTGTTCTTAGAACCTTTAGCCAAAGATTAAG
CAGGGGCTTCAATGATGCGGTTAATGGGTTTGGTGACGACGGGTGGTCTACGATGCATT
GTGATGGAGCGGAAGATATTATCGTTGCTATTAACTCTACAAAGCATTTGAATAATATTTC
TAATTCTCTTTCGTTCCTTGGAGGCGTGCTCTGTGCCAAGGCTTCAATGCTTCTCCAAAA
TGTTCCTCCTGCGGTTTTGATCCGGTTCCTTAGAGAGCATCGATCTGAGTGGGCTGATTT
CAATGTTGATGCATATTCCGCTGCTACACTTAAAGCTGGTAGCTTTGCTTATCCGGGAATG
AGACCAACAAGATTCACTGGGAGTCAGATCATAATGCCACTAGGACATACAATTGAACA
CGAAGAAATGCTAGAAGTTGTTAGACTGGAAGGTCATTCTCTTGCTCAAGAAGATGCAT
TTATGTCACGGGATGTCCATCTCCTTCAGATTTGTACCGGGATTGACGAGAATGCCGTTG
GAGCTTGTTCTGAACTGATATTTGCTCCGATTAATGAGATGTTCCCGGATGATGCTCCACT
TGTTCCCTCTGGATTCCGAGTCATACCCGTTGATGCTAAAACGGGAGATGTACAAGATCT
GTTAACCGCTAATCACCGTACACTAGACTTAACTTCTAGCCTTGAAGTCGGTCCATCACC
TGAGAATGCTTCTGGAAACTCTTTTTCTAGCTCAAGCTCGAGATGTATTCTCACTATCGC
GTTTCAATTCCCTTTTGAAAACAACTTGCAAGAAAATGTTGCTGGTATGGCTTGTCAGTA
TGTGAGGAGCGTGATCTCATCAGTTCAACGTGTTGCAATGGCGATCTCACCGTCTGGGAT
AAGCCCGAGTCTGGGCTCCAAATTGTCCCCAGGATCTCCTGAAGCTGTTACTCTTGCTC
AGTGGATCTCTCAAAGTTACAGTCATCACTTAGGCTCGGAGTTGCTGACGATTGATTCAC
TTGGAAGCGACGACTCGGTACTAAAACTTCTATGGGATCACCAAGATGCCATCCTGTGTT
GCTCATTAAAGCCACAGCCAGTGTTCATGTTTGCGAACCAAGCTGGTCTAGACATGCTA
GAGACAACACTTGTAGCCTTACAAGATATAACACTCGAAAAGATATTCGATGAATCGGGT
CGTAAGGCTATCTGTTCGGACTTCGCCAAGCTAATGCAACAGGGATTTGCTTGCTTGCCT
TCAGGAATCTGTGTGTCAACGATGGGAAGACATGTGAGTTATGAACAAGCTGTTGCTTG
GAAAGTGTTTGCTGCATCTGAAGAAAACAACAACAATCTGCATTGTCTTGCCTTCTCCTT

TGTAAACTGGTCTTTTGTGTGA

>AT2G34710|AT2G34710.1

ATGATGATGGTCCATTCGATGAGCAGAGATATGATGAACAGAGAGTCGCCGGATAAAGG
GTTAGATTCCGGCAAGTATGTGAGGTACACGCCGGAGCAAGTGGAAGCTCTCGAGAGA
GTTTACACTGAGTGTCCTAAGCCAAGTTCTCTAAGAAGACAACAACTCATACGTGAATG
TCCGATTCTCTCTAACATCGAGCCTAAGCAGATCAAAGTTTGGTTTCAGAACCGCAGATG
TCGTGAGAAGCAGAGGAAAGAAGCTGCTCGTCTTCAAACAGTGAACAGAAAACTCAAT
GCCATGAACAAACTCTTGATGGAAGAGAATGATCGTTTGCAGAAGCAAGTTTCTAACTT
GGTCTATGAGAATGGCCACATGAAACATCAACTTCACACTGCTTCTGGGACGACCACAG
ACAACAGCTGTGAGTCTGTGGTCGTGAGTGGTCAGCAACATCAACAGCAAAACCCAAA
TCCTCAGCATCAGCAACGTGATGCTAACAACCCAGCAGGACTCCTTTCTATAGCAGAGG
AGGCCCTAGCAGAGTTCCTTTCCAAGGCTACAGGAACTGCTGTTGACTGGGTTCAGATG
ATTGGGATGAAGCCTGGTCCGGATTCTATTGGCATAGTCGCTATTTCGCGCAACTGCAGC
GGAATTGCAGCACGTGCCTGCGGCCTCGTGAGTTTAGAACCCATGAAGGTTGCTGAAAT
TCTCAAAGATCGTCCATCTTGGCTCCGAGATTGTCGAAGTGTGGATACTCTGAGTGTGAT
ACCTGCTGGAAACGGTGGGACGATCGAGCTTATTTACACGCAGATGTATGCTCCTACGA
CTTTAGCAGCAGCTCGTGACTTTTGGACGCTGAGATATAGCACATGTTTGGAAGATGGA
AGCTATGTGGTTTGTGAAAGGTCGCTTACTTCTGCAACTGGTGGCCCCACTGGGCCACC
TTCTTCAAACTTTGTGAGAGCTGAAATGAAACCAAGCGGGTTTCTCATCCGTCCTTGCG
ATGGTGGTGGTTCCATTCTCCACATTGTTGATCATGTTGATCTGGATGCCTGGAGTGTCCC
TGAAGTCATGAGGCCTCTCTATGAATCATCGAAGATTCTTGCTCAGAAAATGACTGTTGC
TGCTTTGAGACATGTAAGACAAATTGCACAAGAAACAAGTGGAGAAGTTCAGTATGGT
GGAGGGCGCCAACCTGCGGTTTTAAGAACCTTCAGTCAAAGACTCTGTCGGGGTTTCAA
TGATGCTGTTAATGGTTTTGTGGATGATGGATGGTCACCAATGGGTAGCGATGGTGCAGA
GGATGTTACTGTAATGATAAACTTGTCCCCTGGGAAGTTTGGTGGGTCTCAGTACGGTAA
TTCATTCCTTCCAAGCTTTGGTAGTGGCGTGCTTTGTGCCAAGGCATCTATGTTGCTTCA
GAACGTTCCACCCGCTGTGCTGGTTCGATTCCTTAGAGAACACCGATCTGAATGGGCTG
ATTATGGCGTGGATGCTTATGCTGCTGCATCGCTCAGAGCAAGTCCTTTTGCTGTTCCTTG
TGCTAGAGCTGGGGGGTTCCCAAGTAACCAAGTCATTCTTCCTCTTGCGCAGACAGTTG
AACATGAAGAGTCACTTGAGGTGGTTAGACTTGAAGGTCACGCTTACTCACCCGAAGA
CATGGGTTTAGCTCGGGATATGTATTTGCTACAGCTTTGTAGCGGTGTTGATGAAAATGTG
GTTGGAGGTTGTGCACAGCTTGTATTTGCCCCTATCGATGAATCATTTGCTGATGATGCAC
CTTTGCTTCCTTCCGGTTTCCGCATCATACCTCTTGAACAGAAATCTACTCCGAACGGTG
CATCTGCAAACCGTACCCTGGATTTAGCCTCAGCTTTAGAAGGATCCACACGTCAAGCT
GGTGAAGCCGACCCAAATGGCTGTAACTTTAGGTCGGTACTAACCATAGCATTCCAGTTC
ACATTTGATAACCATTCAAGAGACAGTGTTGCTTCAATGGCACGTCAGTACGTGCGAAG
CATAGTAGGATCGATTCAGAGGGTTGCTCTAGCCATTGCTCCTCGTCCTGGCTCCAATATC
AGTCCAATATCTGTTCCCACTTCCCCTGAAGCTCTCACTCTGGTCCGTTGGATCTCCCGG
AGTTACAGCCTTCACACTGGTGCAGATCTCTTTGGATCTGATTCTCAAACCAGTGGTGAC
ACGTTGCTGCATCAACTCTGGAATCACTCTGATGCAATCTTGTGCTGCTCCCTCAAAACA
AACGCTTCACCGGTTTTCACATTCGCAAACCAAACCGGTTTAGACATGCTGGAAACGAC
TCTTGTAGCCCTTCAAGACATAATGCTAGACAAGACCCTTGACGAACCTGGTCGTAAAG
CTCTTTGCTCTGAGTTCCCCAAGATCATGCAACAGGGCTATGCTCATCTGCCGGCAGGAG
TATGTGCGTCAAGCATGGGAAGGATGGTATCTTACGAGCAGGCAACGGTGTGGAAAGTT
CTTGAAGACGATGAATCAAACCACTGCTTAGCTTTCATGTTCGTGAATTGGTCGTTCGTT
TGA

>AT5G49850|AT5G49850.1

ATGACTGAAAGATCGGAAGCGTTAGGGAAGGATGGCAACCGTAGGTGGGATGACAAAT

CTGACCACGATGATGTAACAAAGATATATGTAAATTATTCTCTTATGGGCATAGAGTCCAT
TCGCTTCGATTATGTCAAAAGTGGAAAACCAATTGAAGGACCCTTCCGTGGTGAAACGT
ATAATACATACACGCATACGTTTGAGATTAACCATCTAAAAAATGAACATCTCGAATCTGT
TGAGGGCTCCTACACACAGAGAGGGATTCAAACACTTCAATTTAAAACCAACTTGCGGA
TTTCTGAACCGATTGGATATCCTGGCAAGGATGGTATTAAGTTCATATTAGCAGTAGAGG
GAAAAAAAATCATTGGGTTTCACGGATCAACTTATTTCAGGCTGTACTCTCTAGGAGCAT
ATTTCACTCGGGTTACTCCTACAAGAATAGAAGCGATAGGGGGCAAGGTAGGCACAAAG
TGGGATGATGGAGTCGACCAGGCAGGTTTTACAAAGATACATGTACGAAGTGGTCAAGA
AGGCATACAATTCATCAAGTTTGAGTATGTTGACAAGAATGGGCGTCTGAGAGATGGGT
CAATCCATGGTTCTATTTACAGAAGAGGTTCCCCACACGTGTTTGAGATTAGACATGTTG
ACAAAGAATATCTGGTATCGGTAGAGGGTTACTATGACGGTGATGGCGACTGTGCGGTCA
TTCAAGCACTTCGATTCAGAACCAACGTCAAGACTTCACAACTTATGGGACCCAAAACG
GGTAAGAAGTTTAGACTTGCAGCCAGTGGAATGAAGATTGTCGGGTTTCATGGATATGCT
GAGAAGAATCTTACCTCTCTTGGAGGATATTTCACACCGATTATTCCAACAAAGTCAGAA
TGCCAAGGAGTTACTGAACGAAGCACCCTTTGGGATAGTGGTGCTTTTGAAGGTATTAG
AAAGGTTTCCGTTACATGGAGATCTTATTGTATACGGTGTTTCCGGATCAACTATGAAAA
CGATGGCAAAGTAGTAAAGCGTGCCCATGGAATGAACGACGATTCGCGAATCACTGACG
AGTTTGTTGTTGACTATCCATATGAAGTTATCACGTCCATTGTGGGGACCATGAATGATAG
TTATGTTACGTCATTTGTTTTCAAAACATCAAAAGGGAGAACCTCTAGGACATTTGGTGA
GAGAACTTCTGATTCAGTTGAATTTGTCATTGAGAGCAAAGGTTGTGCCGTTGTCGGATT
TCATGGATGGTATGCACCTCTTGGGGCTGGGTATATTACGGCTCTTGGTGCACATTTTTAT
CCGATGCCTCTTCCTCCTGCTGCGGAAAAGCTAGAAGCACAAGGTGGTGCTGGAGGAG
TTCCATGGGACGATGGTAGTAATTTTGAACGTGTTAGAAAGATATACATTGGAACATGTG
AGGTTGGTATTGTTTCCGTCAGGTTTTTGTATGAAAATGACATTGAAGAGATAGTAGTGG
GAGATCATCATGGGAACAAGAACCTACTTCGACATGAAGAGTTCGACCTGGACAATGCT
TGCGAATACCTCACATCAGTGGAAGGTAGTTATGACGTAATACCTGGAAGTGAAGATGTT
GAAGTTATACTCATGCTCAAGTTCACCACCAACAAGCGAACTTCTCCATGTTATGGGCTC
GATGATGACCCAACCTTCGTGCTCCACAAGGCAGGTCACAGAATCATTGGGTTCCATGG
AAAATCAAGTAACATGCTTCATAAACTTGGGATCCATGTGCTCCCCATCACCGACCCATG
A

>AT5G06100|AT5G06100.2

ATGAGTTACACGAGCACTGACAGTGACCATAATGAGTCACCAGCTGCTGATGATAATGG
AAGTGACTGCAGAAGTAGATGGGATGGTCATGCTCTCAAGAAAGGCCCTTGGAGTTCA
GCTGAAGATGATATTCTTATTGACTATGTGAATAAGCATGGTGAGGGTAACTGGAATGCT
GTGCAGAAACACACCAGCTTGTTTCGTTGTGGTAAAAGCTGTCGTCTAAGATGGGCTAA
TCATCTGAGGCCAAATTTGAAGAAAGGAGCTTTTAGTCAAGAAGAAGAACAGCTTATCG
TTGAATTGCATGCCAAGATGGGTAATAGATGGGCACGTATGGCTGCACATTTGCCTGGCC
GAACGGATAATGAGATAAAGAATTATTGGAACACTCGTATCAAGAGGCGACAACGAGCT
GGTTTGCCACTTTATCCTCCTGAGATGCATGTTGAGGCACTTGAGTGGAGTCAAGAGTAT
GCCAAGAGTAGAGTTATGGGAGAAGATAGAAGACATCAAGATTTCTTGCAGCTGGGGA
GTTGTGAATCTAATGTCTTCTTTGATACTCTTAATTTTACCGACATGGTACCTGGTACTTTT
GATCTAGCAGATATGACTGCCTACAAAAATATGGGTAACTGTGCAAGTTCTCCTCGATAT
GAAAACTTCATGACACCAACAATCCCCTCCTCGAAGCGACTTTGGGAATCTGAGTTGTT
GTATCCTGGGTGTAGCAGTACCATAAAGCAAGAATTCTCGTCGCCTGAACAATTCCGGA
ACACATCTCCACAAACGATTTCCAAAACTTGCAGCTTCTCAGTTCCTTGTGATGTTGAGC
ATCCTCTCTATGGAAACCGACATTCACCTGTTATGATTCCAGATAGCCATACCCCTACGGA
TGGCATTGTTCCTTATTCTAAGCCCTTATATGGGGCAGTGAAGCTGGAGCTCCCTTCATTC
CAATATTCAGAAACAACATTTGACCAGTGGAAGAAATCGTCATCTCCTCCACACTCTGAT
CTCCTTGATCCCTTTGATACTTACATTCAGTCTCCACCACCACCAACGGGGGGAGAAGA
GTCAGATTTATATTCAAATTTTGATACTGGTCTGCTCGATATGTTGCTTCTGGAAGCCAAG

ATCAGAAATAATAGTACAAAGAACAATTTGTACAGGAGCTGCGCTTCAACTATTCCATCA
GCTGATCTTGGCCAGGTTACTGTATCCCAAACTAAATCCGAGGAGTTTGACAATTCCCTT
AAGAGCTTCTTGGTTCATTCCGAAATGTCCACACAAAATGCAGATGAAACTCCACCAAG
GCAGAGAGAAAAAAAGCGGAAACCTCTCTTGGATATAACAAGGCCTGACGTTTTGCTT
GCATCGAGCTGGCTTGACCATGGTTTAGGGATTGTGAAAGAGACAGGTAGCATGAGCGA
CGCACTCGCGGTTCTCCTTGGCGATGACATAGGAAATGACTATATGAATATGAGTGTTGG
GGCATCCTCAGGAGTTGGGTCTTGTTCTTGGAGCAACATGCCACCTGTCTGTCAAATGA
CAGAACTACCCTAA

>AT3G44860|AT3G44860.1

ATGTCGACTTCATTCACAATGATCGGCGGTGAAGGTCCCAACAGTTACCGGGAACATTC
GAAATACCAGGGAGCACTGGTTATAGCTGCAAAGGAAAAGATCAATGAAGCCATCTCCA
CGAAACTCGATATCGACTTTACTTCAAATCTTGTTAACATAGCTGATTTTGGTTGTTCCTC
TGGACCGAACACTTTCACCGCGGTACAAACCTTAATTGATGCTGTGGAAAACAAGTATA
AGAAAGAAAGTAATATTGAGGGAATCGAGTTCCAAGTTTTCTTCAATGATTCTTCAAAC
AACGATTTTAACACTCTCTTCAAGACACTTCCTCCGGCTAGACTGTATTTCGCAAGTGGA
GTACCGGGTTCTTTCTTTGGTCGTGTTCTTCCTAAAAATAGTCTTCATGTGGGAGTTTCTT
CTTACTCACTCCATTTCGTATCCAAGGTTCCCAAAGAAATCAAAGACCGTGACTCGCTTG
TGTGGAACAAAGACATACATTGCTCCGGATCTTCAAAAGAAGTTGTGAAATTGTATCTC
GGTCAATACAAGATCGACGTGGGAAGTTTCTTGACCGCGAGAGCTCAAGAGCTCGTGTC
CGGTGGATTACTATTGCTTCTTGGTTCATGTCGTCCAACTGGAGTTCAAATGTTTGAAAC
GGTTGAAGGCATGATGATTGATTTCATTGGAAGTTCTCTTAATGAAATTGCTAACCAGGG
TCTCATAGATCAACAAAAGCTTGACACTTTTAAATTGCCTATCTATGCTCCAAATGTGGAT
GAATTGAAGCAAATCATCGAGGATAACAAGTGTTTCACGATTGAGGCATTCGAAAAGAT
TAGCCACGCAAAGGGGGAGTATCCGTTAGACCCCGAGTATTTGACGTCCGCGTTTAAGG
TCACTGTTGGAGGATCAGTAGCTTCACTATTTGGGCAAGATGGGATGGAGAAAACCTAT
GAGCTTGTGAAAGAGAAGACGCAAGAAATGCTTCCTCAAATAGCCAAAGCCAAACCTG
GAATGCAATACCTCATTGTGCTTCGAAGGAACTGA

>AT3G60630|AT3G60630.1

ATGCCCCTGCCCTTTGAGCAATTTCAAGGGAAGGGGGTTCTGGGTTTCTTAGATTCTTCT
TCTTCTCCGGGATACAAAATCTGGGCTAATCCAGAGAAGCTCCATGGACGAGTAGAAGA
AGATCTCTGCTTTGTTGTCAACAATGGTGGTTTCTCGGAGCCGACGTCTGTTTTAGACTC
TGTTAGAAGTCCAAGCCCTTTCGTCTCTTCTTCAACCACCACGCTGTCTTCTTCTCACGG
TGGTCCCAGCGGCGGCGGCGCTGCTGCTGCTACTTTTTCCGGCGCCGATGGGAAATGCG
ACCAAATGGGTTTCGAGGATCTCGATGGTGTTCTCTCCGGTGGCTCGCCGGGACAAGAA
CAGAGTATTTTTAGATTAATCATGGCTGGCGATGTAGTGGATCCGGGTTCGGAGTTTGTG
GGTTTCGACATCGGTTCTGGATCCGACCCGGTTATTGATAATCCTAATCCACTCTTTGGAT
ATGGCTTCCCTTTTCAAAACGCACCGGAAGAAGAAAAGTTTCAGATTTCAATAAACCCA
AATCCGGGTTTCTTCTCGGATCCTCCGTCGTCTCCTCCTGCGAAACGGCTCAATTCGGGT
CAACCCGGATCTCAACACCTCCAGTGGGTTTTCCCGTTCTCGGATCCGGGTCACGAATC
TCACGACCCGTTTCTCACACCGCCAAAGATAGCCGGAGAAGACCAAAACGACCAAGAC
CAGTCAGCGGTAATCATCGACCAGCTATTCTCTGCGGCGGCGGAGCTCACCACAAACGG
CGGAGATAACAATCCCGTTCTCGCGCAAGGGATATTGGCGCGGCTCAATCACAACCTTA
ACAACAACAACGACGACACTAACAACAATCCTAAACCTCCGTTCCACAGAGCAGCTTC
GTATATAACAGAAGCTCTTCACTCTCTCCTTCAAGACTCATCATTATCACCACCGTCTCTC
TCACCTCCTCAAAACCTAATCTTTCGGATCGCAGCTTACAGAGCTTTCTCAGAAACGTCA
CCGTTTCTTCAATTCGTCAACTTCACAGCAAACCAAACGATTCTCGAGTCATTCGAAGG
GTTTGATCGGATCCACATTGTCGATTTCGATATCGGTTATGGAGGTCAATGGGCGTCTCTG
ATTCAAGAGCTCGCCGGAAAAAGAAACAGATCTTCATCAGCTCCGTCGCTAAAGATTAC

AGCTTTCGCTTCTCCTTCAACTGTCTCCGACGAATTCGAGCTCCGATTCACTGAAGAAAA
TCTCAGAAGCTTCGCCGGCGAAACAGGTGTCTCCTTCGAAATCGAGCTCTTAAACATGG
AGATTCTCTTGAATCCAACTTATTGGCCACTGTCTTTATTCCGATCATCGGAGAAAGAAG
CAATCGCTGTGAATCTCCCAATCAGCTCCATGGTCTCCGGTTACCTCCCATTGATACTTCG
TTTCCTCAAGCAAATCTCACCAAACGTCGTCGTTTGCTCAGACAGAAGCTGCGACCGTA
ACAACGACGCGCCGTTCCCTAACGGTGTGATTAACGCGCTTCAGTACTACACATCTCTGC
TCGAGTCTCTCGACTCTGGGAATCTGAATAATGCGGAAGCTGCTACGAGTATTGAGAGG
TTTTGTGTGCAACCGTCGATACAGAAACTGTTGACGAATCGTTACCGTTGGATGGAGAG
ATCACCGCCGTGGAGAAGCTTATTTGGGCAATGTGGGTTTACTCCTGTGACGCTGAGTC
AGACGGCGGAGACACAAGCGGAGTATTTGTTGCAGAGGAATCCAATGAGAGGGTTTCA
CTTGGAGAAGAGACAGTCTTCGTCGCCTTCACTTGTCTTGTGTTGGCAGAGGAAAGAA
CTTGTTACTGTCTCAGCTTGGAAATGTTAA

>AT5G53660|AT5G53660.1

ATGGACTTTCTCAAAGTTTCAGACAAGACAACAATTCCATATAGAAGTGATTCTTTGTTT
AGTTTGAATCAGCAACAATACAAAGAGTCTTCTTTTGGATTCAGAGACATGGAGATTCAT
CCGCATCCTACTCCATATGCAGGAAATGGACTTTTGGGTTGTTATTACTATTACCCTTTCA
CAAACGCACAATTGAAGGAGCTTGAGAGACAAGCAATGATCTACAAGTACATGATCGCA
TCTATTCCTGTTCCTTTCGATCTACTTGTTTCTTCACCATCCTCTGCCTCTCCTTGTAACAA
TAAAAACATCGCCGGAGATTTAGAGCCGGGAAGATGCCGGAGAACAGACGGAAAGAAA
TGGAGATGCGCGAAAGAAGTCGTCTCTAATCACAAATACTGTGAGAAACACTTACACAG
AGGTCGTCCTCGTTCAAGAAAGCATGTGGAACCTCCTTATTCTCGCCCTAACAACAATG
GTGGTTCTGTGAAAAACAGAGATCTCAAAAAGCTTCCTCAAAAGTTATCTAGTAGTTCC
ATCAAAGACAAAACACTTGAGCCAATGGAGGTTTCATCATCAATCTCAAACTATAGAGA
CTCCAGAGGAAGTGAGAAATTTACTGTATTGGCAACAACAGAGCAAGAGAACAAGTAT
CTGAATTTCATAGATGTATGGTCCGATGGAGTAAGATCATCTGAAAAACAGAGTACAACT
TCAACACCTGTTTCTTCTTCCAATGGCAATCTCTCTCTTTACTCGCTTGATCTCTCAATGG
GAGGAAACAACTTAATGGGCCAAGACGAAATGGGCCTGATACAAATGGGCTTAGGTGTA
ATCGGGTCGGGTAGTGAGGATCATCACGGGTATGGTCCTTATGGTGTGACTTCTTCACTA
GAGGAGATGTCAAGCTGGCTTGCTCCGATGTCTACCACACCTGGTGGACCATTAGCGGA
GATACTGAGGCCGAGTACGAATTTGGCGATCTCTGGTGATATCGAATCGTATAGCTTGAT
GGAGACTCCCACTCCAAGCTCGTCCCCGTCTAGAGTGATGAAGAAGATGACTAGTTCAG
TGTCCGACGAAAGCAGCCAGGTTTAG

>AT3G05690|AT3G05690.1

ATGGCTATGCAAACTGTGAGAGAAGGTCTCTTCTCTGCTCCACAGACTTCTTGGTGGAC
TGCTTTTGGATCTCAGCCGTTGGCTCCGGAGAGTCTCGCCGGCGATTCTGACTCATTCGC
CGGAGTTAAGGTCGGATCTGTCGGAGAGACAGGACAACGTGTGGATAAACAGAGCAAC
TCTGCAACGCACTTAGCTTTCTCACTTGGTGATGTAAAGAGTCCAAGACTTGTGCCAAA
GCCTCATGGAGCTACTTTCTCAATGCAATCACCTTGCTTGGAACTTGGATTTTCTCAGCC
ACCGATCTATACAAAGTATCCCTATGGAGAACAACAATACTATGGAGTTGTTTCAGCCTAT
GGATCTCAGAGCAGGGTAATGCTTCCTCTAAACATGGAAACGGAAGATAGTACCATCTAT
GTGAACTCAAAGCAATACCATGGAATCATAAGGAGACGCCAATCCCGCGCAAAGGCTGC
TGCTGTTCTTGATCAGAAGAAATTGAGTAGTAGATGCCGCAAGCCATATATGCATCATTC
GCGCCATCTCCATGCATTGCGGCGTCCTAGAGGATCCGGTGGGAGATTCTTGAACACTA
AAAGTCAGAACTTGGAAAATAGCGGAACCAATGCAAAGAAAGGTGATGGAAGTATGCA
GATTCAGTCTCAGCCTAAGCCTCAGCAAAGTAACTCTCAGAATTCTGAAGTTGTTCATCC
GGAAAACGGGACCATGAACTTATCGAACGGATTAAATGTGTCGGGATCAGAAGTTACTA
GCATGAACTACTTCCTAAGTTCTCCCGTTCATTCTCTTGGTGGCATGGTAATGCCTAGCA
AGTGGATAGCAGCAGCAGCAGCAATGGATAATGGCTGCTGCAATTTCAAAACCTGA

>AT3G57230|AT3G57230.2

ATGGGAAGGGGCAAGATCGCGATTAAGAGGATCAATAACTCTACGAGCCGTCAGGTTAC
GTTCTCGAAGCGAAGGAATGGATTGTTGAAGAAAGCTAAGGAGCTTGCGATTCTCTGCG
ATGCTGAGGTTGGTGTCATCATCTTCTCCAGCACCGGTAGGCTCTACGATTTCTCCAGCT
CCAGCATGAAATCGGTCATAGAGAGATACAGCGATGCCAAAGGAGAAACCAGTTCAGA
AAATGATCCCGCTTCAGAAATTCAGGAAATGTATATAGTAACTCTGGAAAAGTATGCATA
TTCAGAAGAGCTTGTACTTGACAGGCAAATGATGGGGGAGGAGCTCTCTGGACTAAGT
GTAGAAGCTTTACAGAATTTGGAAAATCAGCTTGAATTGAGCCTTCGTGGCGTTCGAAT
GAAAAAGGATCAAATGTTAATCGAAGAAATACAAGTACTTAACCGAGAGGGGAATCTCG
TTCACCAAGAGAATTTAGACCTCCACAAGAAAGTAAACCTAATGCACCAACAGAACATG
GAACTACATGAAAAGGTTTCAGAGGTCGAGGGTGTGAAAATCGCAAACAAGAATTCTC
TTCTCACAAATGGTCTAGACATGAGAGATACCTCGAACGAACATGTCCATCTTCAGCTCA
GCCAACCGCAGCATGATCATGAGACGCATTCAAAAGCTATCCAACTCAACTATTTTTCCT
TCATTGCATAA

>AT3G26810|AT3G26810.1

ATGAATTATTTCCCAGATGAAGTAATAGAGCATGTATTCGACTTTGTAACATCTCACAAAG
ACAGGAATGCTATATCTCTTGTATGCAAATCATGGTACAAGATTGAAAGATACAGTAGGC
AAAAGGTTTTCATTGGAAACTGTTATGCCATTAATCCAGAGAGGTTGCTTCGGAGATTCC
CATGTCTAAAGTCTTTGACTTTGAAAGGAAAACCTCATTTTGCGGATTTCAATTTGGTTC
CTCATGAATGGGGAGGTTTTGTGCTACCTTGGATTGAGGCTTTGGCTAGAAGCCGTGTAG
GACTTGAAGAGCTTAGGTTGAAGAGGATGGTTGTTACTGATGAGAGTCTTGAGCTGCTT
TCTCGTTCTTTTGTCAATTTTAAGTCTTTGGTCCTTGTTAGCTGTGAAGGTTTTACCACTG
ATGGTCTTGCCTCTATTGCCGCTAATTGCAGGCATCTTCGGGATCTTGATTTGCAAGAGA
ATGAAATCGATGATCATAGAGGTCAATGGTTAAGTTGTTTCCCAGACACTTGCACGACTC
TTGTCACGCTAAACTTTGCGTGCCTCGAAGGAGAAACTAATCTGGTTGCTCTAGAGAGG
CTTGTTGCTAGGTCTCCAAACCTAAAGAGTCTGAAGCTAAATCGTGCAGTACCGTTAGAT
GCACTCGCAAGGTTAATGGCGTGTGCGCCGCAGATAGTTGACTTAGGAGTAGGGTCTTA
TGAGAATGACCCAGATTCCGAGTCTTACTTGAAACTCATGGCTGTCATAAAGAAATGCA
CCTCGTTGAGGAGTTTGTCGGGTTTTCTAGAGGCTGCTCCTCACTGTCTCTCAGCTTTCC
ACCCAATATGTCATAACCTCACCTCCTTGAATCTTAGTTACGCAGCTGAGATTCATGGTAG
CCACCTTATTAAGCTTATTCAGCATTGCAAGAAACTTCAGCGGTTATGGATTTTGGATAGT
ATAGGTGACAAAGGGCTTGAAGTTGTAGCTTCTACATGTAAAGAGTTACAAGAGCTTAG
GGTTTTTCCATCTGATTTACTCGGTGGAGGCAACACAGCTGTGACCGAAGAAGGTCTAG
TTGCCATCTCGGCAGGCTGCCCTAAGCTCCACTCTATACTCTACTTCTGCCAACAAATGA
CAAACGCAGCTCTCGTAACCGTTGCCAAGAACTGTCCAAATTTCATCCGTTTCCGACTCT
GCATCCTCGAGCCAAACAAACCCGATCACGTCACATCTCAACCTCTAGACGAAGGCTTT
GGAGCAATCGTCAAAGCCTGCAAGAGCCTGAGAAGGCTTTCTCTCTCAGGTCTCCTTAC
AGACCAAGTCTTCCTCTACATCGGAATGTACGCGAATCAGCTCGAGATGCTCTCCATAGC
CTTTGCAGGAGATACAGACAAAGGCATGCTATATGTGTTGAATGGTTGCAAAAAGATGA
AGAAACTAGAGATAAGGGATAGTCCGTTTGGGGACACGGCGCTTCTTGCTGATGTGAGC
AAGTATGAAACAATGCGATCCCTTTGGATGTCTTCATGTGAAGTCACACTCAGTGGATGC
AAAAGGCTCGCAGAGAAAGCGCCATGGCTCAATGTAGAGATCATAAACGAGAATGATAA
TAACCGGATGGAAGAAAACGGACACGAGGGGAGGCAGAAAGTGGATAAGTTGTATCTG
TACCGGACTGTGGTTGGGACAAGAATGGATGCGCCGCCATTTGTGTGGATTCTCTAA

>AT5G61430|AT5G61430.1

ATGGAGACTTTTTGTGGGTTTCAAAAGGAGGAAGAGCAGATGGATTTACCTCCTGGGTT

CAGGTTTCATCCAACAGATGAAGAACTCATAACTCACTATCTCCATAAGAAGGTTCTTGA
CACCAGCTTCTCAGCTAAAGCTATCGGTGAAGTTGATTTAAACAAATCAGAGCCATGGG
AGTTACCATGGATGGCAAAAATGGGTGAGAAAGAATGGTATTTTTTCTGTGTGAGAGAC
AGAAAGTATCCCACCGGTTTAAGAACTAACCGAGCAACTGAAGCCGGTTATTGGAAGGC
GACCGGGAAGGATAAAGAGATATACCGAGGCAAATCACTTGTTGGGATGAAGAAGACA
CTTGTTTTCTATAGAGGAAGAGCTCCTAAAGGTCAGAAAACCAACTGGGTGATGCATGA
GTACAGGCTTGAAGGAAAATTCTCTGCCCATAACTTGCCGAAAACCGCAAAGAATGAAT
GGGTGATATGCAGGGTGTTCCAAAAGAGTGCTGGAGGGAAGAAGATCCCGATTTCGAG
TCTAATCCGAATCGGTTCACTCGGAACCGACTTTAACCCTTCGCTTTTGCCCTCTTTAAC
CGATTCTTCGCCTTACAACGATAAAACCAAAACAGAACCGGTCTACGTGCCCTGCTTCT
CCAACCAAACGGATCAAAACCAAGGAACCACACTCAATTGCTTCAGCAGCCCTGTTCTT
AACTCGATCCAAGCCGACATTTTTCACAGGATTCCACTCTATCAAACTCAGTCCCTCCAG
GTTTCTATGAATCTACAGAGCCCGGTTCTCACGCAAGAACACTCAGTTCTACATGCTATG
ATCGAGAACAACAGAAGACAAAGTCTCAAAACGATGAGTGTCTCACAAGAAACCGGA
GTTTCAACTGACATGAACACTGATATCTCATCGGATTTTGAATTTGGTAAGAGACGGTTT
GATTCTCAAGAAGATCCGTCTTCCTCTACTGGACCGGTTGATCTTGAACCTTTCTGGAAT
TACTGA

>AT5G67180|AT5G67180.1

ATGTGGAACCTTAACGACTCACCCGATCATCACGAAGAATCCGACAGTAGAGGGAATCC
GGTCGGACACGTGTCAAACGGGATGAGTCAATCTGCAACGTGGCTACCGTTTGTGCTTC
CGGTGACCAGGAATTTCTTTCCGGCTCAAAGCATGGAACCGGGAGTTCGTTGGTCCGGT
TTCAATAGTGTCGGTAAGTCGGATCCAAGCGGATCGGGTCGACCAGAAGAGCCGGAGAT
ATCACCGCCGATAAAGAAGAGCCGACGTGGTCCTCGCTCACGTAGCTCTCAGTATCGAG
GAGTTACGTTTTACCGACGAACCGGAAGATGGGAGTCACATATTTGGGACTGTGGGAAG
CAAGTGTACTTAGGTGGATTTGACACTGCACATGCTGCTGCTCGAGCCTATGATCGAGCG
GCGATTAAATTCAGAGGTGTAGATGCAGATATTAATTTCGACATTGAAGACTACCTTGAC
GATTTGAAACAGATGGGAAATTTGACAAAGGAAGAGTTTATGCATGTACTTAGAAGGCA
AAGCACTGGGTTTCCAAGAGGCAGTTCTAAATACAGAGGTGTTACTTTGCACAAATGTG
GAAGATGGGAGTCACGTTTGGGTCAATTCCTCAACAAAAAGTACGTTTATTTGGGTCTC
TTTGATACCGAGATTGAAGCTGCTAGAGCTTATGATAAAGCGGCAATAAAGTGTAATGGG
AAAGACGCTGTTACCAATTTTGACCCTAAAGTATACGAGGAAGAAGAAGATCTTAGCTC
AGAGACGACGAGGAACGGTCATAATCTTGGTCTAAGTCTAGGAGAATCATCATCTGAAG
AGTTTAGACTCAAGTCTGATATTGCAAGCATAAGAAGTAGGATTAGAGATGAGGAGAGA
TTATTAGGGAGCGATCTATCGCTAGCAATGATGACGACGACTGTCAGATCGGAAAAGCA
GCAATCGGACGGTGGAGGAAATCGAGTGGTGGGAATGGCAGCATCATCAGGATTCTCTC
CTCAACCTTCCCCTTACCGCATTCCTCGCACCTTCCACTTCTCTCGTCCCTGA

>ENSG00000017427|ENST00000307046

ATGGGAAAAATCAGCAGTCTTCCAACCCAATTATTTAAGTGCTGCTTTTGTGATTTCTTG
AAGGTGAAGATGCACACCATGTCCTCCTCGCATCTCTTCTACCTGGCGCTGTGCCTGCTC
ACCTTCACCAGCTCTGCCACGGCTGGACCGGAGACGCTCTGCGGGGCTGAGCTGGTGG
ATGCTCTTCAGTTCGTGTGTGGAGACAGGGGCTTTTATTTCAACAAGCCCACAGGGTAT
GGCTCCAGCAGTCGGAGGGCGCCTCAGACAGGCATCGTGGATGAGTGCTGCTTCCGGA
GCTGTGATCTAAGGAGGCTGGAGATGTATTGCGCACCCCTCAAGCCTGCCAAGTCAGCT
CGCTCTGTCCGTGCCCAGCGCCACACCGACATGCCCAAGACCCAGAAGTATCAGCCCCC
ATCTACCAACAAGAACACGAAGTCTCAGAGAAGGAAAGGTTGGCCAAAGACACATCCA
GGAGGGGAACAGAAGGAGGGGACAGAAGCAAGTCTGCAGATCAGAGGAAAGAAGAA
AGAGCAGAGGAGGGAGATTGGAAGTAGAAATGCTGAATGCAGAGGCAAAAAAGGAAA
ATGA

>ENSG00000017427|ENST00000337514

ATGGGAAAAATCAGCAGTCTTCCAACCCAATTATTTAAGTGCTGCTTTTGTGATTTCTTG
AAGGTGAAGATGCACACCATGTCCTCCTCGCATCTCTTCTACCTGGCGCTGTGCCTGCTC
ACCTTCACCAGCTCTGCCACGGCTGGACCGGAGACGCTCTGCGGGGCTGAGCTGGTGG
ATGCTCTTCAGTTCGTGTGTGGAGACAGGGGCTTTTATTTCAACAAGCCCACAGGGTAT
GGCTCCAGCAGTCGGAGGGCGCCTCAGACAGGCATCGTGGATGAGTGCTGCTTCCGGA
GCTGTGATCTAAGGAGGCTGGAGATGTATTGCGCACCCCTCAAGCCTGCCAAGTCAGCT
CGCTCTGTCCGTGCCCAGCGCCACACCGACATGCCCAAGACCCAGAAGGAAGTACATTT
GAAGAACGCAAGTAGAGGGAGTGCAGGAAACAAGAACTACAGGATGTAG

>ENSG00000017427|ENST00000392904

ATGGGAAAAATCAGCAGTCTTCCAACCCAATTATTTAAGTGCTGCTTTTGTGATTTCTTG
AAGGTGAAGATGCACACCATGTCCTCCTCGCATCTCTTCTACCTGGCGCTGTGCCTGCTC
ACCTTCACCAGCTCTGCCACGGCTGGACCGGAGACGCTCTGCGGGGCTGAGCTGGTGG
ATGCTCTTCAGTTCGTGTGTGGAGACAGGGGCTTTTATTTCAACAAGCCCACAGGGTAT
GGCTCCAGCAGTCGGAGGGCGCCTCAGACAGGCATCGTGGATGAGTGCTGCTTCCGGA
GCTGTGATCTAAGGAGGCTGGAGATGTATTGCGCACCCCTCAAGCCTGCCAAGTCAGCT
CGCTCTGTCCGTGCCCAGCGCCACACCGACATGCCCAAGACCCAGAAGTATCAGCCCCC
ATCTACCAACAAGAACACGAAGTCTCAGAGAAGGAAAGGAAGTACATTTGAAGAACGC
AAGTAG

>ENSG00000082258|ENST00000264157

ATGGCGTCGGGCCGTGGAGCTTCTTCTCGCTGGTTCTTTACTCGGGAACAGCTGGAGAA
CACGCCGAGCCGCCGCTGCGGAGTGGAGGCGGATAAAGAGCTCTCGTGCCGCCAGCAG
GCGGCCAACCTCATCCAGGAGATGGGACAGCGTCTCAATGTCTCTCAGCTTACAATAAA
CACTGCGATTGTTTATATGCACAGGTTTTATATGCACCATTCTTTCACCAAATTCAACAAA
AATATAATATCGTCTACTGCATTATTTTTGGCTGCAAAAGTGGAAGAACAGGCTCGAAAA
CTTGAACATGTTATCAAAGTAGCACATGCTTGTCTTCATCCTCTAGAGCCACTGCTGGAT
ACTAAATGTGATGCTTACCTTCAACAGACTCAAGAACTGGTTATACTTGAAACCATAATG
CTACAAACTCTAGGTTTTGAGATCACCATTGAACACCCACACACAGATGTGGTGAAATG
TACCCAGTTAGTAAGAGCAAGCAAGGATTTGGCACAGACATCCTATTTCATGGCTACCAA
CAGTCTGCATCTTACAACCTTCTGTCTTCAGTACAAACCAACAGTGATAGCATGTGTATG
CATTCATTTGGCTTGCAAATGGTCCAATTGGGAGATCCCTGTATCAACTGATGGAAAGCA
TTGGTGGGAATATGTGGATCCTACAGTTACTCTAGAATTATTAGATGAGCTAACACATGA
GTTTCTACAAATATTGGAGAAAACGCCTAATAGGTTGAAGAAGATTCGAAACTGGAGGG
CTAATCAGGCAGCTAGGAAACCAAAAGTAGATGGACAGGTATCAGAGACACCACTTCTT
GGTTCATCTTTGGTCCAGAATTCCATTTTAGTAGATAGTGTCACTGGTGTGCCTACAAAC
CCAAGTTTTCAGAAACCATCTACATCAGCATTCCCTGCGCCAGTACCTCTAAATTCAGGA
AATATTTCTGTTCAAGACAGCCATACATCTGATAATTTGTCAATGCTAGCAACAGGAATG
CCAAGTACTTCATACGGTTTATCATCACACCAGGAATGGCCTCAACATCAAGACTCAGCA
AGGACAGAACAGCTATATTCACAGAAACAGGAGACATCTTTGTCTGGTAGCCAGTACAA
CATCAACTTCCAGCAGGGACCTTCTATATCACTGCATTCAGGATTACATCACAGACCTGA
CAAAATTTCAGATCATTCTTCTGTTAAGCAAGAATATACTCATAAAGCAGGGAGCAGTAA
ACACCATGGGCCAATTTCCACTACTCCAGGAATAATTCCTCAGAAAATGTCTTTAGATAA
ATATAGAGAAAAGCGTAAACTAGAAACTCTTGATCTCGATGTAAGGGATCATTATATAGC
TGCCCAGGTAGAACAGCAGCACAAACAAGGGCAGTCACAGGCAGCCAGCAGCAGTTC
TGTTACTTCTCCCATTAAAATGAAAATACCTATCGCAAATACTGAAAAATACATGGCAGA
CAAAAAGGAAAAGAGTGGGTCACTGAAATTACGGATTCCAATACCACCCACTGATAAAA

GCGCCAGTAAAGAAGAACTGAAAATGAAAATAAAAGTTTCTTCTTCAGAAAGACACAG
CTCTTCTGATGAAGGCAGTGGGAAGAGCAAACATTCAAGCCCACATATTAGCAGAGACC
ATAAGGAGAAGCACAAGGAGCATCCTTCAAGCCGCCACCACACCAGCAGCCACAAGCA
TTCCCACTCGCATAGTGGCAGCAGCAGCGGTGGCAGTAAACACAGTGCCGACGGAATA
CCACCCACTGTTCTGAGGAGTCCTGTTGGCCTGAGCAGTGATGGCATTTCCTCTAGCTCC
AGCTCTTCAAGGAAGAGGCTGCATGTCAATGATGCATCTCACAACCACCACTCCAAAAT
GAGCAAAAGTTCCAAAAGTTCAGGTAGTTCATCTAGTTCTTCCTCCTCTGTTAAGCAGTA
TATATCCTCTCACAACTCTGTTTTTAACCATCCCTTACCCCCTCCTCCCCCTGTCACATACC
AGGTGGGCTACGGACATCTCAGCACCCTCGTGAAACTGGACAAGAAGCCAGTGGAGAC
CAACGGTCCTGATGCCAATCACGAGTACAGTACAAGCAGCCAGCATATGGACTACAAAG
ACACATTCGACATGCTGGACTCACTGTTAAGTGCCCAAGGAATGAACATGTAA

>ENSG00000082258|ENST00000295238

ATGGCGTCGGGCCGTGGAGCTTCTTCTCGCTGGTTCTTTACTCGGGAACAGCTGGAGAA
CACGCCGAGCCGCCGCTGCGGAGTGGAGGCGGATAAAGAGCTCTCGTGCCGCCAGCAG
GCGGCCAACCTCATCCAGGAGATGGGACAGCGTCTCAATGTCTCTCAGCTTACAATAAA
CACTGCGATTGTTTATATGCACAGGTTTTATATGCACCATTCTTTCACCAAATTCAACAAA
AATATAATATCGTCTACTGCATTATTTTTGGCTGCAAAAGTGGAAGAACAGGCTCGAAAA
CTTGAACATGTTATCAAAGTAGCACATGCTTGTCTTCATCCTCTAGAGCCACTGCTGGAT
ACTAAATGTGATGCTTACCTTCAACAGACTCAAGAACTGGTTATACTTGAAACCATAATG
CTACAAACTCTAGGTTTTGAGATCACCATTGAACACCCACACACAGATGTGGTGAAATG
TACCCAGTTAGTAAGAGCAAGCAAGGATTTGGCACAGACATCCTATTTCATGGCTACCAA
CAGTCTGCATCTTACAACCTTCTGTCTTCAGTACAAACCAACAGTGATAGCATGTGTATG
CATTCATTTGGCTTGCAAATGGTCCAATTGGGAGATCCCTGTATCAACTGATGGAAAGCA
TTGGTGGGAATATGTGGATCCTACAGTTACTCTAGAATTATTAGATGAGCTAACACATGA
GTTTCTACAAATATTGGAGAAAACGCCTAATAGGTTGAAGAAGATTCGAAACTGGAGGG
CTAATCAGGCAGCTAGGAAACCAAAAGTAGATGGACAGGTATCAGAGACACCACTTCTT
GGTTCATCTTTGGTCCAGAATTCCATTTTAGTAGATAGTGTCACTGGTGTGCCTACAAAC
CCAAGTTTTCAGAAACCATCTACATCAGCATTCCCTGCGCCAGTACCTCTAAATTCAGGA
AATATTTCTGTTCAAGACAGCCATACATCTGATAATTTGTCAATGCTAGCAACAGGAATG
CCAAGTACTTCATACGGTTTATCATCACACCAGGAATGGCCTCAACATCAAGACTCAGCA
AGGACAGAACAGCTATATTCACAGAAACAGGAGACATCTTTGTCTGGTAGCCAGTACAA
CATCAACTTCCAGCAGGGACCTTCTATATCACTGCATTCAGGATTACATCACAGACCTGA
CAAAATTTCAGATCATTCTTCTGTTAAGCAAGAATATACTCATAAAGCAGGGAGCAGTAA
ACACCATGGGCCAATTTCCACTACTCCAGGAATAATTCCTCAGAAAATGTCTTTAGATAA
ATATAGAGAAAAGCGTAAACTAGAAACTCTTGATCTCGATGTAAGGGATCATTATATAGC
TGCCCAGGTAGAACAGCAGCACAAACAAGGGCAGTCACAGGCAGCCAGCAGCAGTTC
TGTTACTTCTCCCATTAAAATGAAAATACCTATCGCAAATACTGAAAAATACATGGCAGA
CAAAAAGGAAAAGAGTGGGTCACTGAAATTACGGATTCCAATACCACCCACTGATAAAA
GCGCCAGTAAAGAAGAACTGAAAATGAAAATAAAAGTTTCTTCTTCAGAAAGACACAG
CTCTTCTGATGAAGGCAGTGGGAAGAGCAAACATTCAAGCCCACATATTAGCAGAGACC
ATAAGGAGAAGCACAAGGAGCATCCTTCAAGCCGCCACCACACCAGCAGCCACAAGCA
TTCCCACTCGCATAGTGGCAGCAGCAGCGGTGGCAGTAAACACAGTGCCGACGGAATA
CCACCCACTGTTCTGAGGAGTCCTGTTGGCCTGAGCAGTGATGGCATTTCCTCTAGCTCC
AGCTCTTCAAGGAAGAGGCTGCATGTCAATGATGCATCTCACAACCACCACTCCAAAAT
GAGCAAAAGTTCCAAAAGTTCAGGTGGGCTACGGACATCTCAGCACCCTCGTGAAACT
GGACAAGAAGCCAGTGGAGACCAACGGTCCTGA

>ENSG00000082258|ENST00000417175

ATGGCGTCGGGCCGTGGAGCTTCTTCTCGCTGGTTCTTTACTCGGGAACAGCTGGAGAA

CACGCCGAGCCGCCGCTGCGGAGTGGAGGCGGATAAAGAGCTCTCGTGCCGCCAGCAG
GCGGCCAACCTCATCCAGGAGATGGGACAGCGTCTCAATGTCTCTCAGCTTACAATAAA
CACTGCGATTGTTTATATGCACAGGTTTTATATGCACCATTCTTTCACCAAATTCAACAAA
AATATAATATCGTCTACTGCATTATTTTTGGCTGCAAAAGTGGAAGAACAGGCTCGAAAA
CTTGAACATGTTATCAAAGTAGCACATGCTTGTCTTCATCCTCTAGAGCCACTGCTGGAT
ACTAAATGTGATGCTTACCTTCAACAGACTCAAGAACTGGTTATACTTGAAACCATAATG
CTACAAACTCTAGGTTTTGAGATCACCATTGAACACCCACACACAGATGTGGTGAAATG
TACCCAGTTAGTAAGAGGAGAATGCTTTATTGCAACTTTTAGCTGA

>ENSG00000082258|ENST00000419781

ATGGCGTCGGGCCGTGGAGCTTCTTCTCGCTGGTTCTTTACTCGGGAACAGCTGGAGAA
CACGCCGAGCCGCCGCTGCGGAGTGGAGGCGGATAAAGAGCTCTCGTGCCGCCAGCAG
GCGGCCAACCTCATCCAGGAGATGGGACAGCGTCTCAATGTCTCTCAGCTTACAATAAA
CACTGCGATTGTTTATATGCACAGGTTTTATATGCACCATTCTTTCACCAAATTCAACAAA
AATATAATATCGTCTACTGCATTATTTTTGGCTGCAAAAGTGGAAGAACAGGCTCGAAAA
CTTGAACATGTTATCAAAGTAGCACATGCTTGTCTTCATCCTCTAGAGCCACTGCTGGAT
ACTAAATGTGATGCTTACCTTCAACAGACTCAAGAACTGGTTATACTTGAAACCATAATG
CTACAAACTCTAGGTTTTGAGATCACCATTGAACACCCACACACAGATGTGGTGAAATG
TACCCAGTTAGTAAGAGATCTTTGGGGGATAGCAAGAAACATTTGA

>ENSG00000082258|ENST00000438691

NNGATCCCTGTATCAACTGATGGAAAGCATTGGTGGGAATATGTGGATCCTACAGTTACT
CTAGAATTATTAGATGAGCTAACACATGAGTTTCTACAAATATTGGAGAAAACGCCTAAT
AGGTTGAAGAAGATTCGAAACTGGAGGGTAAGAGAATTGACAGGGTTTAACAGAATTT
CAGTCTTTTATGTAACATTTACATAG

>ENSG00000102554|ENST00000377687

ATGGCTACAAGGGTGCTGAGCATGAGCGCCCGCCTGGGACCCGTGCCCCAGCCGCCGG
CGCCGCAGGACGAGCCGGTGTTCGCGCAGCTCAAGCCGGTGCTGGGCGCCGCGAATCC
GGCCCGCGACGCGGCGCTCTTCCCCGGCGAGGAGCTGAAGCACGCGCACCACCGCCCG
CAGGCGCAGCCCGCGCCCGCGCAGGCCCCGCAGCCGGCCCAGCCGCCCGCCACCGGCC
CGCGGCTGCCTCCAGAGGACCTGGTCCAGACAAGATGTGAAATGGAGAAGTATCTGAC
ACCTCAGCTTCCTCCAGTTCCTATAATTCCAGAGCATAAAAAGTATAGACGAGACAGTGC
CTCAGTCGTAGACCAGTTCTTCACTGACACTGAAGGGTTACCTTACAGTATCAACATGA
ACGTCTTCCTCCCTGACATCACTCACCTGAGAACTGGCCTCTACAAATCCCAGAGACCG
TGCGTAACACACATCAAGACAGAACCTGTTGCCATTTTCAGCCACCAGAGTGAAACGAC
TGCCCCTCCTCCGGCCCCGACCCAGGCCCTCCCTGAGTTCACCAGTATATTCAGCTCACA
CCAGACCGCAGCTCCAGAGGTGAACAATATTTTCATCAAACAAGAACTTCCTACACCAG
ATCTTCATCTTTCTGTCCCTACCCAGCAGGGCCACCTGTACCAGCTACTGAATACACCGG
ATCTAGATATGCCCAGTTCTACAAATCAGACAGCAGCAATGGACACTCTTAATGTTTCTAT
GTCAGCTGCCATGGCAGGCCTTAACACACACACCTCTGCTGTTCCGCAGACTGCAGTGA
AACAATTCCAGGGCATGCCCCCTTGCACATACACAATGCCAAGTCAGTTTCTTCCACAA
CAGGCCACTTACTTTCCCCCGTCACCACCAAGCTCAGAGCCTGGAAGTCCAGATAGACA
AGCAGAGATGCTCCAGAATTTAACCCCACCTCCATCCTATGCTGCTACAATTGCTTCTAA
ACTGGCAATTCACAATCCAAATTTACCCACCACCCTGCCAGTTAACTCACAAAACATCC
AACCTGTCAGATACAATAGAAGGAGTAACCCCGATTTGGAGAAACGACGCATCCACTAC
TGCGATTACCCTGGTTGCACAAAAGTTTATACCAAGTCTTCTCATTTAAAAGCTCACCTG
AGGACTCACACTGGTGAAAAGCCATACAAGTGTACCTGGGAAGGCTGCGACTGGAGGT
TCGCGCGATCGGATGAGCTGACCCGCCACTACCGGAAGCACACAGGCGCCAAGCCCTT

CCAGTGCGGGGTGTGCAACCGCAGCTTCTCGCGCTCTGACCACCTGGCCCTGCATATGA
AGAGGCACCAGAACTGA

>ENSG00000104131|ENST00000261868

ATGGCGGCGGCGGCGGCGGCGGCGGGGGACTCGGACTCCTGGGACGCCGACGCTTTCT
CCGTGGAAGACCCAGTGCGGAAGGTGGGGGGCGGCGGCACTGCCGGCGGGGACCGCT
GGGAAGGCGAGGACGAGGACGAGGACGTCAAGGATAACTGGGATGACGATGATGATGA
AAAAAAAGAGGAAGCAGAAGTAAAACCAGAGGTAAAAATTTCAGAAAAGAAAAAAAT
AGCAGAGAAGATAAAAGAGAAAGAACGGCAACAGAAGAAAAGGCAAGAAGAAATTA
AAAAGAGGTTAGAAGAACCCGAAGAACCTAAAGTGCTAACACCAGAAGAACAATTAGC
AGATAAACTGCGGCTAAAGAAATTACAGGAAGAGTCAGACCTCGAATTAGCAAAGGAA
ACTTTTGGTGTTAATAATGCAGTTTATGGAATAGATGCTATGAACCCATCTTCAAGAGATG
ACTTTACAGAGTTTGGAAAGTTACTAAAAGATAAAATTACACAATATGAAAAGTCACTAT
ATTATGCCAGTTTTTTGGAAGTCTTAGTTCGAGATGTGTGTATTTCATTGGAAATTGATGA
CTTGAAAAAAATTACCAATTCACTGACTGTGCTTTGCAGTGAAAAACAGAAGCAAGAA
AAGCAAAGCAAAGCCAAAAAGAAGAAGAAAGGTGTGGTTCCTGGAGGGGGATTAAAA
GCCACCATGAAAGATGATCTGGCAGATTATGGTGGTTATGATGGAGGATATGTACAAGAC
TATGAAGACTTCATGTGA

>ENSG00000104131|ENST00000424492

ATGGCGGCGGCGGCGGCGGCGGCGGGGGACTCGGACTCCTGGGACGCCGACGCTTTCT
CCGTGGAAGACCCAGTGCGGAAGGTGGGGGGCGGCGGCACTGCCGGCGGGGACCGCT
GGGAAGGCGAGGACGAGGACGAGGACGTCAAGTTAGAAGAACCCGAAGAACCTAAAG
TGCTAACACCAGAAGAACAATTAGCAGATAAACTGCGGCTAAAGAAATTACAGGAAGA
GTCAGACCTCGAATTAGCAAAGGAAACTTTTGGTGTTAATAATGCAGTTTATGGAATAGA
TGCTATGAACCCATCTTCAAGAGATGACTTTACAGAGTTTGGAAAGTTACTAAAAGATAA
AATTACACAATATGAAAAGTCACTATATTATGCCAGTTTTTTGGAAGTCTTAGTTCGAGAT
GTGTGTATTTCATTGGAAATTGATGACTTGAAAAAAATTACCAATTCACTGACTGTGCTT
TGCAGTGAAAAACAGAAGCAAGAAAAGCAAAGCAAAGCCAAAAAGAAGAAGAAAGG
TGTGGTTCCTGGAGGGGGATTAAAAGCCACCATGAAAGATGATCTGGCAGATTATGGTG
GTTATGATGGAGGATATGTACAAGACTATGAAGACTTCATGTGA

>ENSG00000104375|ENST00000419617

ATGGAGCAGCCGCCGGCGCCTAAGAGTAAACTAAAAAAGCTGAGTGAAGACAGTTTGA
CTAAGCAGCCTGAAGAAGTTTTTGATGTATTAGAGAAGCTTGGAGAAGGGTCTTATGGA
AGTGTATTTAAAGCAATACACAAGGAATCCGGTCAAGTTGTCGCAATTAAACAAGTACC
TGTTGAATCAGATCTTCAGGAAATAATCAAAGAAATTTCCATAATGCAGCAATGTGACAG
CCCATATGTTGTAAAGTACTATGGCAGTTATTTTAAGAATACAGACCTCTGGATTGTTATG
GAGTACTGTGGCGCTGGCTCTGTCTCAGACATAATTAGATTACGAAACAAGACATTAATA
GAAGATGAAATTGCAACCATTCTTAAATCTACATTGAAAGGACTAGAATATTTGCACTTTA
TGAGAAAAATACACAGAGATATAAAAGCTGGAAATATTCTCCTCAATACAGAAGGACAT
GCAAAATTGGCAGATTTTGGAGTGGCTGGTCAGTTAACAGATACAATGGCAAAACGCAA
TACTGTAATAGGAACTCCATTTTGGATGGCTCCTGAGGTGATTCAAGAAATAGGCTATAA
CTGTGTGGCCGACATCTGGTCCCTTGGCATTACTTCTATAGAAATGGCTGAAGGAAAACC
TCCTTATGCTGATATACATCCAATGAGGGCTATTTTTATGATTCCCACAAATCCACCACCA
ACATTCAGAAAGCCAGAACTTTGGTCCGATGATTTCACCGATTTTGTTAAAAAGTGTTTG
GTGAAGAATCCTGAGCAGAGAGCTACTGCAACACAACTTTTACAGCATCCTTTTATCAA
GAATGCCAAACCTGTATCAATATTAAGAGACCTGATCACAGAAGCTATGGAGATCAAAG
CTAAAAGACATGAGGAACAGCAACGAGAATTGGAAGAGGAAGAAGAAAATTCGGATG

AAGATGAGCTGGATTCCCACACCATGGTGAAGACTAGTGTGGAGAGTGTGGGCACCATG
CGGGCCACAAGCACGATGAGTGAAGGGGCCCAGACCATGATTGAACATAATAGCACGAT
GTTGGAATCCGACTTGGGGACCATGGTGATAAACAGTGAGGATGAGGAAGAAGAAGAT
GGAACTATGAAAAGAAATGCAACCTCACCACAAGTACAAAGACCATCTTTCATGGACTA
CTTTGATAAGCAAGACTTCAAGAATAAGAGTCACGAAAACTGTAATCAGAACATGCATG
AACCCTTCCCTATGTCCAAAAACGTTTTTCCTGATAACTGGAAAGTTCCTCAAGATGGAG
ACTTTGACTTTTTGAAAAATCTAAGTTTAGAAGAACTACAGATGCGGTTAAAAGCACTG
GACCCCATGATGGAACGGGAGATAGAAGAACTTCGTCAGAGATACACTGCGAAAAGAC
AGCCCATTCTGGATGCGATGGATGCAAAGAAAAGAAGGCAGCAAAACTTTTGA

>ENSG00000110917|ENST00000228506

ATGCTGGGAGCCTGGGCGGTTGAGGGAACCGCTGTGGCGCTCCTGCGACTGCTGCTGC
TGCTGCTGCCGCCGGCGATCCGGGGACCCGGGCTCGGCGTGGCCGGCGTGGCCGGCGC
GGCGGGGGCCGGGCTGCCCGAGAGCGTCATTTGGGCGGTCAACGCGGGTGGAGAGGC
GCATGTGGACGTGCACGGGATCCACTTCCGCAAGGACCCTTTGGAAGGCCGGGTGGGC
CGAGCCTCAGACTATGGCATGAAACTGCCAATCCTGCGTTCCAACCCTGAGGACCAGAT
CCTGTATCAAACTGAGCGGTACAATGAGGAGACCTTTGGCTACGAAGTGCCCATCAAAG
AGGAGGGGGACTACGTGCTGGTCTTGAAATTTGCAGAGGTCTACTTTGCACAGTCCCAG
CAAAAGGTATTTGATGTACGATTGAATGGCCACGTCGTGGTGAAGGACTTGGATATCTTT
GATCGTGTTGGGCATAGCACAGCTCACGATGAAATTATACCTATGAGCATCAGAAAGGG
GAAGCTGAGTGTCCAGGGGGAGGTGTCCACCTTCACAGGGAAACTCTACATTGAGTTT
GTCAAGGGGTACTATGACAATCCCAAGGTCTGTGCACTCTACATCATGGCTGGGACAGT
GGATGATGTACCAAAGCTTCAGCCTCATCCGGGATTGGAGAAGAAAGAAGAGGAAGAA
GAAGAAGAAGAATATGATGAAGGGTCTAATCTCAAAAAACAGACCAATAAGAACCGGG
TGCAGTCAGGCCCCCGCACACCCAACCCCTATGCCTCGGACAACAGCAGCCTCATGTTT
CCCATCCTGGTGGCCTTCGGAGTCTTCATTCCAACCCTCTTCTGCCTCTGCCGGTTGTGA

>ENSG00000138594|ENST00000308580

ATGGCACTGCCATTCCGTAAGGACTTAGAAAAGTACAAAGACCTTGATGAAGATGAGCT
CCTTGGGAATCTGTCAGAAACAGAACTGAAACAACTGGAAACTGTTTTGGATGATCTTG
ACCCCGAGAATGCCCTTCTGCCTGCAGGGTTCCGGCAGAAGAACCAGACATCAAAGTC
CACCACAGGGCCATTTGATAGAGAGCATCTCCTTTCATATCTGGAGAAAGAAGCATTGG
AGCATAAAGACAGGGAAGACTATGTGCCCTACACTGGAGAAAAAAAAGGGAAAATATT
TATCCCCAAACAGAAACCTGTACAGACTTTTACAGAAGAAAAAGTGTCTCTTGATCCAG
AATTAGAAGAAGCTTTGACAAGTGCTTCTGATACAGAATTGTGTGACCTCGCAGCAATT
CTTGGGATGCACAATTTGATAACGAATACAAAGTTCTGTAATATAATGGGAAGTAGTAAT
GGTGTTGACCAAGAACATTTTTCAAATGTGGTCAAAGGTGAAAAGATTCTTCCGGTATTT
GATGAGCCACCAAATCCAACCAATGTAGAAGAGAGTTTGAAGAGAACTAAAGAAAACG
ATGCTCATCTTGTTGAAGTTAATTTGAATAATATAAAGAATATCCCAATTCCAACCCTAAA
AGATTTTGCAAAGGCTTTGGAAACCAACACACATGTGAAATGTTTCAGTCTTGCAGCCA
CCCGGAGCAATGACCCTGTTGCTACTGCTTTTGCAGAAATGCTGAAAGTGAACAAAACT
TTGAAGAGCTTAAATGTGGAGTCCAACTTTATCACGGGAGTTGGGATTCTGGCACTGATT
GATGCGTTAAGAGATAATGAAACCCTGGCAGAGCTCAAGATTGACAATCAGAGGCAGC
AGTTGGGGACAGCTGTAGAATTGGAAATGGCCAAGATGCTTGAGGAAAATACAAATATC
CTTAAATTTGGATATCAGTTTACACAGCAGGGACCACGAACCAGAGCAGCTAATGCTATA
ACAAAAAACAATGACTTAGTGCGTAAGAGACGAGTTGAAGGAGATCACCAGTAA

>ENSG00000153574|ENST00000543560

GCCCGCCAGCTCATCCTGCAGTATGGCTTGACCCTCAGTGATCTGGATCGACACCCAGA

GATCGACCTTGCCATCGATGGTGCTGATGAAGTAGATGCTGATCTCAATCTCATCAAGGG
TGGCGGAGGCTGCCTGACCCAGGAGAAGATTGTGGCTGGCTATGCTAGTCGCTTCATCG
TGATCGCTGATTTCAGGAAAGATTCGAAGAATCTCGGGGATCAGTGGCACAAGGGAATC
CCCATCGAGGTCATCCCAATGGCCTATGTCCCAGTGAGCCGAGCTGTGAGCCAGAAGTT
TGGGGGCGTGGTTGAACTTCGAATGGCTGTCAACAAGGCTGGTCCTGTGGTGACAGATA
ATGGGAATTTTATCTTGGACTGGAAGTTTGACCGGGTACACAAATGGAGTGAAGTGAAT
ACAGCTATCAAAATGATCCCAGGTGTGGTGGACACAGGCCTATTCATCAACATGGCTGA
GAGAGTCTACTTTGGGATGCAGGATGGCTCAGTGAACATGAGGGAGAAGCCTTTCTGTT
GA

>ENSG00000167588|ENST00000548814

ATGGCTAGCAAGAAAGTCTGCATTGTAGGCTCCGGGAACTGGGGCTCAGCCATCGCCAA
GATCGTGGGTGGCAATGCAGCCCAGCTGGCACAGTTTGACCCACGGGTGACCATGTGG
GTATTTGAGGAAGACATTGGAGGCAAAAAGCTGACTGAGATCATCAACACGCAGCATG
AGAATGTCAAATACCTGCCAGGGCACAAGTTGCCCCCAAATGTGTTCATCGGCAAGATC
TGTGACCAGCTCAAGGGCCATCTGAAGGCAAACGCCACTGGCATATCTCTTATTAAGGG
GGTAGACGAGGGCCCCAATGGGCTGAAGCTCATCTCGGAAGTGATTGGGGAGCGCCTC
GGCATCCCCATGAGTGTGCTGATGGGGGCCAACATTGCCAGCGAGGTGGCTGATGAGAA
GTTCTGTGAGACAACCATTGGCTGCAAGGACCCGGCCCAGGGACAACTCCTGAAAGAG
CTGATGCAGACACCAAACTTCCGTATCACAGTGGTGCAAGAGGTGGACACAGTAGAGA
TCTGTGGAGCCTTAAAGAATGTAGTGGCCGTGGGGGCTGGCTTCTGTGATGGCCTGGGC
TTTGGCGACAACACCAAGGCGGCAGTGATCCGGCTGGGACTCATGGAGATGATAGCCTT
CGCCAAGCTCTTCTGCAGTGGCCCTGTGTCCTCTGCCACCTTCTTGGAGAGCTGTGGTG
TTGCTGACCTGATCACTACCTGCTATGGAGGGCGGAACCGGAAAGTGGCTGAGGCCTTT
GCGCGTACAGGAAAGTCCATTGAGCAGCTGGAGAAAGAGTTGCTGAATGGGCAGAAAC
TGCAGGGGCCCGAGACAGCCCGGGAGCTATACAGCATCCTCCAGCACAAGGGCCTGGT
AGACAAGTTTCCCTTGTTCATGGCTGTGTACAAGGTGTGCTACGAGGGCCAGCCAGTGG
GTGAATTCATCCACTGCCTGCAGAATCATCCAGAACATATGTGA

>ENSG00000169057|ENST00000303391

ATGGTAGCTGGGATGTTAGGGCTCAGGGAAGAAAAGTCAGAAGACCAGGACCTCCAGG
GCCTCAAGGACAAACCCCTCAAGTTTAAAAAGGTGAAGAAAGATAAGAAAGAAGAGA
AAGAGGGCAAGCATGAGCCCGTGCAGCCATCAGCCCACCACTCTGCTGAGCCCGCAGA
GGCAGGCAAAGCAGAGACATCAGAAGGGTCAGGCTCCGCCCCGGCTGTGCCGGAAGC
TTCTGCCTCCCCCAAACAGCGGCGCTCCATCATCCGTGACCGGGGACCCATGTATGATGA
CCCCACCCTGCCTGAAGGCTGGACACGGAAGCTTAAGCAAAGGAAATCTGGCCGCTCT
GCTGGGAAGTATGATGTGTATTTGATCAATCCCCAGGGAAAAGCCTTTCGCTCTAAAGTG
GAGTTGATTGCGTACTTCGAAAAGGTAGGCGACACATCCCTGGACCCTAATGATTTTGAC
TTCACGGTAACTGGGAGAGGGAGCCCCTCCCGGCGAGAGCAGAAACCACCTAAGAAGC
CCAAATCTCCCAAAGCTCCAGGAACTGGCAGAGGCCGGGGACGCCCCAAAGGGAGCG
GCACCACGAGACCCAAGGCGGCCACGTCAGAGGGTGTGCAGGTGAAAAGGGTCCTGG
AGAAAAGTCCTGGGAAGCTCCTTGTCAAGATGCCTTTTCAAACTTCGCCAGGGGGCAA
GGCTGAGGGGGGTGGGGCCACCACATCCACCCAGGTCATGGTGATCAAACGCCCCGGC
AGGAAGCGAAAAGCTGAGGCCGACCCTCAGGCCATTCCCAAGAAACGGGGCCGAAAG
CCGGGGAGTGTGGTGGCAGCCGCTGCCGCCGAGGCCAAAAAGAAAGCCGTGAAGGAG
TCTTCTATCCGATCTGTGCAGGAGACCGTACTCCCCATCAAGAAGCGCAAGACCCGGGA
GACGGTCAGCATCGAGGTCAAGGAAGTGGTGAAGCCCCTGCTGGTGTCCACCCTCGGT
GAGAAGAGCGGGAAAGGACTGAAGACCTGTAAGAGCCCTGGGCGGAAAAGCAAGGAG
AGCAGCCCCAAGGGGCGCAGCAGCAGCGCCTCCTCACCCCCCAAGAAGGAGCACCAC
CACCATCACCACCACTCAGAGTCCCCAAAGGCCCCCGTGCCACTGCTCCCACCCCTGCC

CCCACCTCCACCTGAGCCCGAGAGCTCCGAGGACCCCACCAGCCCCCCTGAGCCCCAG
GACTTGAGCAGCAGCGTCTGCAAAGAGGAGAAGATGCCCAGAGGAGGCTCACTGGAG
AGCGACGGCTGCCCCAAGGAGCCAGCTAAGACTCAGCCCGCGGTTGCCACCGCCGCCA
CGGCCGCAGAAAAGTACAAACACCGAGGGGAGGGAGAGCGCAAAGACATTGTTTCATC
CTCCATGCCAAGGCCAAACAGAGAGGAGCCTGTGGACAGCCGGACGCCCGTGACCGA
GAGAGTTAGCTGA

>ENSG00000169057|ENST00000369964

ATGGTAGCTGGGATGTTAGGGCTCAGGGAAGAAAAGTCAGAAGACCAGGACCTCCAGG
GCCTCAAGGACAAACCCCTCAAGTTTAAAAAGGTGAAGAAAGATAAGAAAGAAGAGA
AAGAGGGCAAGCATGAGCCCGTGCAGCCATCAGCCCACCACTCTGCTGAGCCCGCAGA
GGCAGGCAAAGCAGAGACATCAGAAGGGTCAGGCTCCGCCCCGGCTGTGCCGGAAGC
TTCTGCCTCCCCCAAACAGCGGCGCTCCATCATCCGTGACCGGGGACCCATGTATGATGA
CCCCACCCTGCCTGAAGGCTGGACACGGAAGCTTAAGCAAAGGAAATCTGGCCGCTCT
GCTGGGAAGTATGATGTGTATTTGATCAATCCCCAGGGAAAAGCCTTTCGCTCTAAAGTG
GAGTTGATTGCGTACTTCGAAAAGGTAGGCGACACATCCCTGGACCCTAATGATTTTGAC
TTCACGGTAACTGGGAGAGGGAGCCCCTCCCGGCGAGAGCAGAAACCACCTAAGAAGC
CCAAATCTCCCAAAGCTCCAGGAACTGGCAGAGGCCGGGGACGCCCCAAAGGGAGCG
GCACCACGAGACCCAAGGCGGCCACGTCAGAGGGTGTGCAGGTGAAAAGGGTCCTGG
AGAAAAGTCCTGGGAAGCTCCTTGTCAAGATGCCTTTTCAAACTTCGCCAGGGGGCAA
GGCTGAGGGGGGTGGGGCCACCACATCCACCCAGGTCATGGTGATCAAACGCCCCGGC
AGGAAGCGAAGCCCGAGAGCTCCGAGGACCCCACCAGCCCCCCTGAGCCCCAGGACTT
GA

>ENSG00000169057|ENST00000407218

ATGGTAGCTGGGATGTTAGGGCTCAGGGAAGAAAAGTCAGAAGACCAGGACCTCCAGG
GCCTCAAGGACAAACCCCTCAAGTTTAAAAAGGTGAAGAAAGATAAGAAAGAAGAGA
AAGAGGGCAAGCATGAGCCCGTGCAGCCATCAGCCCACCACTCTGCTGAGCCCGCAGA
GGCAGGCAAAGCAGAGACATCAGAAGGGTCAGGCTCCGCCCCGGCTGTGCCGGAAGC
TTCTGCCTCCCCCAAACAGCGGCGCTCCATCATCCGTGACCGGGGACCCATGTATGATGA
CCCCACCCTGCCTGAAGGCTGGACACGGAAGCTTAAGCAAAGGAAATCTGGCCGCTCT
GCTGGGAAGTATGATGTGTATTTGATCAATCCCCAGGGAAAAGCCTTTCGCTCTAAAGTG
GAGTTGATTGCGTACTTCGAAAAGCTCCAGGAACTGGCAGAGGCCGGGGACGCCCCAA
AGGGAGCGGCACCACGAGACCCAAGGCGGCCACGTCAGAGGGTGTGCAGGTGA

>ENSG00000169057|ENST00000453960

ATGGCCGCCGCCGCCGCCGCCGCGCCGAGCGGAGGAGGAGGAGGAGGCGAGGAGGAG
AGACTGGAAGAAAAGTCAGAAGACCAGGACCTCCAGGGCCTCAAGGACAAACCCCTC
AAGTTTAAAAAGGTGAAGAAAGATAAGAAAGAAGAGAAAGAGGGCAAGCATGAGCCC
GTGCAGCCATCAGCCCACCACTCTGCTGAGCCCGCAGAGGCAGGCAAAGCAGAGACAT
CAGAAGGGTCAGGCTCCGCCCCGGCTGTGCCGGAAGCTTCTGCCTCCCCCAAACAGCG
GCGCTCCATCATCCGTGACCGGGGACCCATGTATGATGACCCCACCCTGCCTGAAGGCT
GGACACGGAAGCTTAAGCAAAGGAAATCTGGCCGCTCTGCTGGGAAGTATGATGTGTAT
TTGATCAATCCCCAGGGAAAAGCCTTTCGCTCTAAAGTGGAGTTGATTGCGTACTTCGA
AAAGGTAGGCGACACATCCCTGGACCCTAATGATTTTGACTTCACGGTAACTGGGAGAG
GGAGCCCCTCCCGGCGAGAGCAGAAACCACCTAAGAAGCCCAAATCTCCCAAAGCTCC
AGGAACTGGCAGAGGCCGGGGACGCCCCAAAGGGAGCGGCACCACGAGACCCAAGGC
GGCCACGTCAGAGGGTGTGCAGGTGAAAAGGGTCCTGGAGAAAAGTCCTGGGAAGCT
CCTTGTCAAGATGCCTTTTCAAACTTCGCCAGGGGGCAAGGCTGAGGGGGGTGGGGCC

ACCACATCCACCCAGGTCATGGTGATCAAACGCCCCGGCAGGAAGCGAAAAGCTGAGG
CCGACCCTCAGGCCATTCCCAAGAAACGGGGCCGAAAGCCGGGGAGTGTGGTGGCAGC
CGCTGCCGCCGAGGCCAAAAAGAAAGCCGTGAAGGAGTCTTCTATCCGATCTGTGCAG
GAGACCGTACTCCCCATCAAGAAGCGCAAGACCCGGGAGACGGTCAGCATCGAGGTCA
AGGAAGTGGTGAAGCCCCTGCTGGTGTCCACCCTCGGTGAGAAGAGCGGGAAAGGAC
TGAAGACCTGTAAGAGCCCTGGGCGGAAAAGCAAGGAGAGCAGCCCCAAGGGGCGCA
GCAGCAGCGCCTCCTCACCCCCCAAGAAGGAGCACCACCACCATCACCACCACTCAGA
GTCCCCAAAGGCCCCCGTGCCACTGCTCCCACCCCTGCCCCCACCTCCACCTGAGCCCG
AGAGCTCCGAGGACCCCACCAGCCCCCCTGAGCCCCAGGACTTGAGCAGCAGCGTCTG
CAAAGAGGAGAAGATGCCCAGAGGAGGCTCACTGGAGAGCGACGGCTGCCCCAAGGA
GCCAGCTAAGACTCAGCCCGCGGTTGCCACCGCCGCCACGGCCGCAGAAAAGTACAAA
CACCGAGGGGAGGGAGAGCGCAAAGACATTGTTTCATCCTCCATGCCAAGGCCAAACA
GAGAGGAGCCTGTGGACAGCCGGACGCCCGTGACCGAGAGAGTTAGCTGA

>ENSG00000184009|ENST00000331925

ATGGAAGAAGAGATCGCCGCGCTGGTCATTGACAATGGCTCCGGCATGTGCAAAGCTGG
TTTTGCTGGGGACGACGCTCCCCGAGCCGTGTTTCCTTCCATCGTCGGGCGCCCCAGAC
ACCAGGGCGTCATGGTGGGCATGGGCCAGAAGGACTCCTACGTGGGCGACGAGGCCCA
GAGCAAGCGTGGCATCCTGACCCTGAAGTACCCCATTGAGCATGGCATCGTCACCAACT
GGGACGACATGGAGAAGATCTGGCACCACACCTTCTACAACGAGCTGCGCGTGGCCCC
GGAGGAGCACCCAGTGCTGCTGACCGAGGCCCCCCTGAACCCCAAGGCCAACAGAGA
GAAGATGACTCAGATTATGTTTGAGACCTTCAACACCCCGGCCATGTACGTGGCCATCCA
GGCCGTGCTGTCCCTCTACGCCTCTGGGCGCACCACTGGCATTGTCATGGACTCTGGAG
ACGGGGTCACCCACACGGTGCCCATCTACGAGGGCTACGCCCTCCCCCACGCCATCCTG
CGTCTGGACCTGGCTGGCCGGGACCTGACCGACTACCTCATGAAGATCCTCACTGAGCG
AGGCTACAGCTTCACCACCACGGCCGAGCGGGAAATCGTGCGCGACATCAAGGAGAAG
CTGTGCTACGTCGCCCTGGACTTCGAGCAGGAGATGGCCACCGCCGCATCCTCCTCTTC
TCTGGAGAAGAGCTACGAGCTGCCCGATGGCCAGGTCATCACCATTGGCAATGAGCGGT
TCCGGTGTCCGGAGGCGCTGTTCCAGCCTTCCTTCCTGGGTATGGAATCTTGCGGCATCC
ACGAGACCACCTTCAACTCCATCATGAAGTGTGACGTGGACATCCGCAAAGACCTGTAC
GCCAACACGGTGCTGTCGGGCGGCACCACCATGTACCCGGGCATTGCCGACAGGATGC
AGAAGGAGATCACCGCCCTGGCGCCCAGCACCATGAAGATCAAGATCATCGCACCCCC
AGAGCGCAAGTACTCGGTGTGGATCGGTGGCTCCATCCTGGCCTCACTGTCCACCTTCC
AGCAGATGTGGATTAGCAAGCAGGAGTACGACGAGTCGGGCCCCTCCATCGTCCACCGC
AAATGCTTCTAA

>ENSG00000198792|ENST00000403210

ATGCACCTGCGCTGCTACAGCTGCCCCAACGAGCAGCGCTACATCGTGCGCATCCTCTT
CATCGTGCCCATCTACGCCTTTGACTCCTGGCTCAGCCTCCTCTTCTTCACCAACGACCA
GTACTACGTGTACTTCGGCACCGTCCGCGACTGCTATGAGGCCTTGGTCATCTATAATTTC
CTGAGCCTGTGCTATGAGTACCTAGGAGGAGAAAGTTCCATCATGTCGGAGATCAGAGG
AAAACCCATTGAGTCCAGCTGTATGTATGGCACCTGCTGCCTCTGGGGAAAGACTTATTC
CATCGGATTTCTGAGGTTCTGCAAACAGGCCACCCTGCAGTTCTGTGTGGTGAAGCCAC
TCATGGCGGTCAGCACTGTGGTCCTCCAGGCCTTCGGCAAGTACCGGGATGGGGACTTT
GA

>ENSG00000198792|ENST00000411679

ATGACAGTGAGGGGGGGATGTGCTGGCCCCGGATCCAGCGTCGCCCACGACCGCAGCAG
CCTCGCCCAGCGTCTCCGTGATCCCCGAGGGCAGCCCCACTGCCATGGAGCAGCCTGTG

TTCCTGATGACAACTGCCGCTCAGGCCATCTCTGGCTTCTTCGTGTGGACGGCCCTGCTC
ATCACATGCCACCAGCCCAGGGCTCGGCTGGGCCAAGTGTGA

>ENSG00000198792|ENST00000436674

ATGACAGTGAGGGGGGATGTGCTGGCCCCGGATCCAGCGTCGCCCACGACCGCAGCAG
CCTCGCCCAGCGTCTCCGTGATCCCCGAGGGCAGCCCCACTGCCATGGAGCAGCCTGTG
TTCCTGATGACAACTGCCGCTCAGGCCATCTCTGGCTTCTTCGTGTGGACGGCCCTGCTC
ATCACATGCCACCAGCCCAGGGCTCGGCTGGGCCAAGTGTGA

>ENSG00000198792|ENST00000457534

ATGACAGTGAGGGGGGATGTGCTGGCCCCGGATCCAGCGTCGCCCACGACCGCAGCAG
CCTCGCCCAGCGTCTCCGTGATCCCCGAGGGCAGCCCCACTGCCATGGAGCAGCCTGTG
TTCCTGATGACAACTGCCGCTCAGGCCATCTCTGGCTTCTTCGTGTGGACGGCCCTGCTC
ATCACATGCCACCAGAATCCCAGCATCTGCGGACCCATTCTGCTGATTTGA

>ENSG00000205542|ENST00000380633

ATGTCTGACAAACCCGATATGGCTGAGATCGAGAAATTCGATAAGTCGAAACTGAAGAA
GACAGAGACGCAAGAGAAAAATCCACTGCCTTCCAAAGAAACGATTGAACAGGAGAA
GCAAGCAGGCGAATCGTAA

>ENSG00000205542|ENST00000380635

ATGTCTGACAAACCCGATATGGCTGAGATCGAGAAATTCGATAAGTCGAAACTGAAGAA
GACAGAGACGCAAGAGAAAAATCCACTGCCTTCCAAAGAAACGATTGAACAGGAGAA
GCAAGCAGGCGAATCGTAA

>ENSG00000205542|ENST00000380636

ATGTCTGACAAACCCGATATGGCTGAGATCGAGAAATTCGATAAGTCGAAACTGAAGAA
GACAGAGACGCAAGAGAAAAATCCACTGCCTTCCAAAGAAACGATTGAACAGGAGAA
GCAAGCAGGCGAATCGTAA

>ENSG00000205542|ENST00000451311

ATGTCTGACAAACCCGATATGGCTGAGATCGAGAAATTCGATAAGTCGAAACTGAAGAA
GACAGAGACGCAAGAGAAAAATCCACTGCCTTCCAAAGAAACGATTGAACAGGAGAA
GCAAGCAGGCGAATCGTAA

>ENSG00000220205|ENST00000316509

ATGTCTGCTACCGCTGCCACGGCCCCCCCTGCTGCCCCGGCTGGGGAGGGTGGTCCCCC
TGCACCCCCTCCAAACCTCACCAGTAACAGGAGACTGCAGCAGACCCAGGCCCAGGTG
GATGAGGTGGTGGACATCATGAGGGTGAACGTGGACAAGGTCCTGGAGCGAGACCAGA
AGCTGTCGGAGCTGGACGACCGTGCAGATGCACTCCAGGCGGGGGCCTCCCAGTTTGA
AACAAGCGCAGCCAAGCTCAAGCGCAAATACTGGTGGAAAAACCTCAAGATGATGATC
ATCTTGGGAGTGATTTGCGCCATCATCCTCATCATCATCATAGTTTACTTCAGCACTTAA

>ENSG00000220205|ENST00000488857

ATGGACAGGTCTGCTACCGCTGCCACGGCCCCCCCTGCTGCCCCGGCTGGGGAGGGTG
GTCCCCCTGCACCCCCTCCAAACCTCACCAGTAACAGGAGACTGCAGCAGACCCAGGC
CCAGGTGGATGAGGTGGTGGACATCATGAGGGTGAACGTGGACAAGGTCCTGGAGCGA
GACCAGAAGCTGTCGGAGCTGGACGACCGTGCAGATGCACTCCAGGCGGGGGCCTCCC
AGTTTGAAACAAGCGCAGCCAAGCTCAAGCGCAAATACTGGTGGAAAAACCTCAAGAT
GATGATCATCTTGGGAGTGATTTGCGCCATCATCCTCATCATCATCATAGTTTACTTCAGC
ACTTAA

Other sources of datasets (References):

1 Heikham R, Shankar R: Flanking region sequence information to refine microRNA target predictions J Biosci 2010, 35:105–118.

2 Yang Y, Wang YP, Li KB: MiRTif: a support vector machine-based microRNA target interaction filter. BMC Bioinformatics 2008: 12,9

3 Dai X and Zhao PX: psRNATarget: a plant small RNA target analysis server. Nucleic Acids Res 2011, 1-5
