# Supplementary material for: Employing machine learning for reliable miRNA target identification in plants
Source: BMC Genomics. 2011 Dec 29;12:636. doi: 10.1186/1471-2164-12-636 (PMC3293931; doi:10.1186/1471-2164-12-636)
Supplement: Additional file 4 — miRNA groups and their corresponding functional category enrichments with p-values. miRNA targets in Rice transcriptome were grouped according to the miRNA targeting them and their associated GO functional categories for Molecular function and Biological processes. [file 1471-2164-12-636-S4.PDF]

GO functional group enrichments analysis on different miRNA target groups

Grp: miR-156 Biological Function

p-value	Description
5.4937E-5	response to stress
5.6993E-5	transcription

miR-156 Molecular Function

p-value	Description
8.5889E-9	DNA-directed RNA polymerase activity
1.4219E-8	RNA polymerase activity
4.0687E-8	catalytic activity
1.1674E-6	nucleotidyltransferase activity
8.3032E-5	transferase activity
1.0978E-4	hydrolase activity, hydrolyzing O-glycosyl compounds
1.5627E-4	hydrolase activity, acting on glycosyl bonds
1.8338E-4	transferase activity, transferring phosphorus-containing groups

miR-160 Molecular Function

p-value	Description
8.4213E-6	DNA-directed RNA polymerase activity
1.1342E-5	RNA polymerase activity
1.4413E-4	catalytic activity
1.5632E-4	nucleotidyltransferase activity
6.9552E-4	phosphomannomutase activity
6.9552E-4	phosphoribulokinase activity

miR-166 Molecular Function

p-value	Description
8.2499E-5	DNA-directed RNA polymerase activity
1.0055E-4	RNA polymerase activity
5.8184E-4	nucleotidyltransferase activity

miR-166 Biological process

p-value	Description
1.7617E-5	immune response
1.9835E-5	immune system process
2.2174E-4	response to stress
2.2211E-4	defense response
4.4831E-4	RNA secondary structure unwinding
4.4831E-4	Nucleic acid metabolic process

]

miR-172 Molecular function

p-value	Description
2.4532E-3	structural constituent of cytoskeleton
5.8011E-3	polygalacturonase activity

miR-396 Biological function

p-value	Description
1.0088E-5	DNA-directed RNA polymerase activity
1.3585E-5	RNA polymerase activity
5.6700E-5	catalytic activity
1.8672E-4	nucleotidyltransferase activity
2.431E-4	transferase activity, transferring phosporus-containing groups

miR-396 Molecular function

p-value	Description
1.3419E-5	transcription
1.4271E-4	immune response
1.6044E-4	immune system process
2.4321E-4	nucleic acid metabolic process
3.7306E-4	response to stress

miR-444 Biological function

p-value	Description
7.1687E-5	response to stress
3.1385E-4	regulation of cell adhesion

miR 444 Molecular Function

p-value	Description
7.3632E-4	levanase activity
7.3632E-4	brassinosteroid sulfotransferase activity
7.3632E-4	inositol-polyphosphate 5-phosphatase activity
1.1043E-3	inositol trisphosphate phosphatase activity
1.1043E-3	inositol orphosphatidyl inositol phosphatase acitivity
